# Dearomative (4 + 3) Cycloaddition Reactions of 3-Alkenylindoles and 3-Alkenylpyrroles to Afford Cyclohepta[*b*]indoles and Cyclohepta[*b*]pyrroles

**DOI:** 10.1021/acs.orglett.2c02983

**Published:** 2022-10-31

**Authors:** Ferdinand Taenzler, Jiasu Xu, Sudhakar Athe, Viresh H. Rawal

**Affiliations:** Department of Chemistry, University of Chicago, 5735 South Ellis Avenue, Chicago, Illinois 60637, United States

## Abstract

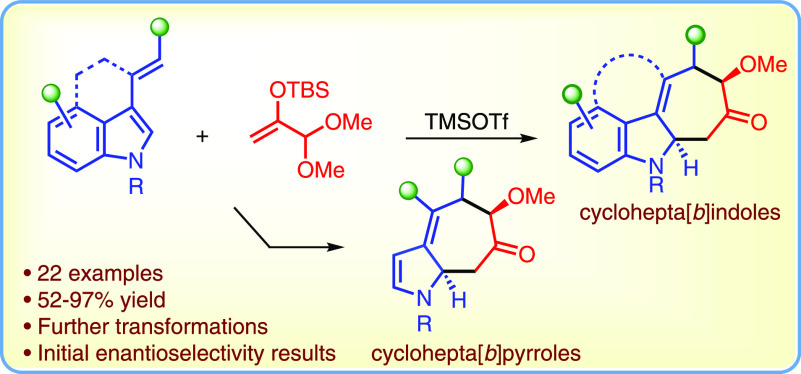

The dearomative (4 + 3) cycloaddition reactions of 3-alkenylindoles with in situ-generated oxyallyl cations furnish cyclohepta[*b*]indoles, functionality-rich frameworks found in many bioactive compounds, including all pentacyclic ambiguine alkaloids. The analogous reactions between oxyallyl cations and 3-alkenylpyrroles afford cyclohepta[*b*]pyrroles. The cycloadducts are generally formed in good to high yields and diastereoselectivities and can be readily transformed into useful derivatives. Additionally, we report preliminary investigations into the enantioselective catalysis of the dearomative (4 + 3) cycloaddition using imidodiphosphorimidate catalysts.

Fundamental heterocycles such as indoles and pyrroles are ubiquitous in natural products and compounds of biomedical interest.^1^ Their overall importance has stimulated numerous efforts directed at the efficient synthesis of common scaffolds containing these heterocycles. Our longstanding interest in the hapalindole family of cyanobacteria metabolites drew our attention to its pentacyclic ambiguine subset, exemplified by ambiguines P and G (Figure 1).^2−4^ Embedded in their complex architectures is a cyclohepta[*b*]indole unit, which is also present in many other natural products and in leads to pharmaceutical drugs, examples of which are shown in Figure 1. Indeed, due to its prevalence in bioactive compounds, cyclohepta[*b*]indole has been recognized as a “privileged” unit for drug design and has motivated the development of assorted methods for its synthesis.^5^ Inspired by the fundamental importance of this scaffold, we considered four different routes for its direct construction via dearomatizing (4 + 3) cycloaddition reactions of simple precursors, with each route conferring distinct capabilities for the synthesis of the ambiguines and other natural products (Figure 2).^6,7^

**Figure 1:**
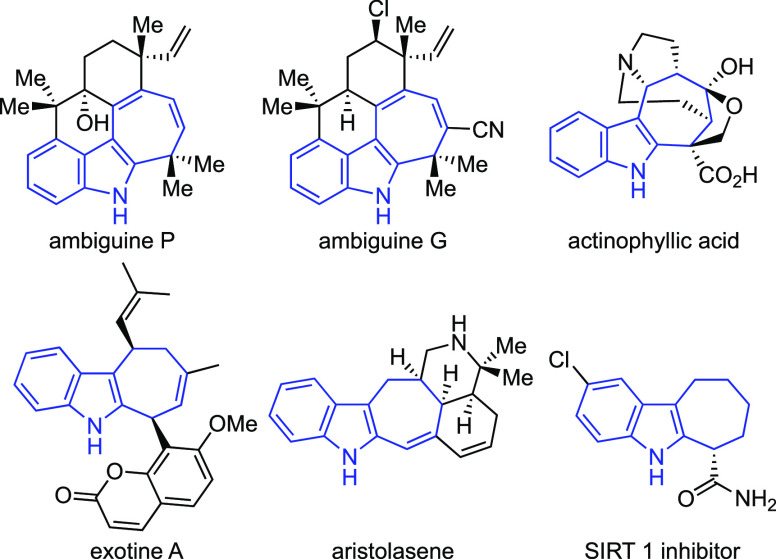
Selected cyclohepta[*b*]indole-containing compounds.

**Figure 2:**
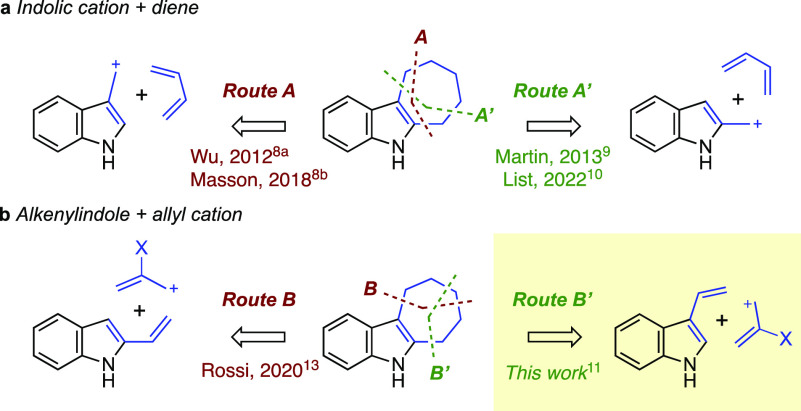
Conceptual (4 + 3) cycloaddition routes to cyclohepta[*b*]indoles.

The first pair of constructions involve the formal (4 + 3) cycloaddition between an indolyl cation and a diene. The (4 + 3) reaction of C3-indolyl cations (route A) was realized by Wu and co-workers, and its enantioselective version was developed by Masson et al. using Brønsted acid catalysis.^8^ The related reaction wherein the C2-indolyl cation is intercepted by a diene has also been studied, and it provided the basis for Martin’s elegant synthesis of actinophyllic acid as well as our recent syntheses of ambiguines P and G.^9,3b,4^ The chiral Brønsted acid-catalyzed enantioselective version of the reaction was recently reported by List et al.^10^ The second pair of disconnections (routes B and B′), which had not been reported when we commenced our studies, involve the reaction of a three-carbon dipole such as an oxyallyl cation or its equivalent with either 2- or 3-alkenyl indoles.^11,12^ Notably, in 2020, Rossi and co-workers reported a comprehensive study demonstrating the successful realization of route B.^13^ Given our interest in the ambiguines, we directed our attention to routes that offered the possibility for the direct introduction of the gem-dimethyl groups on the carbon attached to the indole C2 position. In this report we describe the (4 + 3) cycloadditions of oxyallyl species with a broad range of 3-alkenylindoles, which generate tri- and tetracyclic products possessing the cyclohepta[*b*]indoles, the core skeletal unit of the ambiguines.

To assess the feasibility of the planned cycloaddition reactions, we prepared tricyclic 3-alkenylindole **2a**, which possesses the skeletal features of the ambiguines, as a model substrate (Scheme 1). Ketone **1** was prepared in three steps from indole following known procedures.^14^ Whereas the methylenation of the carbonyl was slow and low-yielding when Wittig or Tebbe procedures (10–30%) were used, presumably due to steric hindrance and vinylogous amide-like reactivity of the carbonyl group, it proceeded well with the Nysted reagent to afford *N-*tosyl-3-alkenylindole **2a** in a good yield.^15^ The corresponding *N*-Me and *N*-Boc indoles **2c** and **2d**, respectively, were prepared by the removal of the tosyl group followed by methylation or Boc protection.^16^

**Scheme 1:**
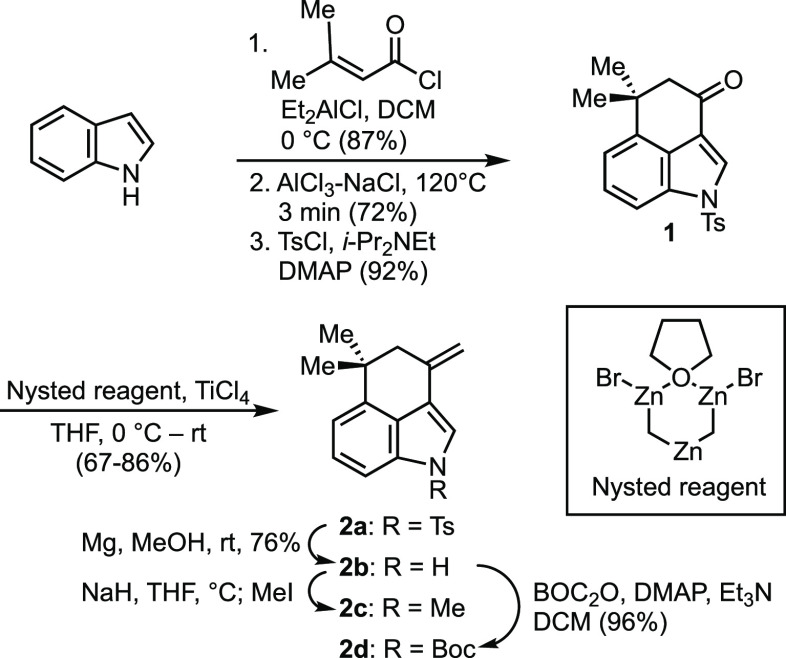
Synthesis of the 3-Alkenyindole Model Compound

Several different oxyallyl cation equivalents were examined for the key (4 + 3) cycloaddition, and the best results were obtained using dimethoxy silyl enol ether **3a**.^17^ The ease of the preparation and purification of such silyl enol ether acetals makes them useful and attractive oxyallyl cation precursors. Upon treatment with a Lewis acid, acetal **3a** is proposed to generate an α-oxygen-stabilized oxyallyl cation (cf. Figure 2b) that reacts with a diene to afford (4 + 3) cycloadducts upon desilylation. Various reagents were examined to promote the desired cycloaddition between indole **2a** and enol ether **3a** (Table 1). While metal-based Lewis acids have been used successfully for other dienes, most were not suitable for the present system. For example, SnCl_4_ caused the degradation of the reactants, whereas Sc(OTf)_3_ caused the proto-isomerization of the double bond of **2a** to the endocyclic position. The mild Lewis acid ZnCl_2_ did give the cycloadduct **4a** (50%), but it was accompanied by byproducts. On other hand, TMSOTf was found to cleanly furnish the desired (4 + 3) cycloadduct. A solvent screen was carried out to further improve the reaction outcome. Gratifyingly, the reaction proceeded cleanly and in nearly quantitative yield in THF and EtNO_2_ (entries 7 and 8, respectively), likely due to their ability to stabilize the in situ-generated oxyallyl cation.

**Table 1:**
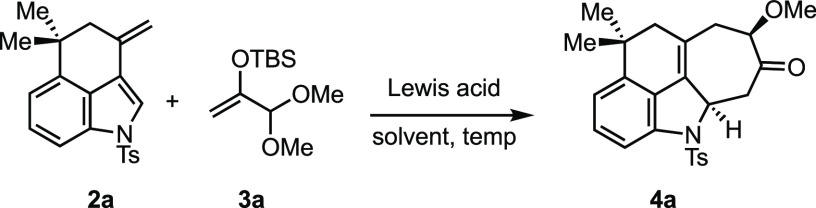
Lewis Acid-Promoted (4 + 3) Cycloaddition between Alkenylindole **2a** and Dimethyl Acetal **3a**

entry	Lewis acid (equiv)	solvent	temp.	yield (%)^b^
1	SnCl_4_ (1.0)	CH_2_Cl_2_	–78 °C	-c
2	ZnCl_2_ (1.1)	CH_2_Cl_2_	0 °C	50
3	TMSOTf (1.0)	CH_2_Cl_2_	–78 °C	67
4	TMSOTf (1.0)	PhMe	–78 °C	62
5	TMSOTf (1.0)	^*t*^BuOMe	–78 °C	54
6	TMSOTf (1.0)	Et_2_O	–78 °C	85
**7**	**TMSOTf (1.0)**	**THF**	**–78 °C**	**97 (91)**^d^
8	TMSOTf (1.0)	EtNO_2_	–78 °C	95

a Reactions were performed with 13–15 mg of alkenylindole **2a** (0.04M) and 1.0–1.5 equiv of **3a**.

b Yields were determined by NMR with 1,3,5-trimethoxybenzene as an internal standard.

c Decomposition of the starting materials.

d Isolated yield.

These optimized conditions were used to perform the (4 + 3) cycloaddition reaction on a slightly larger scale and to examine related cycloadditions. When the reaction was carried out with 79 mg of **2a** in THF, it provided tetracycle **4a** in a 91% isolated yield as a single diastereomer. The connectivity and relative stereochemistry of the (4 + 3) adduct were established unambiguously by X-ray crystallography (Figure 3a). Alkenylindole **2d**, which also possesses an electron-withdrawing group on the indole nitrogen, reacted well and gave the expected product (**4b**) in a high yield. On the other hand, **2b** and **2c** gave unsatisfactory results under the same conditions.^18^

**Figure 3:**
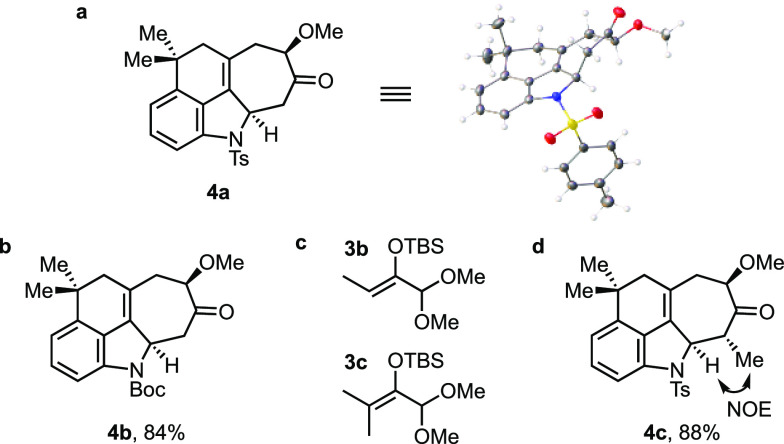
(a) X-ray structure of **4a**. (b) Cycloadduct of **2d**. (c) Methyl-substituted oxyallyl precursors. (d) Cycloadduct of **2a** and **3b**.

Of special interest, vis-à-vis the ambiguines, was the reaction of **2a** with the mono- and dimethyl derivatives of **3a** (**3b** and **3c**, respectively).^19^ We were pleased to find that **3b** reacted well under the standard conditions to give the methylated cycloadduct **4c** in an 88% yield. The relative stereochemistry shown is consistent with the observed NOE. The reaction of dimethyl oxyallyl precursor **3c** gave a complex mixture of isomeric uncyclized compounds along with a small amount of the expected cycloadduct as a mixture of diastereomers and was not explored further.

With suitable conditions in hand, the scope of the dearomative (4 + 3) cycloaddition reaction was examined next (Scheme 2). The substrates required for the cycloadditions were readily prepared through either acylation/methylenation of the parent indole, or oxidative coupling with suitable styrenes, or Suzuki cross-coupling with indole-3-boronic acid.^20^ A broad range of 3-alkenylindoles were examined, and all gave the (4 + 3) cycloadducts in good to high yields. Most reactions were performed in THF using 1.5 equiv of acetal **3a** to ensure the complete consumption of the alkenylindole. The reaction of 3-isopropenyl*-N*-tosyl-indole **5a** with acetal **3a** gave the expected cycloadduct **6a** in a 97% yield as essentially a single diastereomer. The relative stereochemistry in **6a** and other cycloadducts was assigned by analogy to that observed in **4a**. A comparable result was obtained with just 1.2 equiv of the oxyallyl precursor. Although the reaction worked well in EtNO_2_, it gave the product in significantly lower dr, ∼2:1. Interestingly, with the TMS analog of **3a**, just 10 mol % TMSOTf was enough to promote the reaction to >60% conversion, supporting a catalytic pathway for the silyl-triflate. A variety of substituted 3-isopropenylindole substrates possessing alkyl, halogen, or alkoxy substituents on the benzene ring were examined, and all gave the (4 + 3) cycloadducts in high yields (**6c**–**6j**). *N*-Boc-3-isopropenylindole (**5b**) also reacted well, but it gave the cycloadduct (**6b**) in a slightly lower yield.

**Scheme 2:**
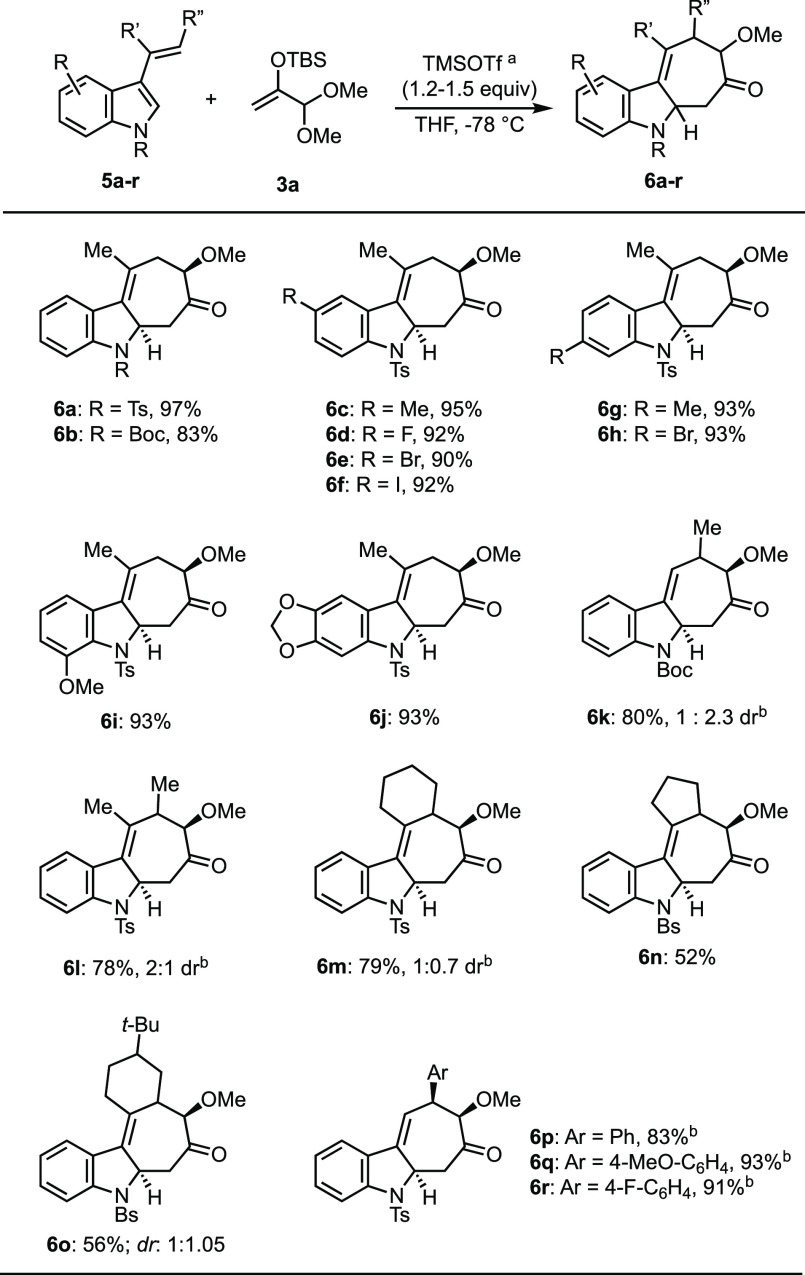
Scope of Dearomative (4 + 3) Cycloadditions between Alkenylindoles and Dimethyl Acetal **3a** See the Supporting Information for a general procedure. Yields given are for the pure isolated compounds. EtNO_2_ was used as the solvent.

Substrates that experience A^1,3^-like strain when the vinyl unit and the indole C2–C3 bond are in a planar *s-cis* orientation were ineffective in the cycloaddition reaction. Thus, 3-(1-phenylethenyl)-*N*-tosylindole and derivatives of **5a** having a methyl group at either the 2- or 4-position of the indole gave no cycloadduct. Similarly, with 3-(1-propenyl)-*N*-Boc-indole, which was prepared as a mixture of *E*- and *Z*-isomers, only the *E*-isomer was reactive, giving tricycle **6k** in an 80% yield.^21^ It is worth noting that alkenylindoles substituted on both alkene carbons were effective substrates, giving the expected cycloadducts (**6l**–**6o**) in good yields. Three styrylindoles were examined (**6p**–**6r**), and all afforded the cycloadducts in good to excellent yields as single diastereomers.

The cycloadducts from the (4 + 3) reaction are well-functionalized for further elaboration, as summarized below (Scheme 3). Acid-catalyzed isomerization of the double bond in the (4 + 3) cycloadducts was expected to reform the indole unit.^22^ Indeed, upon stirring in TFA/CH_2_Cl_2_ at room temperature, cycloadduct **4a** slowly isomerized to afford tetracyclic indole **9**, which was formed as a single diastereomer in a 51% yield (77% brsm). No improvement was seen with other commonly used isomerization reagents (e.g., PTSA, CSA, RhCl_3_, and Fe(OTf)_3_). On the other hand, the isomerization took place rapidly and cleanly with in situ-generated HI,^22b^ giving **9** in an 85% yield.

**Scheme 3:**
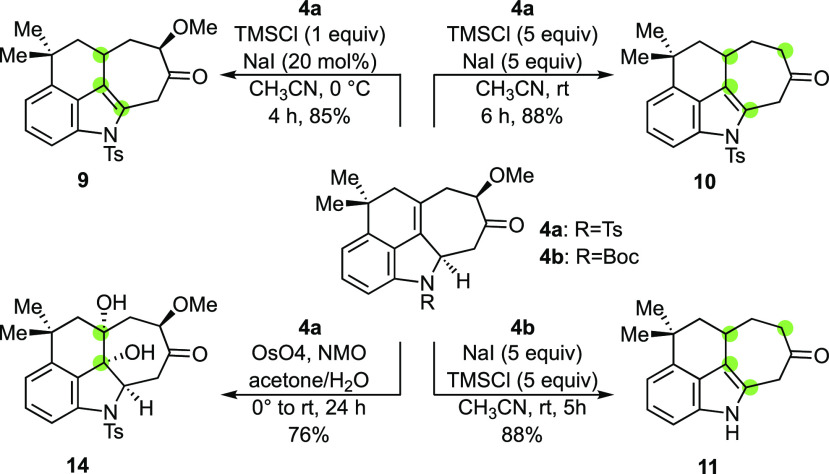
Useful Derivatization of (4 + 3) Cycloadducts

Remarkably, treating **4a** with an excess of trimethylsilyl chloride and sodium iodide effected the isomerization as well as the reductive removal of the α-methoxy group to give ketone **10** in an 88% yield. Subjecting tricycle **6a** to similar conditions gave the corresponding isomerized and demethoxylated ketone (71%).^20^ The deoxygenation is believed to go through an α-iodoketone, which is reductively deiodinated with the formation of molecular iodine.^22d^ Importantly, under analogous conditions, the Boc-protected cycloadduct **4b** afforded the deprotected, isomerized, and deoxygenated tetracycle **11** in an excellent yield. Dihydroxylation of cycloadduct **4a** using OsO_4_ proceeded with excellent selectivity, giving diol **14** as a single diastereomer in a 76% yield (Scheme 3).

While this investigation was inspired by the ambiguines and focused on 3-alkenylindoles, we also examined 3-alkenylpyrrole substrates (**7a**–**7d**) (Scheme 4) and found that they participated in the dearomative (4 + 3) reaction, affording the corresponding cycloadducts (**8a**–**8d**) in good yields^23^ Further expansion of this cycloaddition chemistry, including the use of other oxyallyl species and other heterocycles, should provide rapid access to assorted novel ring systems.

**Scheme 4:**
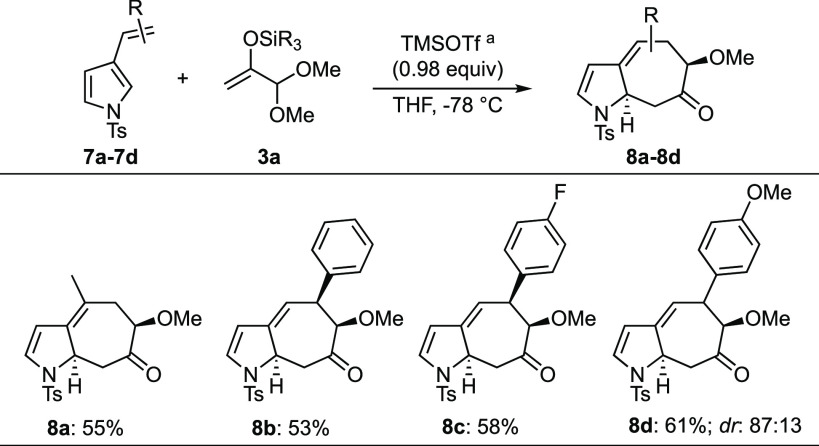
Dearomative (4 + 3) Cycloadditions between Alkenylpyrroles and TBS Enol Ether Dimethyl Acetal **3a** See the Supporting Information for a detailed procedure. Yields given are for the pure isolated compounds.

Lastly, while screening Brønsted and Lewis acids as promoters for the (4 + 3) cycloaddition of **2a** and **3a**, we observed that trifluoromethanesulfonic acid was also effective, albeit not as good as TMSOTf. However, this observation suggested the possibility of performing an enantioselective cycloaddition reaction using a chiral Brønsted acid as a catalyst or activator.^10,24^ A preliminary examination revealed that common chiral phosphoric acids or *N*-triflyl phosphoramides did not promote the cycloaddition, possibly because they were not sufficiently acidic to induce the generation of the requisite oxocarbenium ion intermediate. On the other hand, we were delighted to observe that the more acidic chiral imidodiphosphorimidates (IDPi) developed by List and co-workers^10b^ promoted the cycloaddition when just 5 mol % of the catalyst was used (Scheme 5). A screen of different catalysts showed that IDPi/Ph promoted the cycloaddition to give the adduct in a modest yield (87% brsm) and 55% ee (see the Supporting Information). These preliminary results bode well for the development of highly enantioselective (4 + 3) cycloaddition reactions of these and related substrates.

**Scheme 5:**
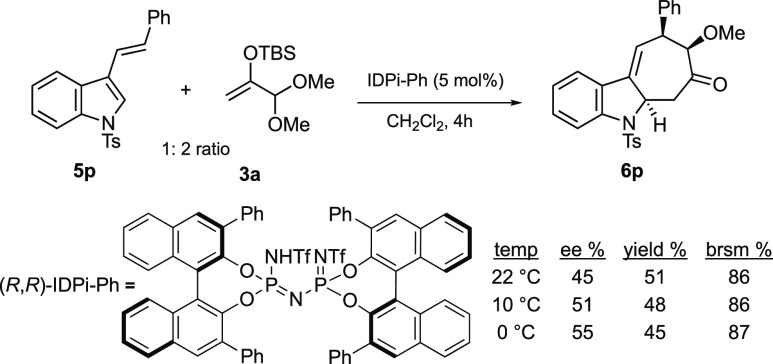
Enantioselective (4 + 3) Reaction of **5p** with **3a**

In summary, we have developed metal-free TMSOTf-mediated (4 + 3) cycloaddition reactions of alkenylindoles and alkenylpyrroles with oxyallyl cations to afford the privileged cyclohepta[*b*]indoles and cyclohepta[*b*]pyrroles in high yields and diastereoselectivities. The present method allows the one-step construction of the structurally complex core skeletons present in many bioactive natural products. The application of the (4 + 3) cycloaddition to the synthesis of ambiguines and the further development of the enantioselective reaction will be reported in due course.

## Supporting Information Available

Experimental procedures, characterization data, and NMR spectra (PDF)
