# Supplementary material for: Dearomative (4 + 3) Cycloaddition Reactions of 3-Alkenylindoles and 3-Alkenylpyrroles to Afford Cyclohepta[b]indoles and Cyclohepta[b]pyrroles
Source: Org Lett. 2022 Oct 31;24(44):8109–14. doi: 10.1021/acs.orglett.2c02983 (PMC9664489; doi:10.1021/acs.orglett.2c02983)
Supplement: Supplementary file 1 — ol2c02983_si_001.pdf [file ol2c02983_si_001.pdf]

Supporting Information for

Dearomative (4 + 3) cycloaddition reactions of 3-alkenylindoles and 3-alkenylpyrroles to afford cyclohepta[*b*]indoles and cyclohepta[*b*]pyrroles

Ferdinand Taenzler, Jiasu Xu, Sudhakar Athe, and Viresh H. Rawal*

Department of Chemistry, The University of Chicago

5735 South Ellis Avenue, Chicago, IL 60637

*Correspondence to: vrawal@uchicago.edu

Table of Contents

1. Material and Methods S2
2. Experimental Procedures and Characterization Data S3
3. Procedure for large scale synthesis of 4a........ S53
4. References........ S54
5. Experimental Spectra........ S55
6. X-ray Crystallographic Data for **4a** S187
7. Abstract from 2019 ACS National Meeting Presentation........ S203

1. Materials and Methods

Unless stated otherwise, reactions were performed under a nitrogen atmosphere using oven- or flame-dried glassware and stir bars. Ambient temperature refers to 22–24 °C. Pre-heated oil baths were used to maintain temperatures above ambient. Lower temperatures were maintained using acetone/CO_2(s) (−78 °C), MeCN/CO_2(s) (−40 °C) and water/ice (0 °C) baths. Dichloromethane (CH_2Cl_2 or DCM), tetrahydrofuran (THF), and dimethylformamide (DMF) were dried by passage through an activated alumina column purification system (Innovative Technology Inc. Pure Solv^{TM}). Commercially obtained reagents were used as received, unless stated otherwise.

Thin-layer chromatography (TLC) was performed using EMD Millipore silica gel 60 Å plates with UV fluorescence quenching (254 nm), KMnO4, or Seebach's stain. Flash column chromatography was performed on SiliCycle SiliaFlash P60 (40-63 µm particle size) using ACS or HPLC grade solvents purchased from Fisher Scientific. ^{1}H NMR spectra were recorded on Bruker 500 spectrometers (at 500 MHz) at 294-297 °K. ^{13}C NMR spectra were recorded on Bruker 500 and Bruker 400 spectrometers (at 125 MHz and 100 MHz, respectively) at 294-297 K. ^{1}H spectra were calibrated from internal standard TMS (δ 0.0). ^{13}C spectra were calibrated from solvent resonance ($CDCl_3$: δ 77.16). NMR data are reported as: chemical shift (δ ppm) (multiplicity, coupling constant (Hz), and integration). High-resolution mass spectral analysis was measured on Agilent Technologies 6224 TOF LC/MS (electrospray ionization). IR spectra were recorded on a Thermo Scientific Nicolet iS50 FT-IR spectrometer and are reported as frequency of absorption (cm^{-1}).

2. Experimental Procedures and Characterization Data

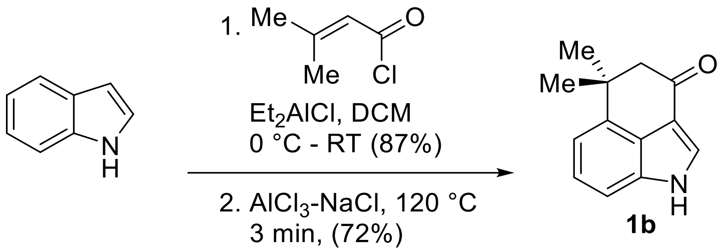

5,5-dimethyl-4,5-dihydrobenzo[cd]indol-3(1*H*)-one (1b). Prepared according to the literature reports.[1, 2, 3]

^{1}H NMR (500 MHz, CDCl$_3$): δ 8.75 (s, 1H), 7.74 (d, J = 2.8 Hz, 1H), 7.31 – 7.28 (m, 2H), 7.19 (t, J = 3.9 Hz, 1H), 2.76 (s, 2H), 1.44 (s, 6H).

^{13}C NMR (100 MHz, CDCl$_3$): δ 194.7, 139.0, 133.9, 127.8, 124.7, 123.7, 116.1, 114.5, 109.6, 55.9, 39.3, 29.5.

HRMS (ESI): calcd for $C_{13}H_{14}NO^+$ [M+H]$^+$: 200.1070, found: 200.1072.

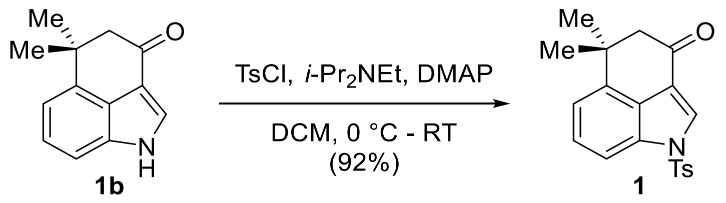

5,5-dimethyl-1-tosyl-4,5-dihydrobenzo[*cd*]indol-3(1*H*)-one (1). A 50 mL flask with a magnetic stir bar was charged with tricyclic ketone **1b** (407 mg, 2.04 mmol, 1 equiv) and 10 mL DCM to afford a yellow solution. *i*-Pr$_2$NEt (0.569 mL, 3.26 mmol, 1.6 equiv) was added, and the mixture was cooled to 0 °C. DMAP (12.5 mg, 0.102 mmol, 0.05 equiv) and TsCl (467 mg, 2.45 mmol, 1.2 equiv) were added sequentially. After 15 min, the cold bath was removed, and the reaction mixture was stirred at ambient temperature for 1.5 h. The mixture was quenched with 6 mL saturated aqueous $NaHCO_3$, and partitioned between 20 mL 1:1 brine:H_2O and 20 mL DCM. The aqueous layer was extracted with DCM (15 mL × 2), and the combined organic layers were dried over Na_2SO_4 and concentrated under reduced pressure. Purification of the crude product by flash chromatography (20:80 EtOAc:hexanes) provided **1** (661 mg, 92% yield) as a white solid.

^{1}H NMR (500 MHz, CDCl$_3$): δ 8.04 (s, 1H), 7.87 (d, *J* = 8.4 Hz, 2H), 7.78 (d, *J* = 8.3 Hz, 1H), 7.39 (t, *J* = 7.4 Hz,1H), 7.30 (d, *J* = 8.1 Hz, 2H), 7.27 – 7.23 (m, 1H), 2.70 (s, 2H), 2.38 (s, 3H), 1.37 (s, 6H).

^{13}C NMR (100 MHz, CDCl$_3$): δ 194.2, 146.0, 139.8, 134.9, 133.3, 130.4, 128.9, 127.4, 126.7, 124.1, 118.7, 118.0, 111.6, 55.9, 39.3, 29.5, 21.8.

HRMS (ESI): calcd for $C_{20}H_{20}NO_3S^+$ $[M+H]^+$: 354.1158, found: 354.1165.

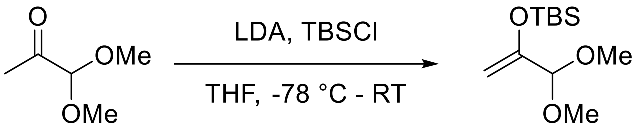

3a

tert-butyl((3,3-dimethoxyprop-1-en-2-yl)oxy)dimethylsilane (3a).

To a stirred solution of diisopropylamine (4.34 mL, 31.0 mmol, 1.2 equiv) in anhydrous THF (100 mL) at -78 °C was added *n*-BuLi (18.75 mL, 30.0 mmol, 1.6 M in hexanes, 1.2 equiv). After 30 min, a solution of methylglyoxal 1,1-dimethyl acetal (2.95 g, 25.0 mmol, 1 equiv) in anhydrous THF (5 mL) was added dropwise. The reaction was allowed to proceed for 1h, whereupon a solution of TBSCl (4.14 g, 27.5 mmol, 1.1 equiv) in anhydrous THF (10 mL) was added by syringe. The reaction was then allowed to warm slowly to room temperature and left overnight. Then, the reaction mixture was diluted with hexanes (100 mL), and the resulting turbid reaction mixture was filtered through a pad of Celite. The filtrate was concentrated and purified by column chromatography (5:95 EtOAc:hexanes) to give **3a** (4.58 g, 79%) as a colorless oil.

^{1}H NMR (500 MHz, CDCl$_3$): δ 4.56 (s, 1H), 4.56 (d, *J* = 1.1 Hz, 1H), 4.33 (d, *J* = 1.3 Hz, 1H), 3.33 (s, 6H), 0.93 (s, 9H), 0.18 (s, 6H).

^{13}C NMR (100 MHz, CDCl$_3$): δ 153.6, 102.1, 92.6, 53.2, 25.7, 18.2, −4.6.

HRMS (ESI): calcd for $C_{11}H_{25}O_3Si^+$ $[M+H]^+$: 233.1567, found: 233.1555.

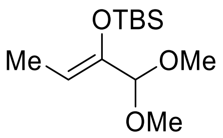

3b

(*Z*)-*tert*-butyl((1,1-dimethoxybut-2-en-2-yl)oxy)dimethylsilane 3b. Prepared according to the literature procedure.[4]

^{1}H NMR (500 MHz, $CDCl_3$): δ 5.01 (qd, *J* = 6.8, 0.8 Hz, 1H), 4.50 (s, 1H), 3.31 (s, 6H), 1.60 (dd, *J* = 6.8, 0.9 Hz, 3H), 0.96 (s, 9H), 0.14 (s, 6H).

^{13}C NMR (100 MHz, $CDCl_3$): δ 146.0, 105.3, 103.0, 53.3, 26.0, 18.6, 10.4, −3.9.

HRMS (ESI): calcd for $C_{12}H_{27}O_3Si^+$ $[M+H]^+$: 247.1724, found: 247.1707.

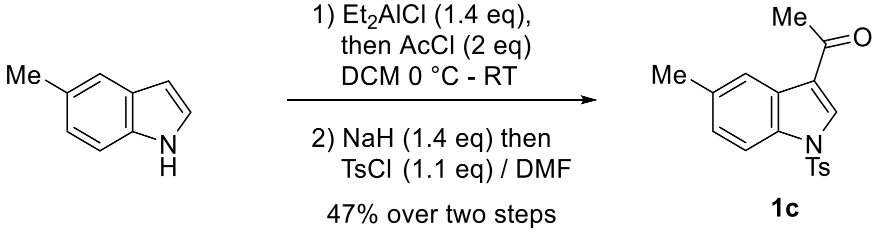

1c

1-(5-methyl-1-tosyl-1H-indol-3-yl)ethan-1-one (1c)

Representative Procedure for synthesis of Acetyl Indoles: **Procedure A**

A 50 mL round-bottomed flask with a magnetic stir bar was charged with 5-methylindole (327 mg, 2.5 mmol, 1 equiv) and 10 mL DCM to afford a colorless solution, which was cooled to 0 °C. Et_2AlCl solution (1.0 M in hexanes; 3.5 mL, 3.5 mmol, 1.4 equiv) was added over 5 min, and the mixture was stirred at 0 °C for 35 min. Then a solution of acetyl chloride (0.36 mL, 5 mmol, 2 equiv) in DCM (1 mL) was added over 10 min, whereupon the mixture turned orange. After stirring for 2 h at 0 °C, the reaction mixture was quenched with 50 mL saturated aqueous $NaHCO_3$ and allowed to warm to ambient temperature. The mixture was partitioned with 30 mL DCM, and the aqueous layer was extracted with DCM (20 mL × 2). The combined organic layers were dried over Na_2SO_4, filtered, and concentrated under reduced pressure to furnish the 3-acetyl indole as a tan

solid that was clean enough to proceed to the next step. Crude acetyl indole was dissolved in 25 mL DMF and treated with NaH (60% dispersion in oil; 140 mg, 3.5 mmol, 1.4 equiv). The reaction was allowed to stir for 1h under nitrogen, during which hydrogen evolution was observed. Tosyl chloride (524 mg, 2.75 mmol, 1.1 equiv) was added in one portion, and the reaction left to stir for 2h. The reaction mixture was then poured into 100 mL water and extracted with ethyl acetate (25 mL x 4). The combined organic layers were diluted with hexanes (100 mL) and then washed with water (25 mL x 4) and brine (25 mL x 1). The combined organic layers were dried over Na_2SO_4 and concentrated under reduced pressure. Purification by flash chromatography (1:2 EtOAc:hexanes) provided indole **1c** (384 mg, 47% yield) as an off white solid.

^{1}H NMR (400 MHz, $CDCl_3$): δ 8.16 (s, 1H), 8.12 (s, 1H), 7.80 (overlapping d & s, 3H), 7.27 (d, *J* = 8.1 Hz, 2H), 7.18 (dd, *J* = 8.5, 1.8 Hz, 1H), 2.56 (s, 3H), 2.43 (s, 3H), 2.36 (s, 3H).

^{13}C NMR (101 MHz, $CDCl_3$): δ 193.6, 145.8, 134.7, 134.6, 133.1, 132.3, 130.2, 127.7, 127.1, 127.1, 122.8, 121.4, 112.6, 27.8, 21.6, 21.4.

HRMS (ESI): calcd for $C_{18}H_{18}NO_3S^+$ $[M+H]^+$: 328.1002, found: 328.0993.

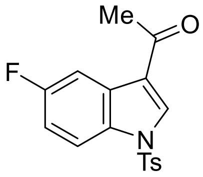

1d

1-(5-fluoro-1-tosyl-1H-indol-3-yl)ethan-1-one (1d)

Prepared using **Procedure A:** 5-fluoro indole (405 mg, 3.0 mmol, 1equiv) was subjected to general **Procedure A** to obtain **1d** (194.8 mg, 42%) as a white solid.

^{1}H NMR (400 MHz, $CDCl_3$): δ 8.22 (s, 1H), 8.01 (dd, *J* = 9.1, 2.6 Hz, 1H), 7.86 (dd, *J* = 8.6, 4.3 Hz, 1H), 7.82 (d, *J* = 8.6 Hz, 2H), 7.30 (d, *J* = 8.3 Hz, 2H), 7.10 (td, *J* = 9.0, 2.7 Hz, 1H), 2.56 (s, 3H), 2.38 (s, 3H).

^{13}C NMR (101 MHz, $CDCl_3$): δ 193.1, 161.7, 159.3, 146.2, 134.3, 133.3, 131.2, 130.3, 128.7, 128.6, 127.1, 121.4, 121.3, 114.2, 114.1, 114.1, 113.8, 109.1, 108.8, 27.6, 21.6.

HRMS (ESI): calcd for $C_{17}H_{15}FNO_3S^+$ $[M+H]^+$: 332.0751, found: 332.0748.

m.p: 163 – 164.5 °C

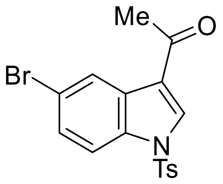

1e

1-(5-bromo-1-tosyl-1H-indol-3-yl)ethan-1-one (1e)

Prepared using **Procedure A:** 5-bromo indole (588 mg, 3.0 mmol, 1 equiv) was subjected to general **Procedure A** to affording **1e** (497 mg, 76%) as a white solid.

^{1}H NMR (400 MHz, CDCl$_3$): δ 8.50 (d, *J* = 1.8 Hz, 1H), 8.18 (s, 1H), 7.80 (t, *J* = 8.2 Hz, 3H), 7.51 – 7.43 (m, 1H), 7.30 (d, *J* = 8.0 Hz, 2H), 2.56 (s, 2H), 2.38 (s, 3H).

^{13}C NMR (101 MHz, CDCl$_3$): δ 193.0, 146.3, 134.2, 133.6, 132.8, 130.3, 129.1, 128.8, 127.1, 125.9, 120.8, 118.7, 114.4, 27.7, 21.7.

HRMS (ESI): calcd for $C_{17}H_{15}BrNO_3S^+$ $[M+H]^+$: 391.9951, found: 391.9942.

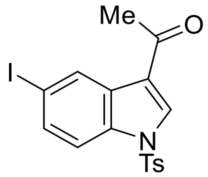

1f

1-(5-iodo-1-tosyl-1H-indol-3-yl)ethan-1-one (1f)

Prepared using **Procedure A:** 5-iodo indole (729 mg, 3.0 mmol, 1equiv) was subjected to general **Procedure A** to **1f** (458 mg, 57%) as a tan solid.

^{1}H NMR (400 MHz, CDCl$_3$): δ 8.70 (dd, *J* = 1.7, 0.7 Hz, 1H), 8.14 (s, 1H), 7.81 (d, *J* = 8.4 Hz, 2H), 7.67 – 7.65 (m, 2H), 7.30 – 7.26 (m, 2H), 2.55 (s, 3H) 2.38 (s, 3H).

^{13}C NMR (**101 MHz, CDCl$_3$**): δ 193.0, 146.3, 134.4, 134.2, 134.1, 132.5, 132.0, 130.3, 129.5, 127.1, 120.6, 114.8, 89.5, 30.9, 27.7, 21.7.

HRMS (ESI): calcd for $C_{17}H_{15}INO_3S^+$ $[M+H]^+$: 439.9812, found: 439.9801.

m.p: 166 –168 °C.

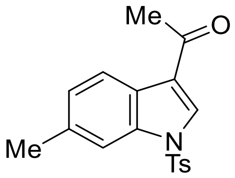

1g

1-(6-methyl-1-tosyl-1H-indol-3-yl)ethan-1-one (1g)

Prepared using **Procedure A:** 6-methyl indole (393.5 mg, 3.0 mmol, 1 equiv) was subjected to general **Procedure A** to **1g** (261.8 mg, 58%) as a tan solid.

^{1}H NMR (400 MHz, CDCl$_3$): δ 8.18 (d, *J* = 8.1 Hz, 1H), 8.12 (s, 1H), 7.82 (d, *J* = 8.4 Hz, 2H), 7.78 (s, 1H), 7.28 (d, *J* = 8.1 Hz, 2H), 7.18 (d, *J* = 8.2 Hz, 1H), 2.56 (s, 3H), 2.43 (s, 3H), 2.36 (s, 3H).

^{13}C NMR (101 MHz, CDCl$_3$): δ 193.5, 145.8, 136.0, 135.3, 134.6, 131.7, 130.2, 127.0, 126.4, 125.2, 122.6, 121.6, 113.0, 27.7, 21.9, 21.6.

HRMS (ESI): calcd for $C_{18}H_{18}NO_3S^+$ $[M+H]^+$: 328.1002, found: 328.0983.

m.p: 216 –218 °C.

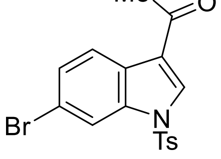

1h

1-(6-bromo-1-tosyl-1H-indol-3-yl)ethan-1-one (1h)

Prepared using **Procedure A:** 6-bromo indole (1.176 g, 6.0 mmol, 1equiv) was subjected to general **Procedure A** to obtain **1h** (715, 55%) as a tan solid.

^{1}H NMR (400 MHz, CDCl$_3$): δ 8.19 (d, *J* = 8.5 Hz, 1H), 8.15 (s, 1H), 8.10 (d, *J* = 1.7 Hz, 1H), 7.61 (d, *J* = 8.1 Hz, 2H), 7.45 (dd, *J* = 8.5, 1.7 Hz, 1H), 7.32 (d, *J* = 8.1 Hz, 2H), 2.55 (s, 3H), 2.40 (s, 3H).

^{13}C NMR (101 MHz, CDCl$_3$): δ 193.1, 146.3, 135.5, 134.2, 132.3, 130.4, 128.2, 127.1, 126.4, 124.3, 121.3, 119.6, 116.1, 27.7, 21.7.

HRMS (ESI): calcd for $C_{17}H_{15}BrNO_3S^+$ $[M+H]^+$: 391.9951, found: 391.9940.

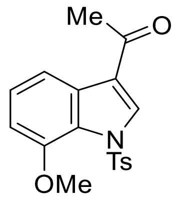

1i

1-(7-methoxy-1-tosyl-1H-indol-3-yl)ethan-1-one (1i)

Prepared using **Procedure A:** 7-methoxy indole (441.2 mg, 3.0 mmol, 1 equiv) was subjected to general **Procedure A** to obtain **1i** (170.9 mg, 34%) as a pale-yellow solid.

^{1}H NMR (400 MHz, CDCl$_3$): δ 8.49 (s, 1H), 7.99 (d, *J* = 7.9 Hz, 1H), 7.76 (d, *J* = 8.3 Hz, 2H), 7.30 (d, *J* = 8.2 Hz, 2H), 7.23 (t, *J* = 8.0 Hz, 1H), 6.74 (d, *J* = 7.9 Hz, 1H), 3.68 (s, 3H), 2.60 (s, 3H), 2.42 (s, 3H).

^{13}C NMR (101 MHz, CDCl$_3$): δ 193.5, 146.8, 145.0, 136.3, 134.8, 130.3, 129.5, 127.5, 125.7, 125.1, 119.9, 115.4, 107.8, 55.4, 27.8, 21.6.

HRMS (ESI): calcd for $C_{18}H_{18}NO_4S^+$ $[M+H]^+$: 344.0951, found: 344.0950.

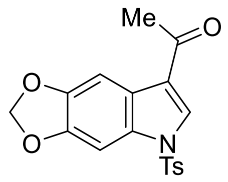

1j

1-(5-tosyl-5H-[1,3]dioxolo[4,5-f]indol-7-yl)ethan-1-one 1h (1j)

Prepared using **Procedure A:** 5H-[1,3]dioxolo[4,5-f]indole[5] (161 mg, 1.0 mmol, 1 equiv) was subjected to general **Procedure A** to obtain **1j** (67 mg, 37%) as a white solid.

^{1}H NMR (400 MHz, $CDCl_3$): δ 8.06 (s, 1H), 7.79 (d, *J* = 8.4 Hz, 2H), 7.72 (s, 1H), 7.39 (s, 1H), 7.29 (d, *J* = 8.2 Hz, 2H), 5.99 (s, 2H), 2.52 (s, 3H), 2.39 (s, 3H).

^{13}C NMR (101 MHz, $CDCl_3$): δ 193.5, 147.2, 146.3, 145.9, 134.5, 130.7, 130.2, 129.8, 127.1, 121.9, 121.7, 101.8, 101.6, 94.4, 27.5, 21.6.

HRMS (ESI): calcd for $C_{18}H_{16}NO_5S^+$ $[M+H]^+$: 358.0744, found: 358.0734.

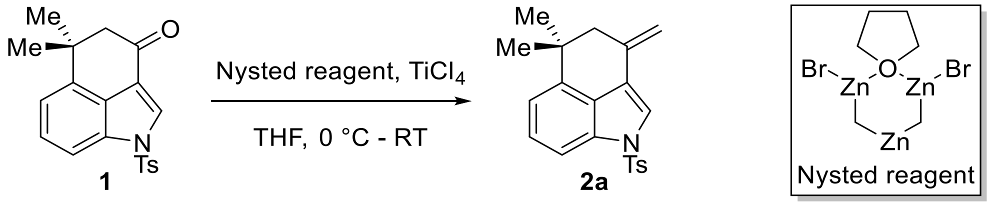

Representative Procedure for Synthesis of Alkenes using Nysted's reagent: **Procedure B**

5,5-dimethyl-3-methylene-1-tosyl-1,3,4,5-tetrahydrobenzo[*cd*]indole (2a). A 100 mL flask with a magnetic stir bar was charged with 12 mL THF and cooled to 0 °C. Nysted reagent solution (20 wt% in THF; 4.71 mL, 2.45 mmol, 1.1 equiv) was added, forming a white suspension. $TiCl_4$ (0.269 mL, 2.45 mmol, 1.1 equiv) was added dropwise, and the suspension turned purple. After 10 min, a solution of *N*-tosyl indole **1** (788 mg, 2.23 mmol, 1 equiv) in 10 mL THF was added dropwise. Then the cold bath was removed, and the grey slurry was stirred at ambient temperature for 2 h. The mixture was carefully quenched with 50 mL 1 M HCl, and partitioned between 20 mL H_2O and 30 mL 1:1 Et_2O:hexanes. The aqueous layer was extracted with 30 mL 1:1 Et_2O:hexanes, and the combined organic layers were washed with brine, dried over Na_2SO_4,

and concentrated under reduced pressure. Purification of the crude product by flash chromatography (10:90 EtOAc:hexanes) provided tricyclic alkene **2a** (521 mg, 66%) as a white solid.

^{1}H NMR (500 MHz, CDCl$_3$): δ 7.81 (d, *J* = 8.4 Hz, 2H), 7.72 (d, *J* = 8.2 Hz, 1H), 7.54 (s, 1H), 7.28 (t, *J* = 7.8 Hz, 1H), 7.24 (d, *J* = 7.9 Hz, 2H), 7.13 (d, *J* = 7.4 Hz, 1H), 5.44 (d, *J* = 1.4 Hz, 1H), 5.09 (d, *J* = 1.4 Hz, 1H), 2.45 (s, 2H), 2.35 (s, 3H), 1.27 (s, 6H).

^{13}C NMR (100 MHz, CDCl$_3$): δ 145.0, 141.4, 136.0, 135.7, 133.5, 130.1, 127.9, 127.0, 125.7, 120.5, 117.55, 117.47, 111.1, 110.4, 48.5, 36.3, 28.8, 21.7.

HRMS (ESI): calcd for $C_{21}H_{22}NO_2S^+$ [M+H]$^+$: 352.1366, found: 352.1359.

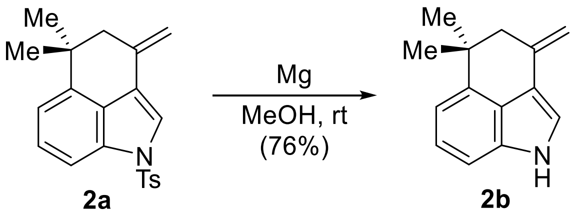

5,5-dimethyl-3-methylene-1,3,4,5-tetrahydrobenzo[*cd*]indole (2b).

To a 20 mL vial containing a stirbar was added **2a** (68.2 mg, 0.194 mmol, 1 equiv), magnesium ribbon (235.9 mg, 0.702 mmol, 50 equiv), and methanol (10 mL). The mixture was sonicated for 15 seconds and afterwards stirred at room temperature for 3h. The reaction mixture was then diluted with saturated ammonium chloride solution (6 mL), transferred to a 60 mL separatory funnel containing 20 mL of saturated ammonium chloride solution. The flask was rinsed with DCM (10 mL) and transferred to the separatory funnel. The layers were separated, and the aqueous phase was extracted with DCM (25 mL). The combined organic fractions were dried over anhydrous sodium sulfate, concentrated in vacuo, and the residue was purified by column chromatography (15:85 EtOAc:hexanes) to give **2b** (29.1 mg, 76%) as a pale yellow solid which was used in next step without further characterization.

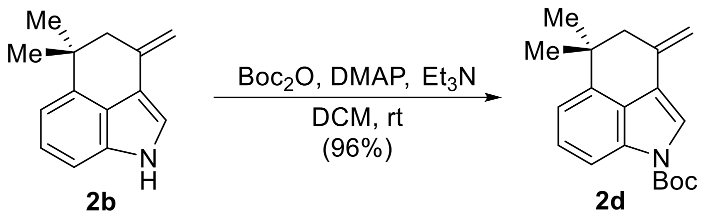

2b **2d**

***tert*-butyl 5,5-dimethyl-3-methylene-4,5-dihydrobenzo[*cd*]indole-1(3H)-carboxylate (2d)**.

To an oved-dried 2-dram vial and stirbar was added **2b** (54.3 mg, 0.275 mmol, 1 equiv), DMAP (3.4 mg, 0.0275 mmol, 0.1 equiv), anhydrous DCM (0.5 mL), triethylamine (0.115 mL, 0.826 mmol, 3 equiv), and di-tert-butyl dicarbonate (66.1 mg, 0.303 mmol, 1.1 equiv). The resulting yellow/brown solution mixture was further diluted with anhydrous DCM (0.6 mL) and left to stir at room temperature overnight. The following morning the reaction mixture was concentrated in vacuo and the residue was purified by column chromatography (5:95 EtOAc:hexanes) to give **2d** (78.5 mg, 96%) as a colorless gel.

^{1}H NMR (500 MHz, $CDCl_3$): δ 7.79 (s, 1H), 7.61 (s, 1H), 7.29 (d, *J* = 7.9 Hz, 1H), 7.15 (d, *J* = 7.2 Hz, 1H), 5.44 (s, 1H), 5.07 (d, *J* = 1.4 Hz, 1H), 2.50 (s, 2H), 1.67 (s, 9H), 1.31 (s, 6H).

^{13}C NMR (100 MHz, CDCl3): δ 150.3, 140.9, 136.6, 133.7, 127.7, 125.4, 119.0, 117.3, 117.0, 112.9, 109.5, 83.7, 48.8, 36.2, 28.9, 28.4.

HRMS (ESI): calcd for $C_{19}H_{24}NO_2^+$ $[M+H]^+$: 298.1802, found: 298.1800.

m.p: 115 –117 °C.

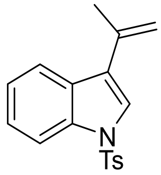

5a

3-(prop-1-en-2-yl)-1-tosyl-1H-indole (5a)

Prepared using **Procedure B:** 1-(1-tosyl-1H-indol-3-yl)ethan-1-one (321 mg, 1.03 mmol, 1 equiv) was subjected to general **Procedure B** with modifications: Nysted reagent solution (20 wt% in THF; 2.56 mL, 1.33 mmol, 1.3 equiv) and $TiCl_4$ solution (1.0 M in DCM; 1.33 mL, 1.33 mmol, 1.3 equiv). Product: *N*-tosyl-3-isopropenyl indole **5a** (244 mg, 77%), obtained as a white solid.

^{1}H NMR (500 MHz, CDCl$_3$): δ 8.00 (dt, *J* = 8.4, 0.9 Hz, 1H), 7.81 (dt, *J* = 8.0, 1.1 Hz, 1H), 7.80 – 7.74 (m, 2H), 7.56 (s, 1H), 7.32 (ddd, *J* = 8.4, 7.2, 1.3 Hz, 1H), 7.30 – 7.24 (m, 1H), 7.25 – 7.19 (m, 2H), 5.51 (s, 1H), 5.25 – 5.20 (m, 1H), 2.34 (s, 3H), 2.17 (dd, *J* = 1.5, 0.8 Hz, 3H).

^{13}C NMR (100 MHz, CDCl$_3$): δ 145.1, 136.3, 135.7, 135.3, 130.0, 129.0, 127.0, 124.8, 124.1, 123.7, 123.6, 121.5, 113.9, 113.8, 23.2, 21.7.

HRMS (ESI): calcd for $C_{18}H_{18}NO_2S^+$ $[M+H]^+$: 312.1053, found: 312.1060.

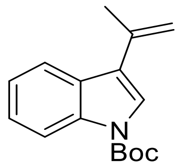

5b

tert-butyl 3-(prop-1-en-2-yl)-1H-indole-1-carboxylate (5b)

Prepared using **Procedure B:** tert-Butyl 3-acetyl-1H-indole-1-carboxylate (321 mg, 1.2 mmol, 1 equiv) was subjected to general **Procedure B** with modifications: Nysted reagent solution (20 wt% in THF; 5 mL, 2.6 mmol, 1.3 equiv) and $TiCl_4$ solution (1.0 M in DCM; 2.6 mL, 2.6 mmol, 1.3 equiv). Product: **5b** (298 mg, 58% yield), obtained as a yellow oil.

^{1}H NMR (500 MHz, CDCl$_3$): δ 8.20 – 8.15 (m, 1H), 7.85 (d, J = 7.8 Hz, 1H), 7.58 (s, 1H), 7.36 – 7.29 (m, 1H), 7.29 – 7.21 (m, 1H), 5.54 (s, 1H), 5.21 (s, 1H), 2.18 (s, 3H), 1.67 (s, 9H).

^{13}C NMR (126 MHz, CDCl$_3$): δ 149.7, 136.7, 136.0, 128.6, 124.4, 123.4, 122.9, 122.3, 120.9, 115.3, 113.0, 83.8, 30.3, 28.2, 23.1.

HRMS (ESI): calcd for $C_{16}H_{20}NO_2^+$ $[M+H]^+$: 258.1489, found: 258.1493.

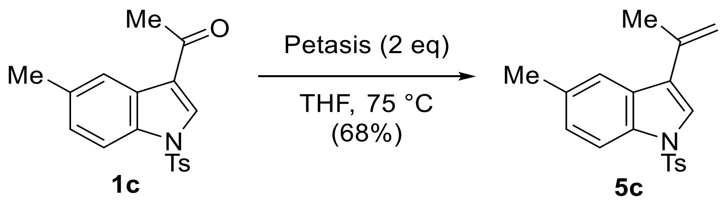

5-methyl-3-(prop-1-en-2-yl)-1-tosyl-1H-indole (5c)

General Procedure for the Synthesis of 3-Alkenyl Indoles with Petasis Reagent:

General procedure C

To a 50 mL round-bottomed flask fitted with a stirbar and maintained under nitrogen was added **1c** (164 mg, 0.5 mmol, 1 equiv) and 5 mL dry THF. After dissolution, Petasis reagent (0.5M solution in THF, 2 mL, 1.0 mmol 2 equiv) was added in dropwise, and the septum was replaced by a reflux condenser. The flask was then lowered into an oil bath preheated to 75 °C, and the reaction was left to stir overnight. The following day, the reaction mixture was diluted with ether (10 mL) and filtered through a short plug of silica gel. The resulting yellow solution was concentrated and purified by column chromatography (10:90 EtOAc:hexanes), providing **5c** (111 mg, 68% yield) as a white solid.

^{1}H NMR (400 MHz, $CDCl_3$): δ 7.87 (d, *J* = 8.5 Hz, 1H), 7.74 (d, *J* = 8.4 Hz, 2H), 7.58 (d, *J* = 0.8 Hz, 1H), 7.51 (s, 1H), 7.19 (d, *J* = 8.2 Hz, 2H), 7.13 (dd, *J* = 8.5, 1.7 Hz, 1H), 5.50 (s, 1H), 5.21 (t, *J* = 1.5 Hz, 1H), 2.41 (s, 3H), 2.31 (s, 3H), 2.15 (s, 3H).

^{13}C NMR (101 MHz, $CDCl_3$): δ 144.9, 136.3, 135.1, 133.8, 133.1, 129.8, 129.1, 126.8, 126.0, 123.8, 123.7, 121.3, 113.6, 113.4, 23.1, 21.5, 21.5.

HRMS (ESI): calcd for $C_{19}H_{20}NO_2S^+$ $[M+H]^+$: 326.1214, found: 326.1222

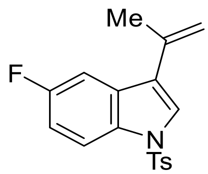

5d

5-fluoro-3-(prop-1-en-2-yl)-1-tosyl-1H-indole (5d)

Prepared using **Procedure C:** Compound **1d** (161 mg, 0.48 mmol, 1 equiv) was subjected to general **Procedure C** to afford **5d** (76.8 mg, 48%) as a white solid.

^{1}H NMR (400 MHz, $CDCl_3$): δ 7.94 (dd, *J* = 9.1, 4.6 Hz, 1H), 7.75 (d, *J* = 8.5 Hz, 2H), 7.58 (s, 1H), 7.45 (dd, *J* = 9.6, 2.5 Hz, 1H), 7.23 (d, *J* = 7.9 Hz, 2H), 7.05 (td, *J* = 8.9, 2.5 Hz, 1H), 5.42 (d, *J* = 1.2 Hz, 1H), 5.22 (p, *J* = 1.5 Hz, 1H), 2.35 (s, 3H), 2.15 (s, 3H).

^{13}C NMR (101 MHz, $CDCl_3$): δ 161.0, 158.6, 145.2, 135.8, 134.9, 131.8, 130.0, 129.9, 129.8, 128.2, 126.8, 125.1, 123.8, 123.8, 114.7, 114.6, 113.7, 112.8, 112.5, 107.3, 107.1, 23.0, 21.6.

HRMS (ESI): calcd for $C_{18}H_{17}FNO_2S^+$ $[M+H]^+$: 330.0959, found: 330.0950.

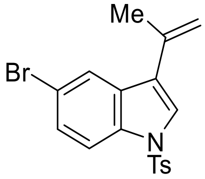

5e

5-bromo-3-(prop-1-en-2-yl)-1-tosyl-1*H*-indole (5e)

Prepared using **Procedure B:** Compound **1e** (390 mg, 1.0 mmol, 1 equiv) was subjected to general **Procedure B** with modifications: Nysted reagent solution (20 wt% in THF; 2.9 mL, 1.5 mmol, 1.5 equiv) and $TiCl_4$ solution (1.0 M in DCM; 1.5 mL, 1.5 mmol, 1.5 equiv). Product: **5e** (171 mg, 44% yield), obtained as a white solid.

^{1}H NMR (400 MHz, $CDCl_3$): δ 7.92 (d, *J* = 1.9 Hz, 1H), 7.87 (d, *J* = 8.8 Hz, 1H), 7.74 (d, *J* = 8.4 Hz, 2H), 7.54 (s, 1H), 7.41 (dd, *J* = 8.8, 1.9 Hz, 1H), 7.29 – 7.11 (m, 2H), 5.43 (d, *J* = 1.1 Hz, 1H), 5.23 (d, *J* = 1.5 Hz, 1H), 2.34 (s, 3H), 2.14 (s, 3H).

^{13}C NMR (101 MHz, $CDCl_3$): δ 145.3, 135.6, 134.8, 134.2, 130.6, 130.0, 127.6, 126.8, 124.6, 124.1, 123.4, 117.2, 115.1, 114.2, 23.0, 21.6.

HRMS (ESI): calcd for $C_{18}H_{17}BrNO_2S^+$ $[M+H]^+$: 390.0158, found 390.0145.

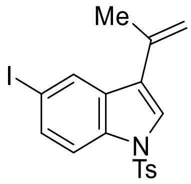

5f

5-iodo-3-(prop-1-en-2-yl)-1-tosyl-1*H*-indole (5f)

Prepared using **Procedure B:** Compound **1f** (439 mg, 1.0 mmol, 1 equiv) was subjected to general **Procedure B** with modifications: Nysted reagent solution (20 wt% in THF; 2.9 mL, 1.5 mmol, 1.5 equiv) and $TiCl_4$ solution (1.0 M in DCM; 1.5 mL, 1.5 mmol, 1.5 equiv). Product: **5f** (197 mg, 45% yield), obtained as a white solid.

^{1}H NMR (400 MHz, $CDCl_3$): δ 7.93 (dd, *J* = 8.4, 4.0 Hz, 1H), 7.75 (d, *J* = 8.4 Hz, 2H), 7.58 (s, 1H), 7.46 (dd, *J* = 8.4, 4.5 Hz, 1H), 7.23 (d, *J* = 8.5 Hz, 2H), 7.05 (dt, *J* = 8.4, 2.5 Hz, 1H), 5.43 (s, 1H), 5.29 – 5.20 (m, 1H), 2.35 (s, 3H), 2.14 (s, 3H).

^{13}C NMR (101 MHz, $CDCl_3$): δ 145.3, 135.6, 134.8, 134.7, 133.2, 131.1, 130.3, 130.0, 126.8, 124.2, 123.1, 115.5, 114.2, 87.9, 23.0, 21.6.

HRMS (ESI): calcd for $C_{18}H_{17}INO_2S^+$ $[M+H]^+$: 438.0019, found: 438.0010.

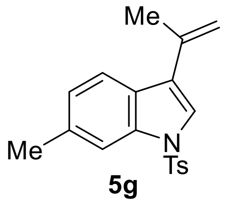

5g

6-methyl-3-(prop-1-en-2-yl)-1-tosyl-1*H*-indole (5g)

Prepared using **Procedure C:** Compound **1g** (163.5 mg, 0.5 mmol, 1 equiv) was subjected to general **Procedure C** to afford **5g** (115 mg, 71%) as a white solid.

^{1}H NMR (400 MHz, $CDCl_3$): δ 7.83 – 7.78 (m, 1H), 7.76 (d, *J* = 8.4 Hz, 2H), 7.67 (d, *J* = 8.2 Hz, 1H), 7.48 (s, 1H), 7.22 (d, *J* = 7.8 Hz, 2H), 7.08 (dd, *J* = 8.2, 1.5 Hz, 1H), 5.50 (s, 1H), 5.20 (t, *J* = 1.5 Hz, 1H), 2.47 (s, 3H), 2.34 (s, 3H), 2.15 (t, *J* = 1.1 Hz, 3H).

^{13}C NMR (101 MHz, $CDCl_3$): δ 144.9, 136.3, 136.0, 135.3, 134.8, 129.9, 126.8, 126.6, 125.0, 123.8, 123.0, 120.9, 113.8, 113.5, 23.0, 21.8, 21.5.**HRMS** (ESI): calcd for $C_{19}H_{20}NO_2S^+$ $[M+H]^+$: 326.1209, found: 326.1204.

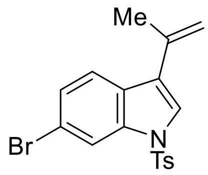

5h

6-bromo-3-(prop-1-en-2-yl)-1-tosyl-1H-indole (5h)

Prepared using **Procedure C:** Compound **1h** (195.5 mg, 0.5 mmol, 1 equiv) was subjected to general **Procedure C** to afford **5h** (91.4 mg, 47%) as a white solid.

^{1}H NMR (400 MHz, CDCl$_3$): δ 8.18 (d, *J* = 1.8 Hz, 1H), 7.77 (d, *J* = 8.4 Hz, 2H), 7.65 (d, *J* = 8.5 Hz, 1H), 7.51 (s, 1H), 7.37 (dd, *J* = 8.5, 1.8 Hz, 1H), 7.25 (d, *J* = 8.5 Hz, 0H), 5.44 (s, 1H), 5.22 (t, *J* = 1.4 Hz, 1H), 2.36 (s, 3H), 2.20 – 2.12 (m, 3H).

^{13}C NMR (101 MHz, CDCl$_3$): δ 145.3, 136.2, 135.8, 134.9, 130.1, 127.7, 126.8, 126.8, 123.9, 123.7, 122.5, 118.4, 116.7, 114.0, 23.0, 21.6.

HRMS (ESI): calcd for $C_{18}H_{17}BrNO_2S^+$ $[M+H]^+$: 390.0158, found: 390.0147.

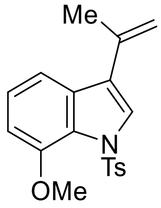

5i

7-methoxy-3-(prop-1-en-2-yl)-1-tosyl-1*H*-indole (5i)

Prepared using **Procedure B**: Compound **1i** (343mg, 1.0 mmol, 1 equiv) was subjected to general **Procedure B** with modifications: Nysted reagent solution (20 wt% in THF; 2.9 mL, 1.5 mmol, 1.5 equiv) and $TiCl_4$ solution (1.0 M in DCM; 1.5 mL, 1.5 mmol, 1.5 equiv). Product **5i** (133 mg, 39% yield) was obtained as a white solid.

^{1}H NMR (400 MHz, CDCl$_3$): δ 7.84 (s, 1H), 7.73 (d, *J* = 8.4 Hz, 2H), 7.45 (d, *J* = 8.1 Hz, 1H), 7.26 (d, *J* = 8.4 Hz, 2H), 7.16 (t, *J* = 8.0 Hz, 1H), 6.70 (d, *J* = 7.0 Hz, 1H), 5.51 (s, 2H), 5.24 (p, *J* = 1.6 Hz, 2H), 3.66 (s, 5H), 2.40 (s, 6H), 2.21 (s, 2H).

^{13}C NMR (101 MHz, $CDCl_3$): δ 147.4, 144.1, 137.5, 136.4, 131.7, 129.3, 127.2, 126.2, 125.4, 124.1, 121.8, 113.8, 113.4, 107.0, 55.4, 23.2, 21.6.

HRMS (ESI): calcd for $C_{19}H_{20}NO_3S^+$ $[M+H]^+$: 342.1158, found: 342.1152.

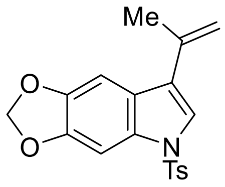

5j

7-(prop-1-en-2-yl)-5-tosyl-5H-[1,3]dioxolo[4,5-f]indole (5j)

Prepared using **Procedure B:** Compound **1j** (122 mg, 0.342 mmol, 1 equiv) was subjected to general **Procedure B** with modifications: Nysted reagent solution (20 wt% in THF; 1 mL, 0.513 mmol, 1.5 equiv) and $TiCl_4$ solution (1.0 M in DCM; 0.513 mL, 0.513 mmol, 1.5 equiv). Product **5j** (43 mg, 35% yield) was obtained as a white solid.

^{1}H NMR (500 MHz, $CDCl_3$): δ 7.74 (d, J = 8.4 Hz, 2H), 7.50 (s, 1H), 7.43 (s, 1H), 7.24 (d, J = 8.2 Hz, 2H), 7.17 (s, 1H), 5.98 (s, 2H), 5.37 (s, 1H), 5.18 (s, 1H), 2.36 (s, 2H), 2.12 (s, 3H).

^{13}C NMR (126 MHz, $CDCl_3$): δ 146.3, 145.3, 145.0, 136.3, 135.0, 130.4, 129.9, 126.8, 124.2, 123.0, 122.5, 113.2, 101.4, 100.2, 95.2, 23.1, 21.6.

HRMS (ESI): calcd for $C_{19}H_{18}NO_4S^+$ $[M+H]^+$: 356.0951, found: 356.0950.

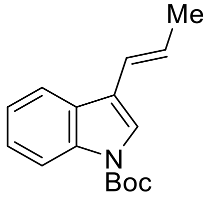

5k

tert-butyl (E)-3-(prop-1-en-1-yl)-1H-indole-1-carboxylate (5k)

Based on a procedure by Terada[6]

To a suspension of ethyltriphenylphosphonium bromide (744 mg, 2.0 mmol, 1 equiv) in 10 mL dry THF was added phenyllithium (1.6 M n-Bu_2O solution, 1.24 mL, 2.0 mmol, 1 equiv) at room temperature and the mixture was stirred for 10 min. The solution was cooled to -78 °C and *tert*-butyl 3-formylindole-1-carboxylate (490 mg, 2.0 mmol, 1 equiv) in 5 mL dry Et_2O was added dropwise. The reaction was stirred for 5 min at -78 °C and then for 30 min at -30 °C, whereupon an additional equivalent of phenyllithium was added and the reaction mixture was kept at -30 °C for 5 min. This reaction was treated with hydrogen chloride (2.0 M Et_2O solution, 1.1 mL, 2.2 mmol, 1.1 equiv), t-butanol (0.29 mL, 3.0 mmol, 1.5 equiv), and potassium t-butoxide (337 mg, 3.0 mmol, 1.5 equiv). The mixture was stirred for 2 h at room temperature. After cooling to 0 °C, the reaction mixture was quenched by saturated aqueous NH_4Cl solution and extracted with Et_2O (10 mL x 3). The combined organic layers were washed with brine, dried over anhydrous sodium sulfate, and evaporated under reduced pressure. Purification by flash chromatography (10:90 EtOAc:hexanes) gave **5k** as a colorless oil (262 mg, 51% yield, *E/Z* = 1:0.6)

^{1}H NMR (400 MHz, $CDCl_3$): Note: mixture of diastereomers: δ 8.12 (d, *J* = 7.7 Hz, 1H), 7.74 (d, *J* = 7.5 Hz, 1H), 7.59 (d, *J* = 6.0 Hz, 1H), 7.57 (s, 1H), 7.37 – 7.29 (m, 1H), 7.28 (d, *J* = 0.8 Hz, 1H), 6.56 – 6.48 (m, 1H), 5.92 (dq, *J* = 11.3, 7.0 Hz, 1H), 1.93 (td, *J* = 6.4, 5.7, 1.7 Hz, 3H), 1.68 (d, *J* = 8.3 Hz, 6H).

^{13}C NMR (101 MHz, $CDCl_3$): Note: mixture of diastereomers: δ 206.9, 130.5, 127.5, 126.3, 124.5, 123.4, 122.7, 122.5, 121.9, 119.8, 119.2, 119.1, 117.3, 115.2, 115.1, 83.7, 30.9, 28.2, 19.0, 15.6.

HRMS (ESI): calcd for $C_{16}H_{20}NO_2^+$ $[M+H]^+$: 258.1489, found: 258.1482.

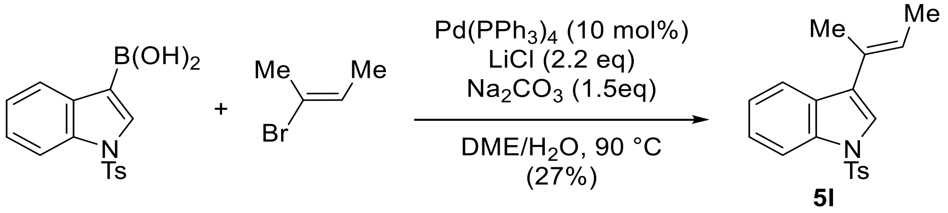

(*E*)-3-(but-2-en-2-yl)-1-tosyl-1H-indole (5l)

(1-Tosyl-1H-indol-3-yl)boronic acid (315.2 mg, 1.0 mmol, 1 equiv), $Pd(PPh_3)_4$ (115 mg, 0.1 mmol, 1 equiv), LiCl (93.3 mg, 2.2 mmol, 2.2 equiv), were combined in a 5 mL round-bottomed flask fitted with a condenser. The system was evacuated and backfilled with argon, twice. A solution of Na_2CO_3 (2M in H_2O, 1.5 mmol, 0.75 mL, 1.5 equiv), (*E*)-2-bromo-2-butene (0.1 mL, 1.0 mmol, 1 equiv), and 2 mL of degassed DME were added, and the flask was placed in an oil bath preheated to 90 °C and left to stir for 2 h. The reaction was cooled to room temperature, transferred with DCM (2 x 5mL) to a 25 mL separatory funnel containing 10 mL water. The aqueous layer was extracted with DCM (2 x 10 mL). The combined organic extracts were dried over sodium sulfate, filtered, and concentrated under reduced pressure. Purification by silica gel chromatography (6:94 to 12:88 EtOAc:hexanes) afforded **5l** as a colorless solidi (88 mg, 27%).

^{1}H NMR (400 MHz, $CDCl_3$): δ 7.99 (dt, *J* = 8.2, 1.0 Hz, 1H), 7.78 – 7.64 (m, 3H), 7.46 (s, 1H), 7.30 (ddd, *J* = 8.4, 7.2, 1.3 Hz, 1H), 7.25 – 7.17 (m, 3H), 6.03 (qq, *J* = 6.9, 1.4 Hz, 1H), 2.33 (s, 3H), 2.04 (p, *J* = 1.1 Hz, 3H), 1.83 (dq, *J* = 6.9, 1.1 Hz, 3H).

^{13}C NMR (101 MHz, $CDCl_3$): δ 144.8, 135.5, 135.2, 129.8, 129.2, 128.1, 126.8, 126.4, 124.5, 123.7, 123.2, 122.2, 121.2, 113.7, 21.5, 16.4, 14.0.

HRMS (ESI): calcd for $C_{19}H_{20}NO_2S^+$ $[M+H]^+$: 326.1209, found: 326.1206.

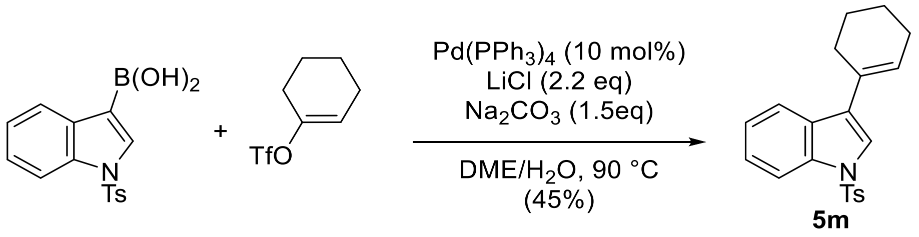

3-(cyclohex-1-en-1-yl)-1-tosyl-1H-indole (5m)

Prepared analogously to **(*E*)-3-(but-2-en-2-yl)-1-tosyl-1H-indole (5l)**: (1-tosyl-1H-indol-3-yl)boronic acid (200 mg, 0.635 mmol, 1 equiv) and triflate (146 mg, 0.635 mmol, 1 equiv), affording **5m** as a white solid (101 mg, 45%).

^{1}H NMR (400 MHz, CDCl$_3$): δ 7.99 (d, *J* = 8.1 Hz, 1H), 7.76 (t, *J* = 8.6 Hz, 2H), 7.46 (s, 1H), 7.34 – 7.17 (m, 6H), 6.27 (dt, *J* = 4.0, 2.2 Hz, 1H), 2.40 (ddt, *J* = 6.3, 4.1, 1.9 Hz, 2H), 2.33 (s, 3H), 2.29 – 2.19 (m, 2H), 1.80 (dtt, *J* = 11.5, 7.7, 4.4 Hz, 2H).

^{13}C NMR (101 MHz, CDCl$_3$): δ 144.8, 135.6, 135.2, 129.8, 129.7, 129.2, 126.8, 126.1, 125.0, 124.5, 123.2, 121.9, 121.3, 113.7, 28.3, 25.7, 22.8, 22.1, 21.5.

HRMS (ESI): calcd for $C_{21}H_{22}NO_2S^+$ $[M+H]^+$: 352.1366, found: 352.1358.

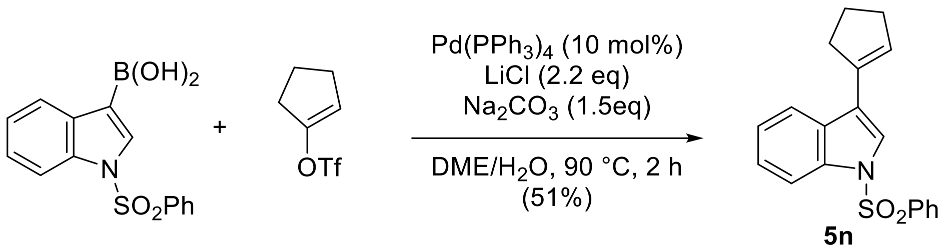

3-(cyclopent-1-en-1-yl)-1-(phenylsulfonyl)-1H-indole (5n):

Prepared analogously to **(*E*)-3-(but-2-en-2-yl)-1-tosyl-1H-indole (5l)**: (1-(phenylsulfonyl)-1H-indol-3-yl)boronic acid (250 mg, 0.83 mmol, 1 equiv) and triflate (179 mg, 0.83 mmol, 1 equiv), affording **5n** as a white solid (137 mg, 51%).

^{1}H NMR (400 MHz, CDCl$_3$): δ 8.01 (d, *J* = 8.0, 1.0 Hz, 1H), 7.90 – 7.85 (m, 2H), 7.80 (d, *J* = 7.8 Hz, 1H), 7.55 – 7.50 (m, 1H), 7.47 (bs, 1H), 7.45 – 7.40 (m, 2H), 7.36 – 7.27 (m, 2H), 6.30 (m, 1H), 2.77 – 2.71 (m, 2H), 2.63 – 2.55 (m, 2H), 2.05 – 1.96 (m, 2H).

^{13}C NMR (101 MHz, CDCl$_3$): δ 138.2, 135.7, 134.9, 133.9, 129.4, 129.3, 127.4, 126.9, 124.9, 123.7, 123.4, 121.5, 120.34, 113.8, 34.8, 33.9, 22.6.

HRMS (ESI): calcd for $C_{19}H_{18}NO_2S^+$ $[M+H]^+$: 324.1053; found: 324.1048.
m.p: 151 –153 ^{o}C.

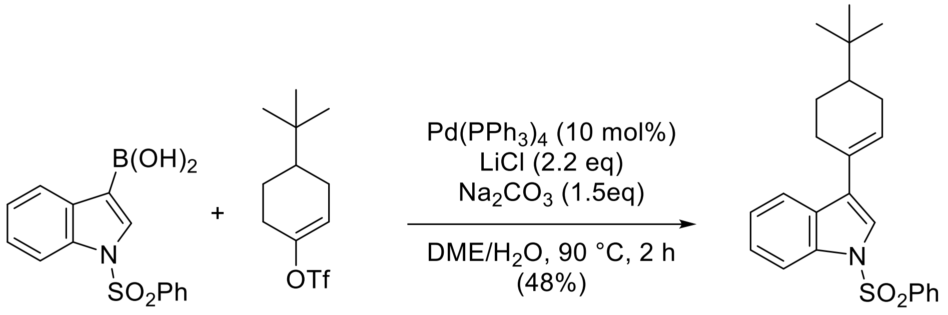

5o

3-(4-(tert-butyl)cyclohex-1-en-1-yl)-1-(phenylsulfonyl)-1H-indole (5o):

Prepared analogously to **(*E*)-3-(but-2-en-2-yl)-1-tosyl-1H-indole (5l)**: (1-(phenylsulfonyl)-1H-indol-3-yl)boronic acid (250 mg, 0.83 mmol, 1 equiv) and triflate (238 mg, 0.83 mmol, 1 equiv), affording **5o** as a syrup (156.6 mg, 48%).

^{1}H NMR (400 MHz, CDCl$_3$): δ 8.01 (d, *J* = 8.2 Hz, 1H), 7.90 – 7.83 (m, 2H), 7.77 (d, *J* = 8.0 Hz, 1H), 7.54 – 7.50 (m, 1H), 7.47 (s, 1H), 7.45 – 7.40 (m, 2H), 7.24 – 7.22 (m, 1H), 6.30 – 6.27 (m, 1H), 2.53 – 2.38 (m, 2H), 2.33 – 2.23 (m, 1H), 2.06 – 1.95 (m, 2H), 1.45 – 1.29 (m, 2H), 0.92 (s, 9H).

^{13}C NMR (101 MHz, CDCl$_3$): δ 138.3, 135.7, 133.8, 129.6, 129.38, 129.35, 126.8, 126.5, 125.0, 124.7, 123.49, 122.0, 121.5, 113.8, 43.9, 32.4, 30.0, 27.4, 27.3, 24.3.

HRMS (ESI): calcd for $C_{24}H_{28}NO_2S^+$ $[M+H]^+$: 394.1835, found: 394.1831.

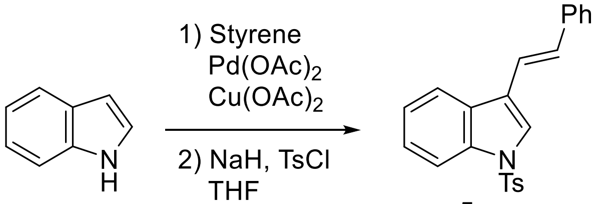

5p

(*E*)-3-styryl-1-tosyl-1H-indole (5p):

To a 50 mL oven-dried round-bottomed flask and stirbar was added 3-styrenyl indole [7] (219 mg, 1.0 mmol, 1 equiv) and dry THF 15 mL. NaH (60% dispersion in mineral oil, 60 mg, 1.5 mmol, 1.5 equiv) was added in one portion. The mixture was allowed to stir for 1 h after which Tosyl chloride (229 mg, 1.2 mmol, 1.2 equiv) was added in one portion and the reaction was left to stir

for 2 h. The reaction was quenched with water 50 mL and transferred to a separatory funnel containing 50 mL of 1:1 EtOAc:hexanes. The layers were separated, and the aqueous fraction was extracted 3 x 25 mL 1:1 EtOAc:hexanes. The combined organic layers were dried over sodium sulfate, filtered, and concentrated under reduced pressure. Purification by flash chromatography (15:85 EtOAc:hexanes) provided **5p** (361 mg, 97% yield) as a light yellow foam.

^{1}H NMR (500 MHz, CDCl$_3$): δ 8.02 (d, *J* = 8.1 Hz, 1H), 7.83 (d, *J* = 7.6 Hz, 1H), 7.77 (d, *J* = 8.2 Hz, 2H), 7.72 (s, 1H), 7.50 (d, *J* = 7.5 Hz, 2H), 7.35 (q, *J* = 7.0 Hz, 3H), 7.27 (dq, *J* = 20.6, 6.8, 6.0 Hz, 2H), 7.18 (d, *J* = 8.2 Hz, 2H), 7.16 (s, 2H), 2.29 (s, 3H).

^{13}C NMR (126 MHz, CDCl$_3$): δ 145.1, 137.4, 135.6, 135.1, 129.9, 129.8, 129.1, 128.7, 127.7, 126.8, 126.2, 125.1, 123.9, 123.6, 120.8, 120.4, 119.2, 113.8, 21.6.

HRMS (ESI): calcd for $C_{23}H_{20}NO_2S^+$ $[M+H]^+$: 374.1209, found: 374.1181.
m.p: 65 – 68 ^{o}C.

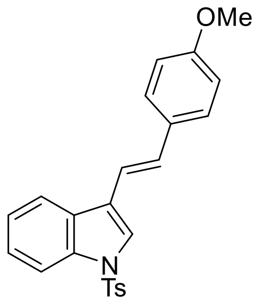

5q

(*E*)-3-(4-methoxystyryl)-1-tosyl-1H-indole (5q):
Prepared analogously to **(*E*)-3-styryl-1-tosyl-1H-indole (5p)**: (*E*)-3-(4-methoxystyryl)-1H-indole (124.7 mg, 0.5 mmol, 1 equiv), affording **5q** (111 mg, 55%).

^{1}H NMR (500 MHz, CDCl$_3$): δ 8.01 (d, *J* = 8.1 Hz, 1H), 7.82 (d, *J* = 7.6 Hz, 1H), 7.78 (d, *J* = 8.4 Hz, 2H), 7.68 (s, 1H), 7.44 (d, *J* = 8.7 Hz, 2H), 7.32 (dt, *J* = 25.9, 7.2 Hz, 2H), 7.20 (d, *J* = 8.1 Hz, 2H), 7.13 (d, *J* = 16.5 Hz, 1H), 7.02 (d, *J* = 16.5 Hz, 1H), 6.91 (d, *J* = 8.7 Hz, 2H), 3.83 (s, 3H), 2.32 (s, 3H).

^{13}C NMR (126 MHz, CDCl$_3$): δ 159.4, 144.9, 135.6, 130.2, 129.9, 129.4, 129.2, 127.4, 126.8, 124.9, 123.5, 123.3, 121.1, 120.4, 116.9, 114.2, 113.8, 55.3, 21.5.

HRMS (ESI): calcd for $C_{24}H_{22}NO_3S^+$ [M+H]$^+$: 404.1315, found: 404.1308.

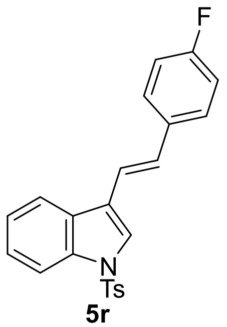

5r

(*E*)-3-(4-fluorostyryl)-1-tosyl-1H-indole (5r):

Prepared analogously to **(*E*)-3-styryl-1-tosyl-1H-indole (5p)**: 3-[(*E*)-2-(4-fluorophenyl)ethenyl]-1H-indole (237 mg, 1 mmol, 1 equiv), affording **5r** (253 mg, 65%).

^{1}H NMR (400 MHz, CDCl$_3$): δ 8.02 (d, J = 7.4 Hz, 1H), 7.82 (d, J = 7.9 Hz, 2H), 7.71 (s, 2H), 7.50 – 7.42 (m, 3H), 7.33 (dtd, J = 21.4, 7.3, 1.3 Hz, 3H), 7.21 (d, J = 8.1 Hz, 3H), 7.18 – 7.00 (m, 7H), 2.32 (s, 3H).

^{13}C NMR (101 MHz, CDCl$_3$): δ 163.6, 161.1, 145.1, 135.6, 135.1, 133.6, 133.5, 129.9, 129.0, 128.6, 127.7, 127.7, 126.8, 125.1, 123.8, 123.6, 120.6, 120.3, 118.9, 118.9, 115.8, 115.6, 113.8, 21.5.

HRMS (ESI): calcd for $C_{23}H_{19}FNO_2S^+$ [M+H]$^+$: 392.1115, found: 392.1106.

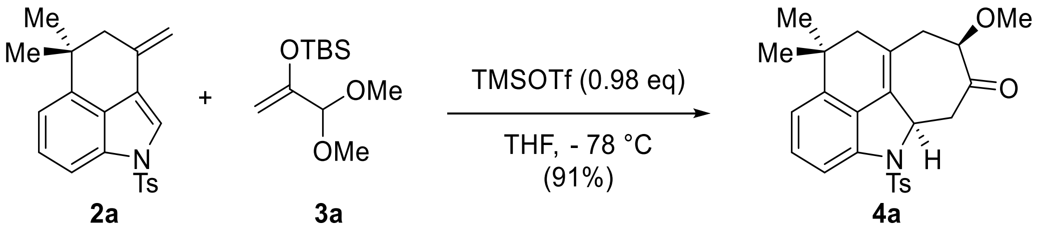

7-methoxy-10,10-dimethyl-4-tosyl-4a,5,7,8,9,10-hexahydrobenzo[*cd*]cyclohepta[*hi*]isoindol-6(4*H*)-one (4a):

Representative Procedure for the (4+3) Cycloaddition: **Procedure D**

A 10 mL round-bottomed flask with a magnetic stirbar was charged with TBS enol ether **3a** (52.3 mg, 0.225 mmol, 1 equiv) and tricyclic diene **2a** (79.1 mg, 0.225 mmol, 1 equiv). 4.5 mL THF was added to give a colorless solution, which was cooled to −78 °C. TMSOTf (0.040 mL, 0.221 mmol, 0.98 equiv) was added dropwise, and the mixture became light yellow immediately. After 1.5 h stirring at −78 °C, the reaction mixture was quenched with 3 mL saturated aqueous $NaHCO_3$ and allowed to warm to ambient temperature. The mixture was partitioned between 30 mL 1:1 brine: H_2O and 20 mL 1:1 EtOAc:hexanes, and the aqueous layer was extracted with 20 mL 1:1 EtOAc:hexanes. The combined organic layers were washed with brine, dried over Na_2SO_4, and concentrated under reduce pressure. The crude material was purified by flash chromatography (gradient elution 25:75 → 30:70 EtOAc:hexanes) to afford tetracycle **4a** (89.2 mg, 91% yield) as a white solid. The procedure for performing this reaction on a 2.2 mmol scale is presented on page S53.

Crystallization of **4a**: In a 2 mL HPLC vial, ca. 3 mg tetracycle **4a** was dissolved in a minimum amount of DCM, and then hexanes were added until the solution started to become cloudy. Another drop of DCM was added to form a colorless solution again. The vial was capped, and a 22G needle was attached. The vial was left at ambient temperature under Argon atmosphere to allow crystals to grow.

^{1}H NMR (500 MHz, $CDCl_3$): δ 7.66 (d, *J* = 8.4 Hz, 2H), 7.43 (d, *J* = 8.1 Hz, 1H), 7.23 (d, *J* = 8.0 Hz, 2H), 7.18 (t, *J* = 7.9 Hz, 1H), 6.92 (d, *J* = 7.5 Hz, 1H), 4.89 (d, *J* = 11.4 Hz, 1H), 4.27 (dd, *J* = 9.7, 4.4 Hz, 1H), 3.49 (dd, *J* = 15.3, 4.6 Hz, 1H), 3.36 (s, 3H), 2.99 (dd, *J* = 15.3, 11.5 Hz, 1H), 2.60 (d, *J* = 17.4 Hz, 1H), 2.45 – 2.28 (m, 2H), 2.37 (s, 3H), 2.05 (d, *J* = 17.4 Hz, 1H), 1.28 (s, 3H), 1.00 (s, 3H)

^{13}C NMR (125 MHz, $CDCl_3$): δ 208.4, 144.4, 140.6, 140.1, 134.4, 129.9, 129.9, 129.8, 127.5, 126.5, 123.7, 119.0, 113.4, 84.4, 61.4, 57.9, 48.4, 47.1, 38.4, 34.5, 29.9, 27.5, 21.7.

HRMS (ESI): calcd for $C_{25}H_{28}NO_4S^+$ $[M+H]^+$: 438.1734, found: 438.1728.
m.p. = 160–162 °C.

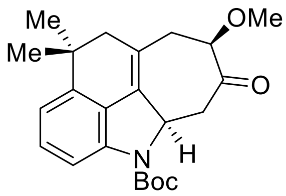

4b

***tert*-butyl (4a*R*,7*R*)-7-methoxy-10,10-dimethyl-6-oxo-5,6,7,8,9,10-hexahydrobenzo[*cd*]cyclohepta[*hi*]isoindole-4(4*aH*)-carboxylate (4b)** was prepared according to the representative procedure for the (4+3) cycloaddition (**Procedure D**) from diene **2d** (121 mg, 0.408 mmol, 1 equiv), TBS enol ether **3a** (142 mg, 0.612 mmol, 1.5 equiv), and TMSOTf (0.109 mL, 0.604 mmol, 1.48 equiv). Tetracycle **4b** (131 mg, 84% yield) was obtained as a light-yellow solid. The product exists as two rotamers in $CDCl_3$ at room temperature in a ratio of 1.6:1.

^{1}H NMR (500 MHz, $CDCl_3$): * denotes minor rotamer: δ 7.58 (br s, 1H*), 7.20 (br s, 1H), 7.15 (br s, 1H and 1H*), 6.90 (d, *J* = 7.5 Hz, 1H and 1H*), 5.40 (br s, 1H), 5.08 (br s, 1H*), 4.50 (br s, 1H), 4.32 (br s, 1H*), 3.66 (d, *J* = 16.7 Hz, 1H), 3.44 – 3.24 (m, 1H*), 3.36 (s, 3H and 3H*), 2.76 (br d, *J* = 19.7 Hz, 1H and 1H*), 2.65 (br s, 1H and 1H*), 2.41 (br s, 2H and 2H*), 2.09 (br d, *J* = 16.9 Hz, 1H and 1H*), 1.60 (s, 9H and 9H*), 1.34 (s, 3H and 3H*), 1.13 (s, 3H and 3H*).

^{13}C NMR (100 MHz, $CDCl_3$): Note: mixture of rotamers: δ 209.3, 208.6, 152.6, 140.3, 139.8, 130.8, 130.0, 129.6, 125.8, 122.6, 117.6, 113.1, 84.9, 83.3, 82.4, 81.8, 59.3, 58.4, 58.0, 47.4, 46.8, 46.3, 38.8, 38.1, 34.4, 30.0, 28.5, 27.4.

HRMS (ESI): calcd for $C_{23}H_{29}NO_4Na^+$ $[M+Na]^+$: 406.1989, found: 406.1984.

m.p: 145 –148 °C.

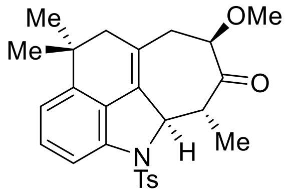

4c

(4a*R*,5*R*,7*R*)-7-methoxy-5,10,10-trimethyl-4-tosyl-4a,5,7,8,9,10-hexahydrobenzo[*cd*]cyclohepta[*hi*]isoindol-6(4*H*)-one (4c) was prepared according to the representative procedure for the

(4+3) cycloaddition (**Procedure D**) from diene **2a** (55.4 mg, 0.158 mmol, 1 equiv), TBS enol ether **3b** (58.3 mg, 0.237 mmol, 1.5 equiv), and TMSOTf (0.042 mL, 0.233 mmol, 1.48 equiv).Tetracycle **4c** (62.9 mg, 88% yield) was obtained as a white solid.

^{1}H NMR (500 MHz, CDCl$_3$): δ 7.44 (d, *J* = 7.9 Hz, 1H), 7.34 (d, *J* = 8.3 Hz, 2H), 7.19 (t, *J* = 7.8 Hz, 1H), 7.07 (d, *J* = 8.0 Hz, 2H), 6.94 (d, *J* = 7.5 Hz, 1H), 4.72 (d, *J* = 10.7 Hz, 1H), 4.39 (dd, *J* = 9.7, 5.1 Hz, 1H), 3.30 (s, 3H), 2.80 (dq, *J* = 10.6, 7.2 Hz, 1H), 2.71 – 2.64 (m, 1H), 2.29 (s, 3H), 2.28 – 2.19 (m, 2H), 1.97 (d, *J* = 17.4 Hz, 1H), 1.53 (d, *J* = 7.5 Hz, 3H), 1.23 (s, 3H), 0.72 (s, 3H).

^{13}C NMR (100 MHz, CDCl$_3$): δ 212.4, 144.1, 140.5, 139.9, 133.2, 130.7, 129.4, 129.3, 129.1, 127.7, 124.2, 120.2, 117.1, 82.7, 66.6, 57.8, 53.3, 47.1, 38.7, 34.2, 29.7, 26.9, 21.5, 15.0.

HRMS (ESI): calcd for $C_{26}H_{30}NO_4S^+$ $[M+H]^+$: 452.1890, found: 452.1886.

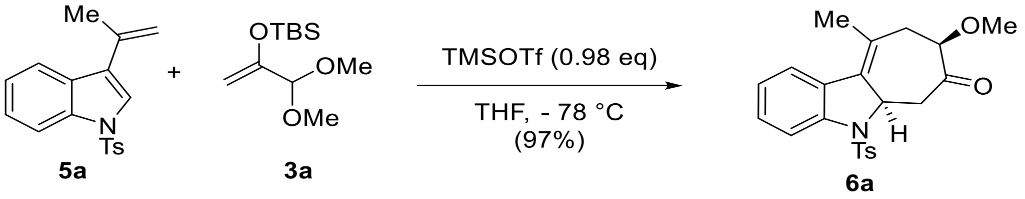

8-methoxy-10-methyl-5-tosyl-5a,6,8,9-tetrahydrocyclohepta[*b*]indol-7(5*H*)-one (6a) was prepared according to the representative procedure for the (4+3) cycloaddition (**Procedure D)** from diene **5a** (76.9 mg, 0.247 mmol, 1 equiv), TBS enol ether **3a** (86.1 mg, 0.371 mmol, 1.5 equiv), and TMSOTf (0.066 mL, 0.366 mmol, 1.48 equiv). Tricycle **6a** (94.9 mg, 97% yield) was obtained as a white solid.

^{1}H NMR (500 MHz, CDCl$_3$): δ 7.76 (ddd, *J* = 8.2, 1.2, 0.6 Hz, 1H), 7.63 – 7.57 (m, 2H), 7.49 (d, *J* = 7.9 Hz, 1H), 7.24 (ddd, *J* = 8.4, 7.4, 1.3 Hz, 1H), 7.19 (dt, *J* = 8.0, 0.7 Hz, 2H), 7.07 (td, *J* = 7.6, 1.1 Hz, 1H), 4.79 (ddq, *J* = 11.2, 3.4, 1.7 Hz, 1H), 4.16 (ddd, *J* = 7.3, 5.1, 0.9 Hz, 1H), 3.33 (s, 3H), 3.31 (ddd, *J* = 14.3, 3.5, 0.9 Hz, 1H), 2.92 (dd, *J* = 14.3, 11.3 Hz, 1H), 2.61 – 2.53 (m, 2H), 2.35 (s, 3H), 2.07 (d, *J* = 1.8 Hz, 3H).

^{13}C NMR (100 MHz, CDCl$_3$): δ 207.1, 144.5, 143.4, 134.3, 132.3, 129.9, 129.4, 128.7, 127.32, 127.28, 125.0, 124.5, 116.1, 84.9, 61.5, 57.9, 49.4, 41.1, 23.4, 21.7.

HRMS (ESI): calcd for $C_{22}H_{24}NO_4S^+$ $[M+H]^+$: 398.1421, found: 398.1417.

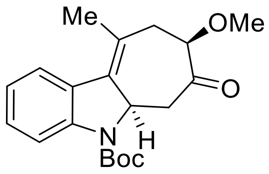

6b

tert-butyl-8-methoxy-10-methyl-7-oxo-6,7,8,9-tetrahydrocyclohepta[b]indole-5(5aH)-carboxylate (6b)

Prepared according to the representative procedure for the (4+3) cycloaddition (**Procedure D)** from diene **5b** (52 mg, 0.2 mmol, 1 equiv), TBS enol ether **3a** (57 mg, 0.3mmol, 1.5 equiv), and TMSOTf (0.05 mL, 0.3 mmol, 1.5 equiv). Tricycle **6b** (57.6 mg, 83% yield) was obtained as a yellow oil.

^{1}H NMR (500 MHz, $CDCl_3$): δ 7.99 (br. s, 1H), 7.58 (d, *J* = 7.8 Hz, 1H), 7.21 (t, *J* = 7.7 Hz, 1H), 7.03 (t, *J* = 7.5 Hz, 1H), 4.96 (br. d, *J* = 184.0 Hz, 1H), 4.04 (br. s, 1H), 3.31 (s, 3H), 2.69 (br. s, 2H), 2.20 (s, 2H), 1.59 (s, 9H), 1.26 (s, 3H), 0.90 (s, 2H).

^{13}C NMR (101 MHz, $CDCl_3$): δ 207.7, 182.0, 128.3, 125.9, 124.4, 122.7, 122.5, 115.5, 115.4, 90.7, 60.4, 57.6, 46.8, 34.7, 32.8, 31.6, 30.3, 28.4, 28.3, 28.2, 25.2, 22.6, 21.2, 21.0, 14.2, 14.1.

HRMS (ESI): calcd for $C_{20}H_{25}NO_4Na^+$ $[M+Na]^+$: 366.1681, found: 366.1668.

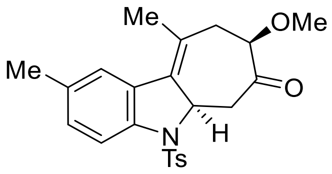

6c

8-methoxy-2,10-dimethyl-5-tosyl-5a,6,8,9-tetrahydrocyclohepta[*b*]indol-7(5*H*)-one (6c) was prepared according to the representative procedure for the (4+3) cycloaddition (**Procedure D)** from diene **5c** (50 mg, 0.154 mmol, 1 equiv), TBS enol ether **3a** (53.6 mg, 0.231mmol, 1.5 equiv),

and TMSOTf (0.03 mL, 0.154 mmol, 1.5 equiv). Tricycle **6c** (60.2 mg, 95% yield) was obtained as a white solid.

^{1}H NMR (500 MHz, CDCl$_3$): δ 7.65 (d, *J* = 8.4 Hz, 1H), 7.59 (d, *J* = 8.2 Hz, 2H), 7.28 (s, 1H), 7.19 (d, *J* = 8.1 Hz, 2H), 7.05 (d, *J* = 8.2 Hz, 1H), 4.75 (d, *J* = 10.1 Hz, 1H), 4.15 (dd, *J* = 6.9, 5.3 Hz, 1H), 3.33 (s, 3H), 3.28 (dd, *J* = 14.2, 3.4 Hz, 1H), 2.90 (dd, *J* = 14.3, 11.4 Hz, 1H), 2.58 – 2.53 (m, 2H), 2.35 (s, 3H), 2.32 (s, 3H), 2.07 (s, 3H).

^{13}C NMR (126 MHz, CDCl$_3$): δ 206.9, 144.1, 141.1, 134.2, 133.9, 132.4, 129.7, 129.3, 129.2, 127.2, 126.8, 125.3, 115.8, 84.9, 61.5, 57.7, 49.2, 40.9, 23.3, 21.5, 21.3.

HRMS (ESI): calcd for $C_{23}H_{26}NO_4S^+$ [M+H]$^+$: 412.1577, found: 412.1582.

m.p: 180 –182 ^{o}C.

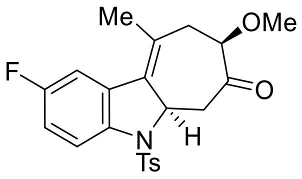

6d

2-fluoro-8-methoxy-10-methyl-5-tosyl-5a,6,8,9-tetrahydrocyclohepta[*b*]indol-7(5*H*)-one (6d) was prepared according to the representative procedure for the (4+3) cycloaddition (**Procedure D)** from diene **5d** (50 mg, 0.152 mmol, 1 equiv), TBS enol ether **3a** (86.1 mg, 0.371 mmol, 1.5 equiv), and TMSOTf (0.03 mL, 0.154 mmol, 1.01 equiv). Tricycle **6d** (58.2 mg, 92% yield) was obtained as a white solid.

^{1}H NMR (500 MHz, CDCl$_3$): δ 7.72 (dd, *J* = 8.9, 4.8 Hz, 1H), 7.57 (d, *J* = 8.2 Hz, 1H), 7.21 (d, *J* = 8.1 Hz, 2H), 7.17 (dd, *J* = 9.5, 2.5 Hz, 1H), 6.95 (td, *J* = 8.7, 2.6 Hz, 1H), 4.75 (d, *J* = 10.2 Hz, 1H), 4.16 – 4.08 (m, 2H), 3.32 (s, 3H), 3.27 (dd, *J* = 14.0, 3.1 Hz, 1H), 2.93 (dd, *J* = 14.1, 11.4 Hz, 1H), 2.63 – 2.48 (m, 3H), 2.37 (s, 2H), 2.05 (s, 3H).

^{13}C NMR (101 MHz, CDCl$_3$): δ 206.7, 161.2, 158.8, 144.5, 139.3, 139.3, 133.7, 131.7, 131.7, 130.9, 130.8, 129.8, 129.1, 127.2, 117.0, 116.9, 115.1, 114.9, 111.8, 111.6, 84.9, 61.9, 57.8, 49.1, 40.9, 23.2, 21.5.

HRMS (ESI): calcd for $C_{22}H_{23}FNO_4S^+$ $[M+H]^+$: 416.1326, found: 416.1325.

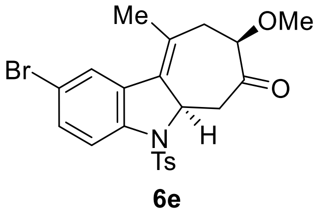

6e

2-bromo-8-methoxy-10-methyl-5-tosyl-5a,6,8,9-tetrahydrocyclohepta[*b*]indol-7(5*H*)-one (6e) was prepared according to the representative procedure for the (4+3) cycloaddition (**Procedure D)** from diene **5e** (50 mg, 0.247 mmol, 1 equiv), TBS enol ether **3a** (86.1 mg, 0.371 mmol, 1.5 equiv), and TMSOTf (0.066 mL, 0.366 mmol, 1.48 equiv). Ttricycle **6e** (55 mg, 90% yield) was obtained as a white solid.

^{1}H NMR (400 MHz, CDCl$_3$): δ 7.64 (d, *J* = 8.6 Hz, 1H), 7.61 – 7.55 (m, 3H), 7.34 (dd, *J* = 8.6, 2.0 Hz, 1H), 7.22 (d, *J* = 7.9 Hz, 2H), 4.72 (d, *J* = 11.0 Hz, 1H), 4.11 (dd, *J* = 8.1, 3.5 Hz, 1H), 3.32 (s, 3H), 3.27 (dd, *J* = 14.0, 2.6 Hz, 1H), 2.92 (dd, *J* = 13.9, 11.3 Hz, 1H), 2.66 – 2.48 (m, 2H), 2.37 (s, 3H), 2.07 (s, 3H).

^{13}C NMR (101 MHz, CDCl$_3$): δ 206.7, 144.6, 142.3, 133.8, 131.2, 131.2, 129.9, 129.3, 127.6, 127.1, 117.4, 117.2, 84.9, 61.8, 57.8, 49.0, 40.9, 23.5, 21.6.

HRMS (ESI): calcd for $C_{22}H_{22}BrNO_4S^+$ $[M+H]^+$: 476.0526, found: 476.0511.

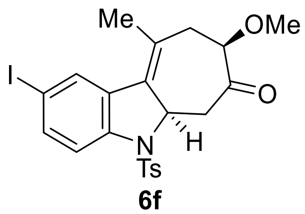

6f

2-iodo-8-methoxy-10-methyl-5-tosyl-5a,6,8,9-tetrahydrocyclohepta[*b*]indol-7(5*H*)-one (6f) was prepared according to the representative procedure for the (4+3) cycloaddition (**Procedure D)** from diene **5f** (50 mg, 0.1144 mmol, 1 equiv), TBS enol ether **3a** (40 mg, 0.1716 mmol, 1.5 equiv), and TMSOTf (0.02 mL, 0.1144 mmol, 1 equiv). Tricycle **6f** (55 mg, 92% yield) was obtained as a white/tan solid.

^{1}H NMR (400 MHz, $CDCl_3$): δ 7.77 (s, 1H), 7.59 (d, *J* = 8.3 Hz, 2H), 7.52 (d, *J* = 0.9 Hz, 2H), 7.22 (d, *J* = 8.0 Hz, 2H), 4.70 (d, *J* = 10.5 Hz, 1H), 4.10 (dd, *J* = 7.9, 3.7 Hz, 1H), 3.31 (s, 3H), 3.26 (dd, *J* = 13.6, 2.9 Hz, 1H), 2.91 (dd, *J* = 13.9, 11.2 Hz, 1H), 2.37 (s, 3H), 2.06 (s, 3H).

^{13}C NMR (101 MHz, CH_3CN+D_2O): δ 204.1, 142.1, 140.5, 134.6, 131.3, 131.0, 129.0, 128.5, 127.4, 126.7, 124.6, 115.2, 85.4, 82.4, 59.1, 55.2, 46.4, 38.4, 21.0, 19.0.

HRMS (ESI): calcd for $C_{22}H_{23}INO_4S^+$ $[M+H]^+$: 524.0387, found: 524.0392.

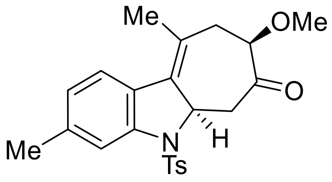

6g

8-methoxy-3,10-dimethyl-5-tosyl-5a,6,8,9-tetrahydrocyclohepta[*b*]indol-7(5*H*)-one (6g) was prepared according to the representative procedure for the (4+3) cycloaddition (**Procedure D)** from diene **5g** (50 mg, 0.154 mmol, 1 equiv), TBS enol ether **3a** (53.6 mg, 0.231mmol, 1.5 equiv), and TMSOTf (0.03 mL, 0.154 mmol, 1 equiv). Tricycle **6g** (58.8 mg, 93% yield) was obtained as a white solid.

^{1}H NMR (500 MHz, $CDCl_3$): δ 7.60 (d, *J* = 8.4 Hz, 3H), 7.36 (d, *J* = 7.9 Hz, 1H), 7.20 (d, *J* = 8.1 Hz, 2H), 6.88 (d, *J* = 7.9 Hz, 1H), 4.78 (d, *J* = 10.4 Hz, 1H), 4.16 (dd, *J* = 7.9, 4.5 Hz, 1H), 3.33 (s, 2H), 3.29 (dd, *J* = 14.3, 3.2 Hz, 1H), 2.90 (dd, *J* = 14.4, 11.4 Hz, 1H), 2.55 (qd, *J* = 16.6, 6.0 Hz, 3H), 2.38 (s, 3H), 2.36 (s, 3H), 2.04 (s, 3H).

^{13}C NMR (126 MHz, $CDCl_3$): δ 206.9, 144.2, 143.5, 139.0, 134.4, 132.1, 129.7, 127.1, 126.7, 125.6, 125.3, 124.5, 116.5, 84.8, 61.6, 57.7, 49.3, 40.9, 23.1, 21.7, 21.5.

HRMS (ESI): calcd for $C_{23}H_{26}NO_4S^+$ $[M+H]^+$: 412.1577, found: 412.1582.

m.p: 174 –176 °C.

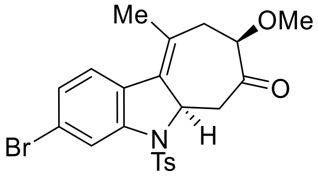

6h

3-bromo-8-methoxy-10-methyl-5-tosyl-5a,6,8,9-tetrahydrocyclohepta[*b*]indol-7(5*H*)-one (6h) was prepared according to the representative procedure for the (4+3) cycloaddition (**Procedure D)** from diene **5h** (50 mg, 0.129 mmol, 1 equiv), TBS enol ether **3a** (44.7 mg, 0.193 mmol, 1.5 equiv), and TMSOTf (0.03 mL, 0.154 mmol, 1.2 equiv). Tricycle **6h** (56.7 mg, 93% yield) was obtained as a white solid.

^{1}H NMR (500 MHz, $CDCl_3$): δ 7.93 (d, *J* = 1.7 Hz, 1H), 7.63 (d, *J* = 8.2 Hz, 2H), 7.32 (d, *J* = 8.4 Hz, 1H), 7.24 (d, *J* = 8.2 Hz, 2H), 7.19 (dd, *J* = 8.4, 1.7 Hz, 1H), 4.76 (d, *J* = 10.7 Hz, 1H), 4.13 (dd, *J* = 7.7, 4.2 Hz, 1H), 3.33 (s, 3H), 3.28 (dd, *J* = 14.0, 3.2 Hz, 1H), 2.91 (dd, *J* = 14.0, 11.3 Hz, 1H), 2.55 (qd, *J* = 16.3, 5.6 Hz, 2H), 2.38 (s, 3H), 2.04 (s, 3H).

^{13}C NMR (126 MHz, $CDCl_3$): δ 206.6, 144.7, 144.4, 134.0, 131.4, 129.9, 128.2, 128.1, 127.4, 127.1, 125.7, 122.2, 118.9, 84.9, 61.8, 57.8, 49.1, 40.9, 23.4, 21.5.

HRMS (ESI): calcd for $C_{22}H_{23}BrNO_4S^+$ $[M+H]^+$: 476.0526, found: 476.0511.

m.p: 192 –195 °C.

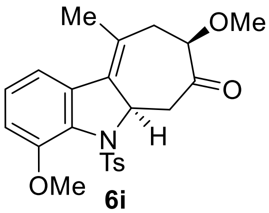

6i

4,8-dimethoxy-10-methyl-5-tosyl-5a,6,8,9-tetrahydrocyclohepta[b]indol-7(5*H*)-one (6i) was prepared according to the representative procedure for the (4+3) cycloaddition (**Procedure D)** from diene **5i** (50 mg, 0.247 mmol, 1 equiv), TBS enol ether **3a** (86.1 mg, 0.371 mmol, 1.5 equiv),

and TMSOTf (0.066 mL, 0.366 mmol, 1.48 equiv). Tricycle **6i** (58 mg, 93% yield) was obtained as a white solid.

^{1}H NMR (400 MHz, CDCl$_3$): δ 7.68 (d, *J* = 8.3 Hz, 2H), 7.26 (d, *J* = 8.0 Hz, 2H), 7.17 (d, *J* = 7.8 Hz, 1H), 7.06 (t, *J* = 8.0 Hz, 1H), 6.76 (d, *J* = 8.0 Hz, 1H), 5.60 (d, *J* = 11.2 Hz, 1H), 4.28 (dd, *J* = 8.1, 4.8 Hz, 1H), 3.55 (s, 3H), 3.34 (s, 3H), 3.13 (dd, *J* = 14.3, 3.5 Hz, 1H), 2.92 – 2.79 (m, 2H), 2.54 (dd, *J* = 16.8, 8.1 Hz, 1H), 2.42 (s, 3H), 2.09 (s, 3H).

^{13}C NMR (101 MHz, CDCl$_3$): δ 206.7, 149.9, 143.1, 138.0, 132.8, 132.7, 132.6, 129.0, 127.2, 126.8, 126.1, 117.2, 112.3, 84.2, 61.3, 57.8, 55.3, 49.1, 41.0, 22.8, 21.5.

HRMS (ESI): calcd for $C_{23}H_{26}NO_5S^+$ $[M+H]^+$: 428.1526, found: 428.1517.

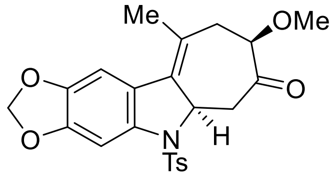

6j

8-methoxy-10-methyl-5-tosyl-5a,6,8,9-tetrahydrocyclohepta[*b*][1,3]dioxolo[4,5-*f*]indol-7(5*H*)-one (6j) was prepared according to the representative procedure for the (4+3) cycloaddition (**Procedure D)** from diene **5i** (50 mg, 0.247 mmol, 1 equiv), TBS enol ether **3a** (86.1 mg, 0.371 mmol, 1.5 equiv), and TMSOTf (0.066 mL, 0.366 mmol, 1.48 equiv). Tricycle **6j** (57.7 mg, 93% yield) was obtained as a white solid.

^{1}H NMR (400 MHz, CDCl$_3$): δ 7.58 (d, *J* = 8.3 Hz, 2H), 7.35 (s, 1H), 7.22 (d, *J* = 8.0 Hz, 2H), 6.95 (s, 1H), 6.01 (d, *J* = 1.2 Hz, 1H), 5.97 (d, *J* = 1.3 Hz, 1H), 4.75 – 4.68 (m, 2H), 4.12 (dd, *J* = 7.4, 4.7 Hz, 1H), 3.32 (s, 3H), 3.23 (dd, *J* = 14.3, 3.0 Hz, 1H), 2.89 (dd, *J* = 14.3, 11.3 Hz, 1H), 2.52 (d, *J* = 6.7 Hz, 2H), 2.37 (s, 3H), 1.98 (s, 3H)

^{13}C NMR (101 MHz, CDCl$_3$): δ 207.0, 148.0, 145.2, 144.3, 138.3, 133.7, 132.1, 129.8, 127.2, 123.7, 122.7, 104.4, 101.8, 98.7, 84.9, 62.0, 57.8, 49.2, 40.8, 22.9, 21.6.

HRMS (ESI): calcd for $C_{23}H_{24}NO_6S^+$ $[M+H]^+$: 442.1319, found: 442.1296.

m.p: 162 –164.2 ^{o}C.

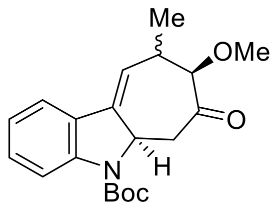

6k

tert-butyl-8-methoxy-9-methyl-7-oxo-6,7,8,9-tetrahydrocyclohepta[*b*]indole-5(5a*H*)-carboxylate (6k) was prepared according to the representative procedure for the (4+3) cycloaddition (**Procedure D)** with modifications from diene **5k** (200 mg, 0.777 mmol, 1 equiv), TBS enol ether **3a** (221.8 mg, 1.166 mmol, 1.5 equiv), and TMSOTf (0.14 mL, 0.777 mmol, 1 equiv) in nitroethane 15 mL. Purification by silica gel chromatography (gradient elution 5:95 → 15:85 acetone:hexanes) gave tricycle **6k** (213 mg, 3:1 dr, 80% yield) as a colorless oil.

^{1}H NMR (400 MHz, $CDCl_3$): δ 7.75 (d, *J* = 8.1 Hz, 1H), 7.35 – 7.29 (m, 2H), 7.21 (t, *J* = 7.8 Hz, 2H), 6.96 (t, *J* = 7.5 Hz, 1H), 6.06 (dd, *J* = 6.4, 3.1 Hz, 2H), 5.05 (s, 1H), 3.71 (d, *J* = 4.5 Hz, 1H), 3.42 (s, 4H), 3.01 – 2.86 (m, 3H), 2.22 (dd, *J* = 13.1, 11.0 Hz, 2H), 1.59 (s, 9H), 1.27 (d, *J* = 6.7 Hz, 3H).

^{13}C NMR (101 MHz, $CDCl_3$): δ 208.3, 206.4, 152.1, 129.5, 126.8, 124.6, 122.9, 122.7, 121.7, 119.7, 116.7, 115.8, 115.7, 90.2, 88.3, 81.8, 65.5, 61.8, 59.0, 58.0, 51.1, 45.1, 35.0, 29.8, 28.4, 28.3, 28.2, 21.0.

HRMS (ESI): calcd for $C_{20}H_{26}NO_4^+$ $[M+H]^+$: 344.1856, found: 344.1818.

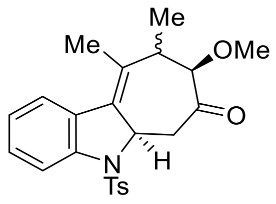

6l

8-methoxy-9,10-dimethyl-5-tosyl-5a,6,8,9-tetrahydrocyclohepta[*b*]indol-7(5*H*)-one (6l) was prepared according to the representative procedure for the (4+3) cycloaddition (**Procedure D)** with modifications from diene **5l** (50 mg, 0.154 mmol, 1 equiv), TBS enol ether **3a** (44 mg, 0.231 mmol, 1.5 equiv), and TMSOTf (0.03 mL, 0.165 mmol, 1.07 equiv) in nitroethane 3mL. Tricycle **6l** (49.3 mg, 2:1 dr, 78% yield) was obtained as a colorless oil

^{1}H NMR (400 MHz, CDCl$_3$): Note: mixture of diastereomers: δ 7.80 (d, *J* = 8.1 Hz, 2H), 7.74 – 7.61 (m, 2H), 7.56 (dd, *J* = 11.0, 8.5 Hz, 2H), 7.40 (d, *J* = 7.7 Hz, 1H), 7.19 (ddd, *J* = 14.6, 9.3, 5.6 Hz, 7H), 7.10 – 6.97 (m, 2H), 4.93 – 4.87 (m, 1H), 4.70 (s, 1H), 4.26 (s, 1H), 3.88 (d, *J* = 2.5 Hz, 1H), 3.47 (s, 2H), 3.25 (s, 1H), 3.08 (dt, *J* = 12.7, 6.7 Hz, 1H), 2.98 – 2.88 (m, 1H), 2.81 – 2.60 (m, 1H), 2.36 (s, 3H), 2.34 (s, 2H), 2.24 – 2.03 (m, 4H), 1.94 (d, *J* = 2.3 Hz, 3H), 1.23 (d, *J* = 7.0 Hz, 2H), 1.18 (d, *J* = 6.7 Hz, 3H).

^{13}C NMR (101 MHz, CDCl$_3$): Note: mixture of diastereomers: δ 208.1, 144.2, 135.8, 129.7, 129.6, 128.4, 128.0, 127.4, 127.1, 124.3, 124.3, 124.2, 124.1, 116.0, 115.6, 91.5, 90.0, 69.0, 59.0, 48.7, 35.7, 34.1, 29.6, 21.5, 17.3, 15.3, 14.3.

HRMS (ESI): calcd for $C_{23}H_{26}NO_4S^+$ $[M+H]^+$: 412.1577, found: 412.1540.

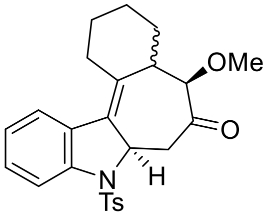

6m

5-methoxy-8-tosyl-1,3,4,4a,5,7,7a,8-octahydrobenzo[3,4]cyclohepta[1,2-*b*]indol-6(2*H*)-one (6m) was prepared according to the representative procedure for the (4+3) cycloaddition (**Procedure D)** with modifications from diene **5m** (50 mg, 0.142 mmol, 1 equiv), TBS enol ether **3a** (50 mg, 0.214 mmol, 1.5 equiv), and TMSOTf (0.025 mL, 0.142 mmol, 1 equiv) in nitroethane (3 mL). Tetracycle **6m** (49.1 mg, 1.3:1 dr, 79% yield) was obtained as a colorless oil.

^{1}H NMR (400 MHz, CDCl$_3$): Note: mixture of diastereomers: δ 7.77 (d, *J* = 8.2 Hz, 2H), 7.66 (d, *J* = 8.3 Hz, 1H), 7.59 (d, *J* = 8.2 Hz, 1H), 7.54 (d, *J* = 7.9 Hz, 1H), 7.02 (dt, *J* = 14.6, 7.6 Hz, 2H), 4.98 (t, *J* = 3.2 Hz, 1H), 4.77 (s, 1H), 4.26 (s, 1H), 3.74 (d, *J* = 3.5 Hz, 1H), 3.44 (s, 1H), 3.27 (s, 2H), 3.09 (s, 0H), 2.90 – 2.61 (m, 4H), 2.51 – 2.39 (m, 2H), 2.37 (s, 2H), 2.35 (s, 2H), 1.74 (dq, *J* = 12.6, 5.9, 5.2 Hz, 3H), 1.65 – 1.55 (m, 6H).

^{13}C NMR (101 MHz, CDCl$_3$): Note: mixture of diastereomers: δ 207.8, 207.6, 144.5, 144.1, 144.1, 137.4, 137.0, 134.6, 134.4, 130.5, 129.7, 129.6, 128.8, 128.4, 128.0, 127.3, 127.2, 127.1,

127.1, 124.7, 124.4, 124.3, 124.1, 116.5, 115.8, 91.1, 90.1, 68.5, 65.6, 59.4, 59.1, 47.8, 46.1, 38.2, 34.5, 31.2, 29.0, 26.5, 26.0, 23.6, 22.1, 21.5, 21.5, 20.5, 20.2.

HRMS (ESI): calcd for $C_{25}H_{28}NO_4S^+$ $[M+H]^+$: 438.1734, found: 438.1720.

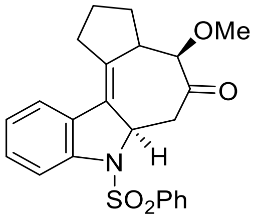

6n

4-Methoxy-7-(phenylsulfonyl)-1,2,3,3a,4,6,6a,7-octahydro-5H-azuleno[5,4-b]indol-5-one (6n): Was prepared according to the representative procedure for the (4+3) cycloaddition (**Procedure D**) from diene **5n** (30 mg, 0.09 mmol, 1 equiv), TBS enol ether **3a** (32.3 mg, 0.14 mmol, 1.5 equiv), and TMSOTf (16.5 µL, 0.09 mmol, 0.98 equiv). Tetracycle **6n** (19.7 mg, 52% yield) was obtained as a light-yellow solid as a single diastereomer.

^{1}H NMR (400 MHz, CDCl$_3$): δ 7.82 (d, *J* = 8.1 Hz, 1H), 7.73 – 7.70 (m, 2H), 7.53 – 7.49 (m, 1H), 7.41 – 7.33 (m, 3H), 7.25 – 7.20 (m, 1H), 7.09– 7.04 (m, 1H), 4.44 (t, *J* = 1.53 Hz, 1H), 4.37– 4.34 (m, 1H), 3.28 (s, 3H), 2.88– 2.81 (m, 1H), 2.78– 2.67 (m, 2H), 2.40 (dt, *J* = 11.0, 2.1 Hz, 1H), 2.35– 2.26 (m, 1H), 2.16 – 2.08 (m, 1H), 1.96 – 1.88 (m, 1H), 1.68 – 1.61 (m, 1H), 1.54 – 1.47 (m, 1H).

^{13}C NMR (101 MHz, CDCl$_3$): δ 209.8, 144.3, 140.8, 137.1, 133.5, 130.8, 129.2, 128.3, 127.4, 125.3, 124.5, 123.9, 115.7, 90.2, 67.61, 58.9, 46.6, 43.5, 35.0, 33.5, 26.0.

HRMS (ESI): calcd for $C_{23}H_{23}NO_4SNa^+$ $[M+Na]^+$: 432.1240, found: 432.1237.

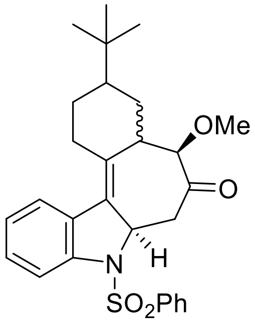

6o

3-(Tert-butyl)-5-methoxy-8-(phenylsulfonyl)-1,3,4,4a,5,7,7a,8 octahydrobenzo[3,4]cyclohepta[1,2-b]indol-6(2H)-one (6o): was prepared according to the representative procedure for the (4+3) cycloaddition (**Procedure D**) from diene **5o** (30 mg, 0.07 mmol, 1 equiv), TBS enol ether **3a** (26.5 mg, 0.11 mmol, 1.5 equiv), and TMSOTf (13.5 µL, 0.07 mmol, 0.98 equiv). Tetracycle **6o** (20.5 mg, 1:1.05 dr, 56% yield) was obtained as a white solid.

^{1}H NMR (500 MHz, CDCl$_3$): Note: mixture of diastereomers: δ 7.81 – 7.77 (m, 4H), 7.71 – 7.68 (m, 2H), 7.54 –7.49 (m, 3H), 7.45 – 7.35 (m, 5H), 7.23 – 7.17 (m, 2H), 7.07 – 6.97 (m, 2H), 4.99 – 4.96 (m, 1H), 4.71 – 4.68 (m, 1H), 4.23 – 4.21 (m, 1H), 3.76 – 3.74 (m, 1H), 3.43 (s, 3H), 3.28 – 3.23 (m, 1H), 3.20 (s, 3H), 2.99 – 2.86 (m, 3H), 2.85 – 2.76 (m, 2H), 2.71 – 2.62 (m, 1H), 2.51 – 2.43 (m, 1H), 2.3 – 2.19 (m, 3H), 1.88 – 1.79 (m, 2H), 1.74 – 1.64 (m, 2H), 1.52 – 1.42 (m, 2H), 0.86 (s, 9H), 0.86 (s, 9H).

^{13}C NMR (101 MHz, CDCl$_3$): Note: mixture of diastereomers: δ 208.0, 207.9, 144.7, 144.3, 137.7, 137.5, 137.2, 136.4, 133.4, 133.3, 130.7, 129.2, 129.20, 128.8, 128.6, 128.1, 127.4, 127.2, 126.7, 124.9, 124.7, 124.5, 124.3, 116.4, 115.8, 91.4, 90.3, 68.8, 65.8, 59.2, 59.1, 49.1, 46.8, 41.4, 40.4, 36.9, 35.0, 32.7, 32.7, 32.4, 30.3, 27.7, 27.4, 27.3, 27.1, 25.3, 24.1.

HRMS (ESI): calcd for $C_{28}H_{34}NO_4S^+$ $[M+H]^+$: 480.2203, found: 480.2201.

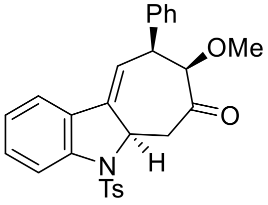

6p

8-methoxy-9-phenyl-5-tosyl-5a,6,8,9-tetrahydrocyclohepta[*b*]indol-7(5*H*)-one (6p) was prepared according to the representative procedure for the (4+3) cycloaddition (**Procedure D)** with modifications from diene **5p** (55 mg, 0.147 mmol, 1 equiv), TBS enol ether **3a** (52 mg, 0.221mmol, 1.5 equiv), and TMSOTf (0.03 mL, 0.165 mmol, 1.12 equiv) in nitroethane 3 mL. The product **6p** (49.1 mg, 83% yield) was obtained as a yellow/white foam.

^{1}H NMR (400 MHz, CDCl$_3$): δ 7.82 (d, *J* = 8.2 Hz, 1H), 7.66 (d, *J* = 8.3 Hz, 1H), 7.35 – 7.20 (m, 7H), 7.16 – 7.08 (m, 2H), 7.05 (q, *J* = 7.8, 7.4 Hz, 1H), 5.96 (t, *J* = 3.1 Hz, 1H), 5.56 (ddt, *J* = 11.2, 5.3, 2.9 Hz, 1H), 3.96 (q, *J* = 3.8 Hz, 1H), 3.87 (d, *J* = 4.2 Hz, 1H), 3.73 (dd, *J* = 17.6, 5.1 Hz, 1H), 3.53 (s, 2H), 2.93 (dd, *J* = 17.5, 11.3 Hz, 1H), 2.37 (s, 2H).

^{13}C NMR (101 MHz, CDCl$_3$): δ 206.1, 144.6, 143.0, 139.9, 138.6, 133.3, 129.9, 129.8, 129.1, 128.8, 128.4, 127.5, 127.4, 124.5, 120.3, 117.2, 116.3, 92.1, 59.0, 58.3, 50.8, 49.3, 21.6.

HRMS (ESI): calcd for $C_{27}H_{26}NO_4S^+$ $[M+H]^+$: 460.1577, found: 460.1545.

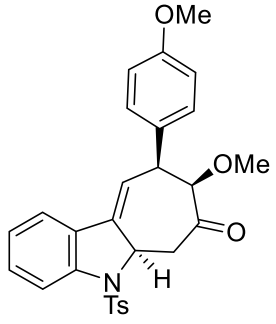

6q

8-methoxy-9-(4-methoxyphenyl)-5-tosyl-5a,6,8,9-tetrahydrocyclohepta[*b*]indol-7(5*H*)-one (6q) was prepared according to the representative procedure for the (4+3) cycloaddition (**Procedure D)** with modifications from diene **5q** (50 mg, 0.124 mmol, 1 equiv), TBS enol ether **3a** (44 mg, 0.186 mmol, 1.5 equiv), and TMSOTf (0.03 mL, 0.165 mmol, 1.3 equiv) in nitroethane 3 mL. Compound **6q** (56.4 mg, 93% yield) was obtained as a pale neon-yellow foam.

^{1}H NMR (400 MHz, CDCl$_3$): δ 7.82 (d, *J* = 8.2 Hz, 1H), 7.65 (d, *J* = 8.3 Hz, 2H), 7.33 – 7.23 (m, 4H), 7.22 (d, *J* = 8.3 Hz, 2H), 7.03 (d, *J* = 8.7 Hz, 2H), 6.83 (d, *J* = 8.7 Hz, 1H), 5.94 (t, *J* = 3.4 Hz, 1H), 5.57 (dq, *J* = 8.1, 2.3 Hz, 1H), 3.93 (q, *J* = 3.9 Hz, 1H), 3.84 (d, *J* = 4.4 Hz, 1H), 3.77 (s, 3H), 3.73 (dd, *J* = 17.7, 5.2 Hz, 1H), 3.53 (s, 3H), 2.92 (dd, *J* = 17.7, 11.3 Hz, 1H), 2.37 (s, 3H).

^{13}C NMR (101 MHz, CDCl$_3$): δ 206.3, 158.9, 144.6, 143.0, 138.2, 133.2, 131.8, 129.9, 129.8, 129.4, 129.2, 127.4, 124.5, 120.3, 117.5, 116.3, 114.2, 92.3, 58.9, 58.3, 55.2, 50.8, 48.5, 21.6.

HRMS (ESI): calcd for $C_{28}H_{28}NO_5S^+$ [M+H]$^+$: 490.1683, found: 490.1678.

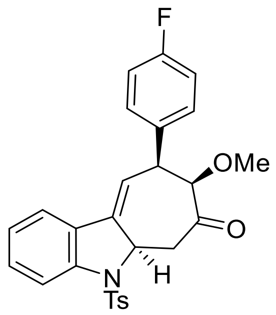

6r

9-(4-fluorophenyl)-8-methoxy-5-tosyl-5a,6,8,9-tetrahydrocyclohepta[*b*]indol-7(5*H*)-one (6r) was prepared according to the representative procedure for the (4+3) cycloaddition (**Procedure D)** with modifications from diene **5r** (50 mg, 0.128 mmol, 1 equiv), TBS enol ether **3a** (45 mg, 0.192 mmol, 1.5 equiv), and TMSOTf (0.03 mL, 0.165 mmol, 1.3 equiv) in nitroethane 3 mL. Compound **6r** (55.4 mg, 91% yield) was obtained as a pale colorless foam.

^{1}H NMR (500 MHz, CDCl$_3$): δ 7.82 (d, *J* = 8.2 Hz, 1H), 7.65 (d, *J* = 8.2 Hz, 2H), 7.31 (t, *J* = 7.8 Hz, 1H), 7.28 – 7.25 (m, 2H), 7.23 (d, *J* = 8.2 Hz, 2H), 7.12 – 7.07 (m, 3H), 7.05 (t, *J* = 7.6 Hz, 1H), 7.02 – 6.97 (m, 2H), 5.91 (t, *J* = 3.2 Hz, 1H), 5.55 (ddt, *J* = 11.1, 5.3, 3.0 Hz, 1H), 3.95 (q, *J* = 4.0 Hz, 1H), 3.83 (d, *J* = 4.3 Hz, 1H), 3.74 (dd, *J* = 17.6, 5.1 Hz, 1H), 3.53 (s, 3H), 2.91 (dd, *J* = 17.5, 11.3 Hz, 1H), 2.37 (s, 3H).

^{13}C NMR (126 MHz, CDCl$_3$): δ 206.0, 163.0, 161.0, 144.6, 143.1, 138.8, 135.8, 133.3, 130.1, 130.0, 129.9, 129.7, 128.9, 127.4, 124.5, 120.3, 116.9, 116.3, 115.7, 115.6, 92.1, 59.0, 58.3, 50.8, 48.5, 21.5.

HRMS (ESI): calcd for $C_{27}H_{25}FNO_4S^+$ [M+H]$^+$: 478.1483, found: 478.1457.

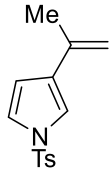

7a

3-(prop-1-en-2-yl)-1-tosyl-1*H*-pyrrole (7a)

Prepared using **general procedure B**

1-(1-tosyl-1H-pyrrol-3-yl)ethan-1-one (263 mg, 1 mmol, 1 equiv) was subjected to general **Procedure B** with modifications: Nysted reagent solution (20 wt% in THF; 2.9 mL, 1.5 mmol, 1.5 equiv) and $TiCl_4$ solution (1.0M in DCM; 1.5 mL, 1.5 mmol, 1.5 equiv). Compound **7a** (128 mg, 49% yield) was obtained as a tan solid.

^{1}H NMR (500 MHz, $CDCl_3$): δ 7.74 (d, *J* = 7.9 Hz, 2H), 7.31 – 7.24 (m, 3H), 7.10 (s, 2H), 6.45 (s, 1H), 5.21 (s, 1H), 4.90 (s, 1H), 2.40 (s, 3H), 1.98 (s, 3H).

^{13}C NMR (126 MHz, $CDCl_3$): δ 145.0, 136.0, 135.0, 130.6, 130.0, 126.8, 121.4, 116.9, 111.3, 110.8, 21.6, 21.0

HRMS (ESI): calcd for $C_{14}H_{16}NO_2S^+$ $[M+H]^+$: 262.0896, found: 262.0889.

(*E*)-3-styryl-1-tosyl-1H-pyrrole (7b):

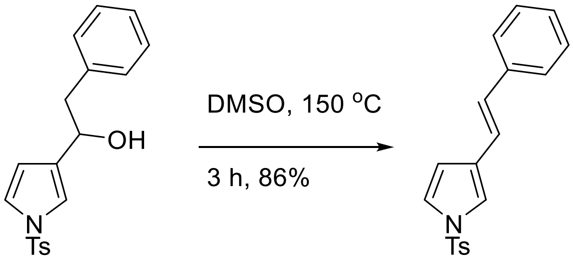

7b

Compound **7b** was prepared according to a literature procedure[8] with slight modification: A solution of alcohol (200 mg, 0.58 mmol, 1.0 equiv) in DMSO (2 mL) was heated to 150 °C and stirred for 3 h. After cooling to room temperature, reaction mixture was diluted with hexanes (5 mL) to precipitate out the crude product. The resulting crude solid product was collected by filtration and further purified by flash column chromatography (10:90 EtOAc:hexanes) to afford the title compound **7b** (163 mg, 86%) as a white fluffy solid. The spectral data were in accordance with that reported in the literature.[8]

^{1}H NMR (400 MHz, CDCl$_3$): δ 7.78-7.74 (m, 2H), 7.44 – 7.39 (m, 2H), 7.35 – 7.28 (m, 4H), 7.25 – 7.18 (m, 2H), 7.15 – 7.13 (m, 1H), 6.90 (d, *J* = 16.0 Hz, 1H), 6.79 (d, *J* = 16.0 Hz, 1H), 6.54 (dd, *J* = 1.6, 3.3 Hz, 1H) 2.40 (s, 3H).

^{13}C NMR (101 MHz, CDCl$_3$): δ 145.2, 137.3, 136.0, 130.1, 128.7, 128.5, 127.7, 127.5, 127.0, 126.2, 122.0, 120.3, 118.8, 111.2, 21.7.

HRMS (ESI): calcd for $C_{19}H_{18}NO_2S^+$ [M+H]$^+$: 324.1053, found: 324.1045.
m.p: 201 –204 ^oC.

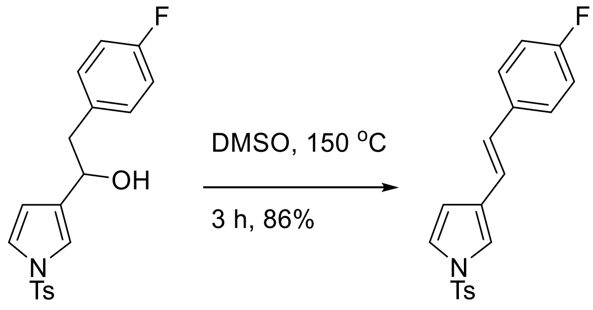

7c

(*E*)-3-(4-fluorostyryl)-1-tosyl-1H-pyrrole (7c):

Prepared analogously to **(*E*)-3-styryl-1-tosyl-1H-pyrrole (7b)** from 2-(4-fluorophenyl)-1-(1-tosyl-1H-pyrrol-3-yl)ethan-1-ol (200 mg, 0.56 mmol), to give **7c** as a white fluffy solid (159.6 mg, 84% yield).

^{1}H NMR (400 MHz, CDCl$_3$): δ 7.76 (d, *J* = 8.4 Hz, 2H), 7.39 –7.35 (m, 2H), 7.29 (d, *J* = 8.1 Hz, 2H), 7.20 –7.19 (m, 1H), 7.15 –7.14 (m, 1H), 7.03 – 6.99 (m, 2H), 6.80 (d, *J* = 16.3 Hz, 1H), 6.75 (d, *J* = 16.3 Hz, , 1H), 6.52 (dd, *J* = 1.6, 3.3 Hz, 1H), 2.39 (s, 3H).

^{13}C NMR (101 MHz, CDCl$_3$): δ 163.28 (d, *J* = 246.6 Hz), 145.2, 136.0, 133.5, 130.1, 127.7 (d, *J* = 8.1 Hz), 127.6, 127.3, 127.0, 122.0, 120.09 (d, *J* = 2.7 Hz), 118.7, 115.7 (d, *J* = 246.6 Hz), 111.1, 21.7.

HRMS (ESI): calcd for $C_{19}H_{117}FNO_2S^+$ [M+H]$^+$: 342.0964, found: 342.0955.

m.p: 160 –162.4 ^oC.

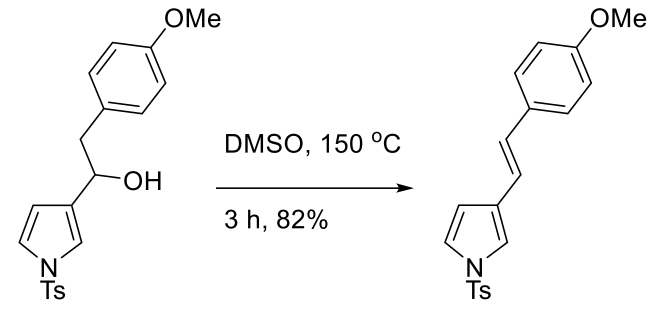

7d

(*E*)-3-(4-methoxystyryl)-1-tosyl-1H-pyrrole (7d):

Prepared analogously to **(*E*)-3-(4-fluorostyryl)-1-tosyl-1H-pyrrole (7b)** from 2-(4-methoxyphenyl)-1-(1-tosyl-1H-pyrrol-3-yl)ethan-1-ol (200 mg, 0.53 mmol), to give **7d** as a white fluffy solid (156 mg, 82%).

^{1}H NMR (400 MHz, $CDCl_3$): δ 7.75 (d, *J* = 8.4 Hz, 2H), 7.37–7.32 (m, 2H), 7.28 (d, *J* = 8.1 Hz, 2H), 7.17 – 7.14 (m, 1H), 7.12 (t, *J* = 2.8 Hz, 1H), 6.88 – 6.85 (m, 2H), 6.75 (s, 2H), 6.51 (dd, *J* = 3.3, 1.6 Hz, 1H), 3.80 (s, 3H), 2.39 (s, 3H).

^{13}C NMR (101 MHz, $CDCl_3$): δ 159.2, 145.1, 136.1, 130.17, 130.13, 128.1, 128.0, 127.4, 126.9, 121.9, 118.29, 118.23, 114.2, 111.2, 55.4, 21.7.

HRMS (ESI): calcd for $C_{20}H_{20}NO_3S^+$ $[M+H]^+$: 354.1163, found: 354.1156.

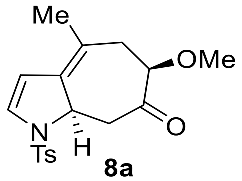

8a

6-methoxy-4-methyl-1-tosyl-5,6,8,8a-tetrahydrocyclohepta[*b*]pyrrol-7(1*H*)-one (8a)

Compound **8a** was prepared according to the representative procedure for the (4+3) cycloaddition (**Procedure D)** from diene **7** (50 mg, 0.19 mmol, 1 equiv), TBS enol ether **3a** (66.6 mg, 0.28 mmol, 1.5 equiv), and TMSOTf (52.0 µL, 0.28 mmol, 1.5 equiv). Tricycle **8a** (36.5 mg, 55% yield) was obtained as a tan solid.

^{1}H NMR (400 MHz, $CDCl_3$): δ 7.68 (d, *J* = 8.2 Hz, 2H), 7.33 (d, *J* = 8.0 Hz, 2H), 6.67 (d, *J* = 4.8 Hz, 1H), 5.76 (d, *J* = 4.1 Hz, 1H), 4.29 (d, *J* = 10.6 Hz, 1H), 4.08 (dd, *J* = 9.1, 4.0 Hz, 1H), 3.34

(s, 3H), 3.21 (dd, J = 14.6, 3.5 Hz, 1H), 2.89 (dd, J = 14.6, 11.5 Hz, 1H), 2.44 (s, 3H), 2.41 – 2.23 (m, 2H).

^{13}C NMR (101 MHz, $CDCl_3$): δ 207.8, 144.3, 137.3, 133.1, 132.9, 129.9, 127.5, 120.7, 112.1, 85.1, 58.9, 57.7, 47.4, 38.7, 22.1, 21.6.

HRMS (ESI): calcd for $C_{18}H_{22}NO_4S^+$ $[M+H]^+$: 348.1264, found: 348.1247.

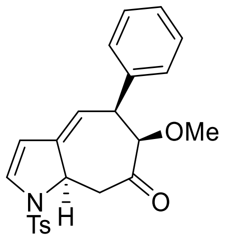

8b

6-methoxy-5-phenyl-1-tosyl-5,6,8,8a-tetrahydrocyclohepta[b]pyrrol-7(1H)-one (8b):

Compound **8b** was prepared according to the representative procedure for the (4+3) cycloaddition (**Procedure D)** from diene **7b** (50 mg, 0.15 mmol, 1 equiv), TBS enol ether **3a** (53.8 mg, 0.23 mmol, 1.5 equiv), and TMSOTf (27.4 µL, 0.15 mmol, 0.98 equiv). Tricycle **8b** (32.9 mg, 52% yield) was obtained as a fluffy solid.

^{1}H NMR (400 MHz, $CDCl_3$): δ 7.70 (d, J = 8.3 Hz, 2H), 7.38 – 7.33 (m, 2H), 7.31 – 7.20 (m, 3H), 7.10 – 7.06 (m, 2H), 6.73 (d, J = 6.7 Hz, 1H), 5.63 (d, J = 4.0 Hz, 1H), 5.34 – 5.32 (m, 1H), 5.01 – 4.93 (m, 1H), 3.82 – 3.74 (m, 2H), 3.54 (dd, J = 17.4, 5.1 Hz, 1H), 3.42 (s, 3H), 2.84 (dd, J = 17.4, 11.4 Hz, 1H), 2.45 (s, 3H).

^{13}C NMR (101 MHz, $CDCl_3$): δ 206.1, 144.7, 144.5, 140.0, 134.7, 132.6, 130.1, 128.8, 128.5, 127.8, 127.52, 116.8, 115.3, 92.5, 58.4, 56.9, 49.5, 49.4, 21.8.

HRMS (ESI): calcd for $C_{23}H_{24}NO_4S^+$ $[M+H]^+$: 410.1426, found: 410.1422.

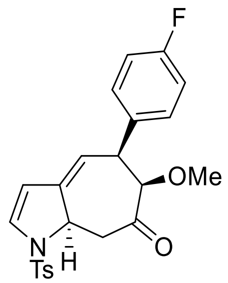

8c

5-(4-fluorophenyl)-6-methoxy-1-tosyl-5,6,8,8a-tetrahydrocyclohepta[b]pyrrol-7(1H)-one (8c):

Compound **8c** was prepared according to the representative procedure for the (4+3) cycloaddition (**Procedure D)** from diene **7c** (50 mg, 0.146 mmol, 1 equiv), TBS enol ether **3a** (51 mg, 0.219 mmol, 1.5 equiv), and TMSOTf (26.5 µL, 0.146 mmol, 0.98 equiv). Tricycle **8c** (36.3 mg, 58% yield) was obtained as a fluffy solid.

^{1}H NMR (400 MHz, C_6D_6): δ 7.66 (d, *J* = 8.3 Hz, 2H), 6.76 – 6.69 (m, 4H), 6.67 – 6.59 (m, 3H), 5.14 – 5.11 (m, 1H), 5.10 – 5.03 (m, 1H), 4.83 (t, *J* = 3.6 Hz, 1H), 3.76 (dd, *J* = 16.9, 5.1 Hz, 1H), 3.57 (d, *J* = 4.9 Hz, 1H), 3.39 (q, *J* = 4.2 Hz, 1H), 2.96 (s, 3H), 2.82 (dd, *J* = 16.9, 11.4 Hz, 1H), 1.77 (s, 3H).

^{13}C NMR (101 MHz, C_6D_6): δ 204.5, 162.3(d, *J* = 245.5 Hz), 144.8, 144.2, 136.74 (d, *J* = 3.1 Hz), 135.3, 133.4, 130.3 (d, *J* = 7.7 Hz), 129.9, 117.0, 115.7 (d, *J* = 21.6 Hz), 114.9, 92.4, 57.7, 57.4, 49.9, 48.8, 21.1.

HRMS (ESI): calcd for $C_{23}H_{23}FNO_4S^+$ $[M+H]^+$: 428.1332, found: 428.1331.

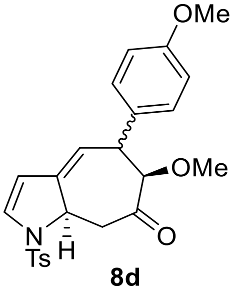

8d

6-methoxy-5-(4-methoxyphenyl)-1-tosyl-5,6,8,8a-tetrahydrocyclohepta[b]pyrrol-7(1H)-one (8d):

Compound **8d** was prepared according to the representative procedure for the (4+3) cycloaddition (**Procedure D)** from diene **7d** (50 mg, 0.141 mmol, 1 equiv), TBS enol ether **3a** (49.3 mg, 0.212 mmol, 1.5 equiv), and TMSOTf (25 µL, 0.139 mmol, 0.98 equiv). Tricycle **8d** (36 mg, 87:13 dr, 58% yield) was obtained as a fluffy solid.

^{1}H NMR (400 MHz, $CDCl_3$): Note: mixture of diastereomers: δ 7.74 (d, *J* = 8.4 Hz, 0.26 H), 7.69 7.72 (d, *J* = 8.4 Hz, 2 H), 7.35 (d, *J* = 8.8 Hz, 2 H), 7.30 (d, *J* = 8.1 Hz, 0.28 H), 7.01– 6.96 (m, 2H), 6.84 – 6.78 (m, 2H), 6.72 (d, *J* = 3.8 Hz, 1H), 6.60 (d, *J* = 3.7 Hz, 0.13 H), 5.62 (d, *J* = 4.3 Hz, 1H), 5.48 – 5.32 (m, 1H), 5.01 – 4.94 (m, 2H), 3.70 (br s, 0.37H), 3.76 – 3.72 (m, 5H), 3.52 (dd, *J* = 17.8, 5.0 Hz, 1H), 3.42 – 3.40 (m, 3.43 H), 2.94 –2.91 (m, 0.16H), 2.86 – 2.76 (dd, *J* = 17.3, 10.7 Hz, 1H), 2.44 (s, 3H), 2.40 (s, 3H).

^{13}C NMR (101 MHz, $CDCl_3$): Note: mixture of diastereomers: δ 206.2, 203.3, 159.4, 158.8, 145.5, 144.6, 144.1, 136.2, 134.5, 132.5, 131.9, 130.2, 130.0, 129.9, 129.7, 129.4, 127.7, 127.2, 117.1, 116.4, 115.3, 114.1, 92.6, 58.7, 58.32, 57.0, 56.8, 55.3, 55.3, 54.9, 54.8, 49.4, 48.5.

HRMS (ESI): calcd for $C_{24}H_{26}NO_5S^+$ $[M+H]^+$: 440.1526, found: 440.1536.

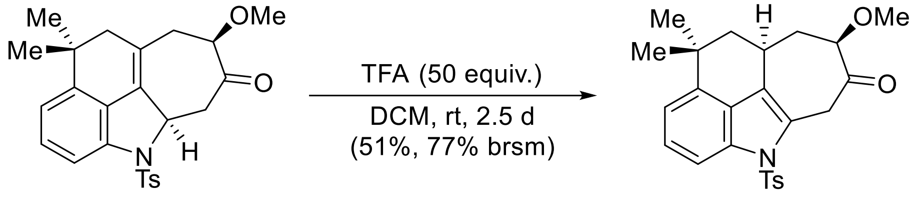

4a **9**

(4a*R*,7*R*)-7-methoxy-10,10-dimethyl-4-tosyl-4a,5,7,8,9,10-hexahydrobenzo[*cd*]cyclohepta-[*hi*]isoindol-6(4*H*)-one (9). A 10 mL round-bottomed flask with a magnetic stirbar was charged with tetracycle **4a** (28.0 mg, 0.0640 mmol, 1 equiv) and 1.6 mL DCM to give a colorless solution. TFA (0.147 mL, 1.92 mmol, 30 equiv) was added, and the resulting orange mixture was stirred at ambient temperature for 2 days. Then another 20 equiv TFA (0.098 mL, 1.28 mmol) was added, and the stirring was continued for an additional 12 h. The reaction mixture was carefully quenched with 4 mL saturated aqueous $NaHCO_3$, and partitioned between 20 mL 1:1 brine:H_2O and 20 mL DCM. The aqueous layer was extracted with 15 mL DCM, and the combined organic layers were dried over Na_2SO_4, and concentrated under reduced pressure. Purification by flash chromatography (20:80 EtOAc:hexanes) provided rearomatized tetracycle **9** (14.3 mg, 51% yield, 77% yield brsm[9]) as a white solid.

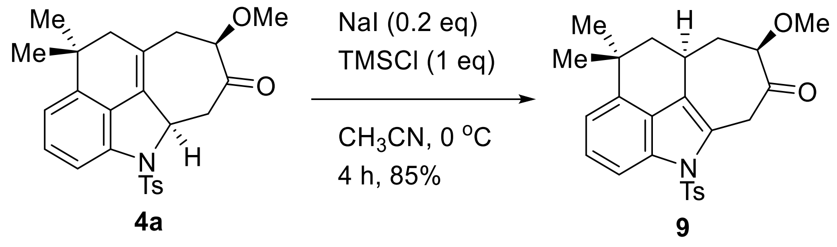

Alternative method for preparation of compound (9):

To a solution of tetracycle compound **4d** (20 mg, 0.045 mmol, 1 equiv) in 2 mL wet CH_3CN at 0 °C were added NaI (1.4 mg, 0.009 mmol, 0.2 equiv) and TMSCl (5.8 µL, 0.045 mmol, 1 equiv) sequentially. After stirring 4 h at the same temperature, the reaction mixture was quenched with saturated aqueous $Na_2S_2O_3$ (0.5 mL) and saturated sodium bicarbonate solution (0.5 mL). The mixture was transferred to a 50 mL separatory funnel and the aqueous layer was extracted with EtOAc (2 x 10 mL). The combined organic fractions were washed with brine, then dried over anhydrous sodium sulfate, filtered, and concentrated under reduced pressure. Purification by silica gel chromatography (20:80 EtOAc:hexanes) produced **9** (17 mg, 85% yield) as a white solid

^{1}H NMR (500 MHz, $CDCl_3$): δ 7.88 (d, *J* = 8.2 Hz, 1H), 7.72 (d, *J* = 8.4 Hz, 2H), 7.29 – 7.21 (m, 3H), 7.12 (d, *J* = 7.3 Hz, 1H), 4.46 (dd, *J* = 15.9, 2.2 Hz, 1H), 4.07 (d, *J* = 16.1 Hz, 1H), 4.01 (dd, *J* = 10.2, 5.4 Hz, 1H), 3.40 (s, 3H), 2.96 (tdd, *J* = 12.0, 5.0, 2.6 Hz, 1H), 2.36 (s, 3H), 2.26 (ddd, *J* = 13.4, 5.4, 3.0 Hz, 1H), 1.78 – 1.65 (m, 2H), 1.59 – 1.51 (m, 1H), 1.40 (s, 3H), 1.17 (s, 3H).

^{13}C NMR (125 MHz, $CDCl_3$): δ 205.1, 144.9, 140.2, 136.2, 135.2, 130.1, 126.69, 126.68, 125.5, 125.4, 122.2, 117.9, 112.5, 86.6, 57.9, 46.9, 40.1, 37.6, 34.7, 30.2, 28.1, 27.8, 21.7.

HRMS (ESI): calcd for $C_{25}H_{28}NO_4S^+$ $[M+H]^+$: 438.1734, found: 438.1736.

m.p: 158.5 –160.8 °C.

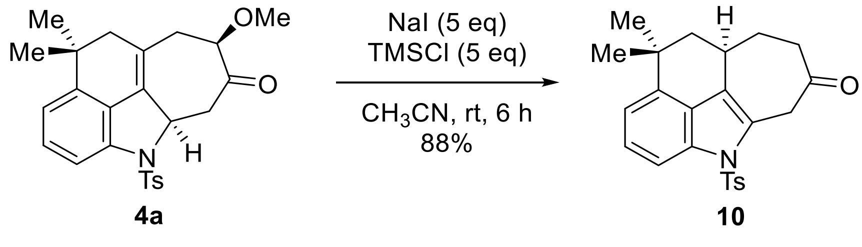

10,10-dimethyl-4-tosyl-5,7,8,8a,9,10-hexahydrobenzo[cd]cyclohepta[hi]isoindol-6(4H)-one (10):

To a solution of tetracyclic compound **4d** (20 mg, 0.045 mmol, 1 equiv) in 2 mL wet CH_3CN at 0 $^{\circ C}$were added NaI (33.7 mg, 0.225 mmol, 5 equiv) and TMSCl (28 µL, 0.225 mmol, 5 equiv) sequentially. After stirring 6 h at the same temperature reaction mixture was quenched with saturated aqueous $Na_2S_2O_3$ (0.5 mL) and saturated sodium bicarbonate solution (0.5 mL). The mixture was transferred to a 50 mL separatory funnel and the aqueous layer was extracted with EtOAc (2 x 10 mL). The combined organic fractions were washed with brine, then dried over anhydrous sodium sulfate, filtered, and concentrated under reduced pressure. Purification by silica gel chromatography (20:80 EtOAc:hexanes) produced **10** (16.3 mg, 88% yield) as a white solid.

^{1}H NMR (400 MHz, $CDCl_3$): δ 7.90 (dd, *J* = 8.3, 0.7 Hz, 1H), 7.73 – 7.69 (m, 2H), 7.28 – 7.20 (m, 3H), 7.12 (dd, *J* = 7.4, 0.7 Hz, 1H), 4.30 – 4.20 (m, 2H), 3.00 – 2.90 (m, 1H), 2.89 – 2.72 (m, 2H), 2.35 (s, 3H), 2.06 – 1.98 (m, 1H), 1.91 – 1.71 (m, 1H), 1.72 (dd, *J* = 12.9, 4.4 Hz, 1H), 1.62 – 1.51 (m, 1H), 1.40 (s, 3H), 1.18 (s, 3H).

^{13}C NMR (101 MHz, CDCl3): δ 206.3, 144.7, 140.1, 136.1, 135.1, 129.9, 126.8, 126.5, 125.1, 124.7, 122.4, 117.8, 112.3, 46.7, 44.4, 43.1, 34.6, 31.8, 30.0, 29.6, 28.1, 21.6.

HRMS (ESI): calcd for $C_{24}H_{25}NO_3SNa^+$ $[M+Na]^+$: 430.1453, found: 430.1454

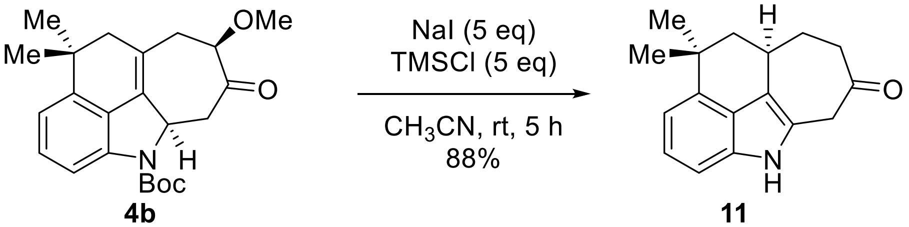

4b **11**

10,10-dimethyl-5,7,8,8a,9,10-hexahydrobenzo[*cd*]cyclohepta[*hi*]isoindol-6(4*H*)-one (11)

To a stirred solution of **4d** (20 mg, 0.052 mmol, 1 equiv) in 2 mL wet CH_3CN was added sodium iodide (39 mg, 0.26 mmol, 5 equiv). The system was then evacuated and backfilled with nitrogen twice. TMSCl (33 µL, 0.26 mmo, 5 equiv) was added in one portion and the reaction was allowed to proceed for 5 h. The reaction was quenched with the addition of saturated sodium thiosulfate solution 5 mL, and saturated sodium bicarbonate solution 5 mL. The mixture was transferred to a 50 mL separatory funnel, and the aqueous layer extracted 5 x 10 mL DCM. The combined organic

fractions were dried over anhydrous sodium sulfate, filtered, and concentrated under reduced pressure. Purification by silica gel chromatography (30:70 EtOAc:hexanes) produced **11** (11.6 mg, 88%) as a white solid.

^{1}H NMR (400 MHz, CDCl$_3$): δ 7.63 (s, 1H), 7.16 – 7.06 (m, 2H), 6.98 (dd, *J* = 6.7, 1.3 Hz, 1H), 4.06 (dd, *J* = 15.7, 2.2 Hz, 1H), 3.75 (d, *J* = 15.7 Hz, 1H), 3.20 (t, *J* = 11.7 Hz, 1H), 3.00 – 2.81 (m, 2H), 2.21 – 2.09 (m, 1H), 1.83 – 1.68 (m, 2H), 1.62 (d, *J* = 12.4 Hz, 1H), 1.45 (s, 3H), 1.27 (s, 3H).

^{13}C NMR (101 MHz, CDCl$_3$): δ 206.8, 139.8, 133.8, 126.5, 122.6, 122.5, 114.3, 113.3, 107.5, 47.4, 44.7, 44.7, 35.1, 32.6, 31.8, 30.2, 28.0.

HRMS (ESI): calcd for $C_{17}H_{20}NO^+$ $[M+H]^+$: 254.1539, found: 254.1530.

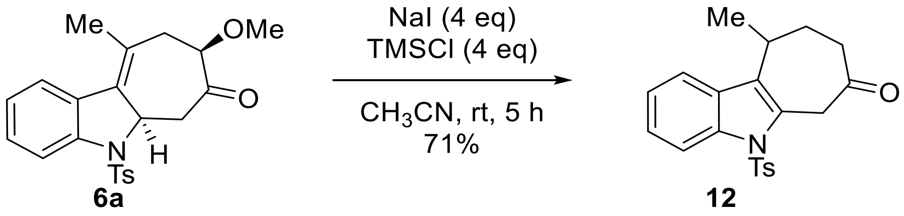

10-methyl-6,8,9,10-tetrahydrocyclohepta[*b*]indol-7(5*H*)-one (12)

Prepared in an analogous fashion to **10**. To a stirred solution of **6a** (20 mg, 0.0504 mmol, 1 equiv) and sodium iodide (30 mg, 0.2 mmol, 4 equiv) in wet acetonitrile (2 mL) was added TMSCl (0.03 mL, 0.2 mmol, 4 equiv). After stirring for 5 h the reaction mixture was worked up and purified as with **10**, to give **12** (13 mg, 71% yield) as a white solid.

^{1}H NMR (400 MHz, CDCl$_3$): δ 8.20 (d, *J* = 8.2 Hz, 1H), 7.65 (d, J = 8.4 Hz, 2H), 7.45 – 7.38 (m, 1H), 7.34 – 7.28 (m, 1H), 7.24 (dd, *J* = 7.3, 1.0 Hz, 1H), 7.19 (d, J = 8.0 Hz, 2H), 4.37 (d, *J* = 2.4 Hz, 2H), 3.36 – 3.24 (m, 1H), 2.72 (ddd, *J* = 17.1, 9.8, 3.7 Hz, 1H), 2.58 (ddd, J = 17.1, 7.7, 3.8 Hz, 1H), 2.34 (s, 3H), 2.32 – 2.18 (m, 1H), 2.05 (dtd, *J* = 14.9, 7.9, 3.8 Hz, 1H), 1.26 (d, *J* = 7.0 Hz, 3H).

^{13}C NMR (101 MHz, CDCl$_3$): δ 206.6, 144.7, 137.0, 135.5, 130.0, 129.8, 126.7, 126.5, 125.9, 124.6, 123.5, 118.6, 115.6, 40.4, 40.3, 30.1, 28.6, 21.5, 19.8.

HRMS (ESI): calcd for $C_{21}H_{22}NO_3S^+$ $[M+H]^+$: 368.1315 found: 368.1297

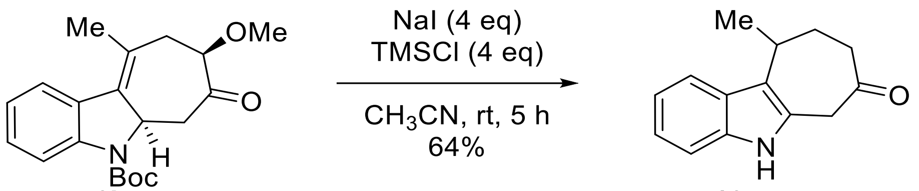

6b **13**

10-methyl-6,8,9,10-tetrahydrocyclohepta[b]indol-7(5H)-one (13):

Prepared (7.9 mg, 64% yield) in an analogous fashion to **11.**

^{1}H NMR (400 MHz, $CDCl_3$): δ 7.58 (s, 1H), 7.49 (d, *J* = 7.8 Hz, 1H), 7.23 (d, *J* = 7.8 Hz, 1H), 7.13 – 7.00 (m, 2H), 4.04 (d, *J* = 15.3 Hz, 1H), 3.62 (d, *J* = 15.3 Hz, 1H), 3.36 – 3.26 (m, 1H), 2.72 (ddd, *J* = 18.1, 9.2, 2.5 Hz, 1H), 2.55 (ddd, *J* = 18.0, 9.5, 2.6 Hz, 1H), 2.23 (dddd, J = 13.8, 9.1, 4.5, 2.5 Hz, 1H), 2.10 – 1.92 (m, 1H), 1.35 (d, *J* = 6.9 Hz, 3H).

^{13}C NMR (101 MHz, $CDCl_3$): δ 206.9, 135.6, 128.5, 124.1, 121.8, 119.5, 118.7, 116.4, 110.6, 42.7, 40.2, 30.7, 29.6, 21.3.

HRMS (ESI): calcd for $C_{14}H_{16}NO^+$ $[M+H]^+$: 214.1226 found: 214.1228.

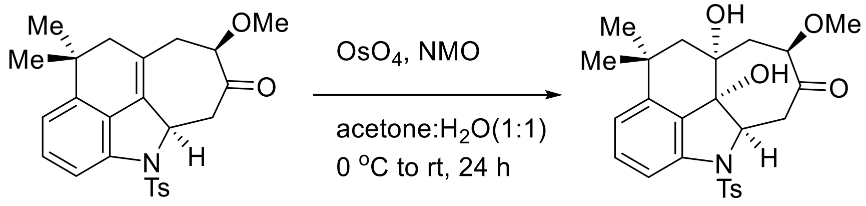

4a **14**

4a1,8a-dihydroxy-7-methoxy-10,10-dimethyl-4-tosyl-4a,4a1,5,7,8,8a,9,10-octahydrobenzo[cd]cyclohepta[hi]isoindol-6(4H)-one (14):

To a solution of compound **4a** (20 mg, 0.04 mmol, 1 equiv) in acetone: water (2 mL, 1:1) at 0 °C were added N-methylmorpholine N-oxide (6.3 mg, 0.05 mmol, 1.2 equiv) and osmium (IV) tetroxide (11.3 µL, 0.004 mmol, 0.1 equiv) sequentially under argon. After stirring for 24 h at room temperature, the reaction was quenched with saturated aqueous $Na_2S_2O_3$ (0.5 mL) at 0 °C, and the resulting mixture was extracted with EtOAc (3 x 10 mL). The combined extracts were washed with brine (5 mL), then dried over Na_2SO_4, filtered, and concentrated under reduced pressure. The resulting residue was purified by silica gel column chromatography (1:1 EtOAc/hexanes) to give the title compound **14** (16.3 mg, 76% yield) as a colorless oil.

^{1}H NMR (400 MHz, CDCl$_3$): δ 7.71 (d, *J* = 8.3 Hz, 2H), 7.51 (d, *J* = 8.4 Hz, 1H), 7.39 (t, *J* = 7.8 Hz, 1H), 7.25 – 7.22 (m, 2H), 7.03 (d, *J* = 7.8 Hz, 1H), 4.34 (dd, *J* = 11.9, 5.4 Hz, 1H), 3.69 (dd, *J* = 10.0, 6.3 Hz, 1H), 3.25 (s, 3H), 3.07 (t, *J* = 11.8 Hz, 1H), 2.83 (dd, *J* = 11.3, 5.7 Hz, 1H), 2.65 (d, *J* = 2.60 Hz, 1H), 2.38 (s, 3H), 2.37– 2.30 (m, 1H), 2.15 (d, *J* = 13.3 Hz, 1H), 1.93 – 1.86 (m, 1H), 1.64 (d, *J* = 13.6 Hz, 1H), 1.33 (s, 3H), 1.26 (s, 3H).

^{13}C NMR (101 MHz, CDCl$_3$): δ 203.9, 144.6, 143.9, 140.2, 135.0, 132.3, 129.9, 128.7, 127.3, 122.2, 115.0, 82.5, 80.7, 73.9, 71.7, 57.8, 52.5, 40.9, 38.2, 36.0, 33.6, 31.6, 21.7.

HRMS (ESI): calcd for $C_{25}H_{28}NO_5S^+$ [M+H−H$_2$O]$^+$: 454.1683, found: 454.1676**Enantioselective (4+3) cycloaddition of 5p with 3a:**

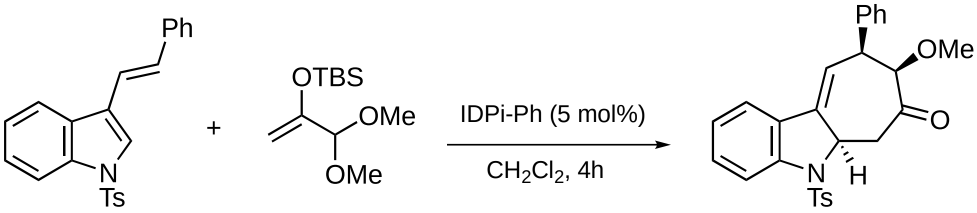

Table 1: Cycloaddition Results

Entry	Temperature	Yield %	*ee %*
1	22 °C	51 (86)[a]	45
2	10 °C	48 (86)[a]	51
3	0 °C	45 (87)[a]	55

a: based on recovered starting material

To an oven dried 2-dram vial and stir bar was added 3-alkenyl indole **5p** (10 mg, 0.026 mmol, 1 equiv), **3a** (12.5 mg, 0.053 mmol, 2 equiv), and solvent (0.6 mL). The vial was purged with argon and IDPI-Ph (1.7 mg, 0.001 mmol, 2 equiv) was added. The reaction solution was left to stir for 4 h at the respective temperatures, whereupon it was treated with saturated sodium bicarbonate solution (1 mL) and extracted with DCM (5 x 3 mL). The combined organic layers were then dried over sodium sulfate, filtered, and concentrated in vacuo to give the crude product, which was purified on preparatory TLC (35:65 EtOAc:hexanes).

The enantiomer ratio was determined using chiral HPLC using a ChiralPack-ID® column, eluting with 50% DCM:hexanes, 1 mL/min.

HPLC for racemic compound 6p:

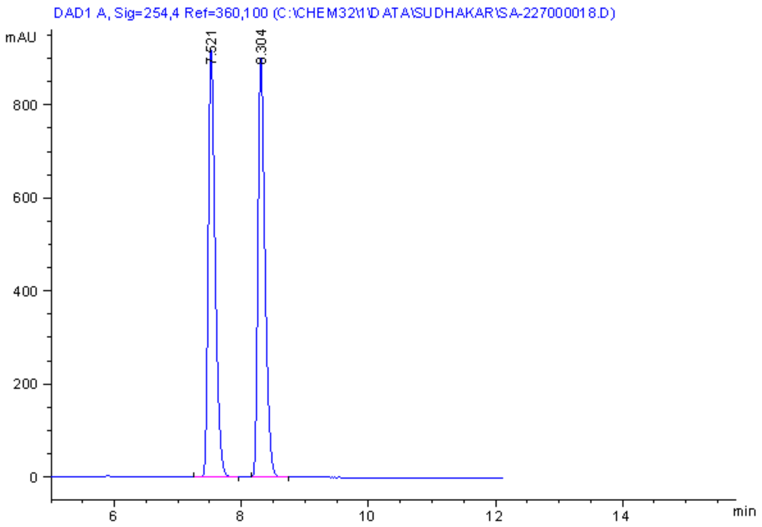

Signal 1: DAD1 A, Sig=254,4 Ref=360,100

Peak #	RetTime [min]	Type	Width [min]	Area [mAU*s]	Height [mAU]	Area %
1	7.521	VB	0.1122	6950.06201	919.01074	50.0863
2	8.304	BB	0.1156	6926.11816	902.19977	49.9137

H

PLC of enantioenrich compound 6p for entry 1 in table 1:

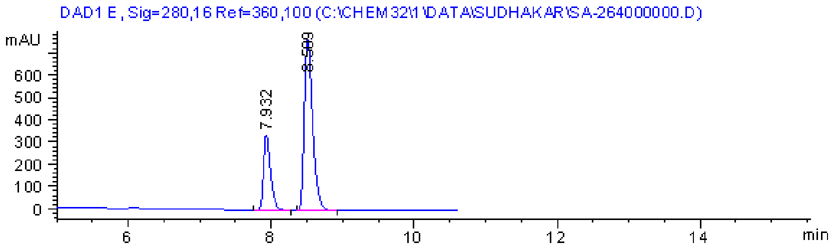

Signal 5: DAD1 E, Sig=280,16 Ref=360,100

Peak #	RetTime [min]	Type	Width [min]	Area [mAU*s]	Height [mAU]	Area %
1	7.932	BB	0.1013	2301.50757	338.70154	27.2866
2	8.509	BB	0.1193	6133.07422	767.19092	72.7134

Totals : 8434.58179 1105.89246

HPLC of enantioenrich compound 6p for entry 2 in table 1:

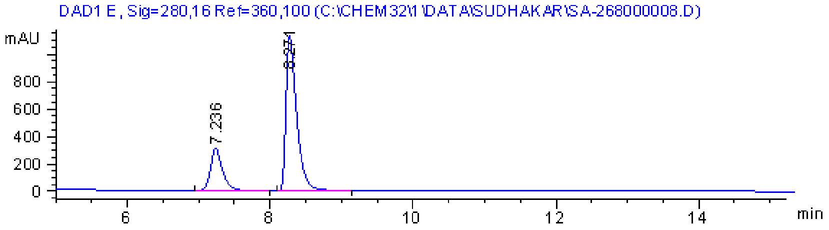

Signal 5: DAD1 E, Sig=280,16 Ref=360,100

Peak #	RetTime [min]	Type	Width [min]	Area [mAU*s]	Height [mAU]	Area %
1	7.236	BB	0.1789	3848.49438	314.12943	24.4257
2	8.271	BB	0.1591	1.19074e4	1128.65906	75.5743

Totals : 1.57559e4 1442.78848

HPLC of enantioenrich compound 6p for entry 3 in table 1:

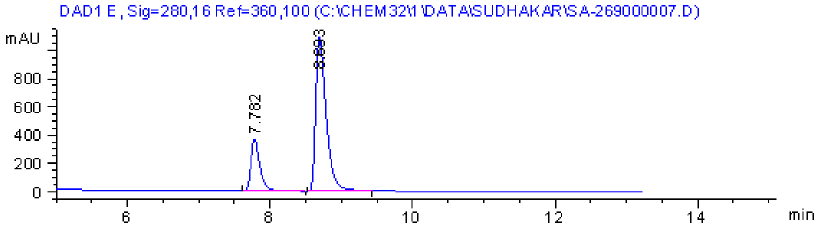

Signal 5: DAD1 E, Sig=280,16 Ref=360,100

Peak #	RetTime [min]	Type	Width [min]	Area [mAU*s]	Height [mAU]	Area %
1	7.782	BB	0.1300	3252.06348	372.02142	22.4012
2	8.693	BV	0.1558	1.12653e4	1097.32935	77.5988

Totals : 1.45174e4 1469.35077

Procedure for large scale synthesis of 4a:

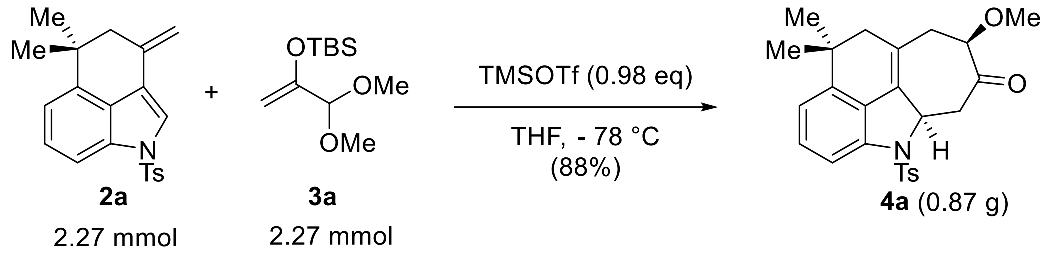

General procedure **D** was followed with **3a** (0.52 g, 2.27 mmol, 1 equiv.), **2a** (0.80 g, 2.27 mmol, 1 equiv.) in THF (45 mL), TMSOTf (0.40 mL, 2.23 mmol, 0.98 equiv.) to furnish **4a** (0.87 g, 88%) as a white fluffy solid after purification using the silica gel column chromatography (30% EtOAc:hexanes).

References

1. V. Kumar, H. N. K. Bulumulla, W. R. Wimalasiri, J. Reisch, *Phytochemistry* **1994**, *36*, 879.

2. T. Okauchi, M. Itonaga, T. Minami, T. Owa, K. Kitoh, H. Yoshino, *Org. Lett*. **2000**, *2*, 1485.

3. (a) J. Bergmann, L. Venemalm, A. Gogoll, *Tetrahedron*, **1990**, *46*, 6067. (b) Chandra, A.; Johnston, J. N. *Angew. Chem. Int. Ed.* **2011**, *50*, 7641.

4. (a) S. Pierau, H. M. R. Hoffmann, *Synlett* **1999**, 213. (b) N. Pichon, A. Harrison-Marchand, L.Toupet, J. Maddaluno, *J. Org. Chem.* **2006**, *71*, 1892.

5. A. K. Sinhababu, R. T. Borchardt R, *J. Org Chem*. **1983**, *48*, 3347.

6. M. Terada, K. Moriya, K. Kanomata, K. Sorimachi, *Angew. Chem. Int. Ed*. **2011**, *50*, 12586.

7. N. P. Grimster, C. Gauntlett, C. R. A. Godfrey, M. J. Gaunt, *Angew. Chem., Int. Ed.,* **2005**, *44*, 3125.

8. (a) R. Settambolo, R. Lazzaroni, T. Messeri, M. Mazzetti, P. Salvadori, *J. Org. Chem*. **1993**, *58,* 7899. (b) J. W. Huffman, V. J. Smith, L. W. Padgett, *Tetrahedron* **2008**, *64*, 2104.

9. The amount of remaining starting material **4a** was determined based on ^{1}H NMR spectrum of the crude material.

Experimental Spectra

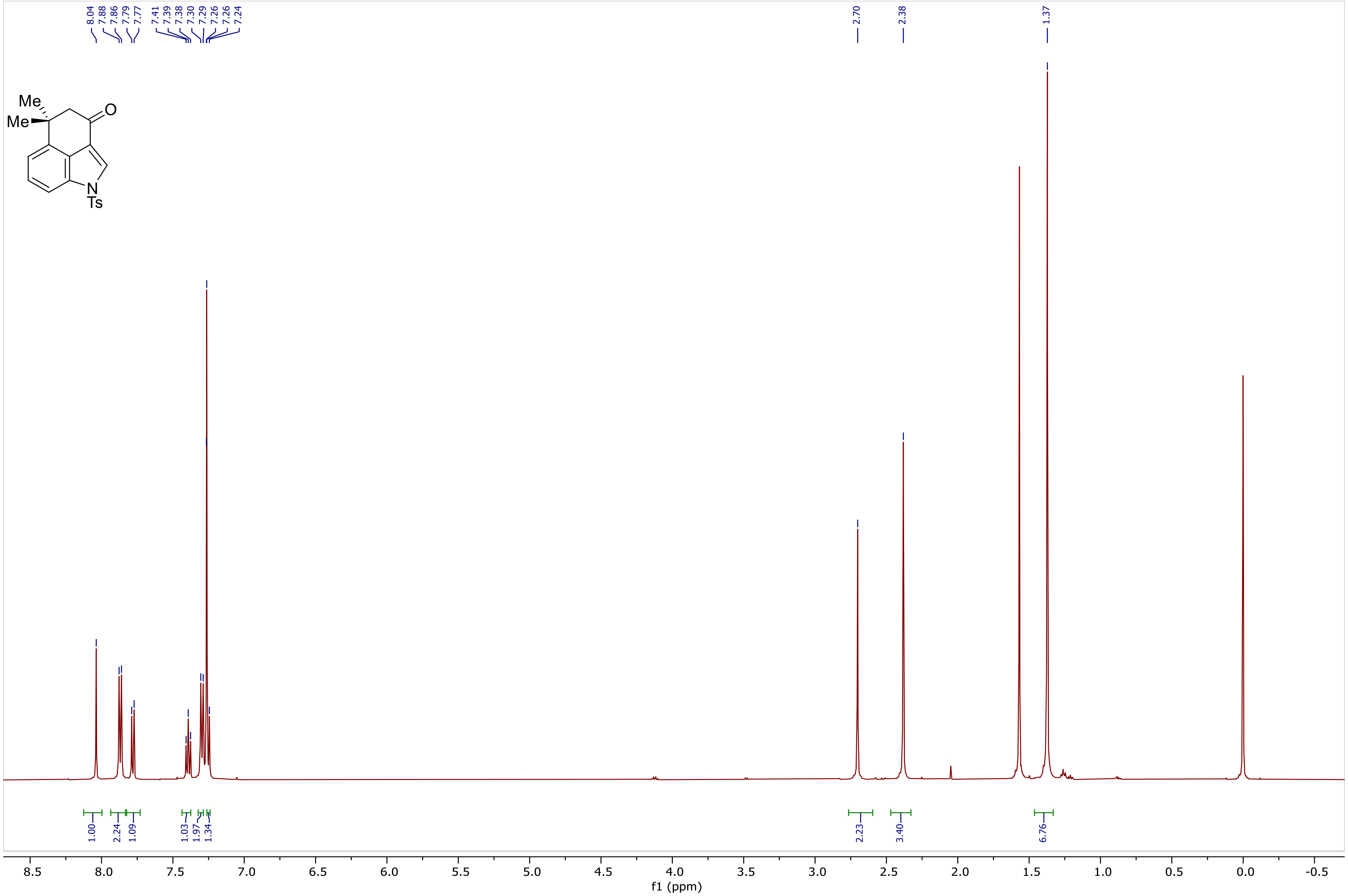

^{1}H NMR (500 MHz, $CDCl_3$) of compound **1**

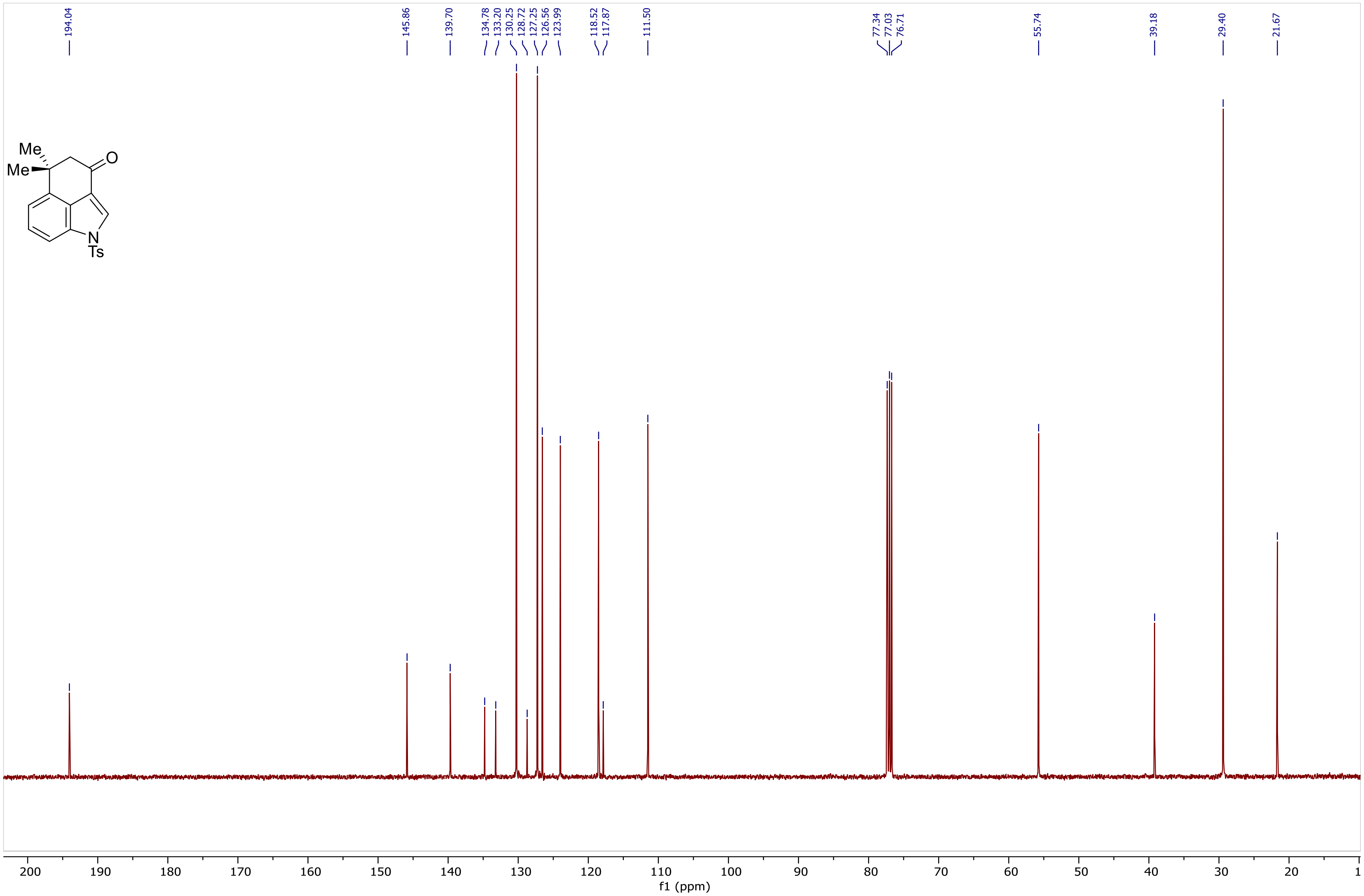

^{13}C NMR (100 MHz, $CDCl_3$) of compound **1**

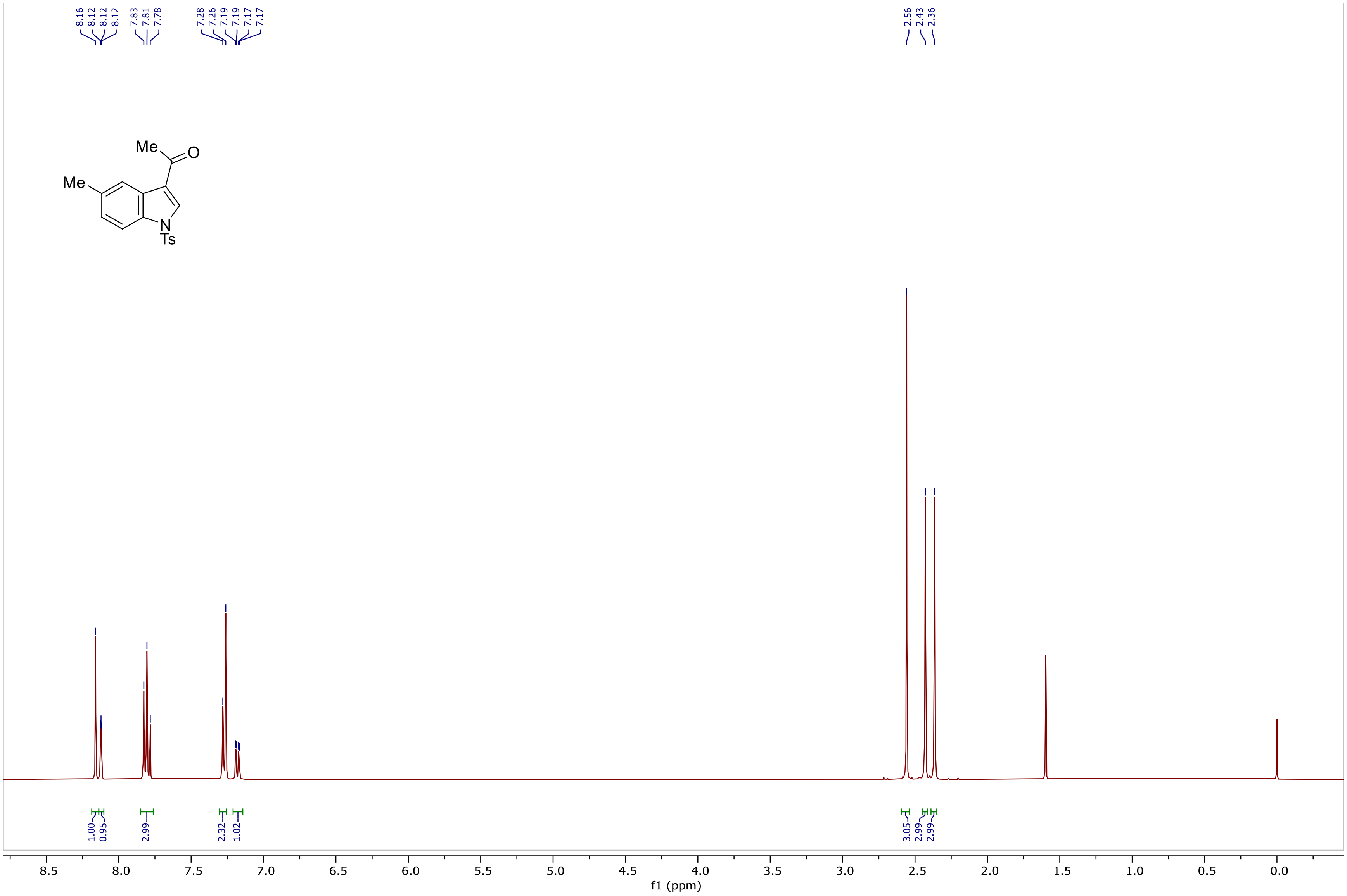

^{1}H NMR (400 MHz, $CDCl_3$) of compound **1c**

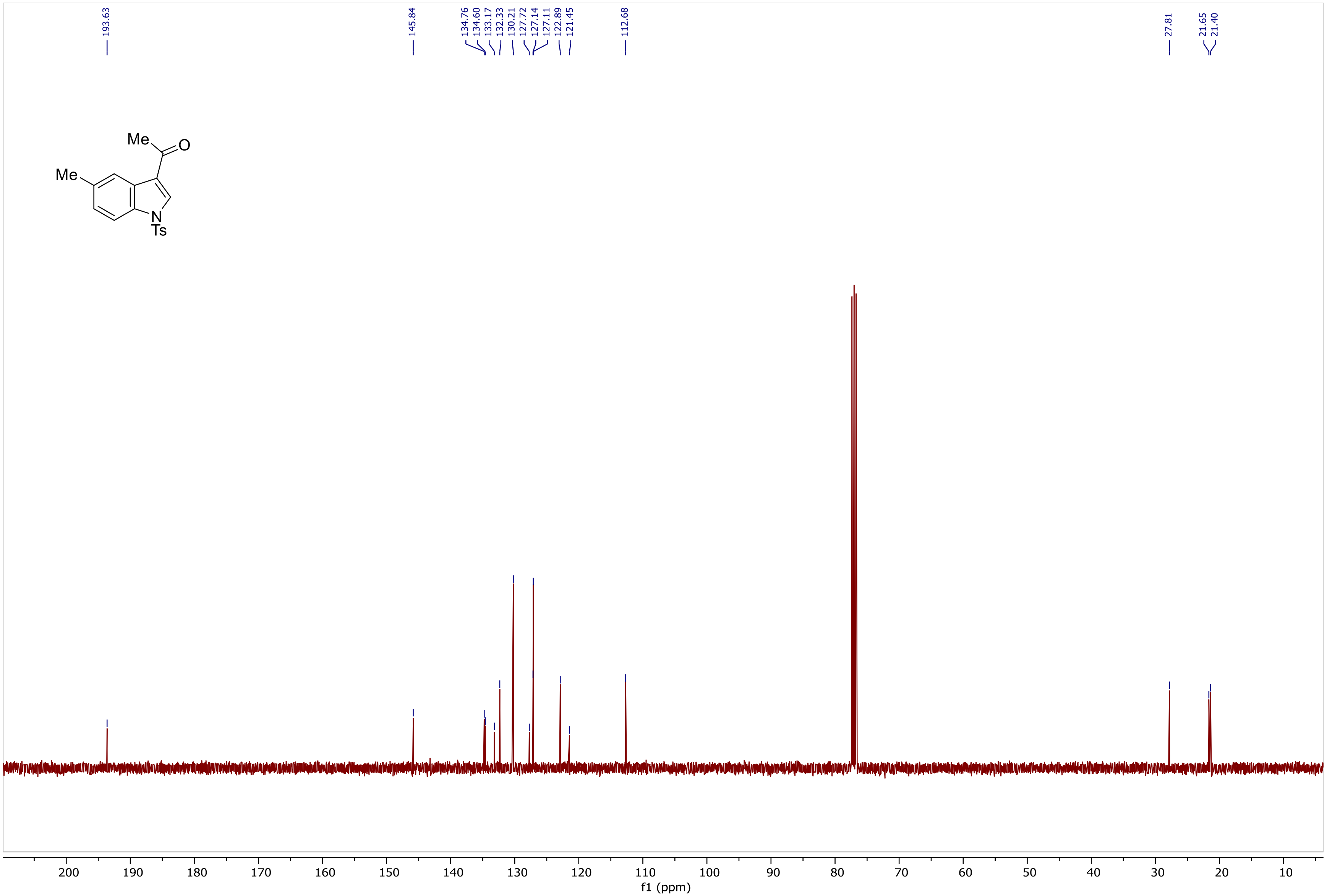

^{13}C NMR (101 MHz, $CDCl_3$) of compound **1c**

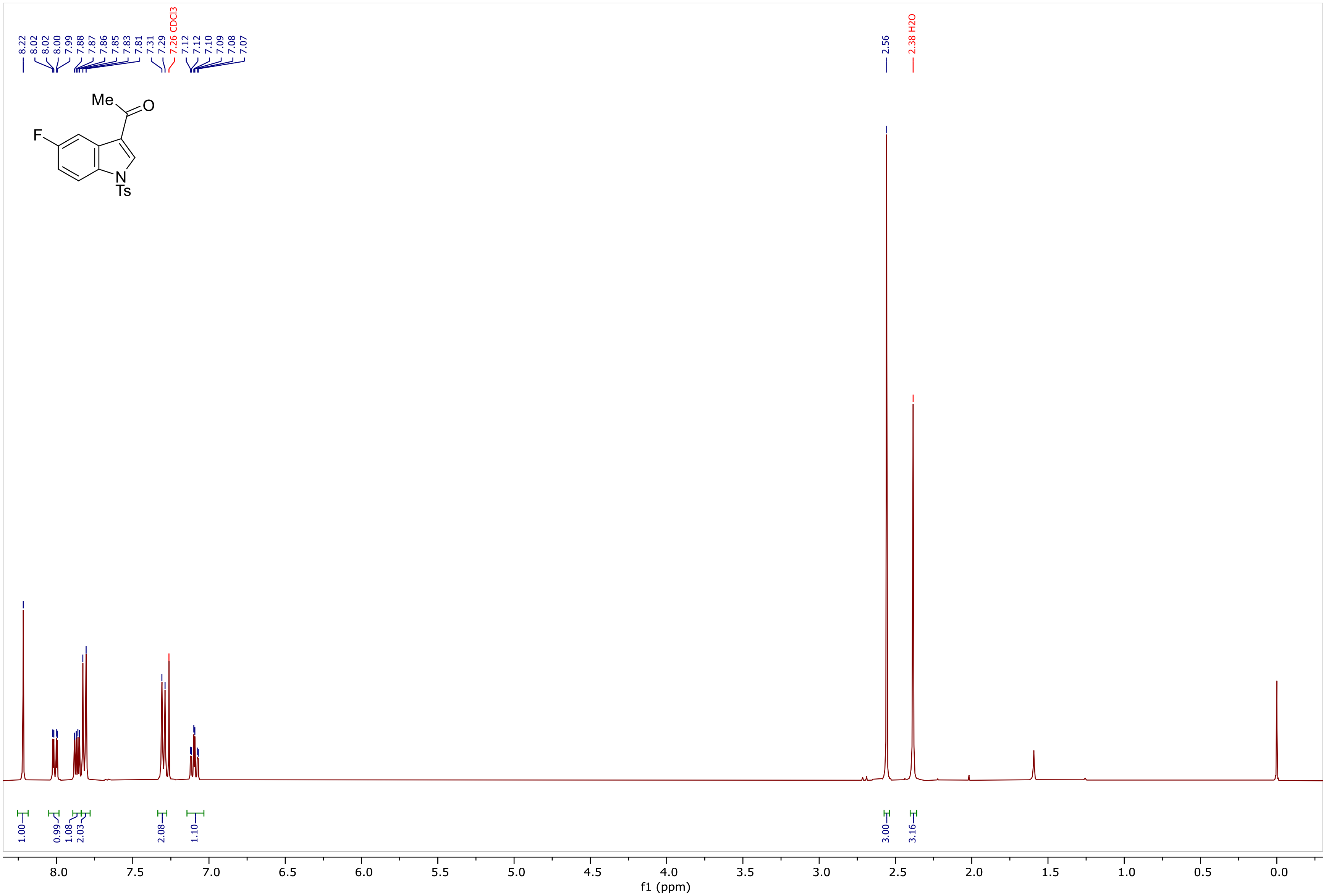

^{1}H NMR (400 MHz, $CDCl_3$) of compound **1d**

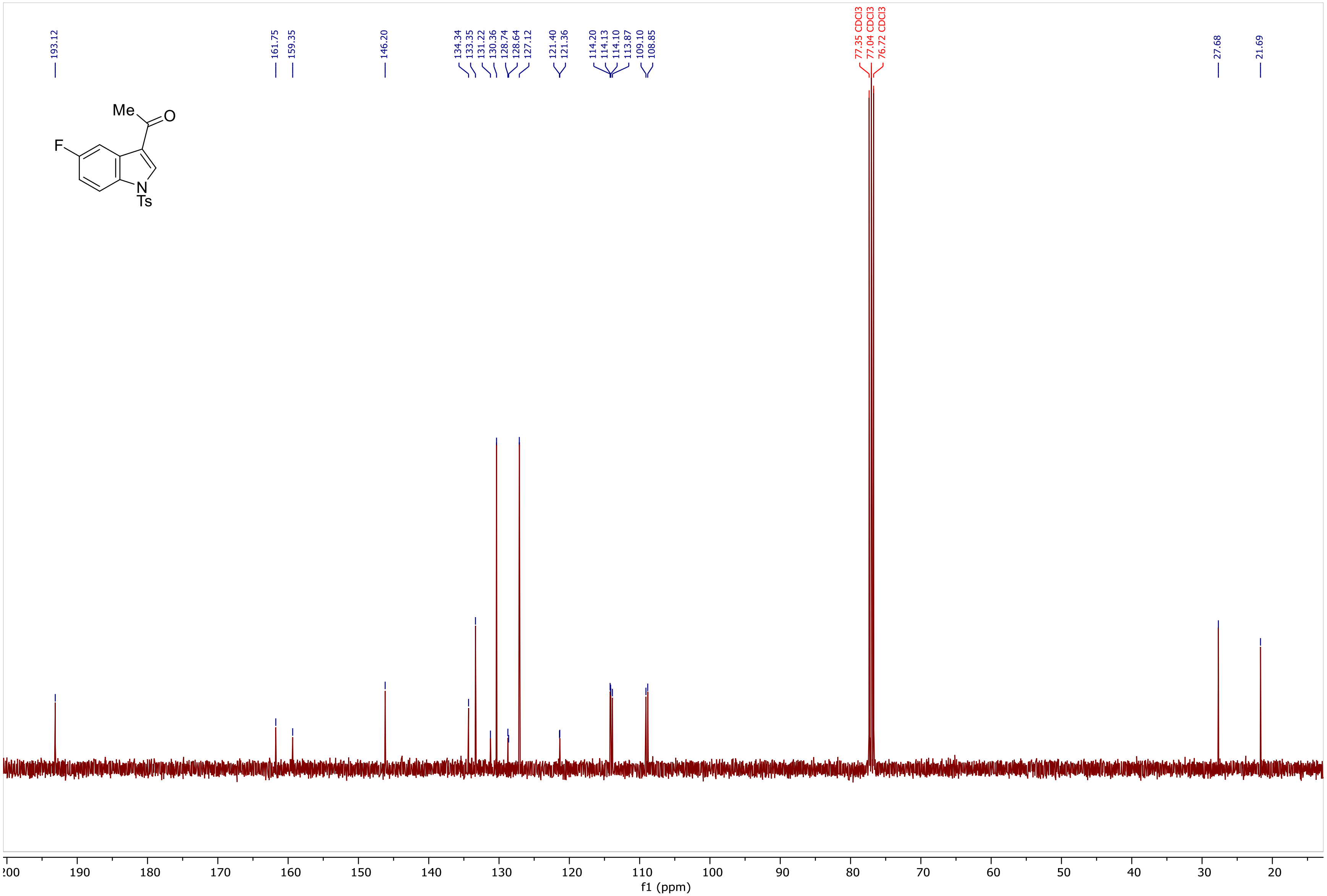

^{13}C NMR (101 MHz, $CDCl_3$) of compound **1d**

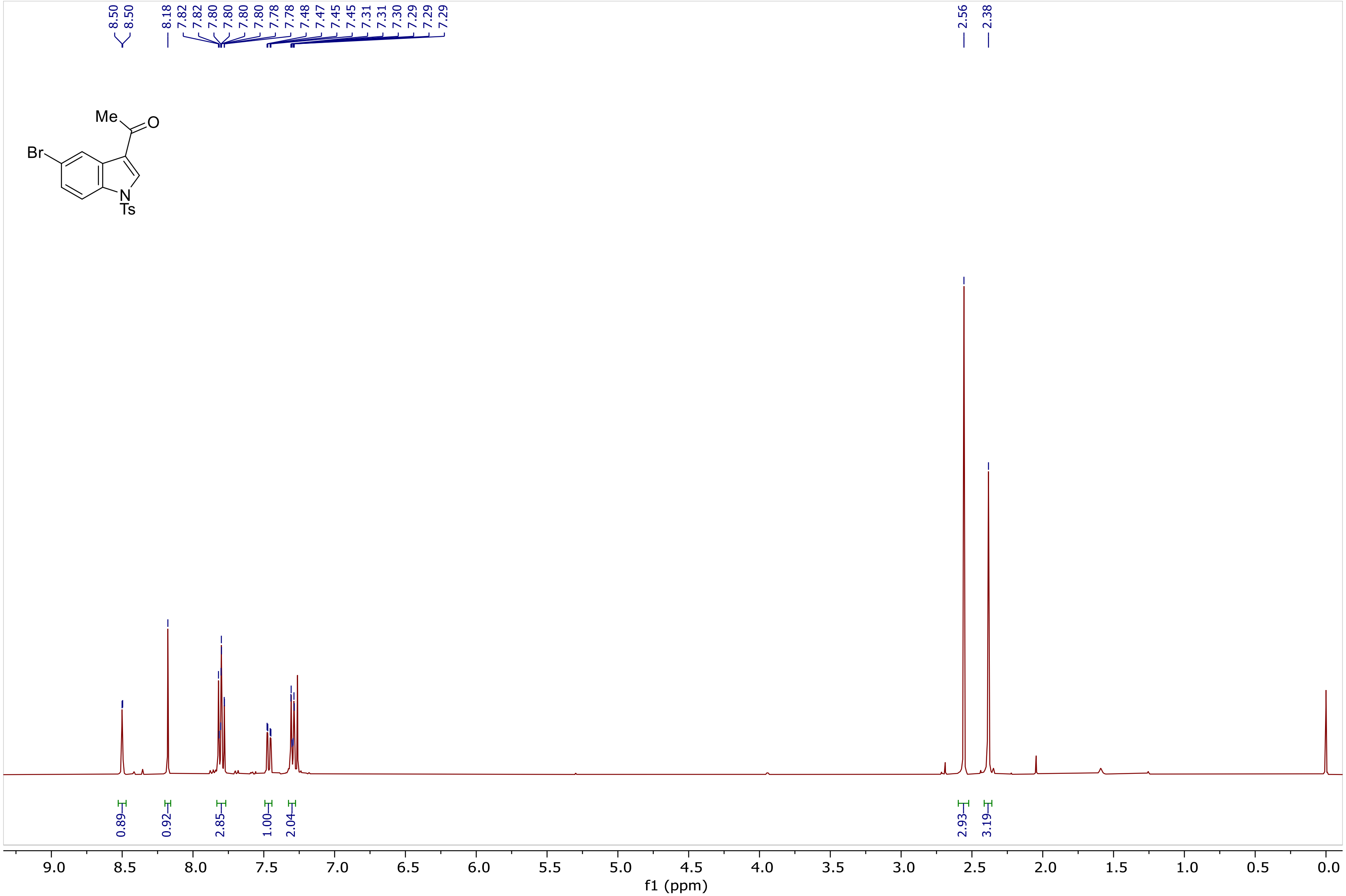

^{1}H NMR (400 MHz, $CDCl_3$) of compound **1e**

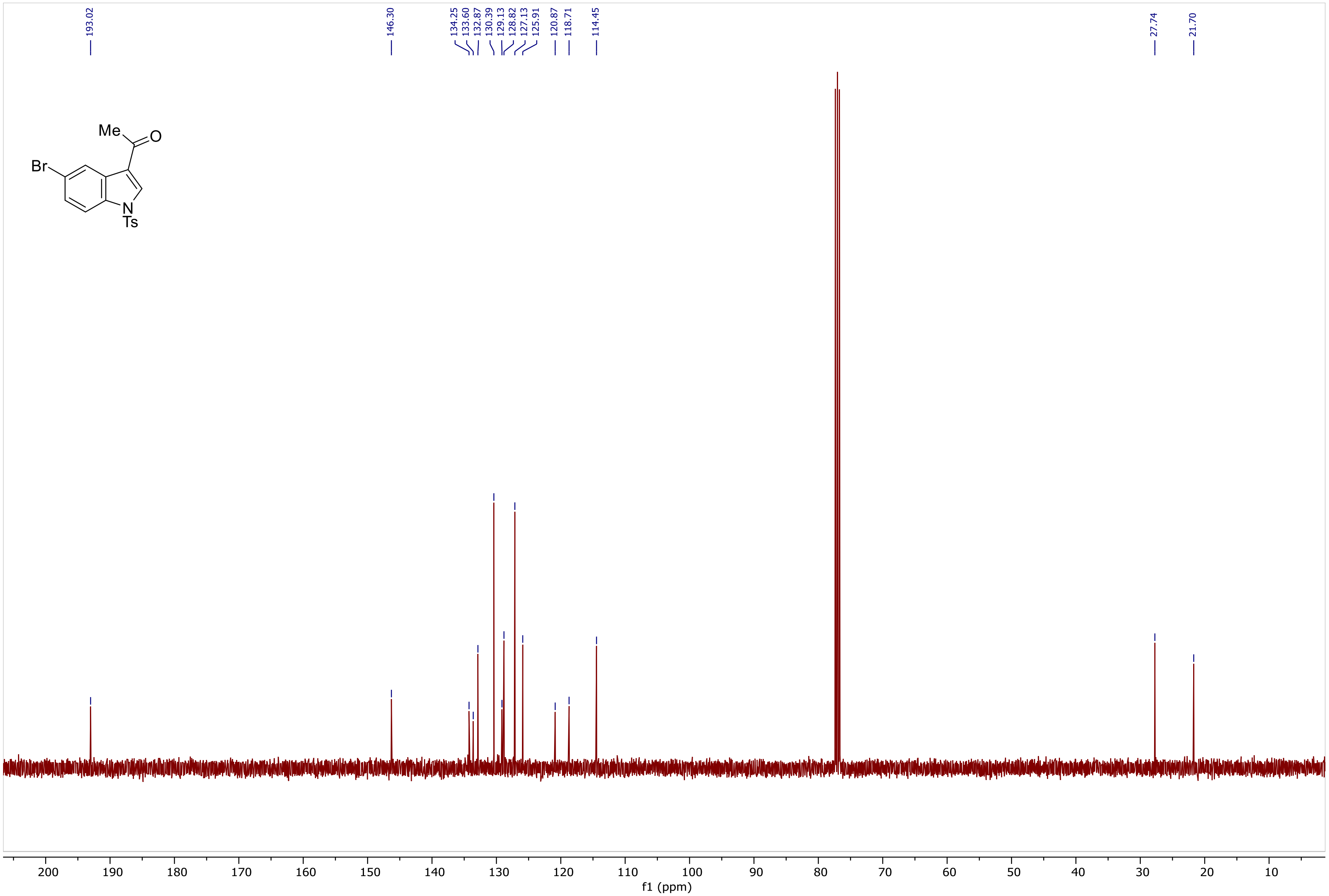

^{13}C NMR (101 MHz, $CDCl_3$) of compound **1e**

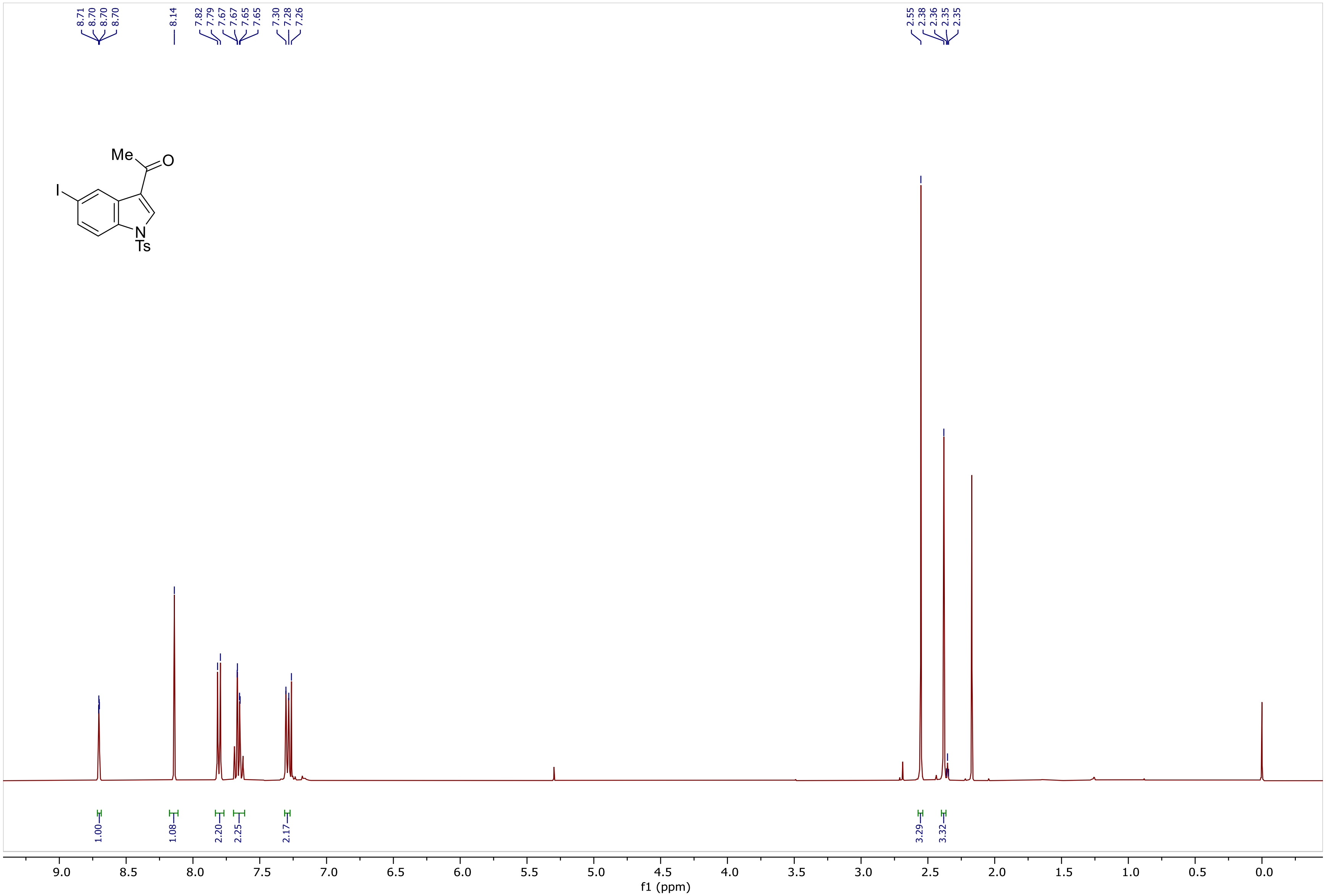

^{1}H NMR (400 MHz, $CDCl_3$) of compound **1f**

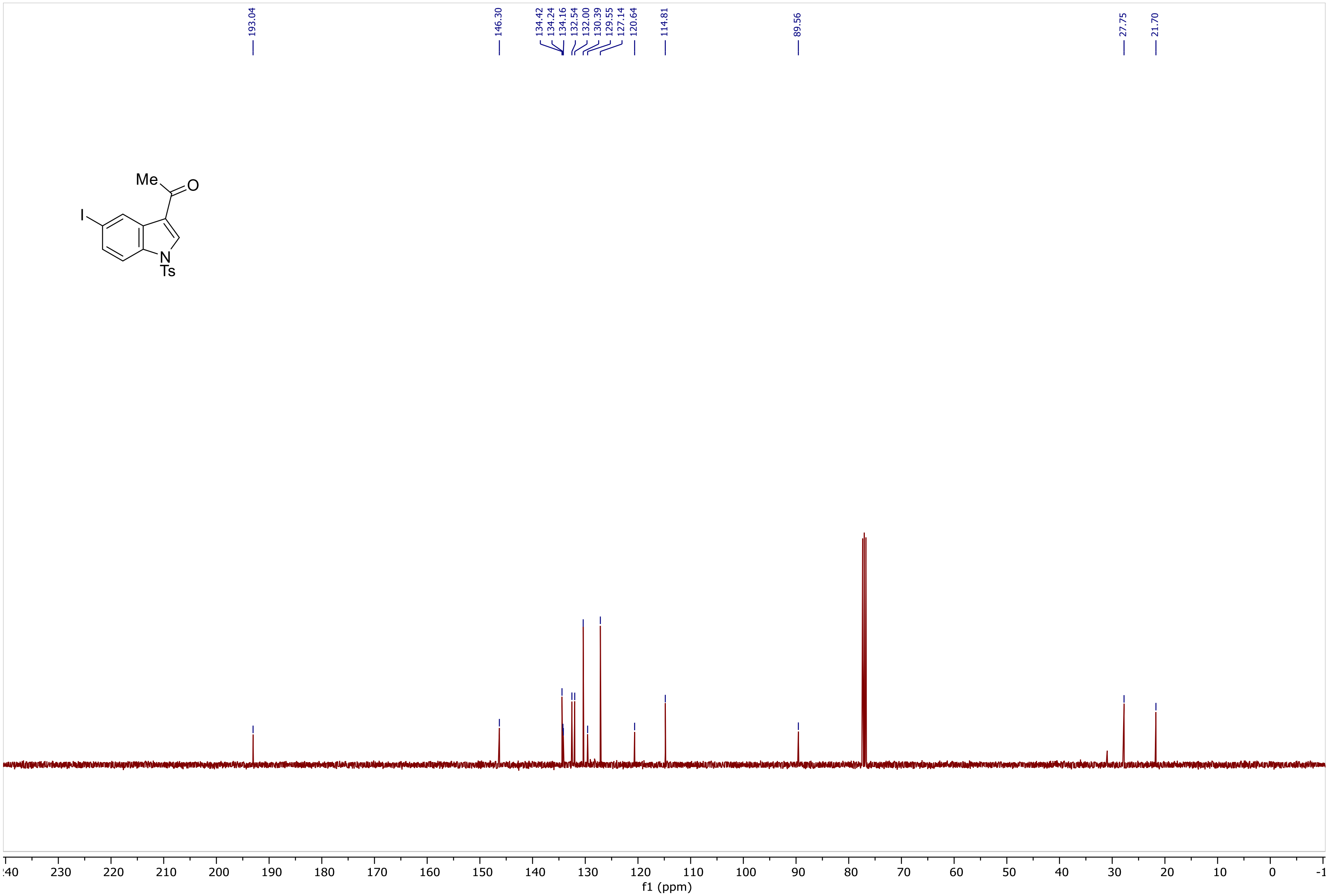

^{13}C NMR (101 MHz, $CDCl_3$) of compound **1f**

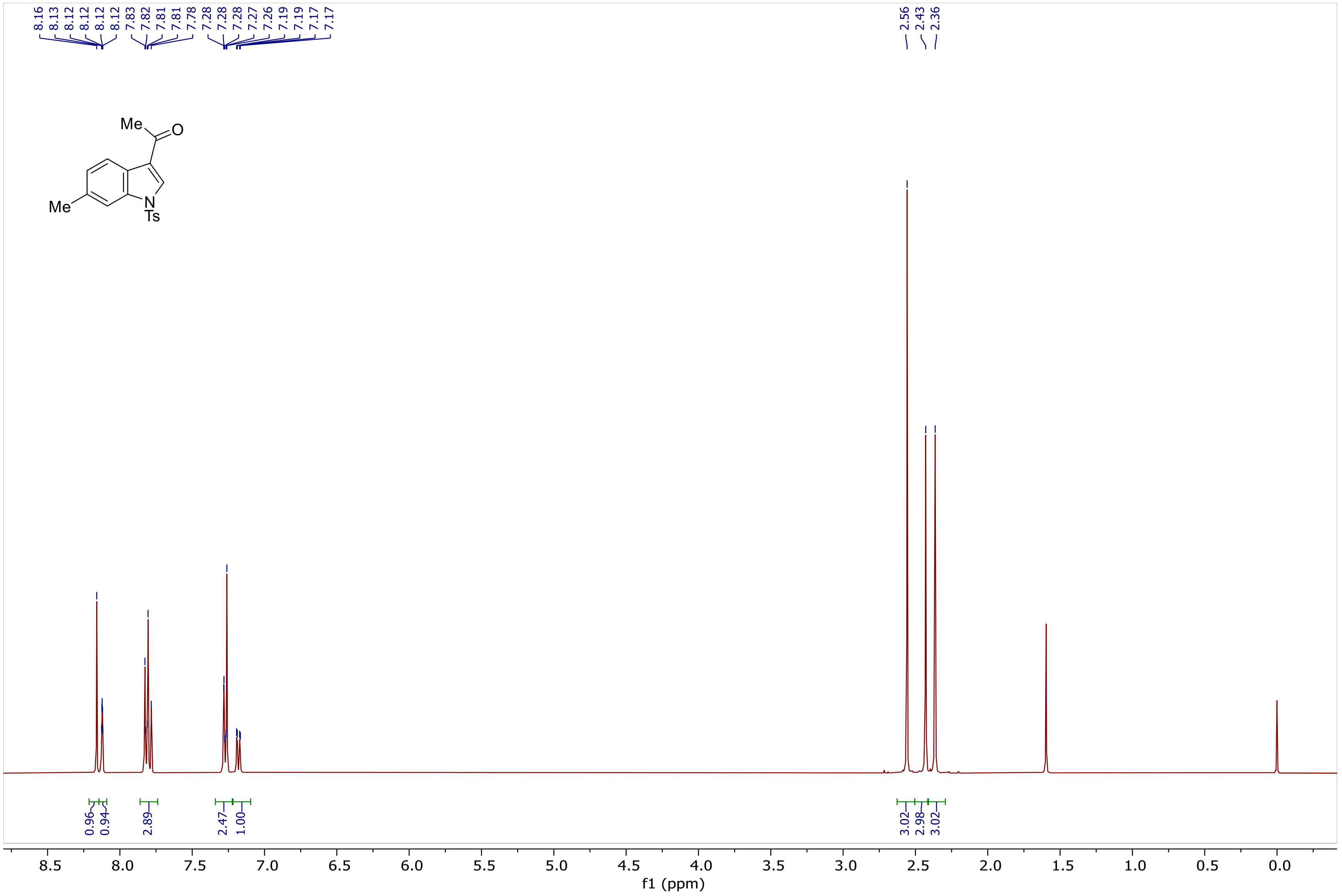

^{1}H NMR (400 MHz, $CDCl_3$) of compound **1g**

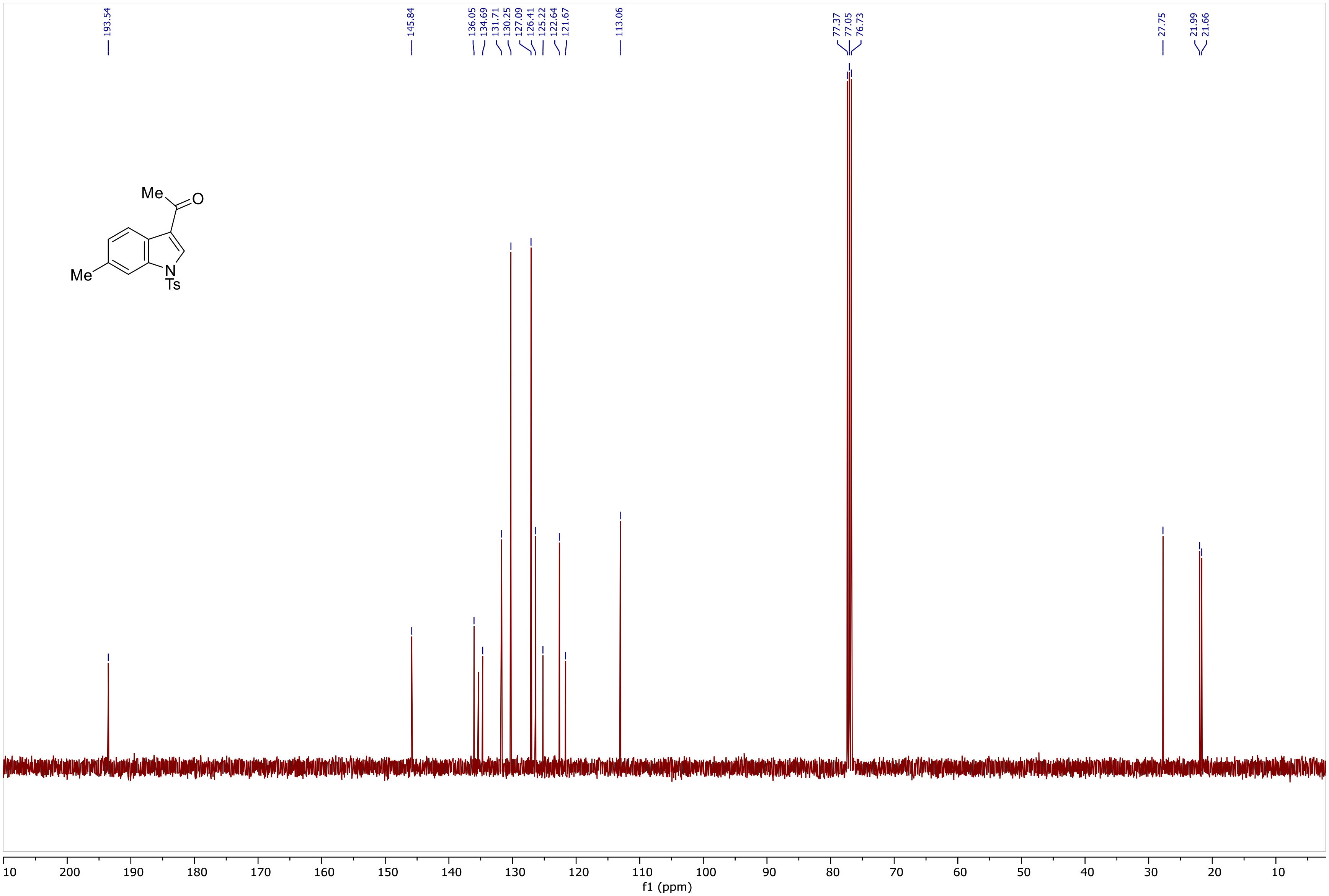

^{13}C NMR (101 MHz, $CDCl_3$) of compound **1g**

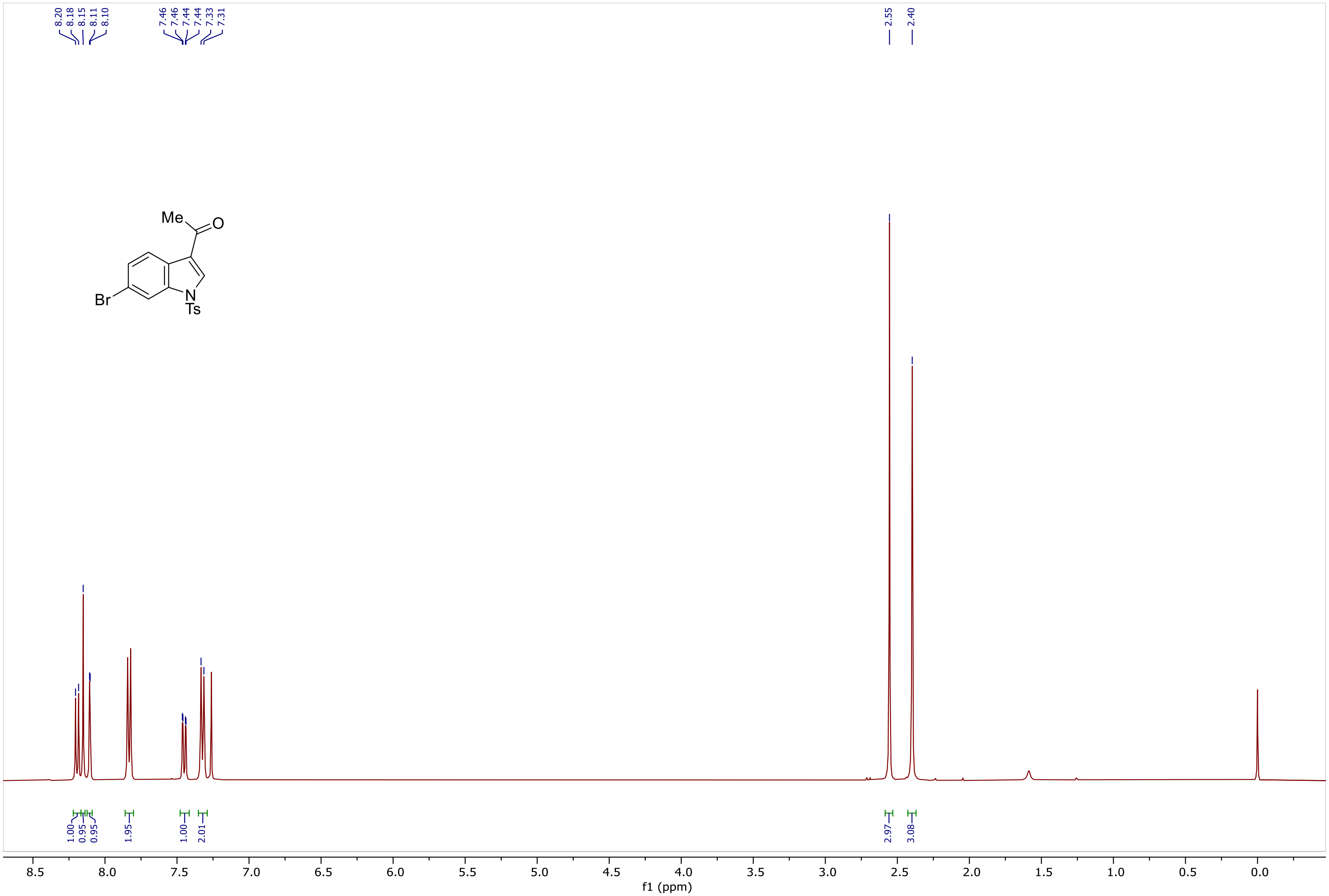

^{1}H NMR (400 MHz, $CDCl_3$) of compound **1h**

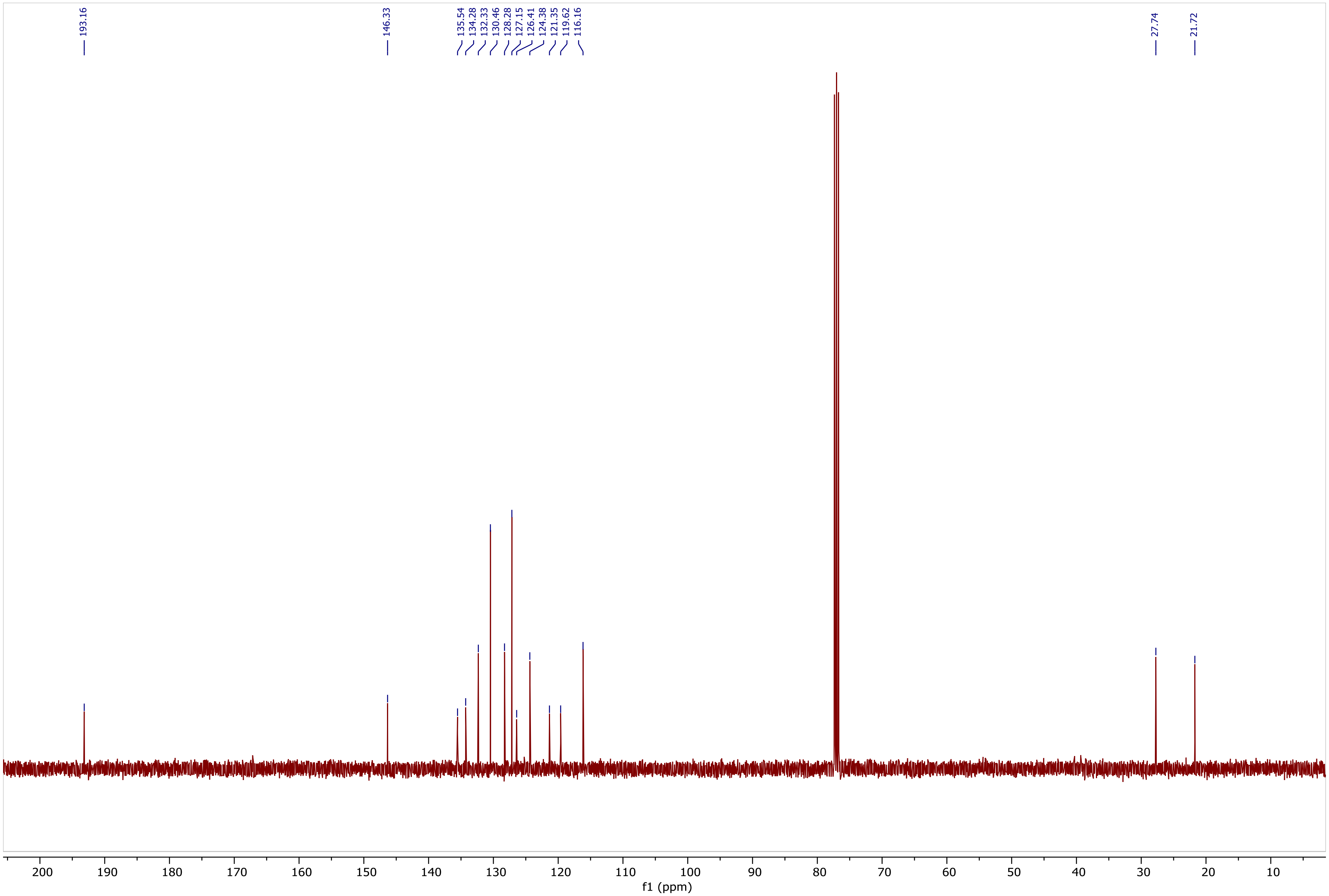

^{13}C NMR (101 MHz, $CDCl_3$) of compound **1h**

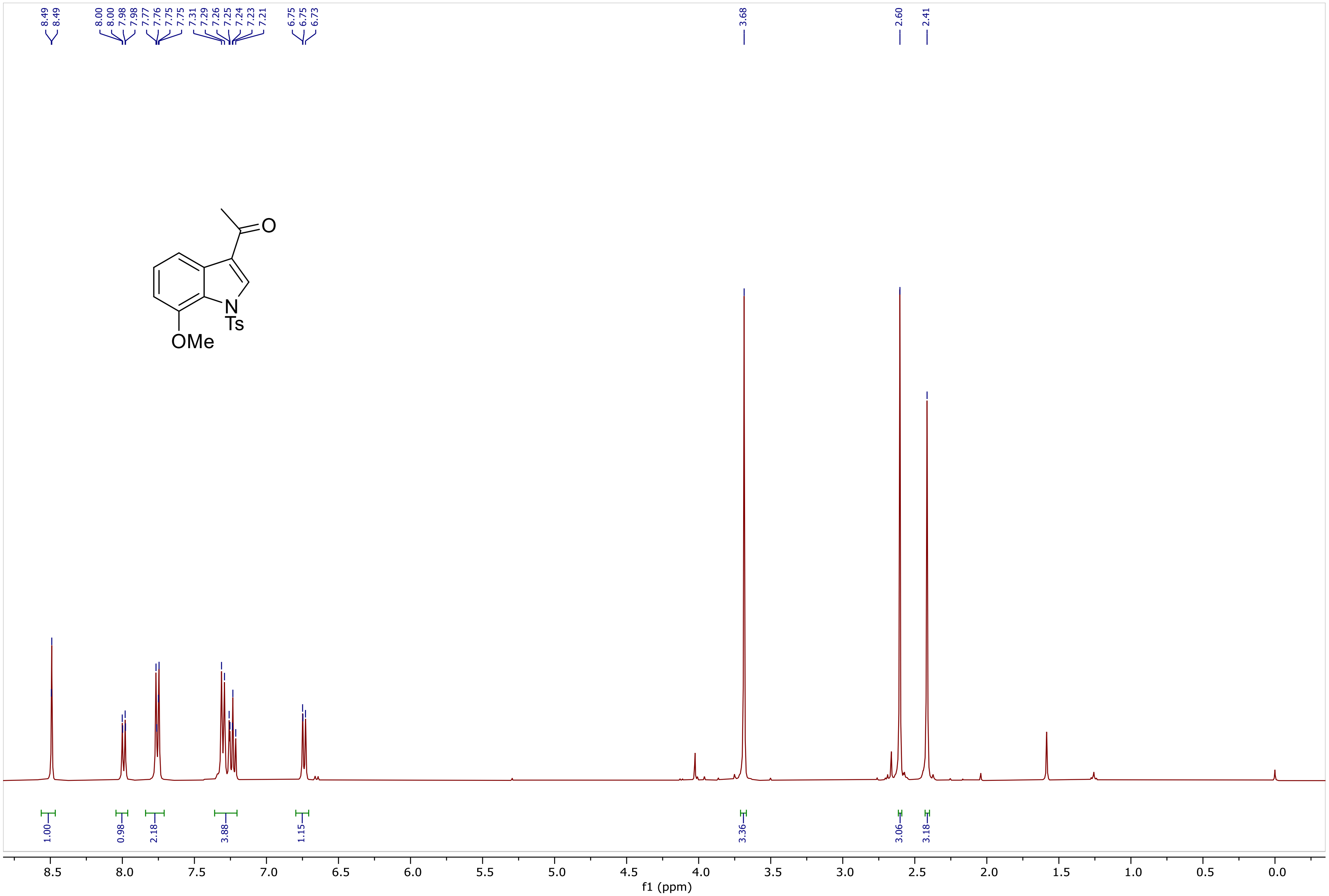

^{1}H NMR (400 MHz, $CDCl_3$) of compound **1i**

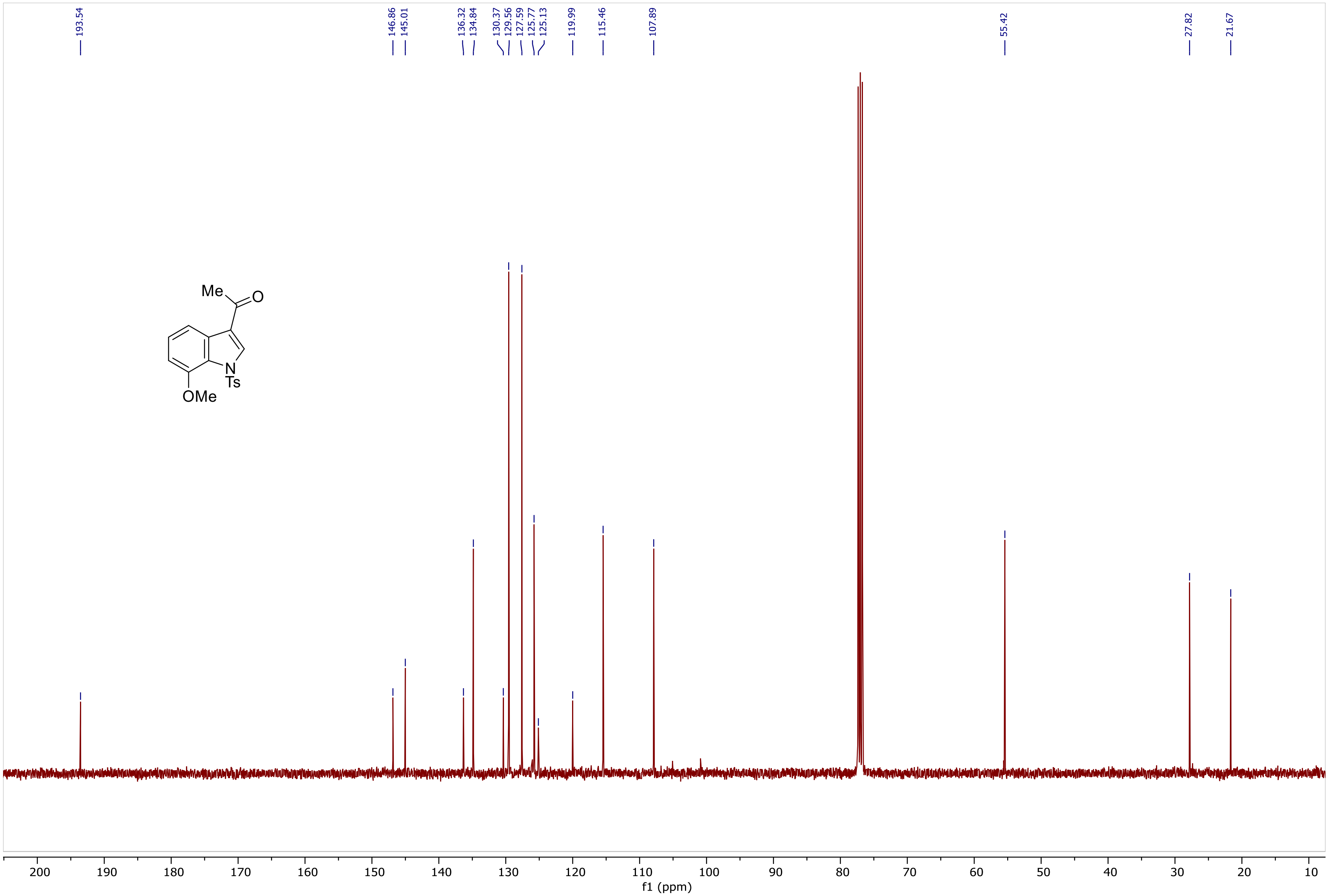

^{13}C NMR (101 MHz, $CDCl_3$) of compound **1i**

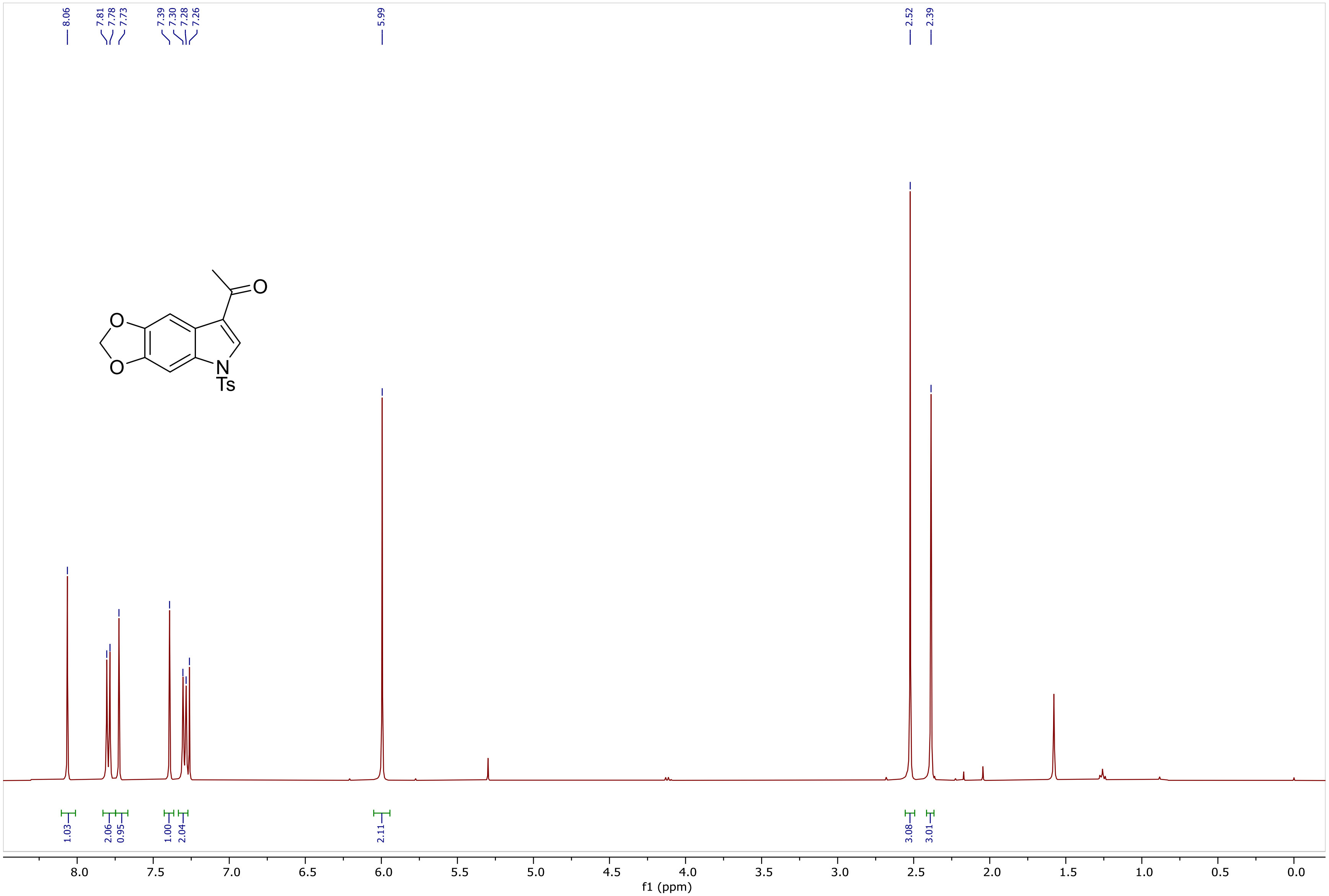

^{1}H NMR (400 MHz, $CDCl_3$) of compound **1j**

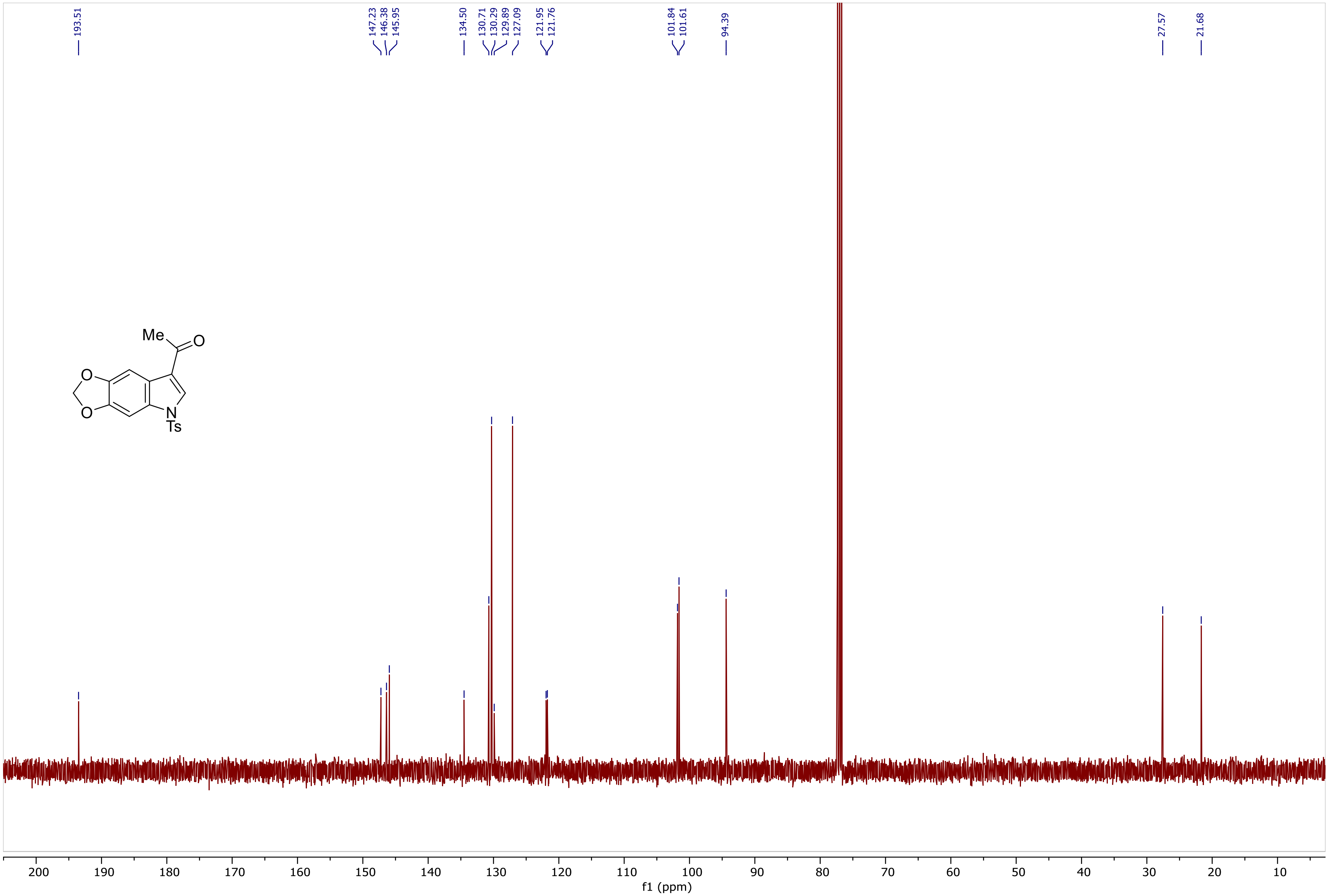

^{13}C NMR (101 MHz, $CDCl_3$) of compound **1j**

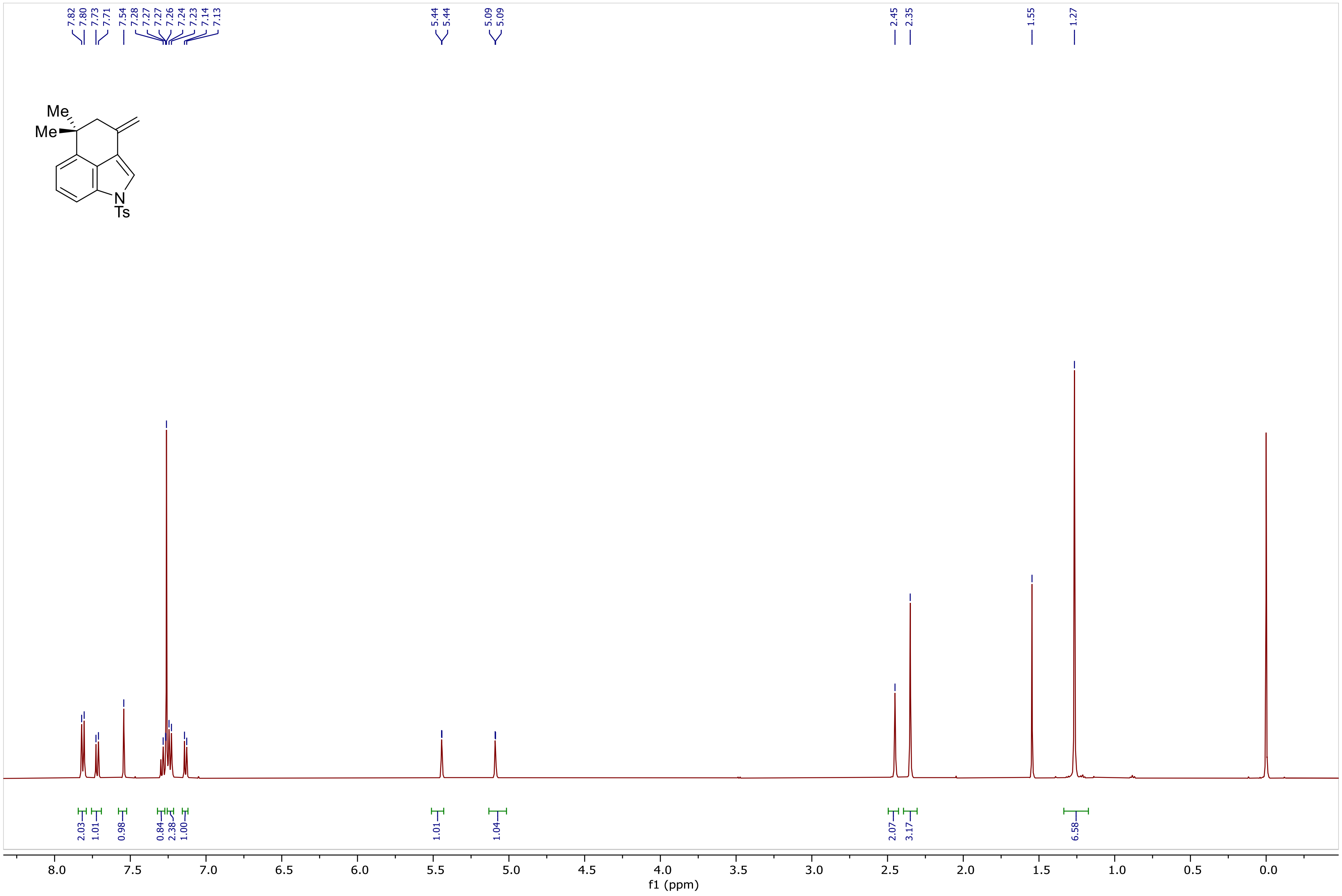

^{1}H NMR (400 MHz, $CDCl_3$) of compound **2a**

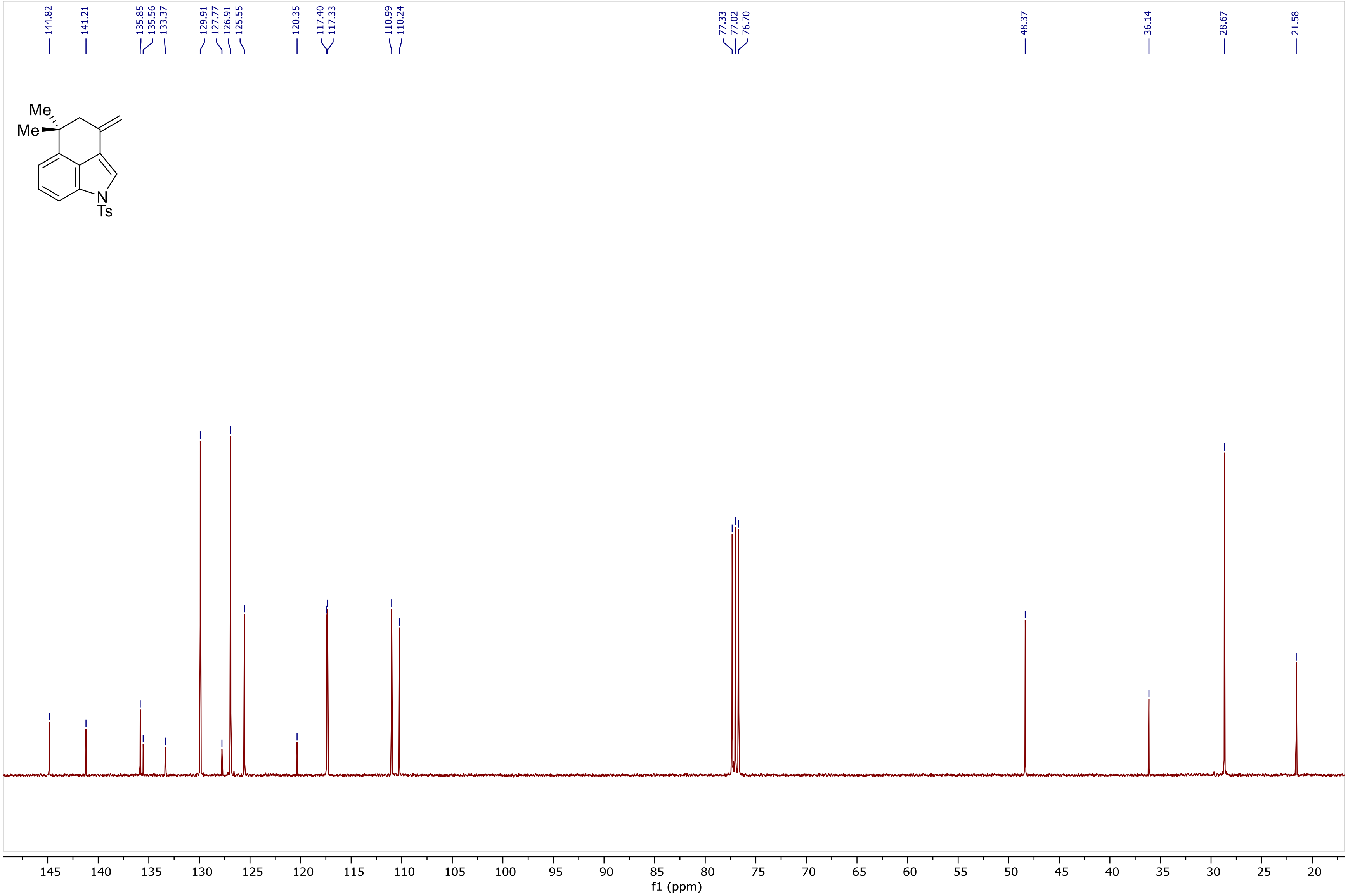

^{13}C NMR (101 MHz, $CDCl_3$) of compound **2a**

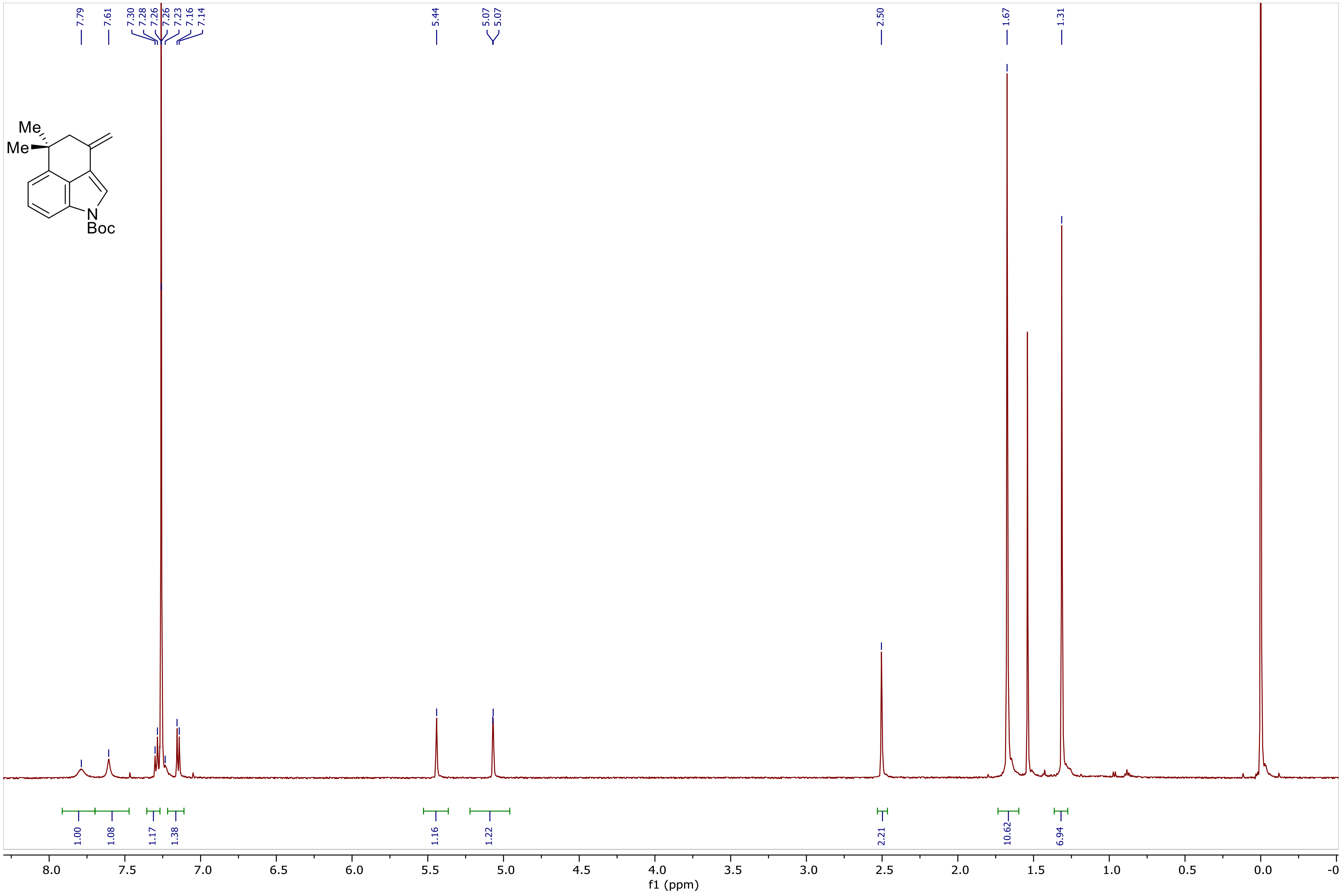

^{1}H NMR (400 MHz, $CDCl_3$) of compound **2d**

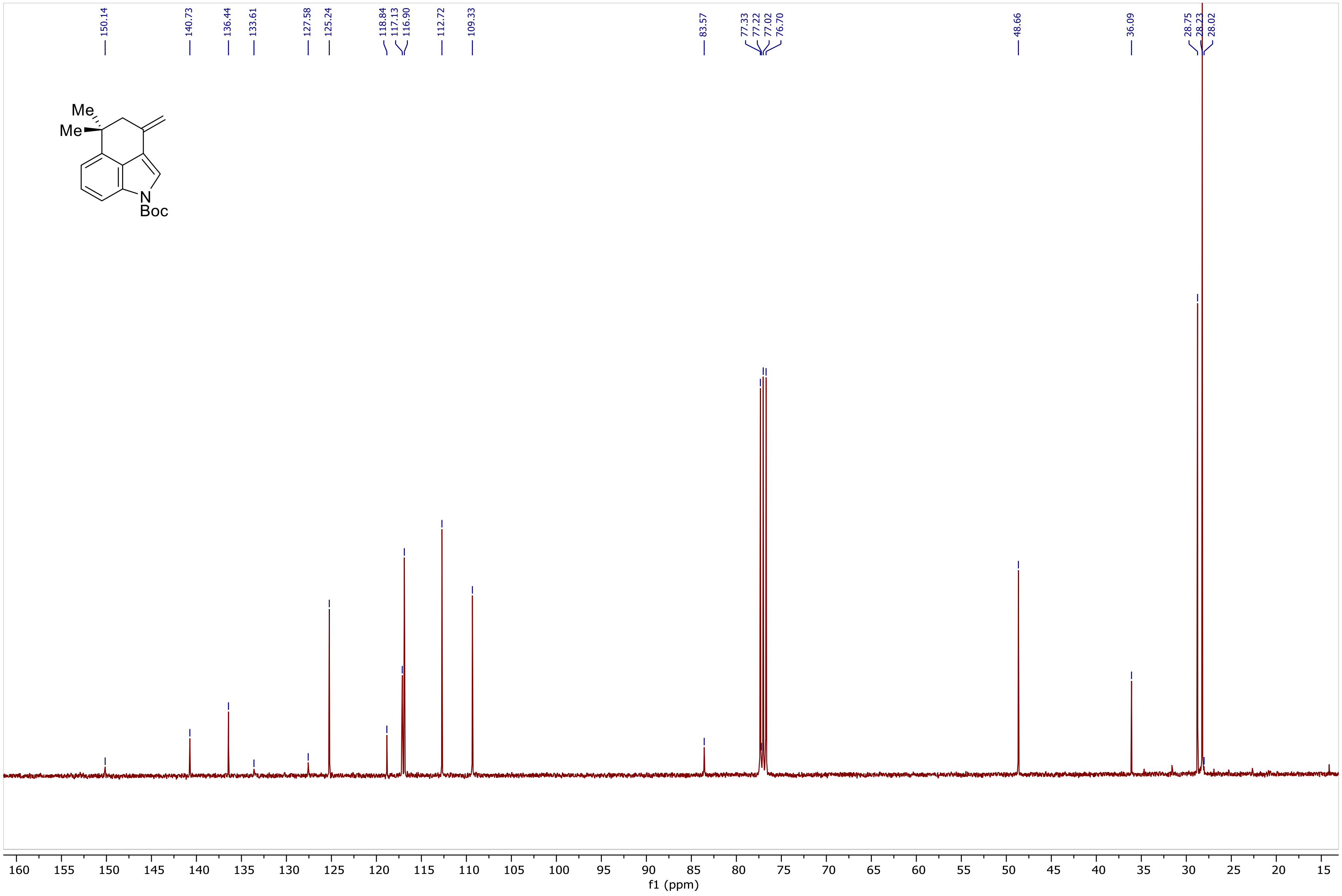

^{13}C NMR (101 MHz, $CDCl_3$) of compound **2d**

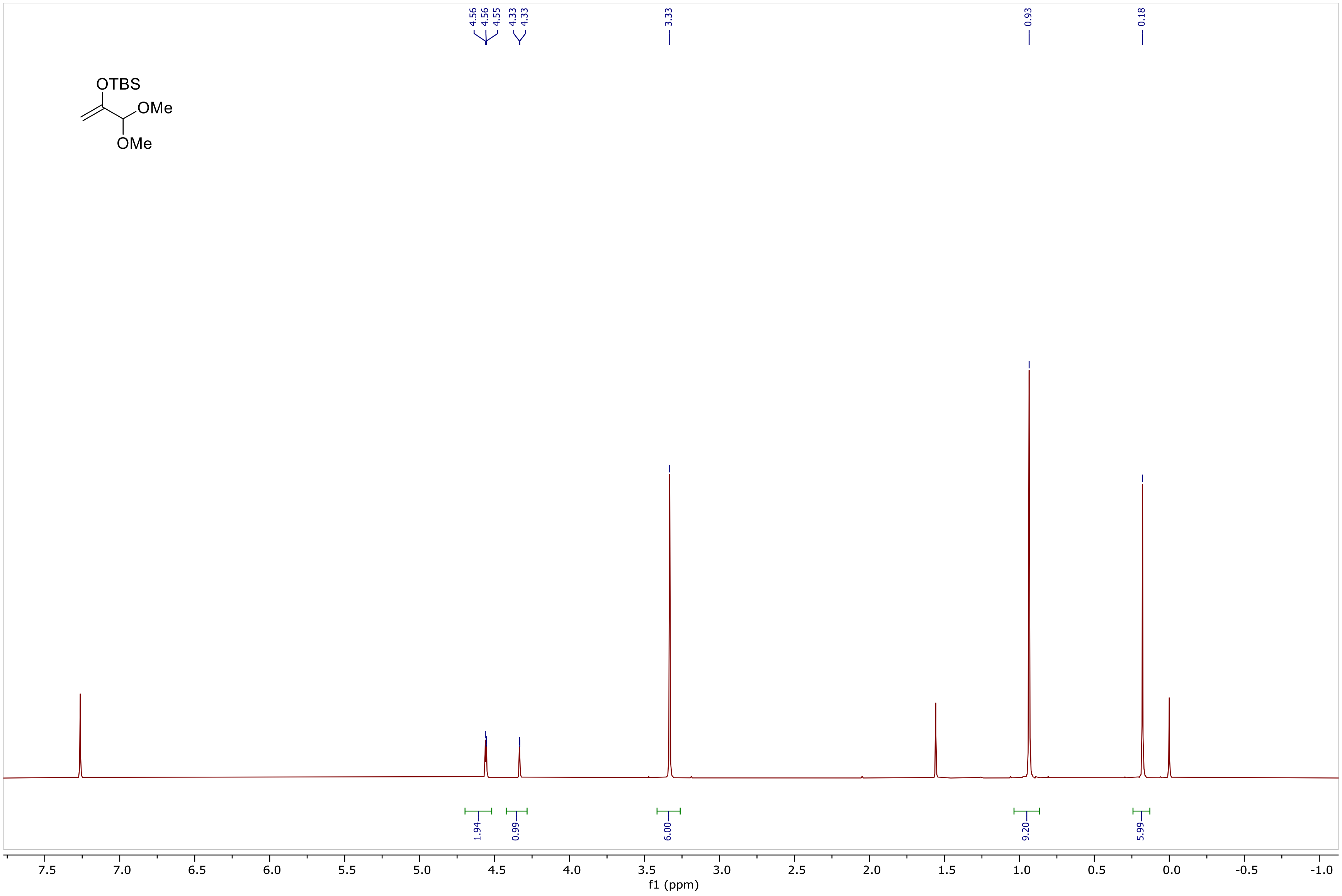

^{1}H NMR (400 MHz, $CDCl_3$) of compound **3a**

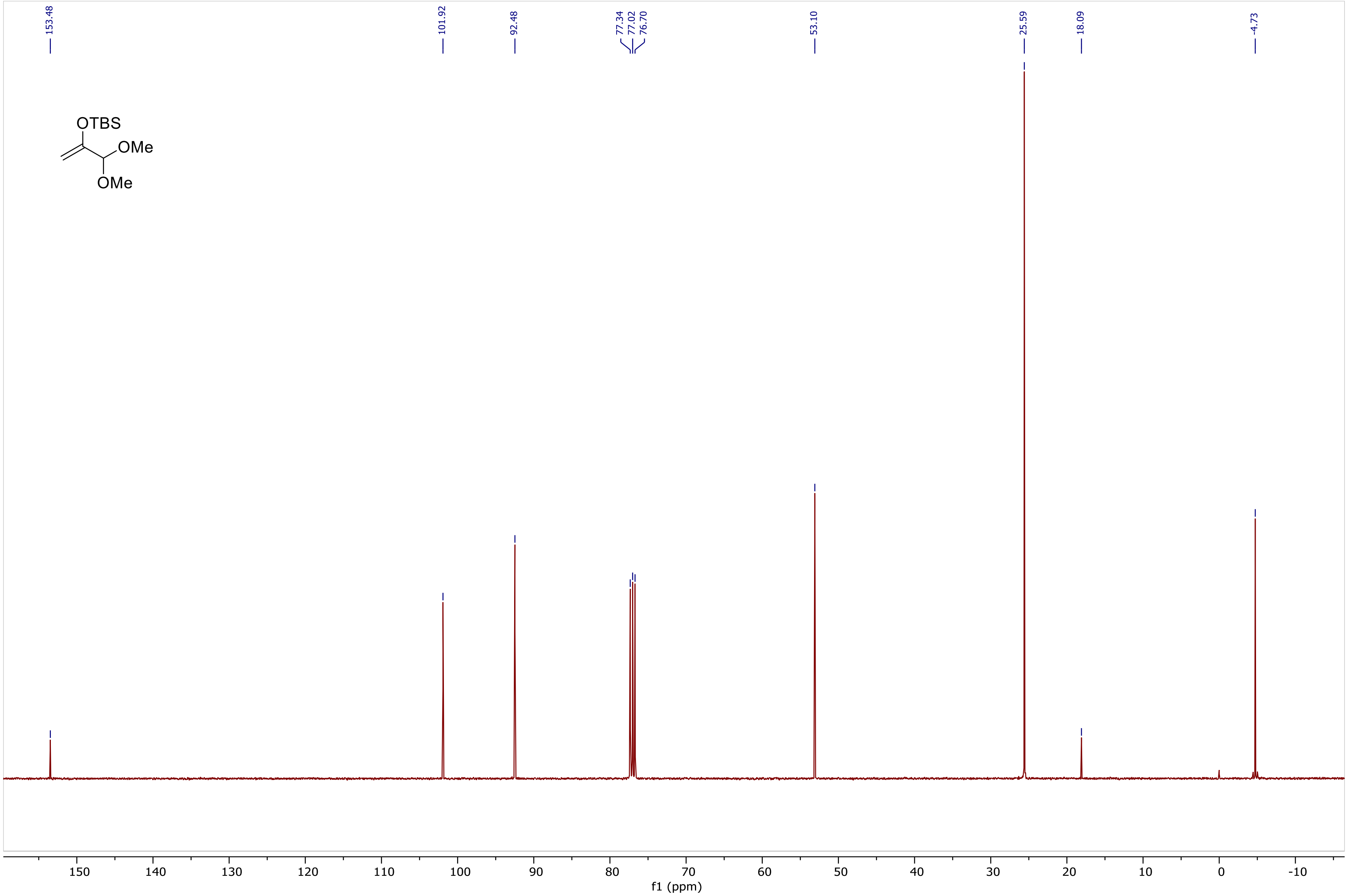

^{13}C NMR (101 MHz, $CDCl_3$) of compound **3a**

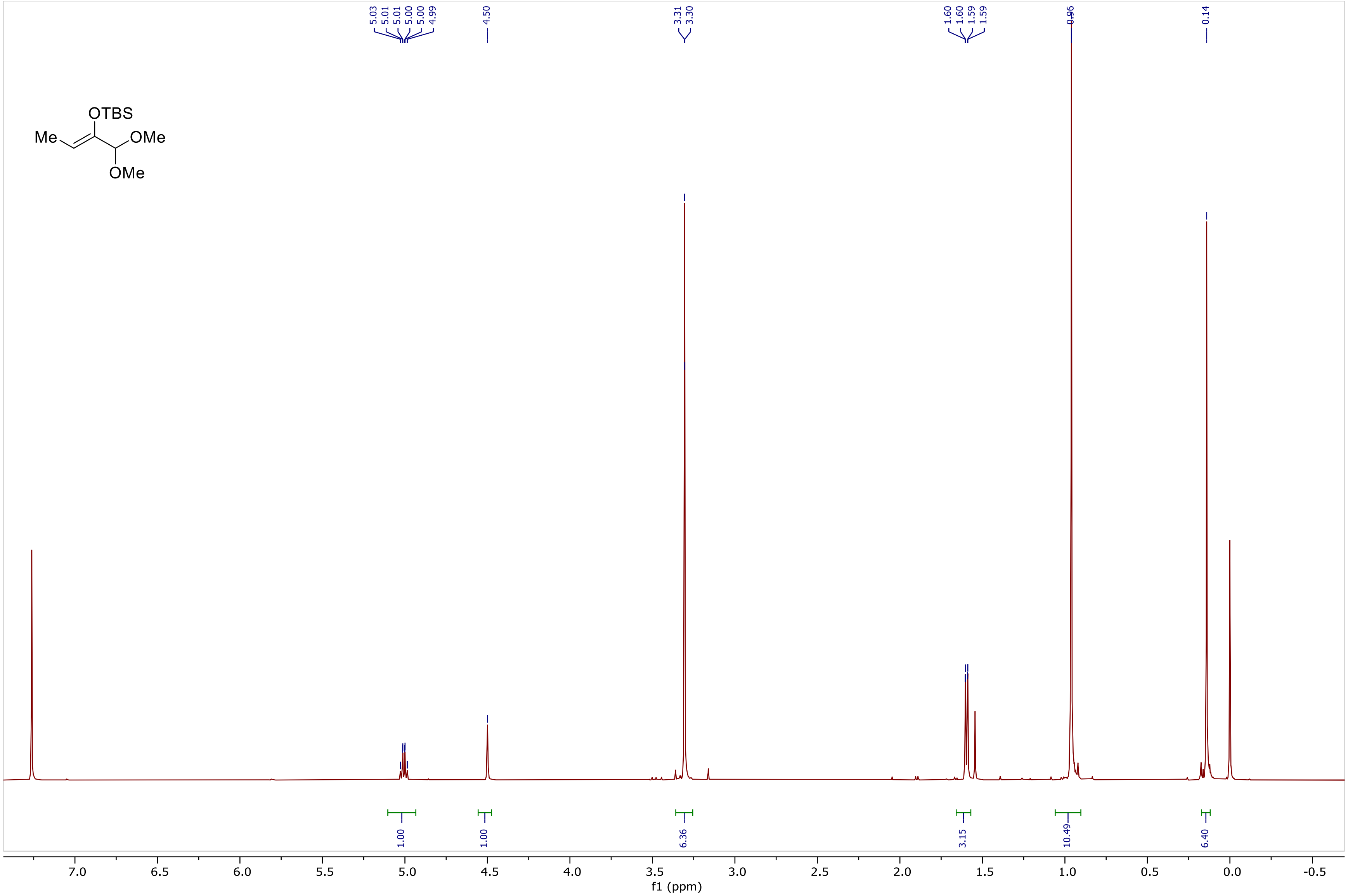

^{1}H NMR (400 MHz, $CDCl_3$) of compound **3b**

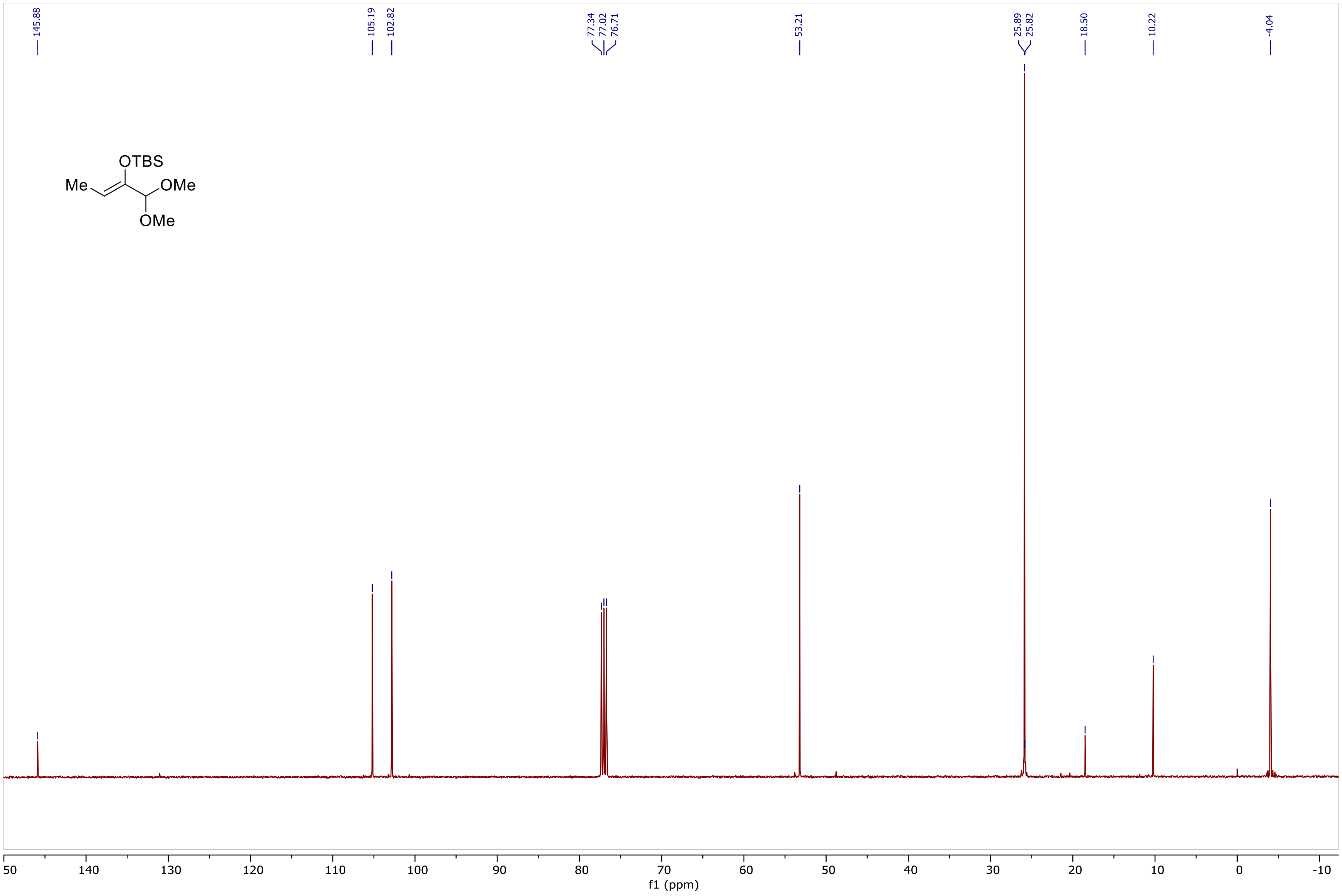

^{13}C NMR (101 MHz, $CDCl_3$) of compound **3b**

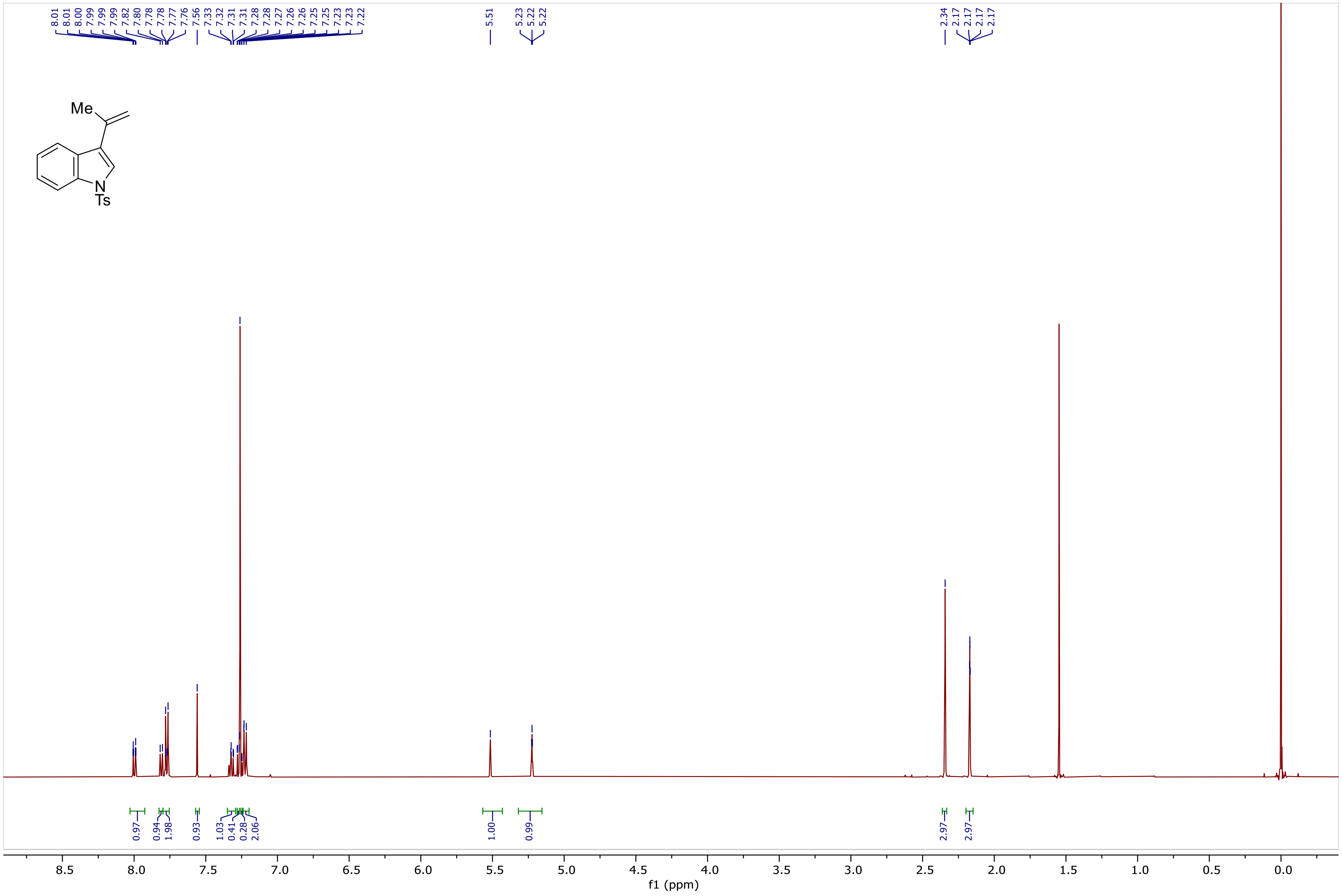

^{1}H NMR (400 MHz, $CDCl_3$) of compound **5a**

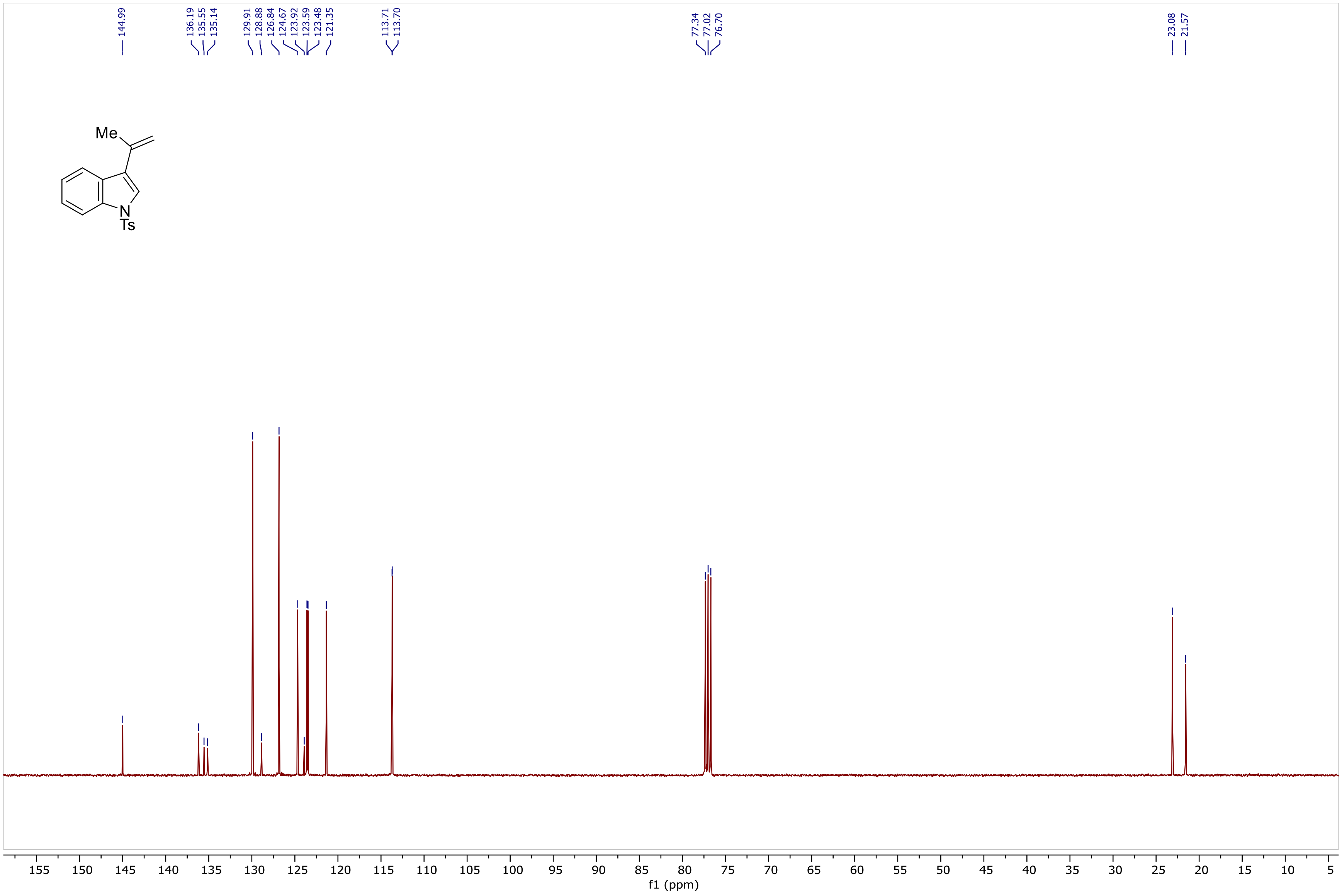

^{13}C NMR (101 MHz, $CDCl_3$) of compound **5a**

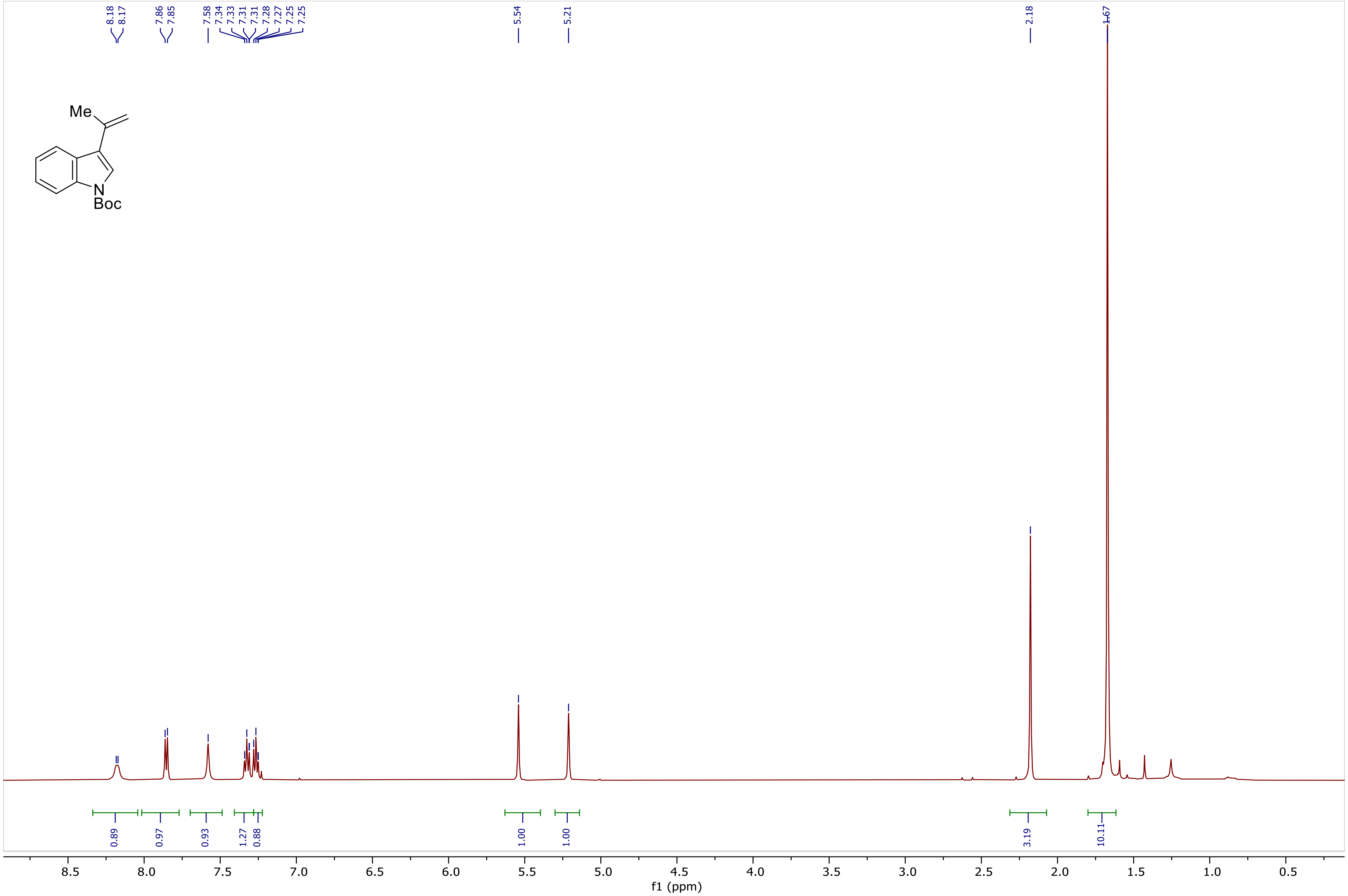

^{1}H NMR (400 MHz, $CDCl_3$) of compound **5b**

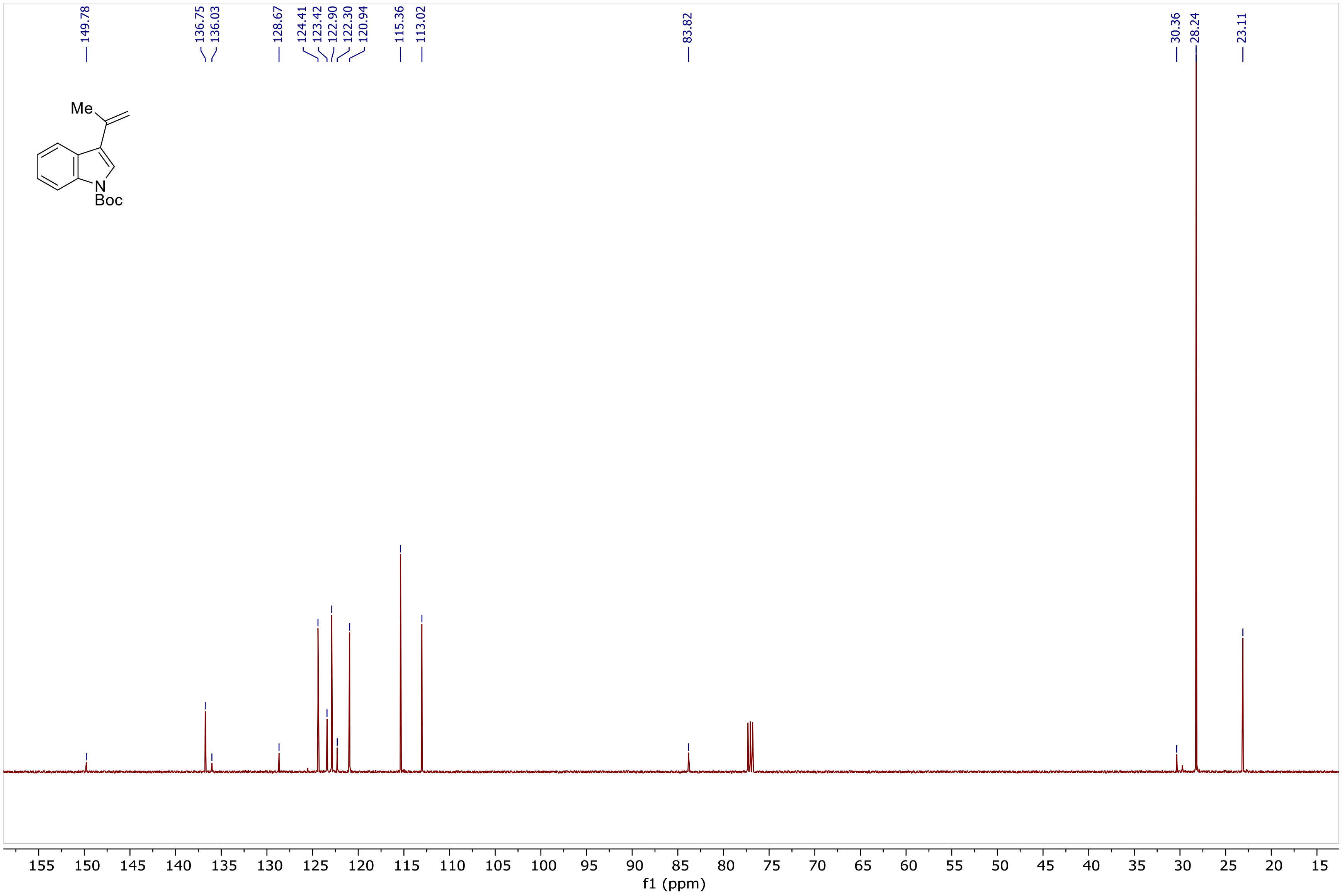

^{13}C NMR (101 MHz, $CDCl_3$) of compound **5b**

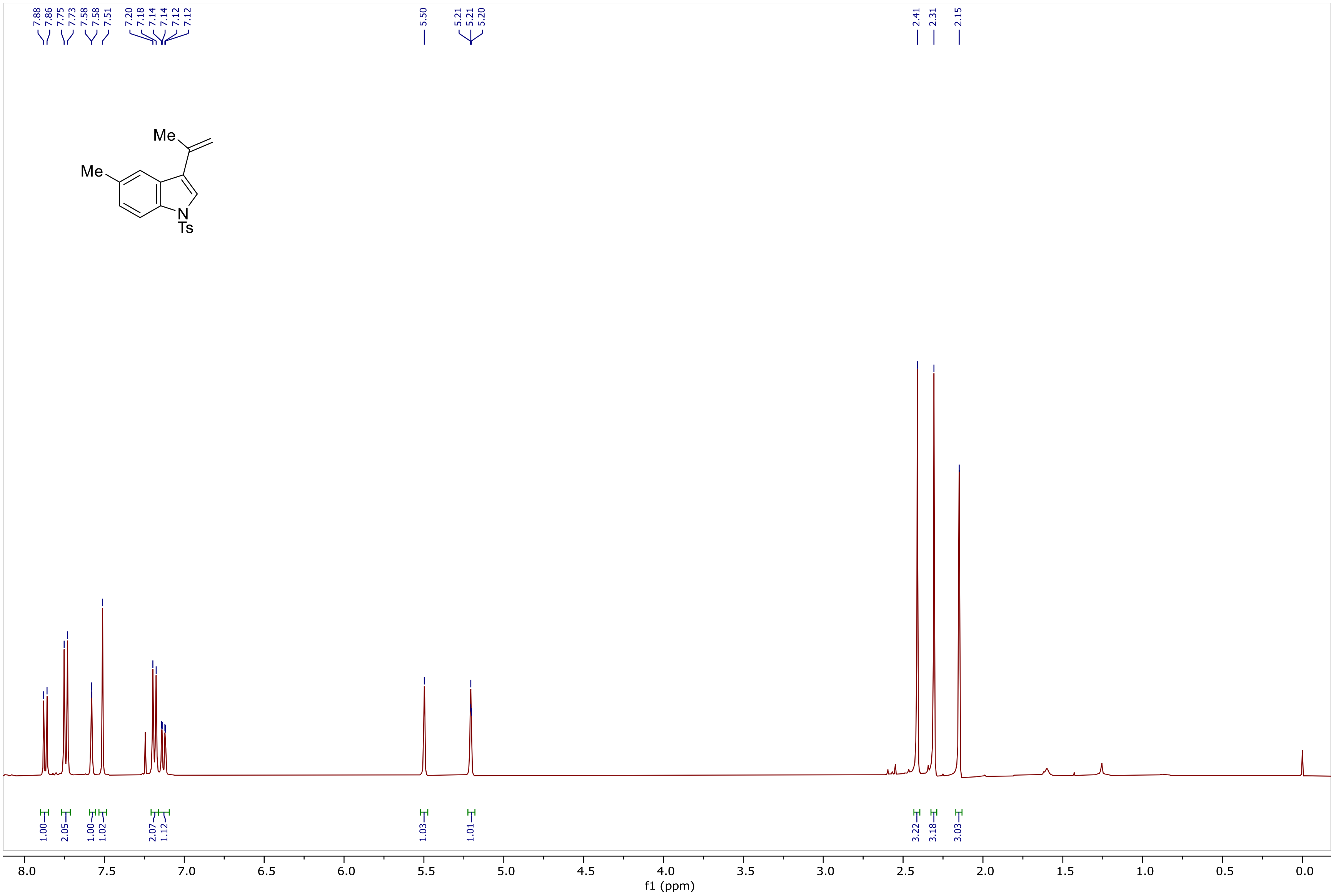

^{1}H NMR (400 MHz, $CDCl_3$) of compound **5c**

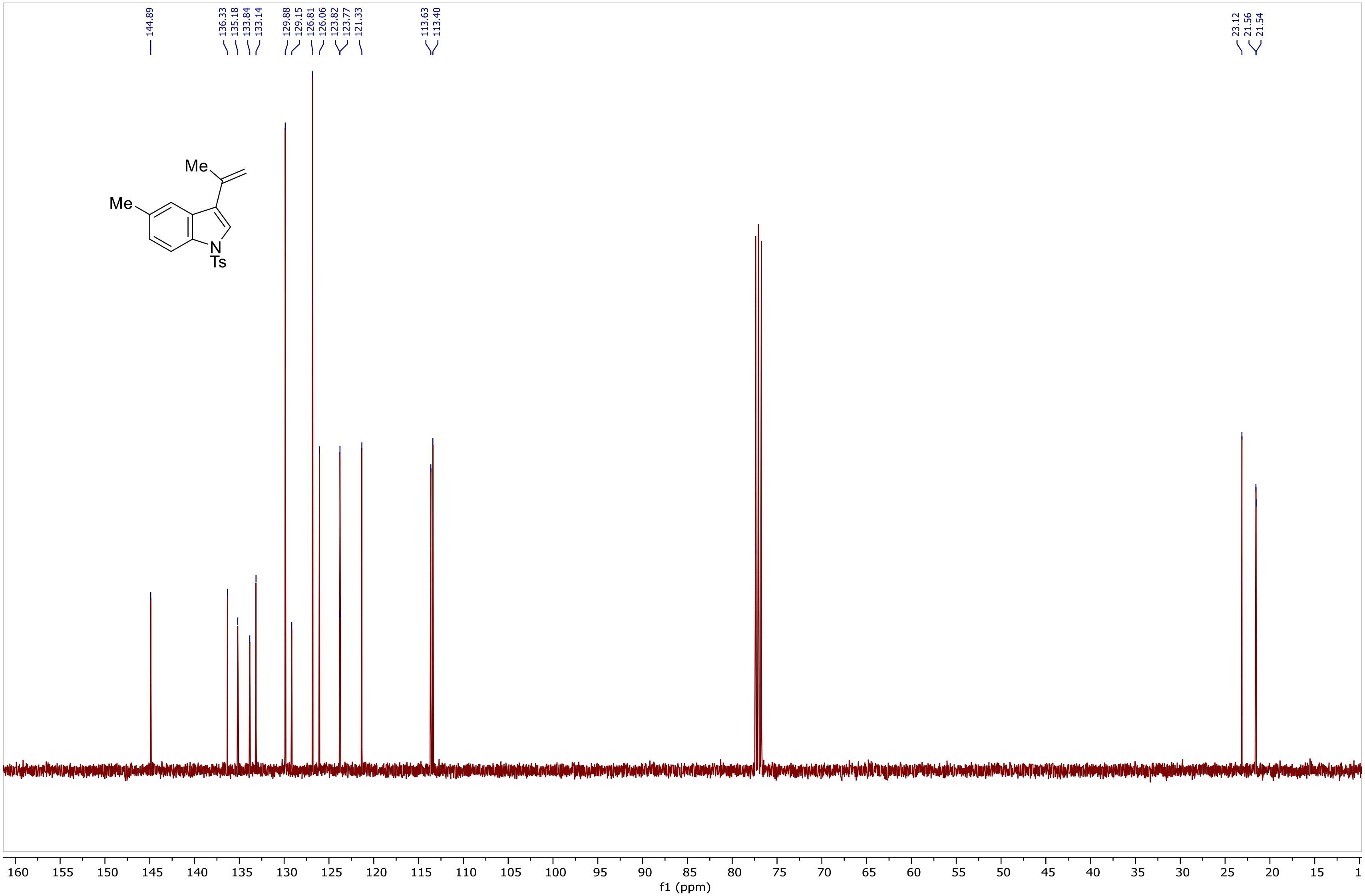

^{13}C NMR (101 MHz, $CDCl_3$) of compound **5c**

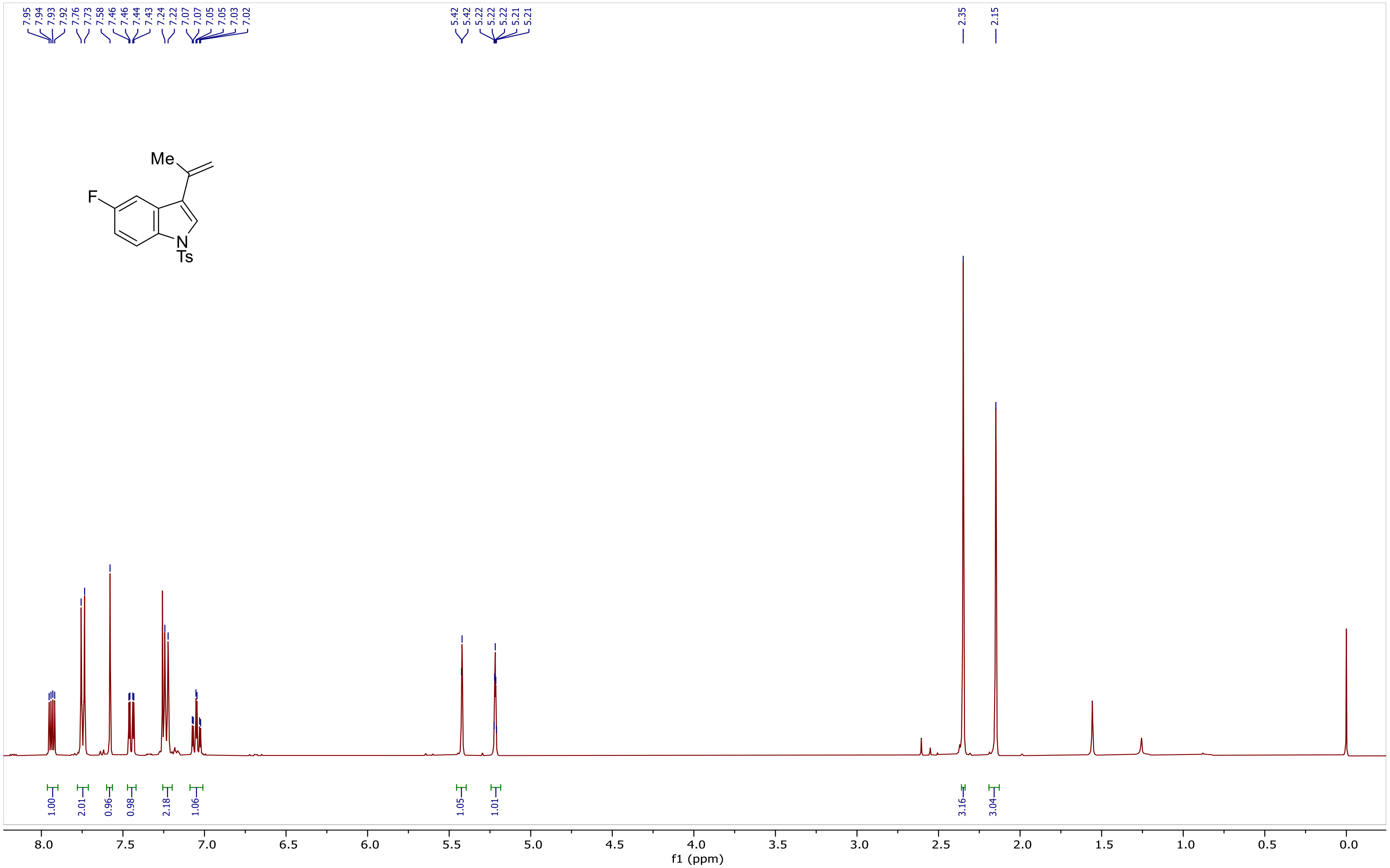

^{1}H NMR (400 MHz, $CDCl_3$) of compound **5d**

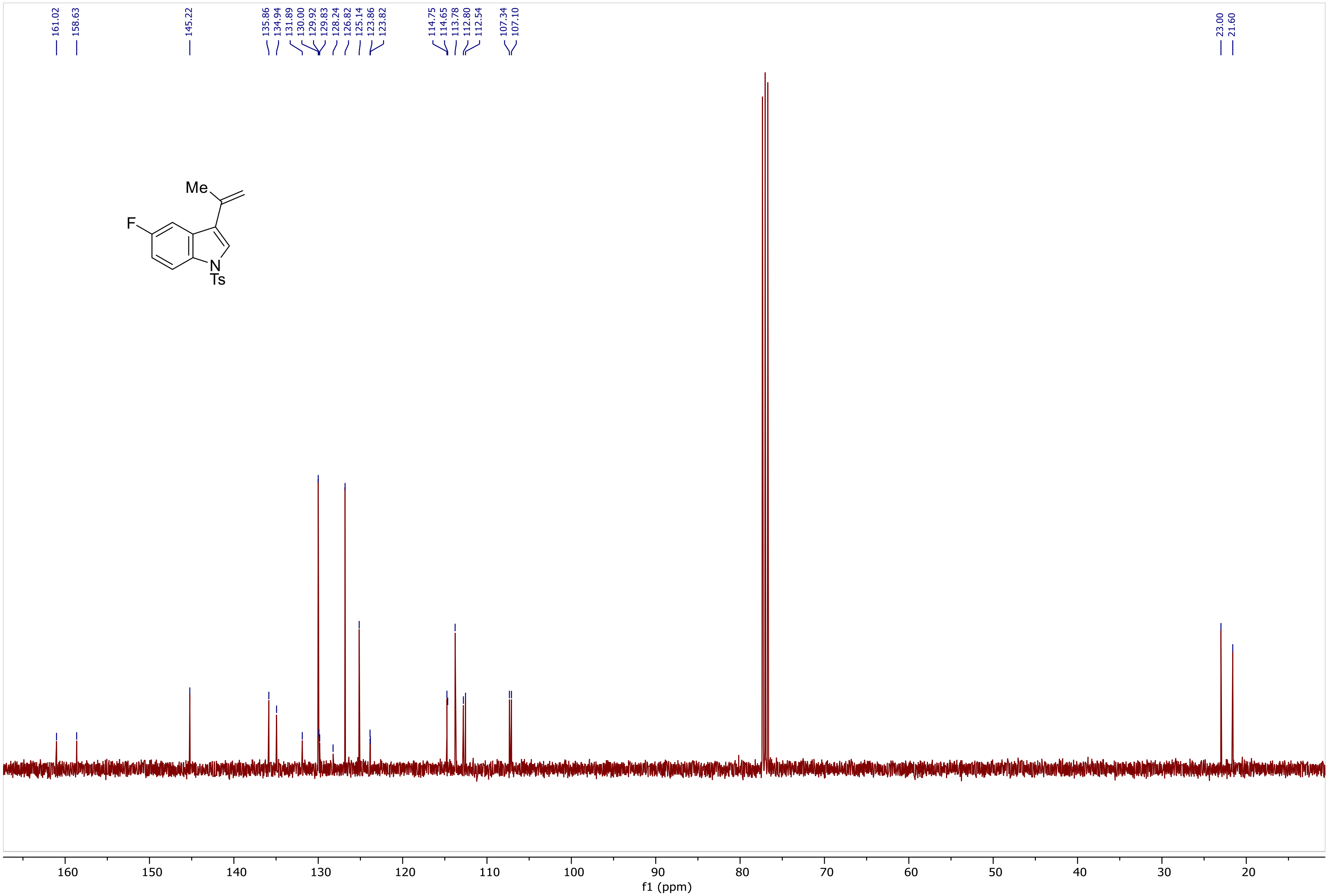

^{13}C NMR (101 MHz, $CDCl_3$) of compound **5d**

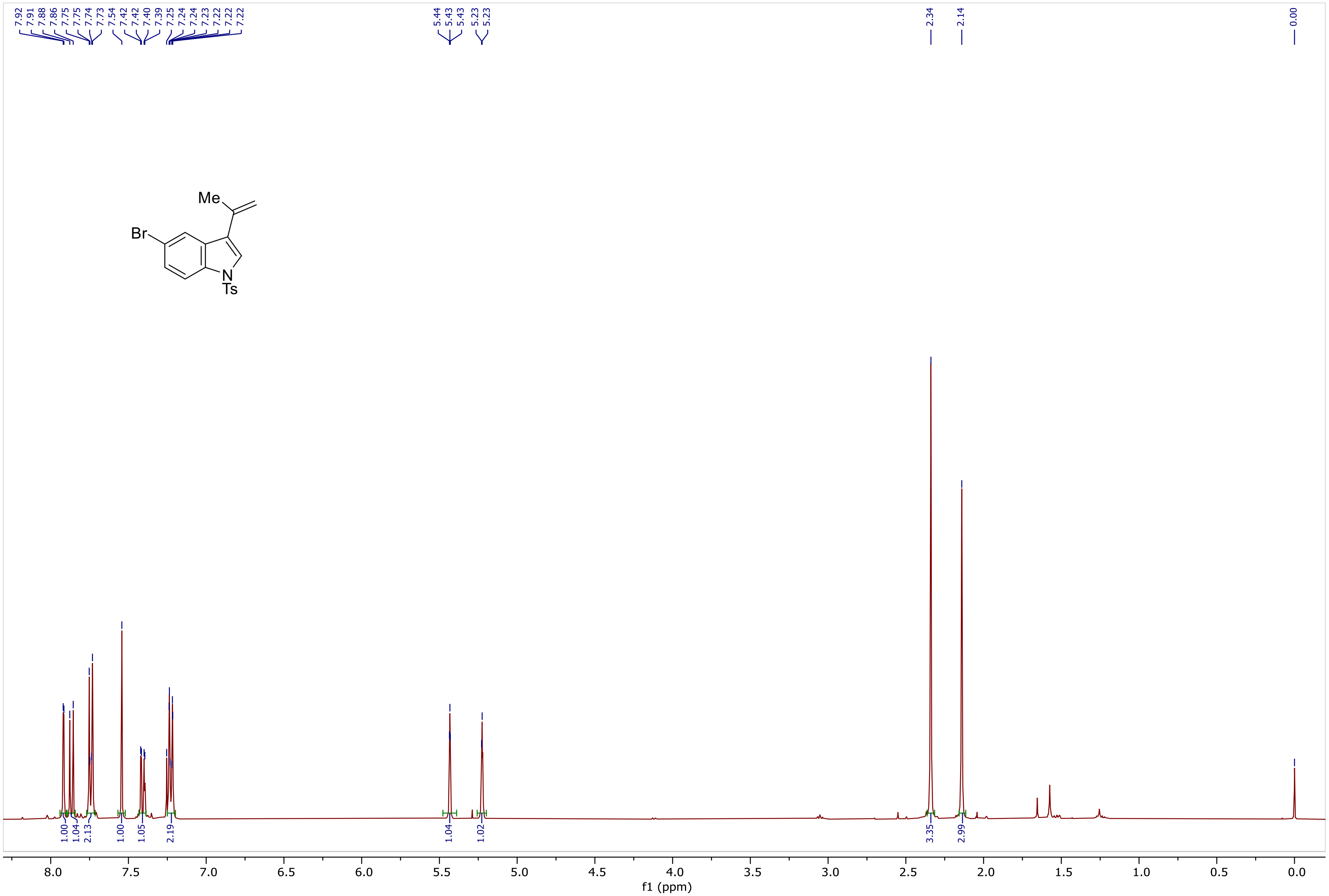

^{1}H NMR (400 MHz, $CDCl_3$) of compound **5e**

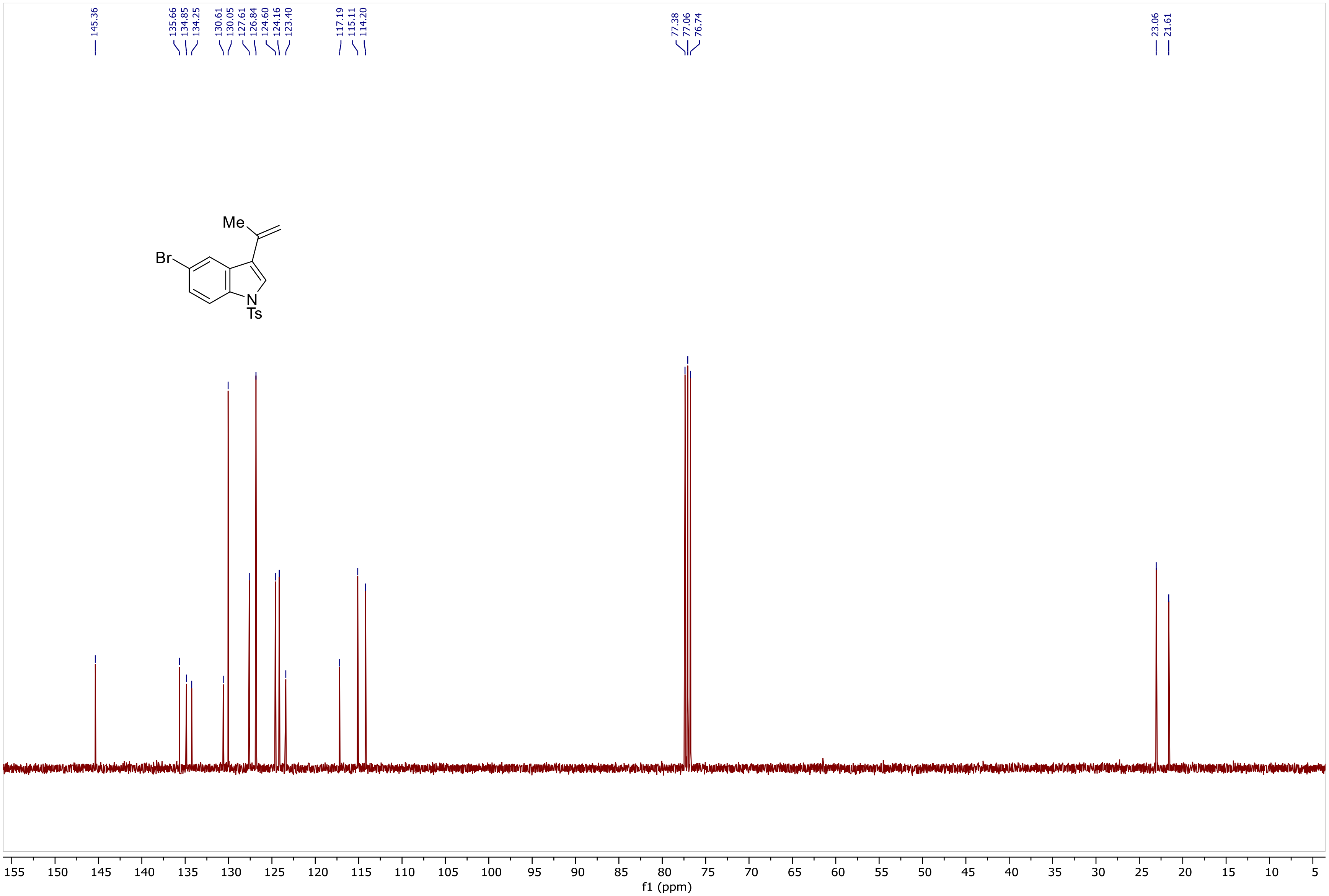

^{13}C NMR (101 MHz, $CDCl_3$) of compound **5e**

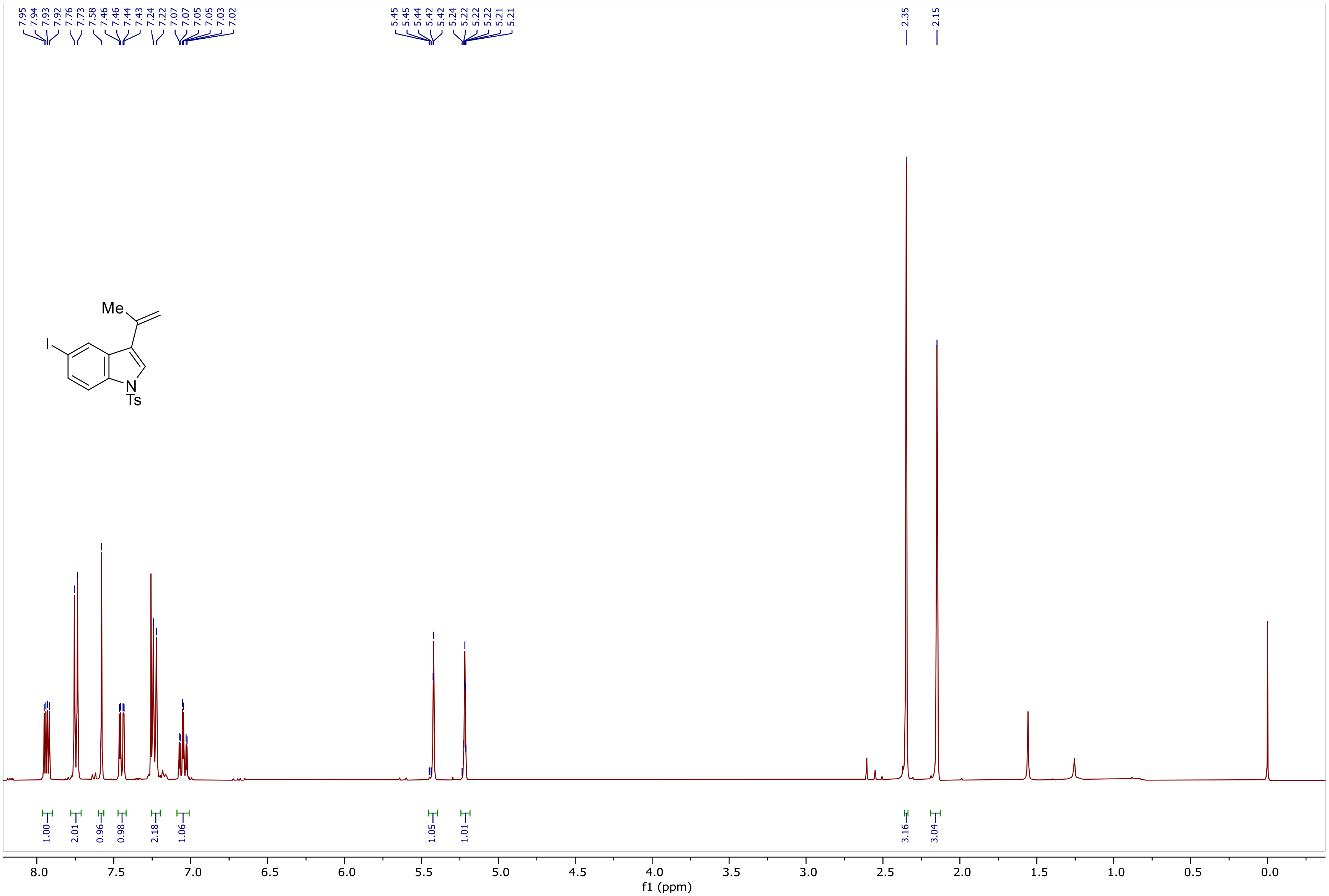

^{1}H NMR (400 MHz, $CDCl_3$) of compound **5f**

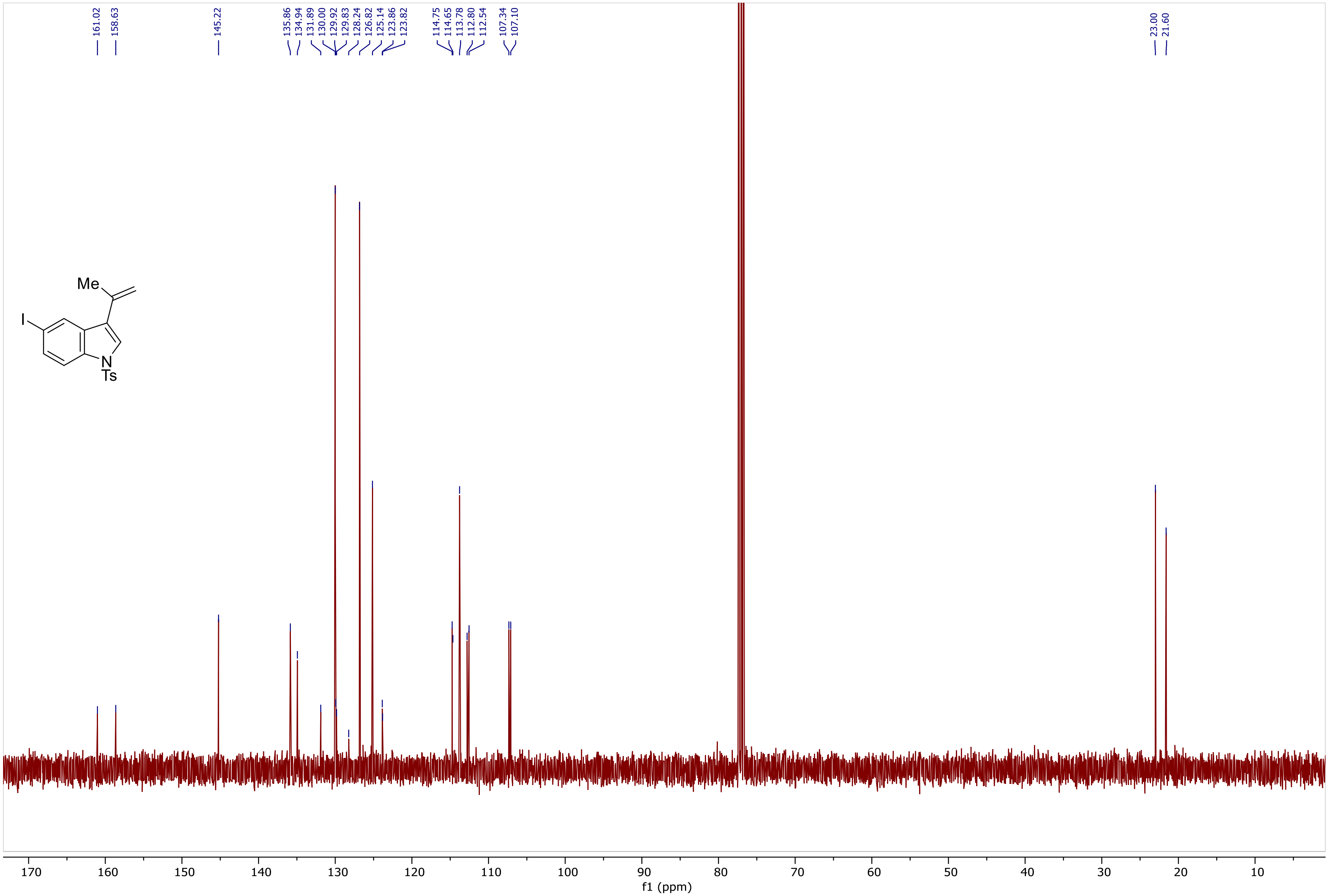

^{13}C NMR (101 MHz, $CDCl_3$) of compound **5f**

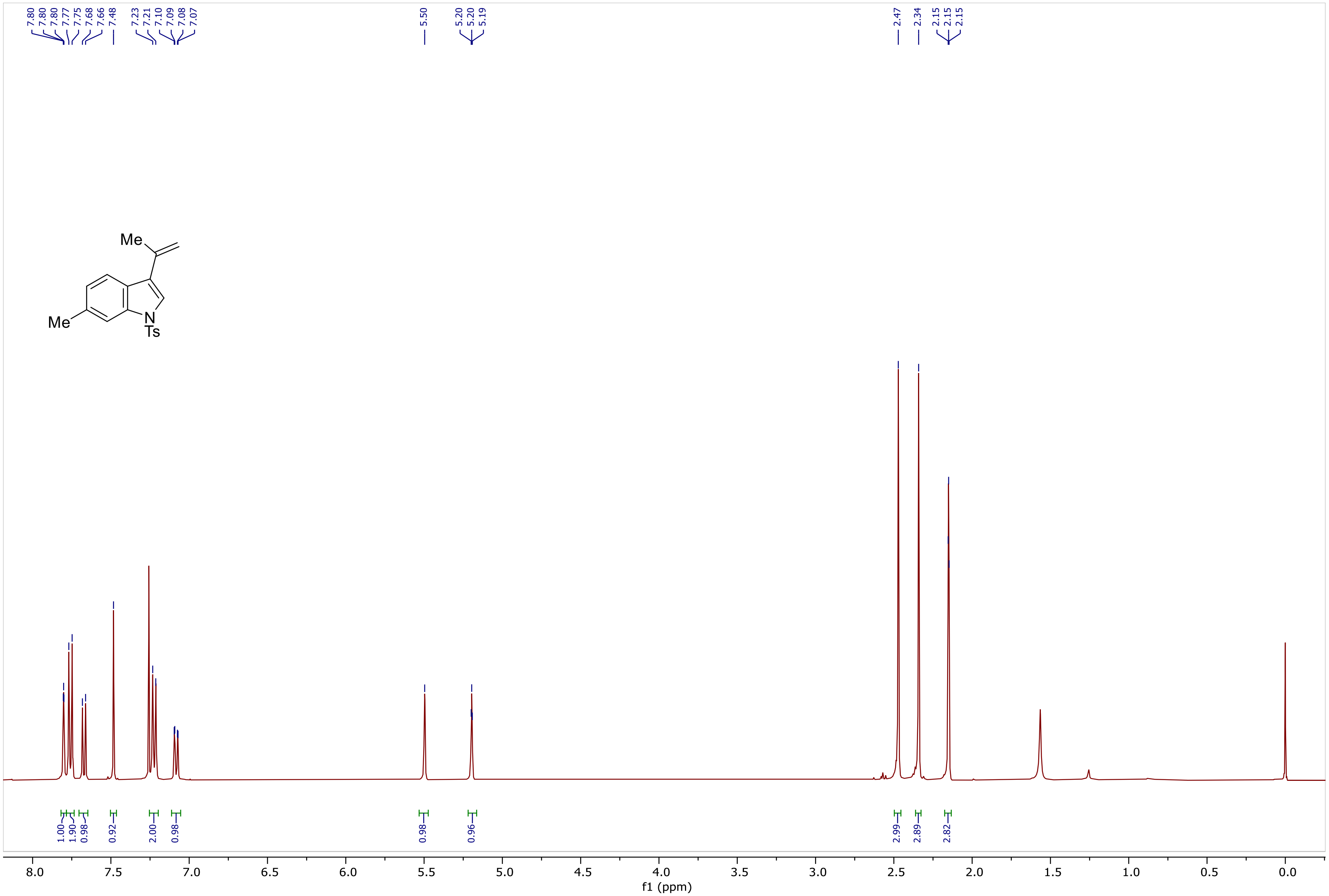

^{1}H NMR (400 MHz, $CDCl_3$) of compound **5g**

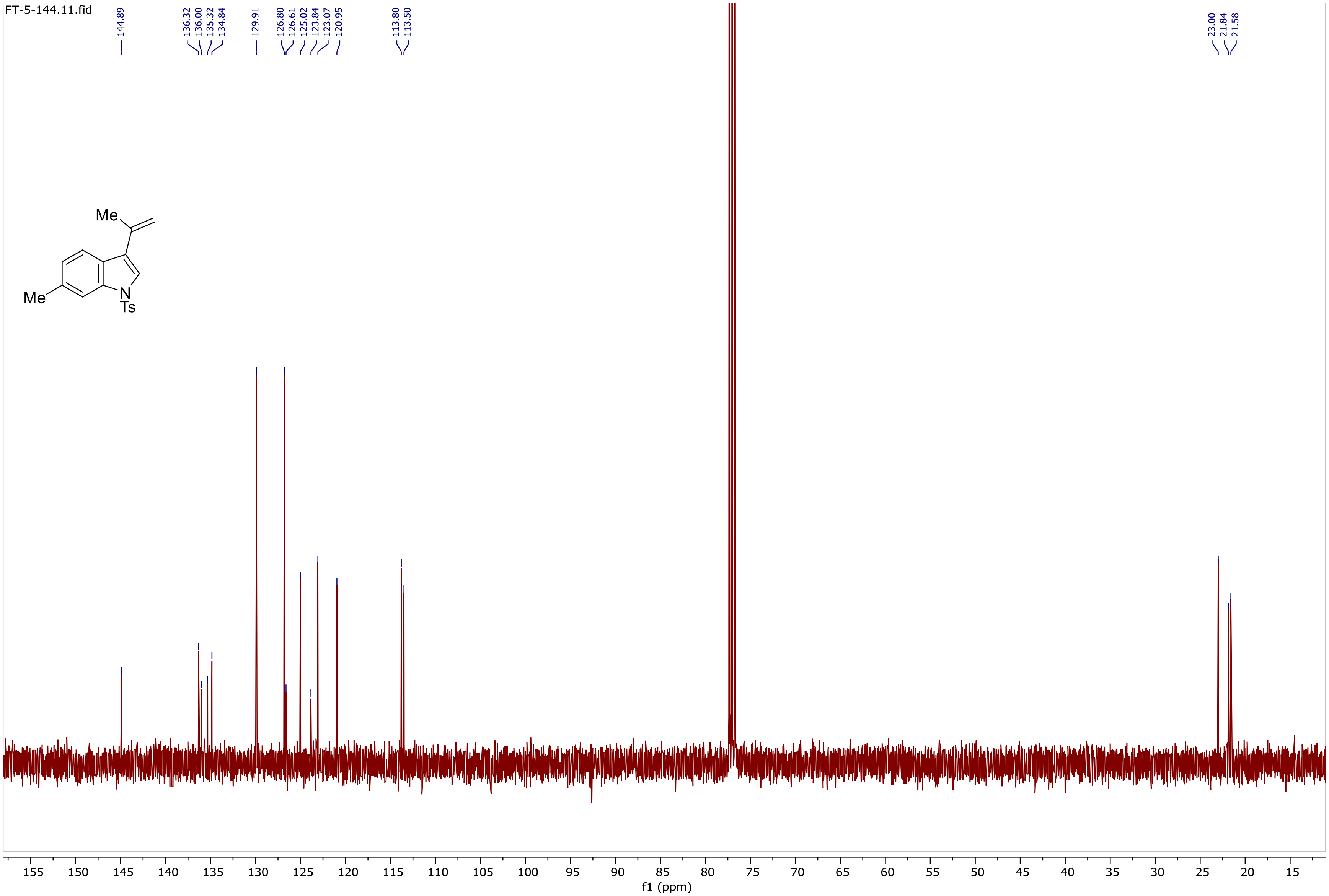

^{13}C NMR (101 MHz, $CDCl_3$) of compound **5g**

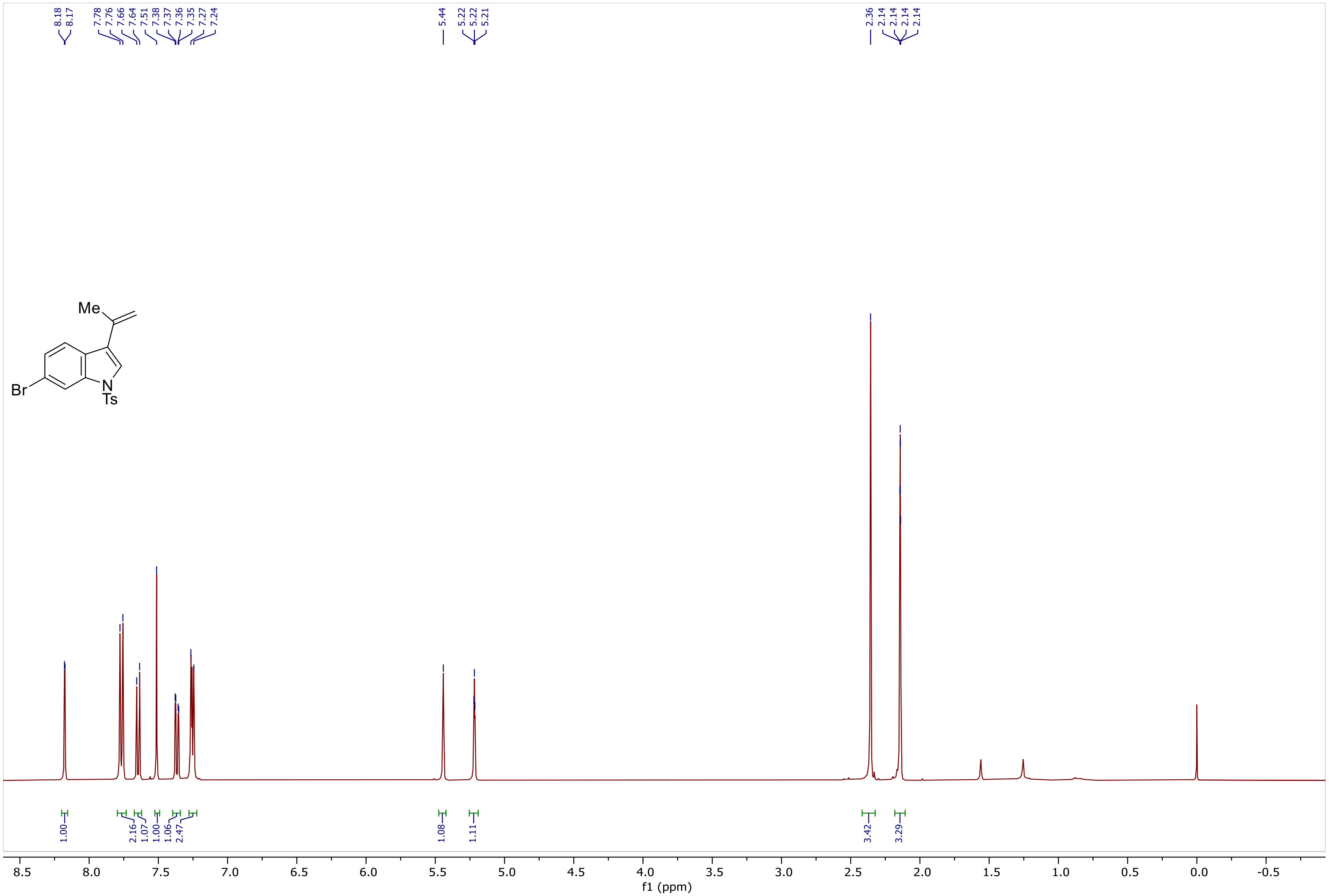

^{1}H NMR (400 MHz, $CDCl_3$) of compound **5h**

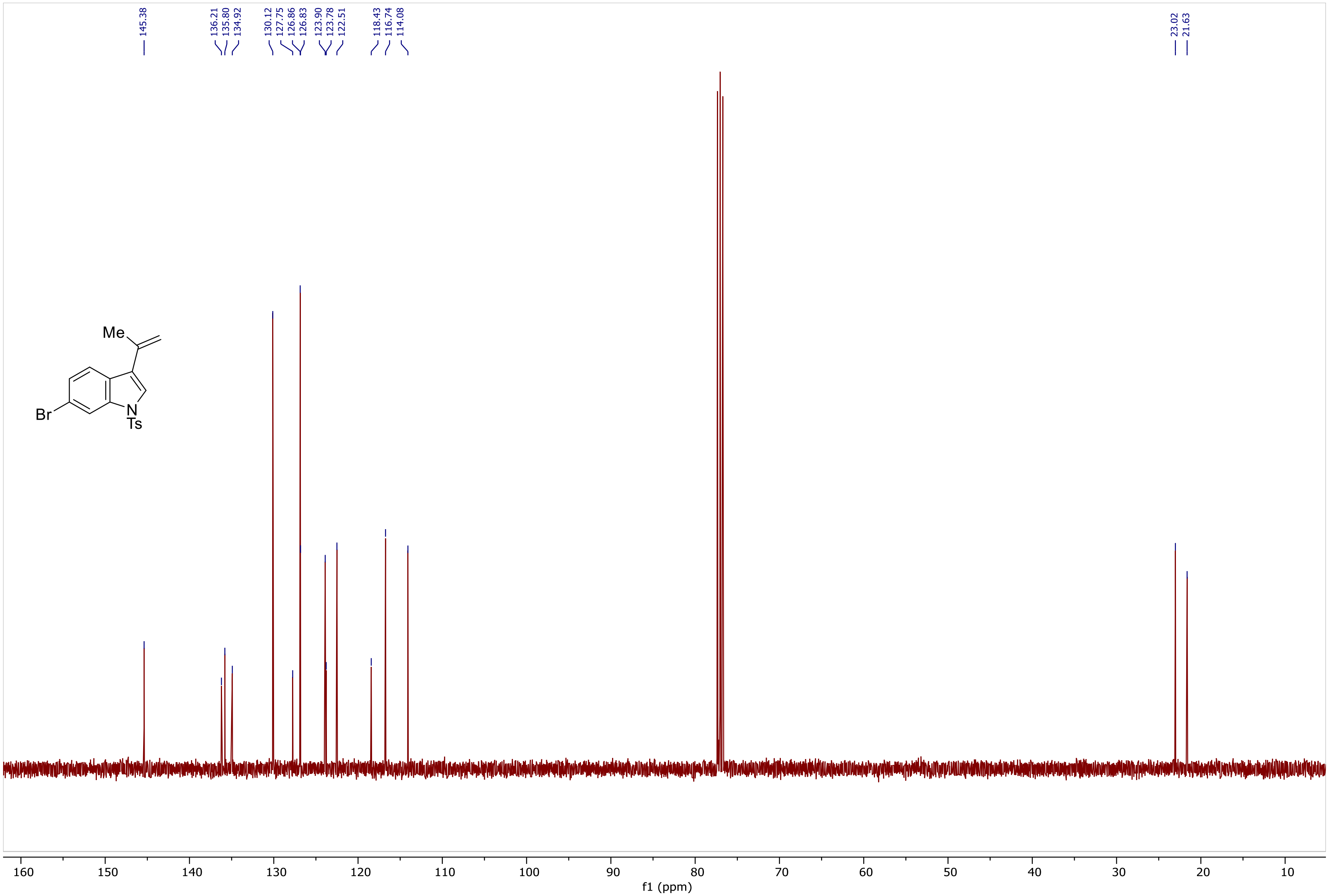

^{13}C NMR (101 MHz, $CDCl_3$) of compound **5h**

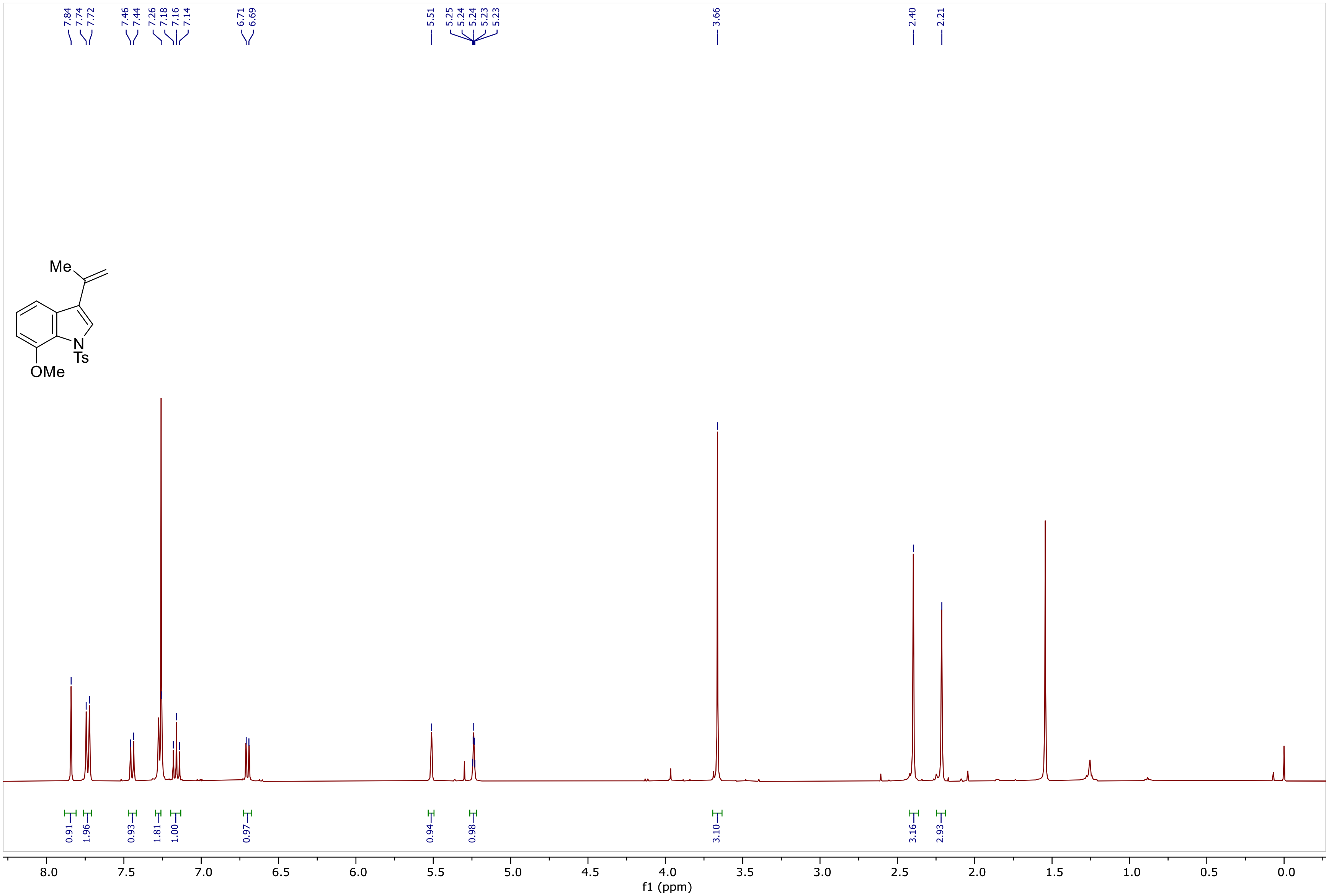

^{1}H NMR (400 MHz, $CDCl_3$) of compound **5i**

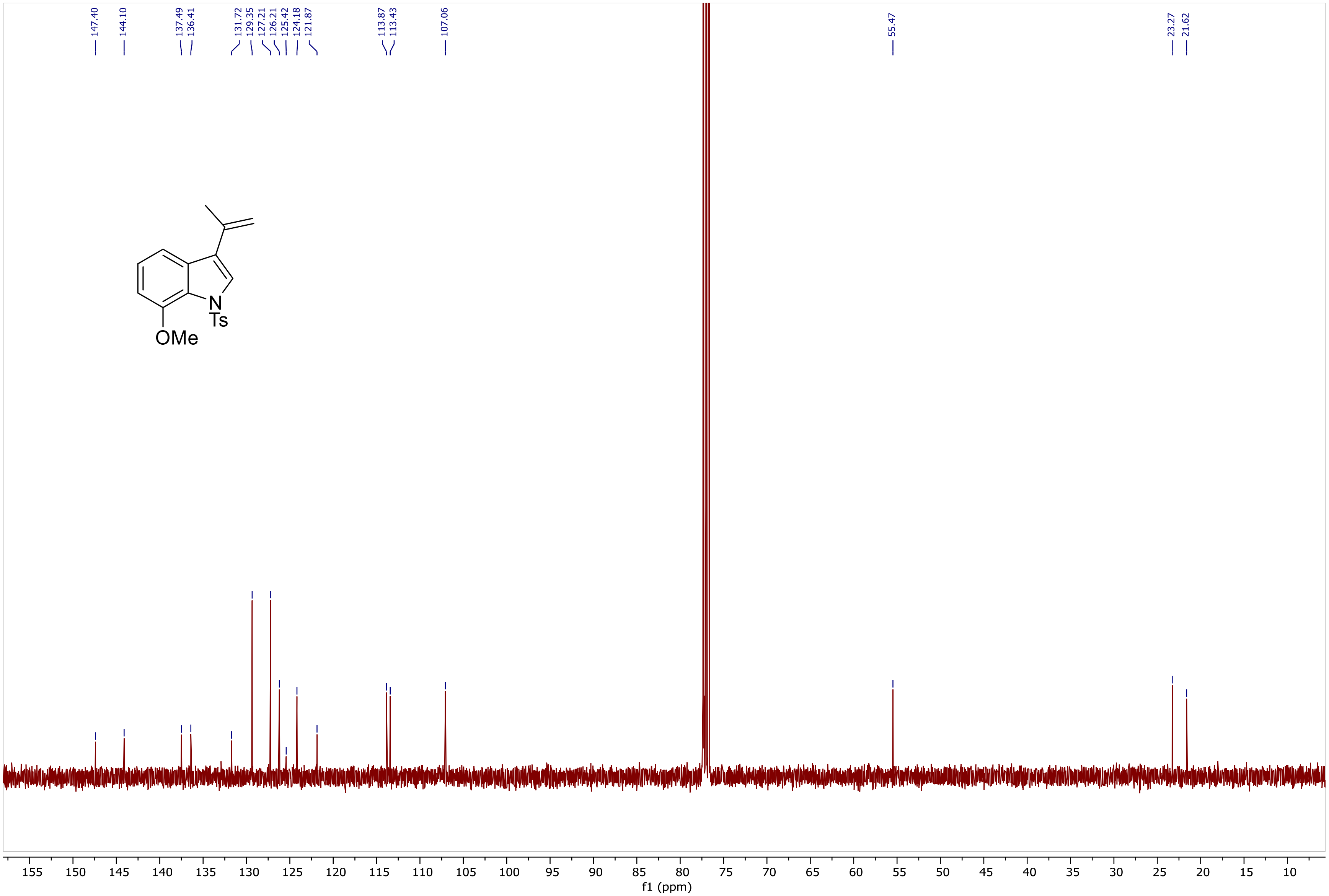

^{13}C NMR (101 MHz, $CDCl_3$) of compound **5i**

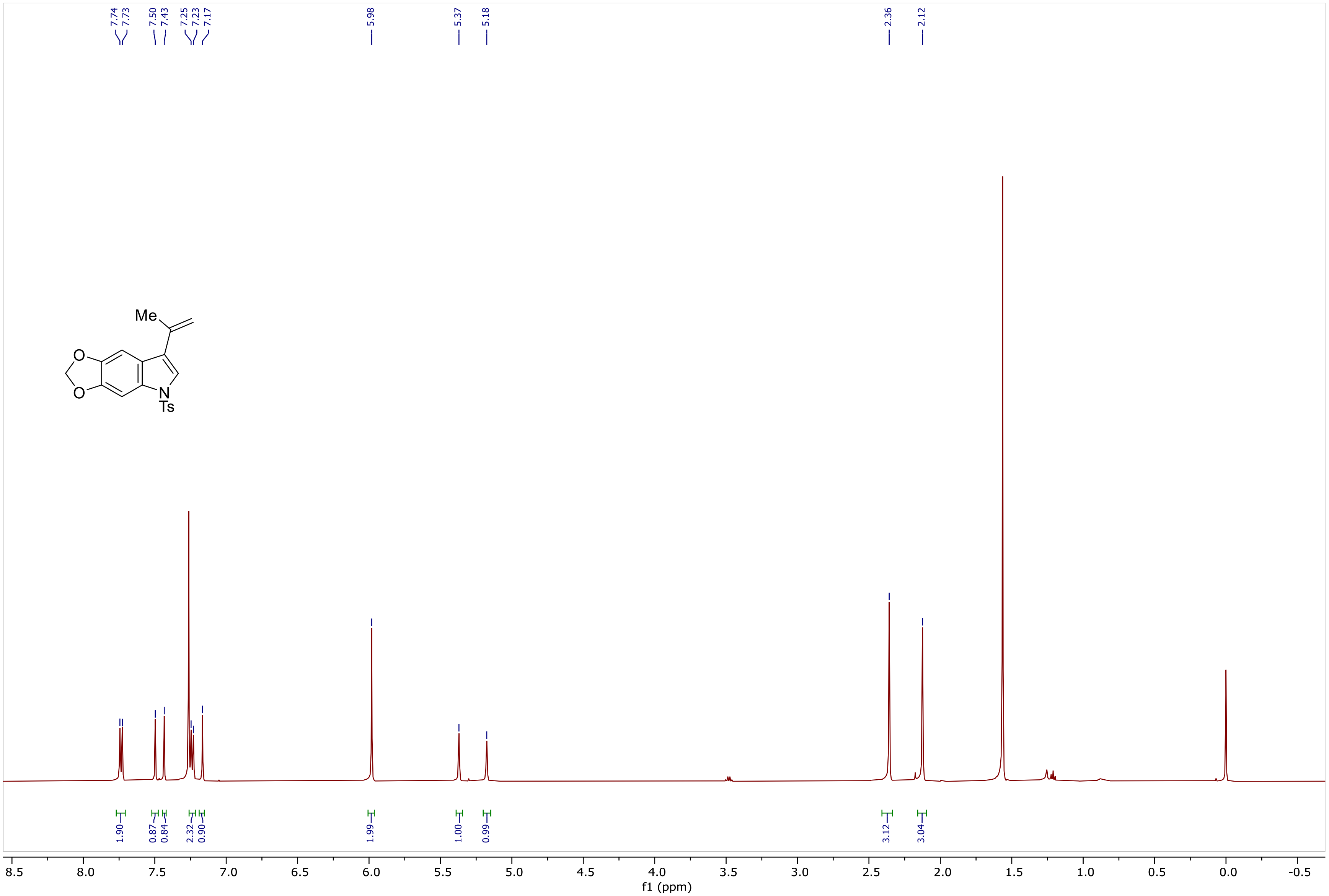

^{1}H NMR (400 MHz, $CDCl_3$) of compound **5j**

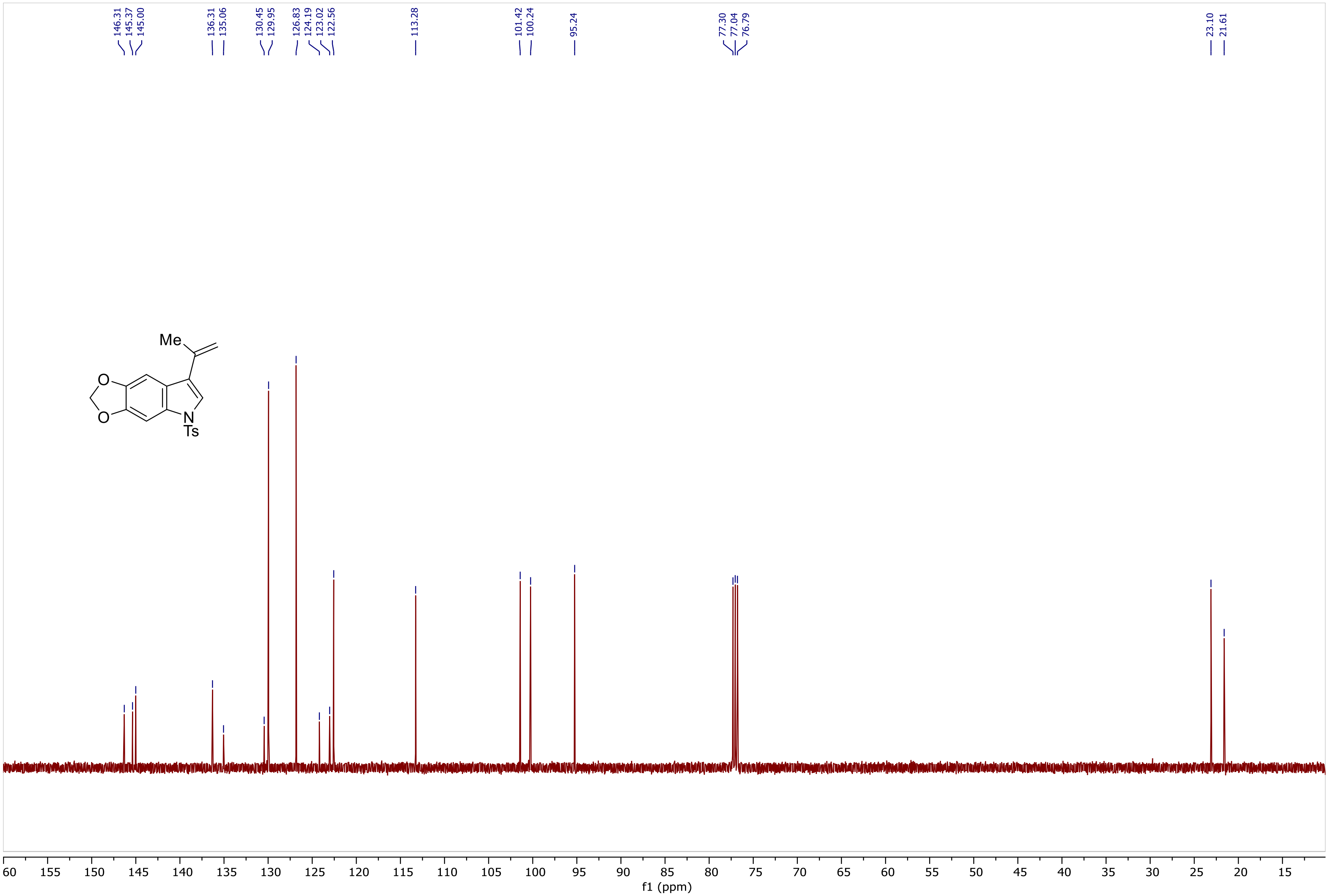

^{13}C NMR (101 MHz, $CDCl_3$) of compound **5j**

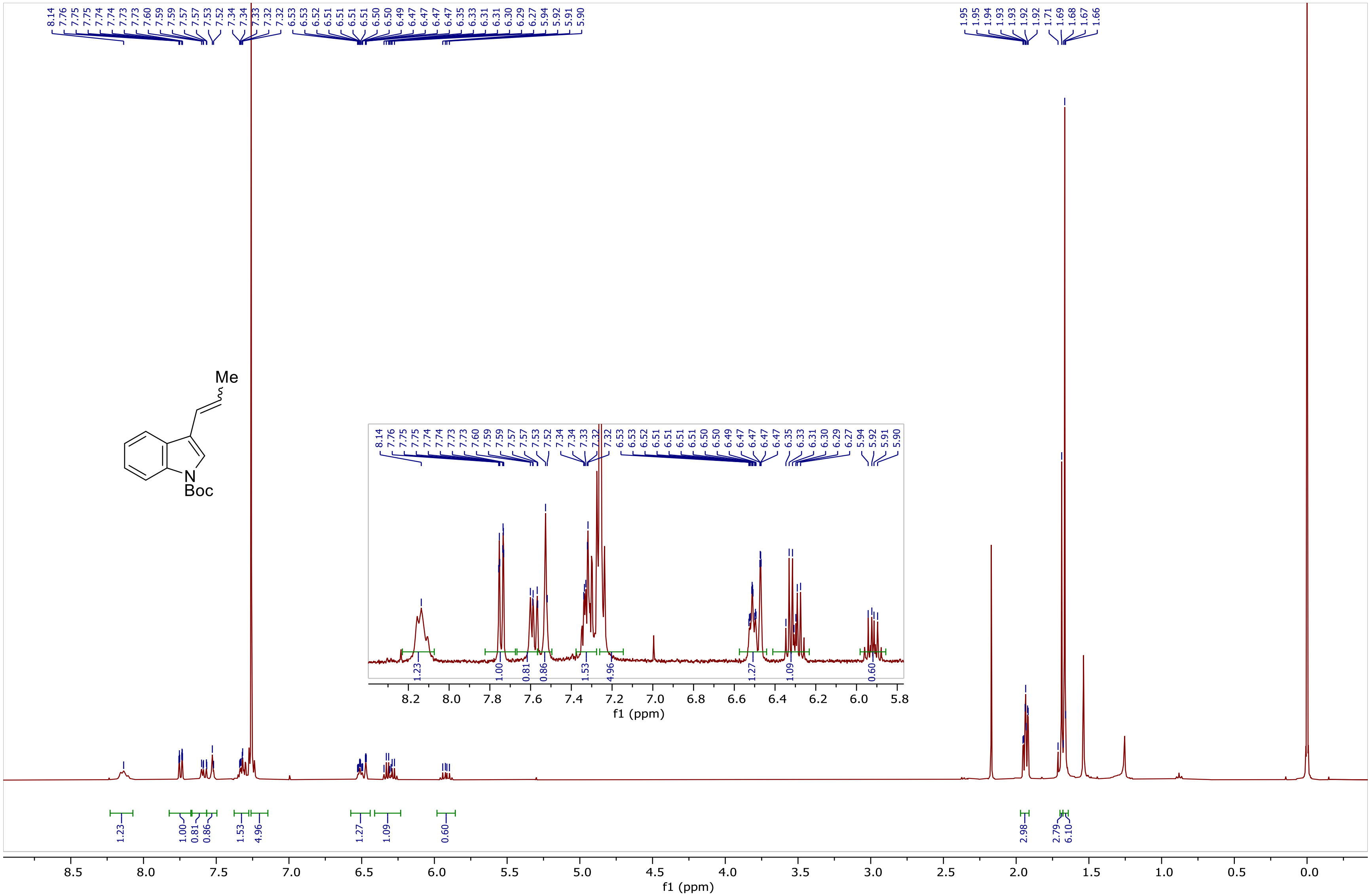

^{1}H NMR (400 MHz, $CDCl_3$) of compound **5k**

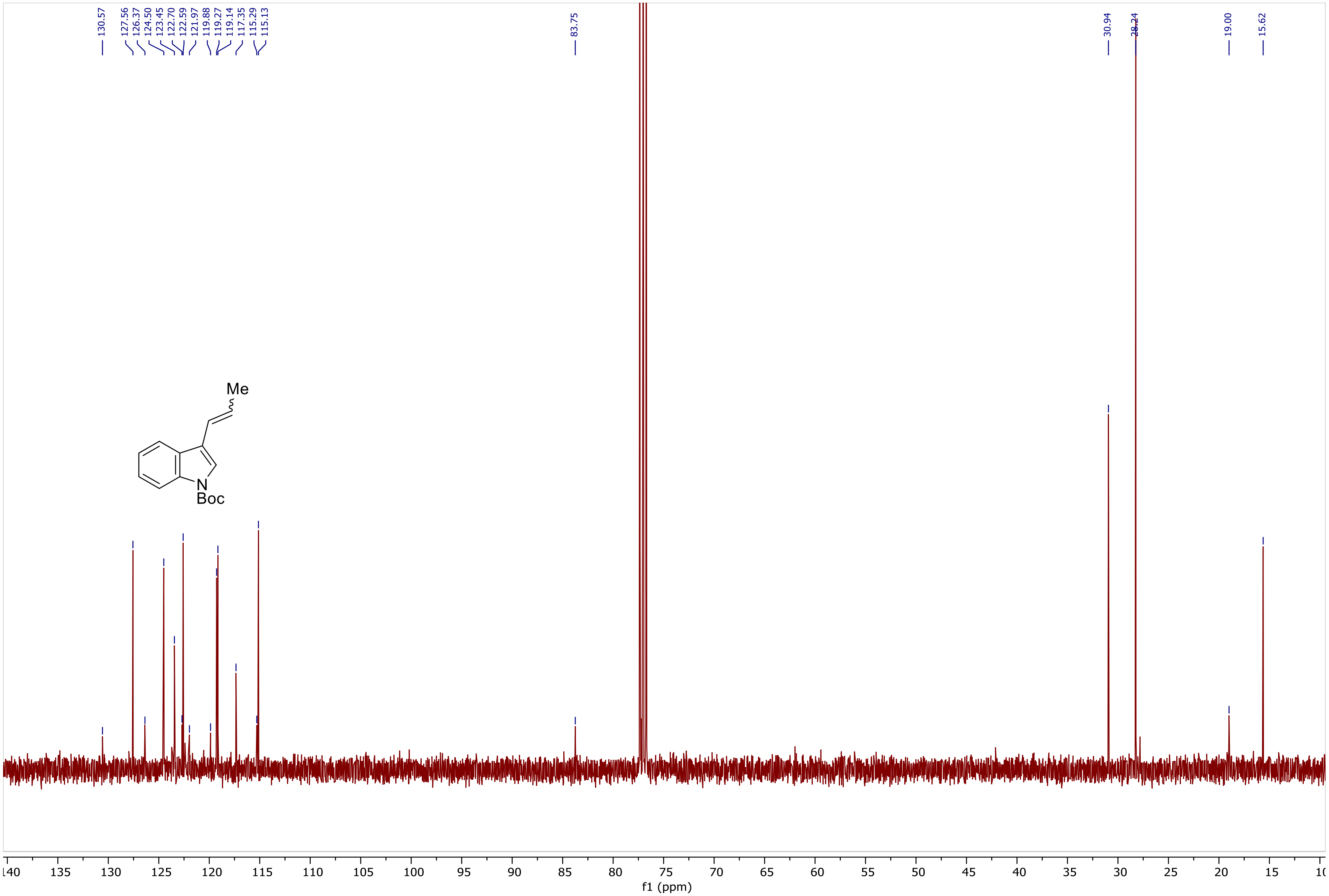

^{13}C NMR (101 MHz, $CDCl_3$) of compound **5k**

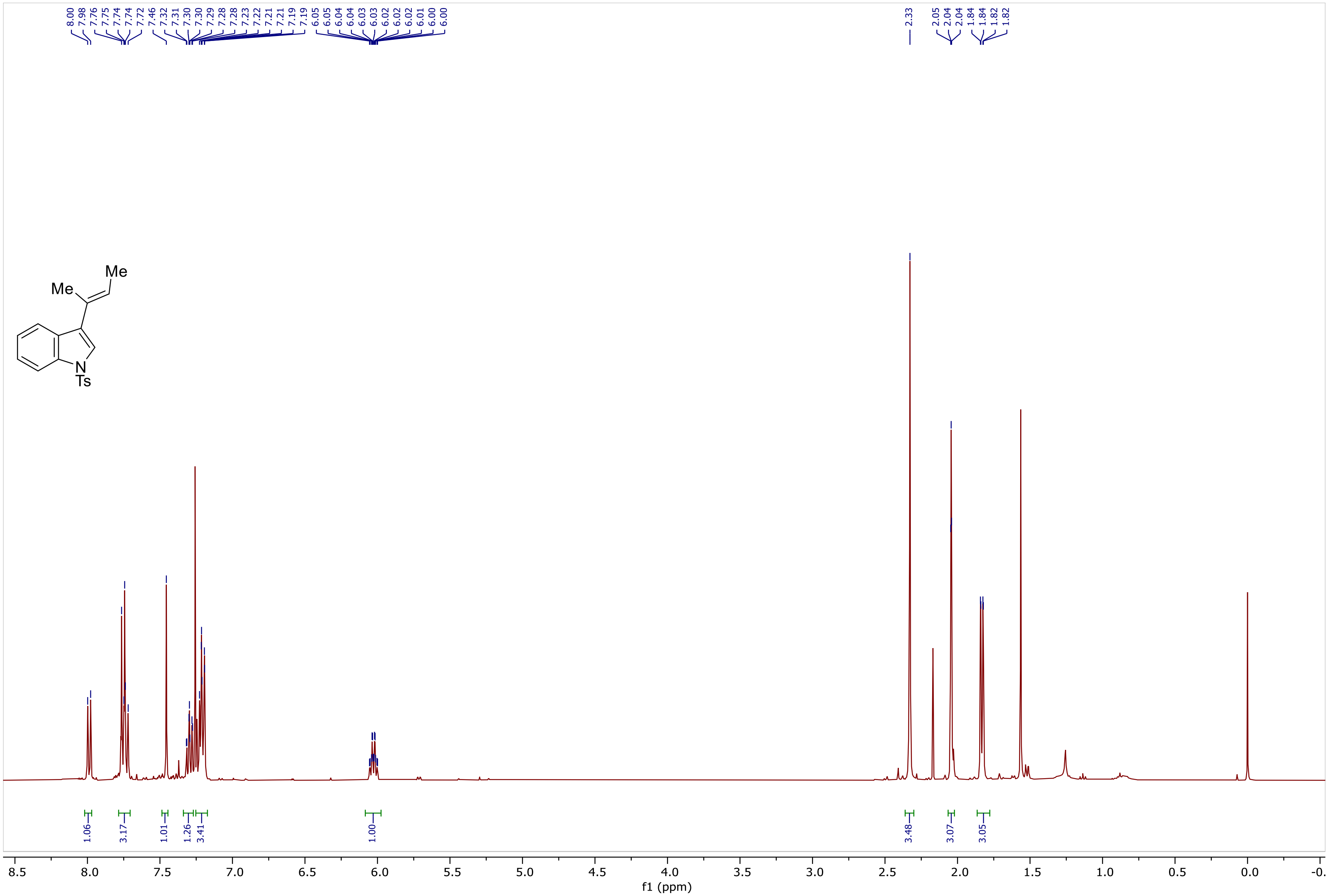

^{1}H NMR (400 MHz, $CDCl_3$) of compound **5l**

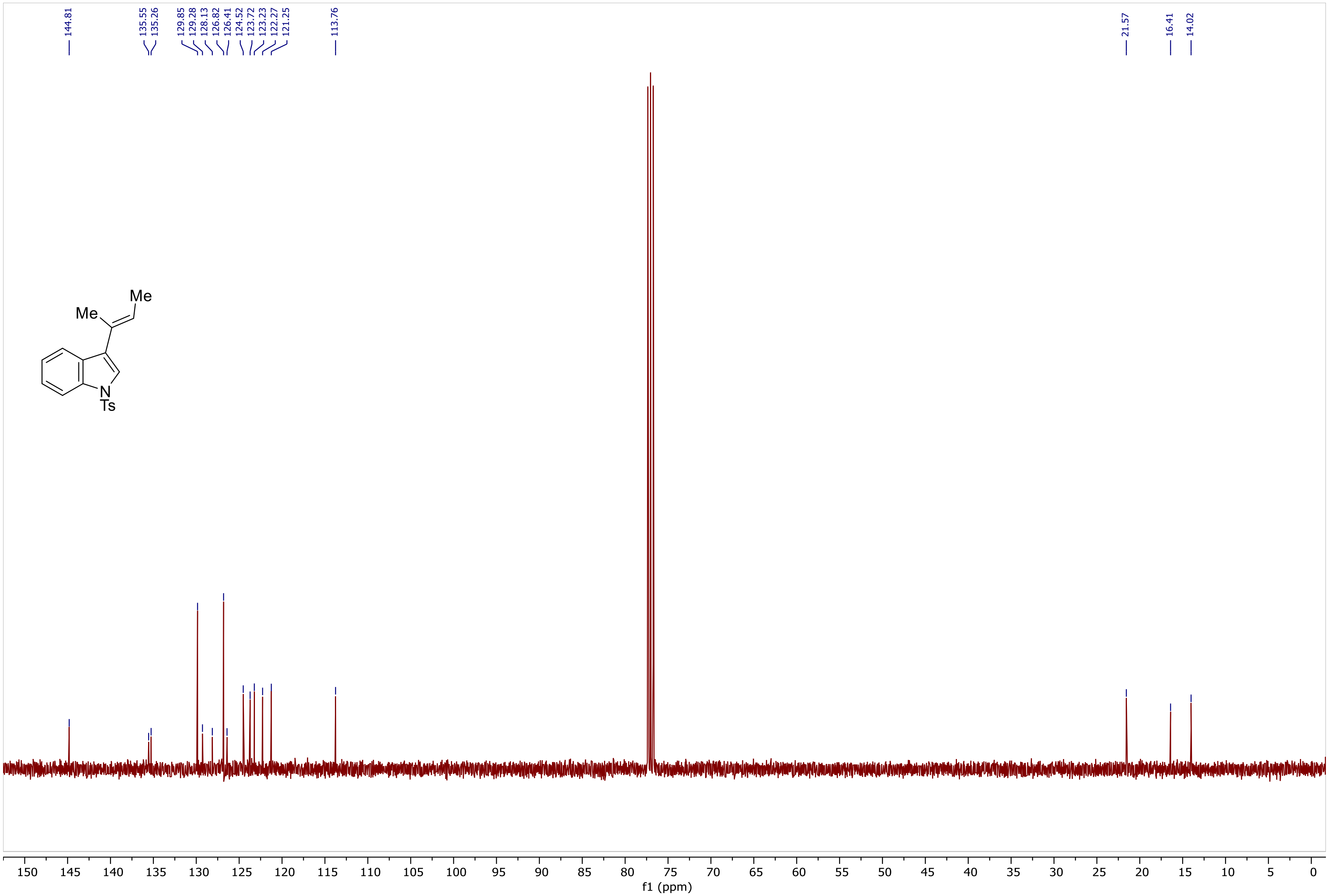

^{13}C NMR (101 MHz, $CDCl_3$) of compound **5l**

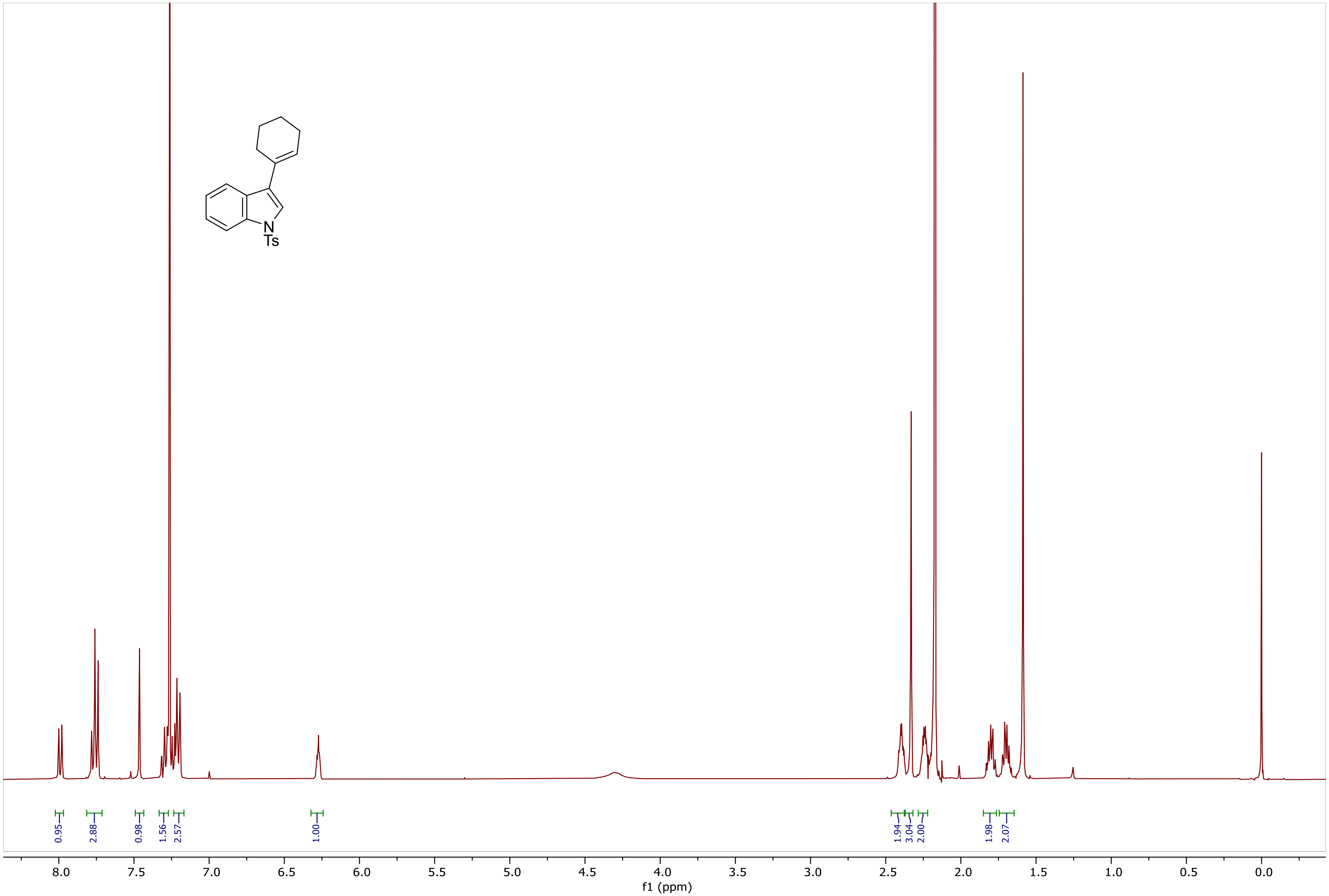

^{1}H NMR (400 MHz, $CDCl_3$) of compound **5m**

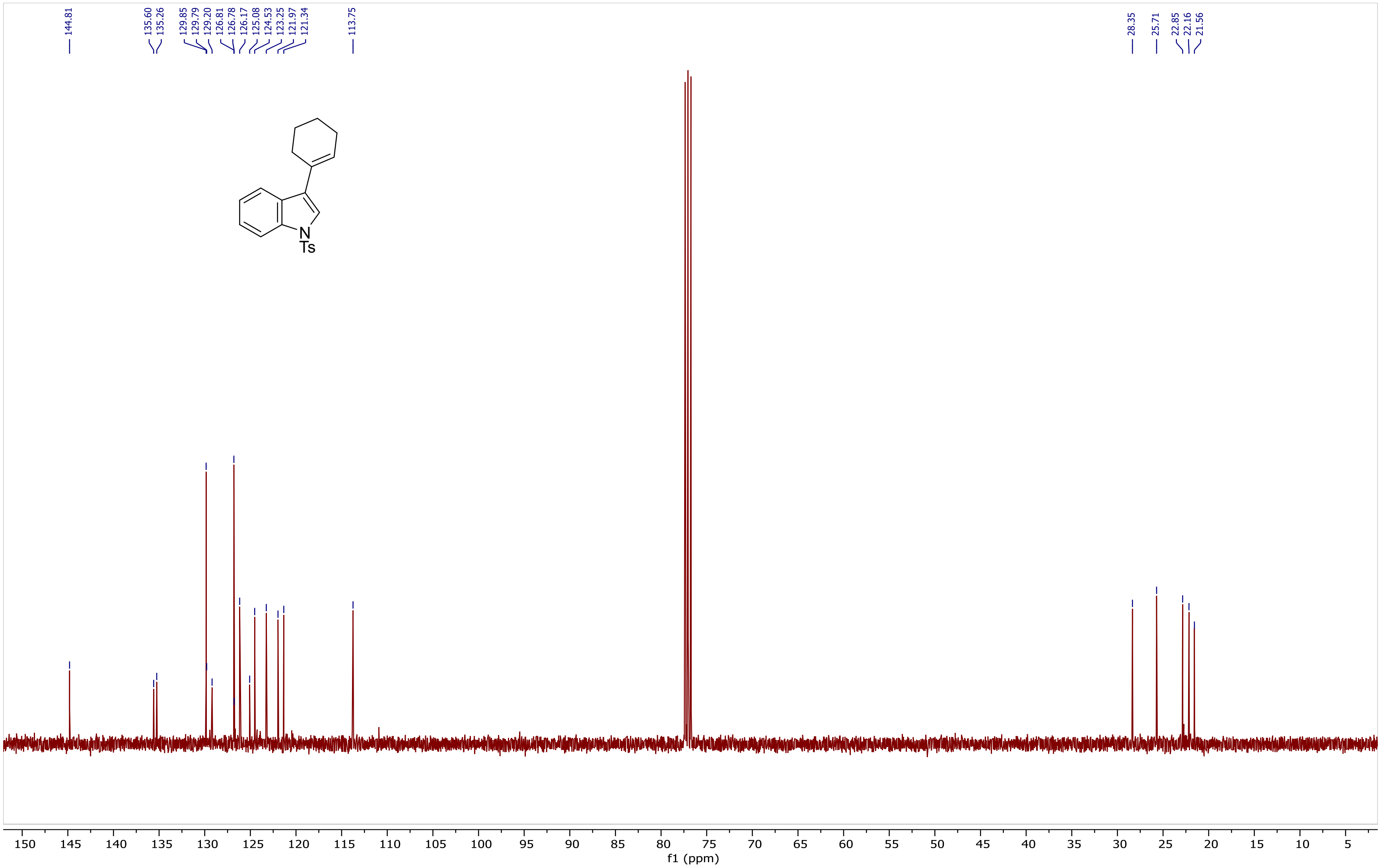

^{13}C NMR (101 MHz, $CDCl_3$) of compound **5m**

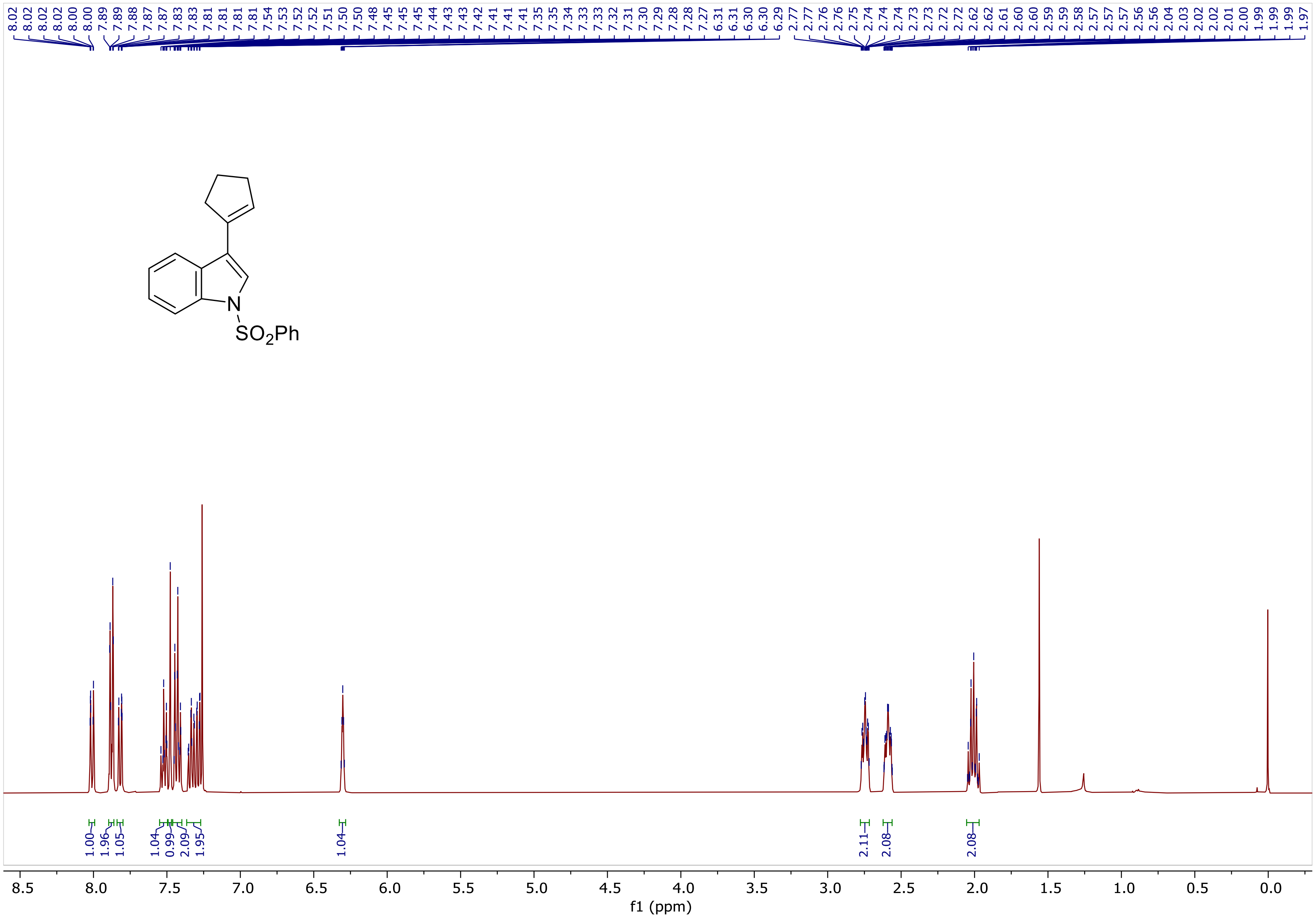

^{1}H NMR (400 MHz, $CDCl_3$) of compound **5n**

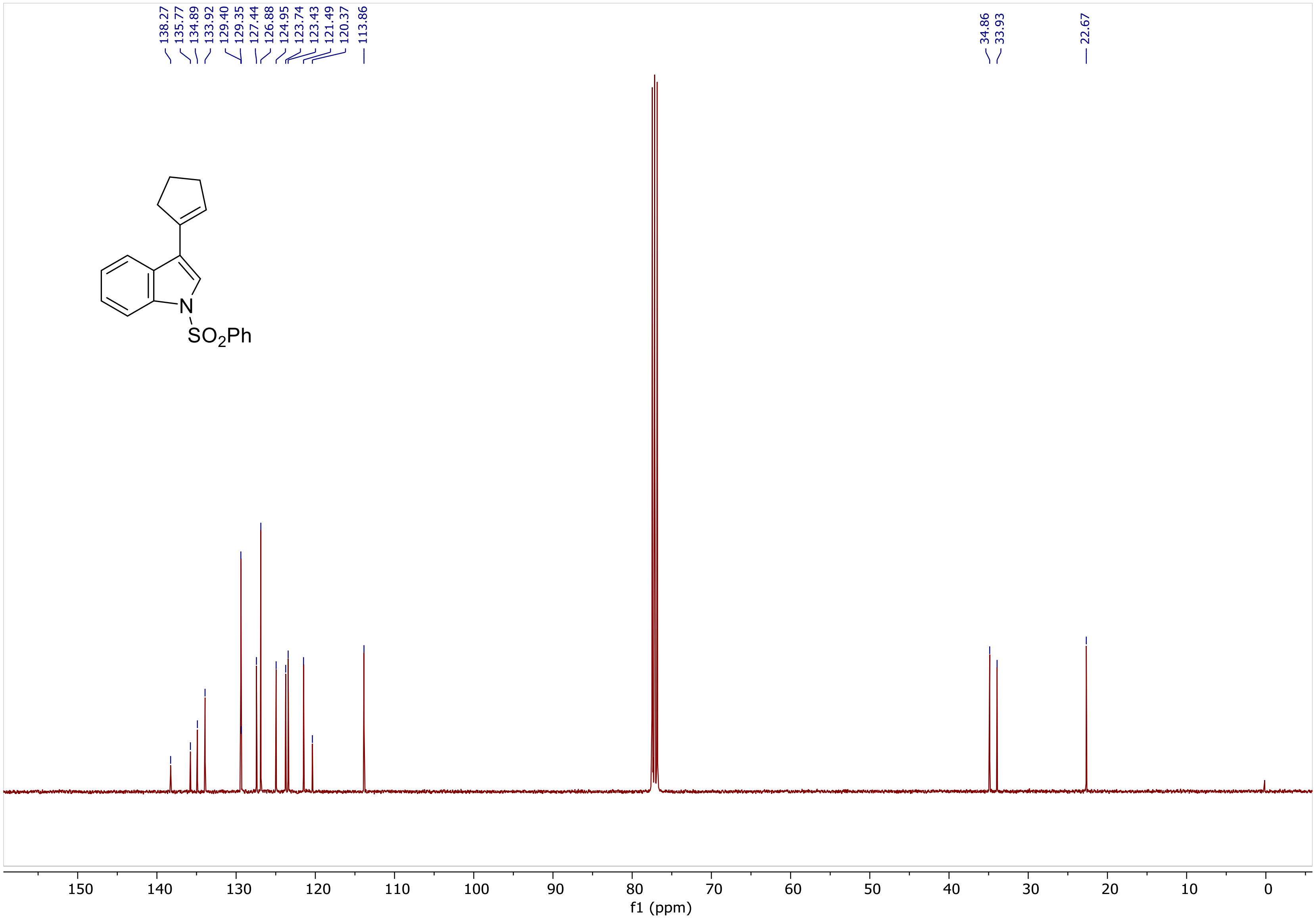

^{13}C NMR (101 MHz, $CDCl_3$) of compound **5n**

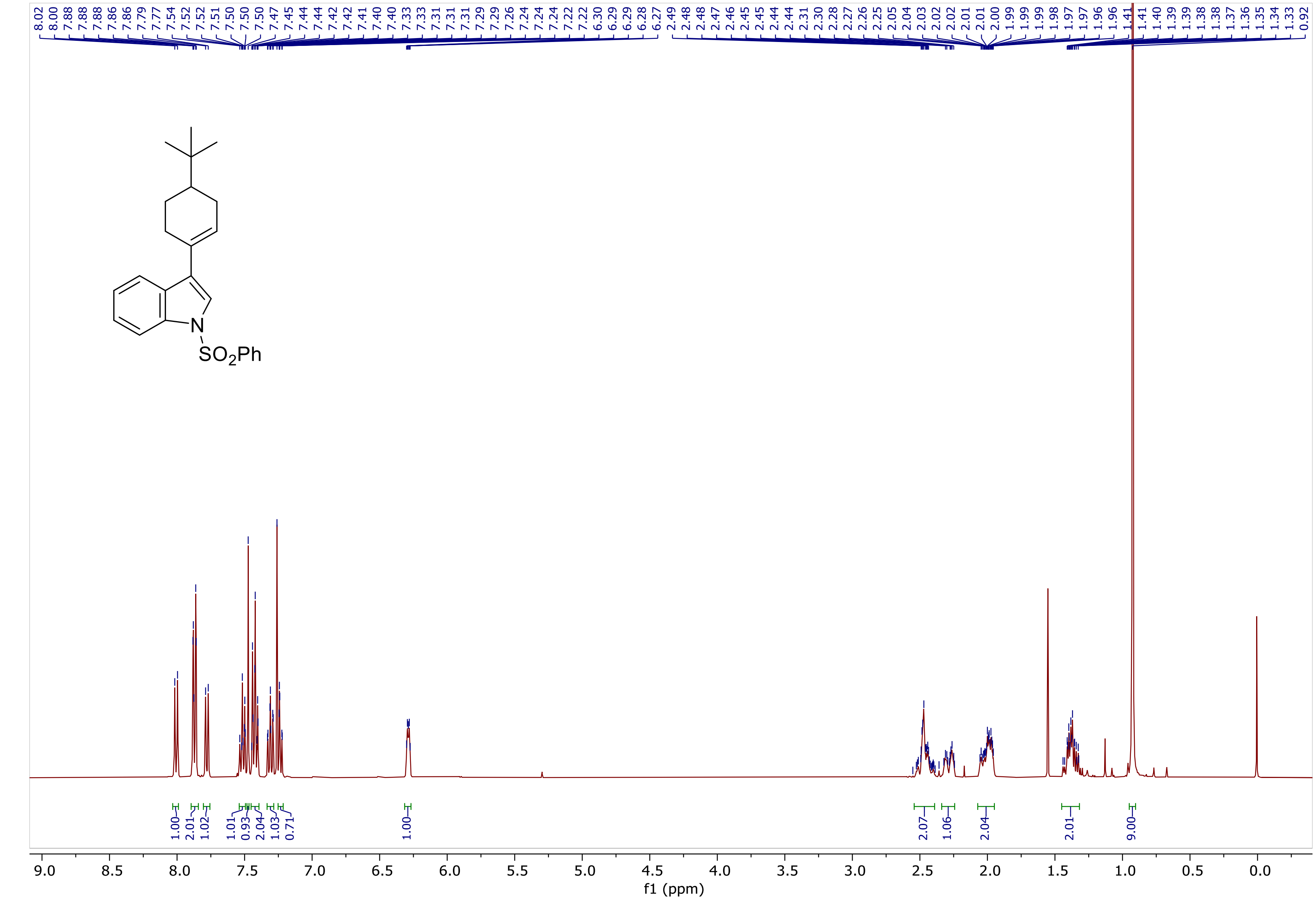

^{1}H NMR (400 MHz, $CDCl_3$) of compound **5o**

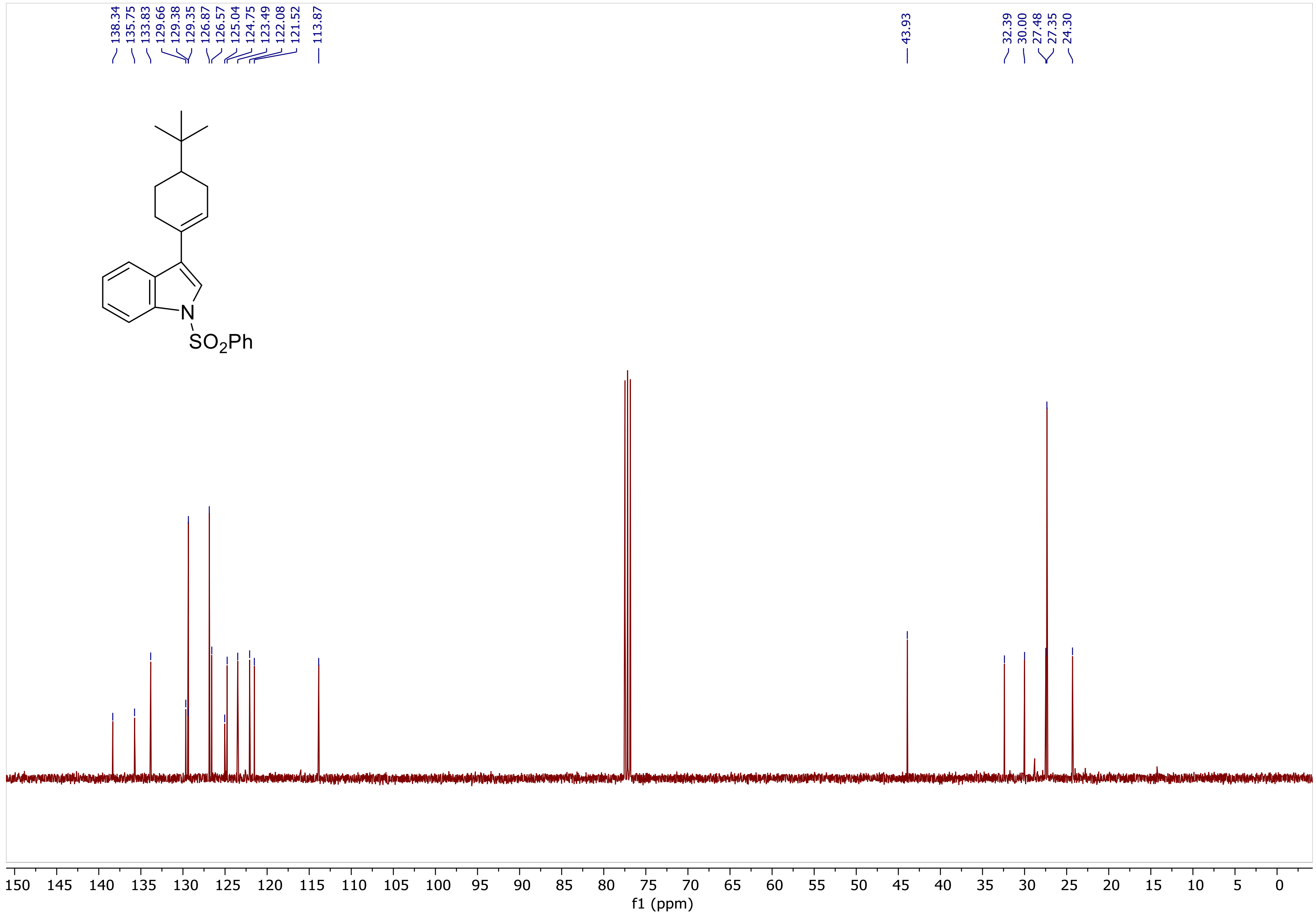

^{13}C NMR (101 MHz, $CDCl_3$) of compound **5o**

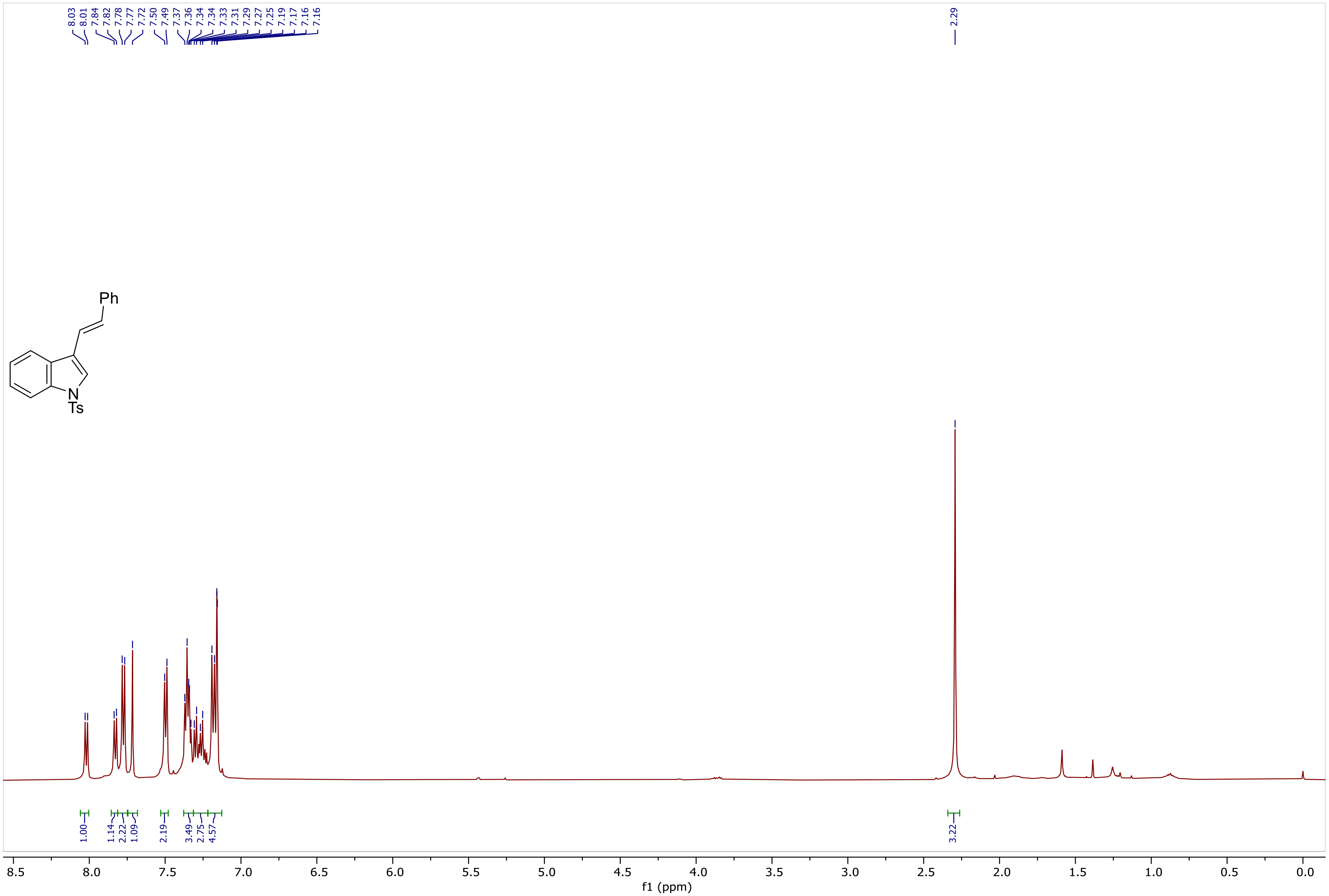

^{1}H NMR (400 MHz, $CDCl_3$) of compound **5p**

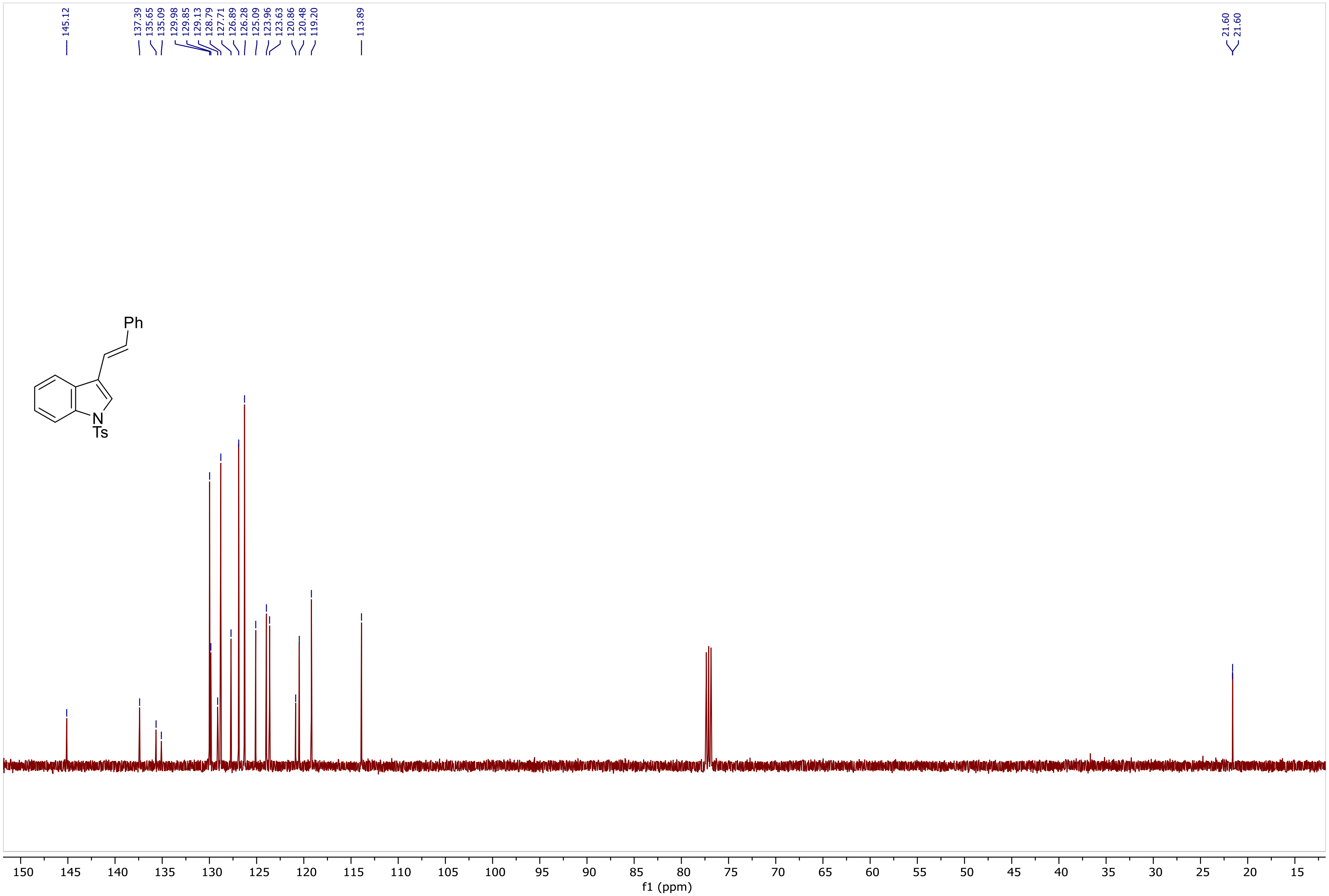

^{13}C NMR (101 MHz, $CDCl_3$) of compound **5p**

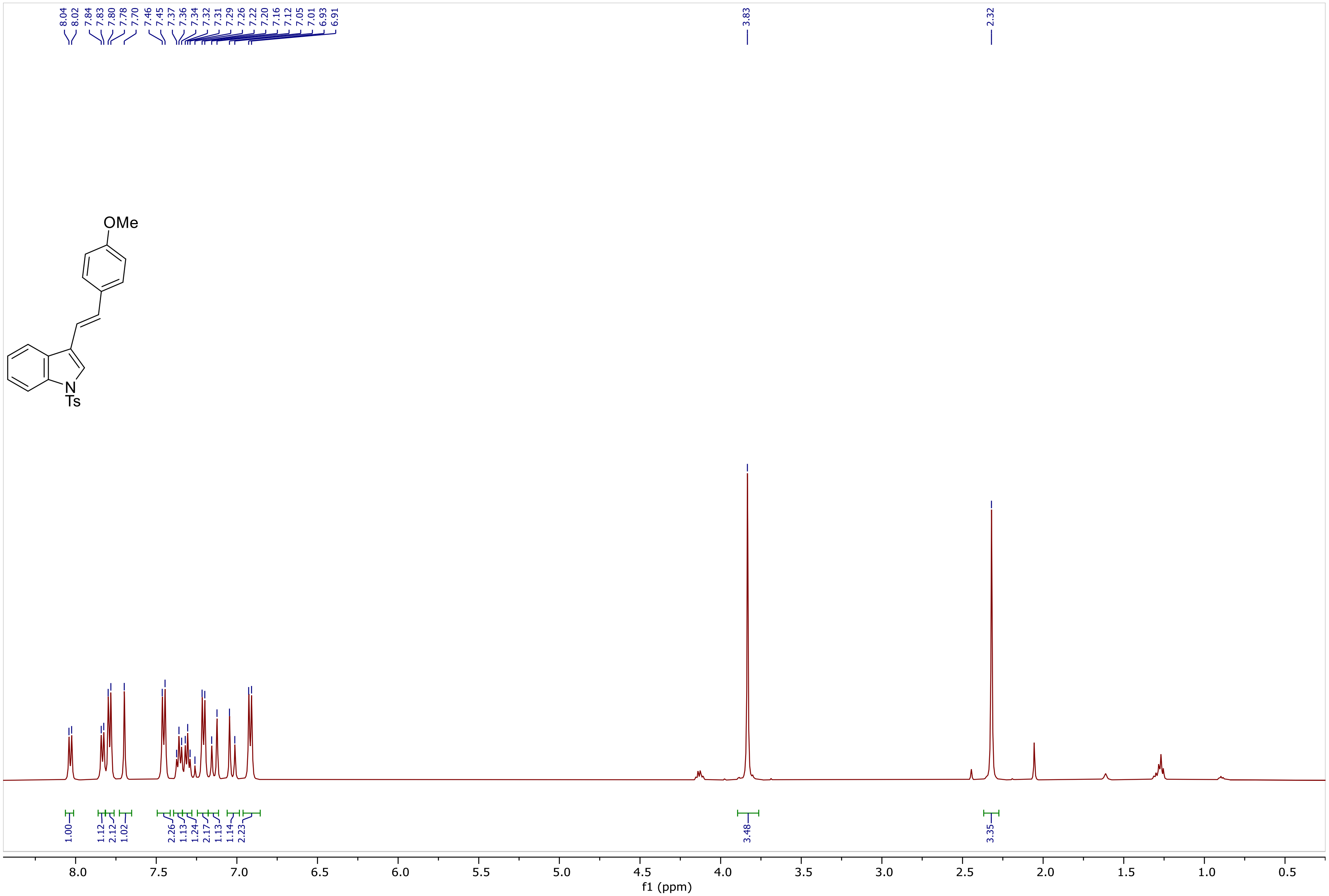

^{1}H NMR (400 MHz, $CDCl_3$) of compound **5q**

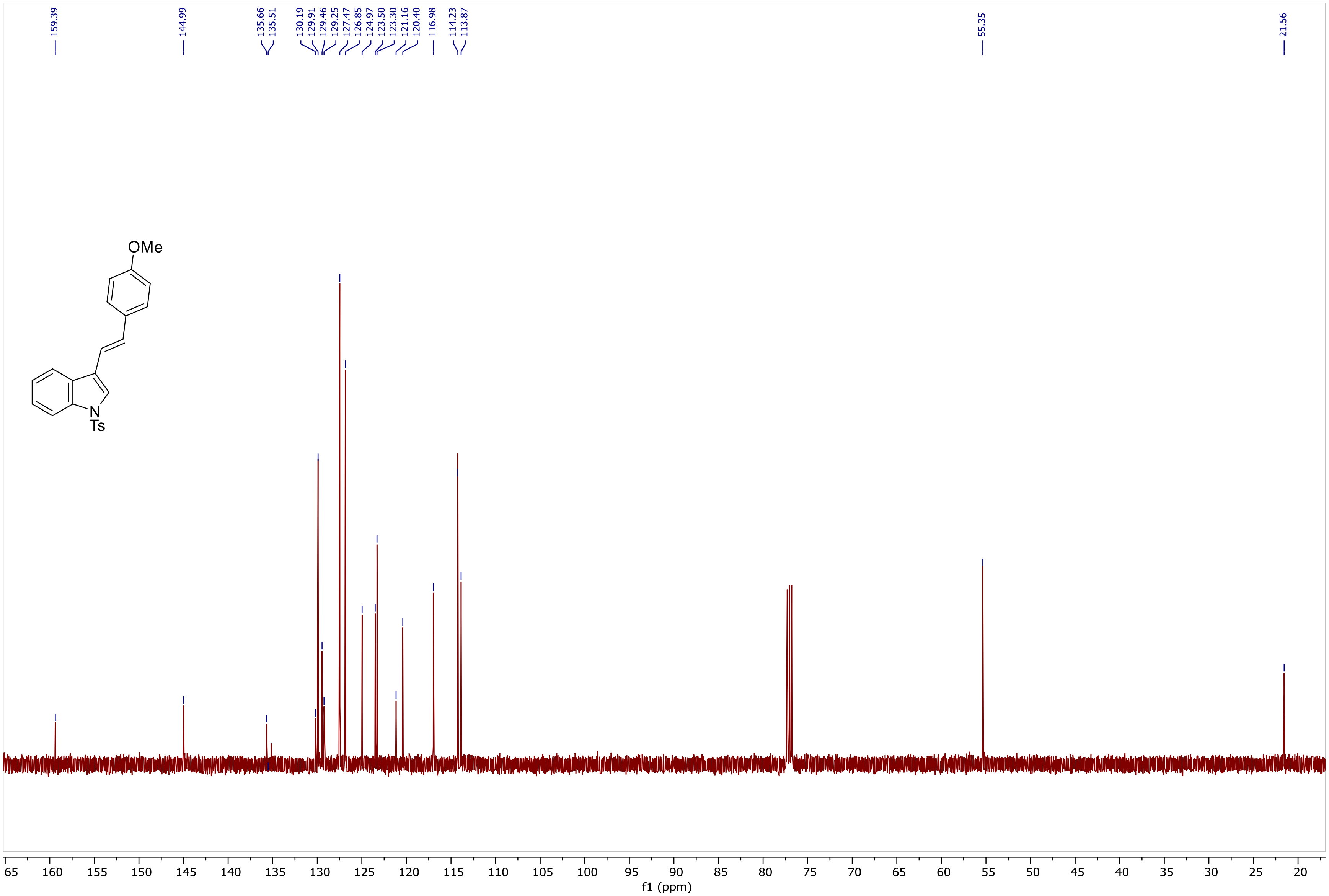

^{13}C NMR (101 MHz, $CDCl_3$) of compound **5q**

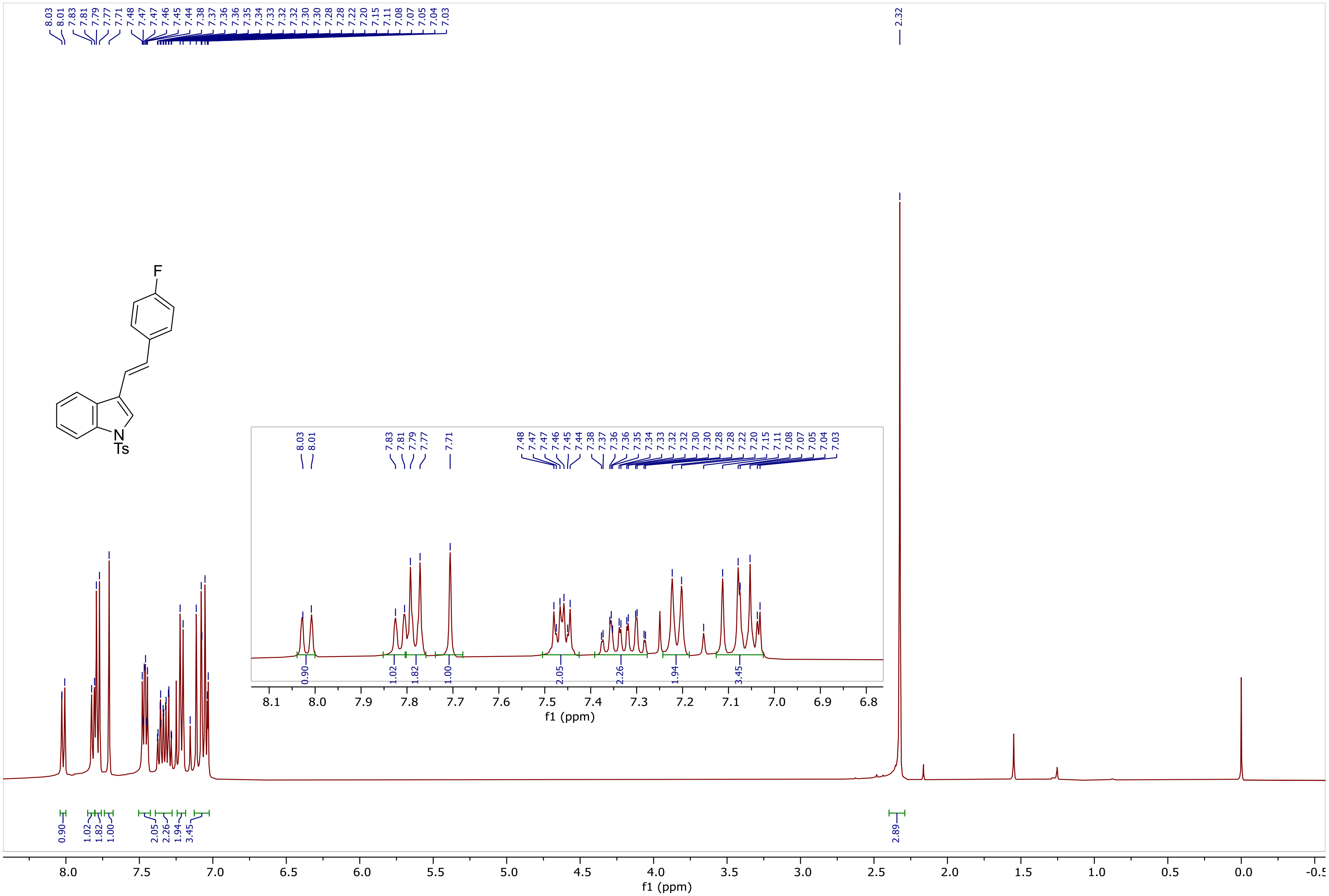

^{1}H NMR (400 MHz, $CDCl_3$) of compound **5r**

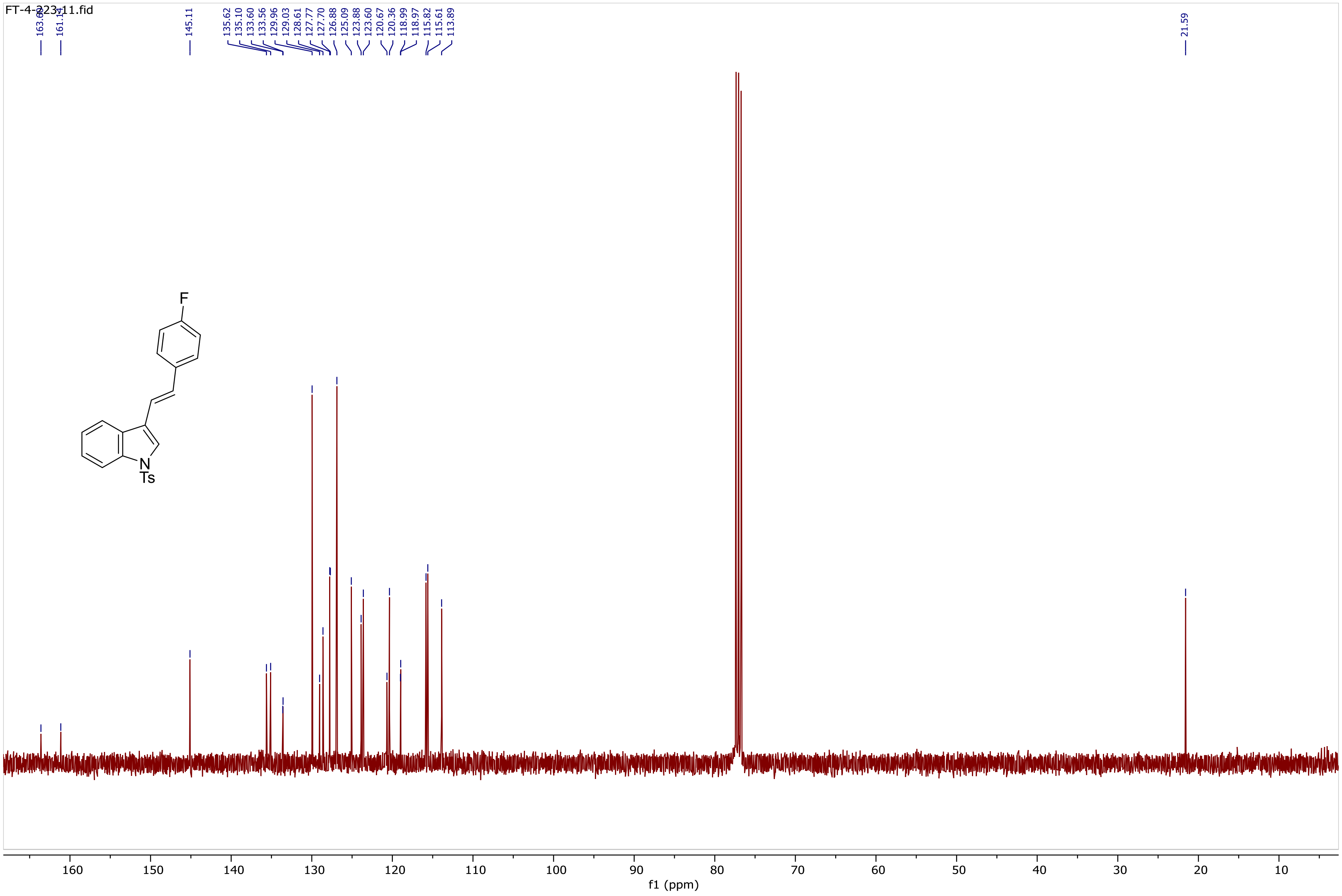

^{13}C NMR (101 MHz, $CDCl_3$) of compound **5r**

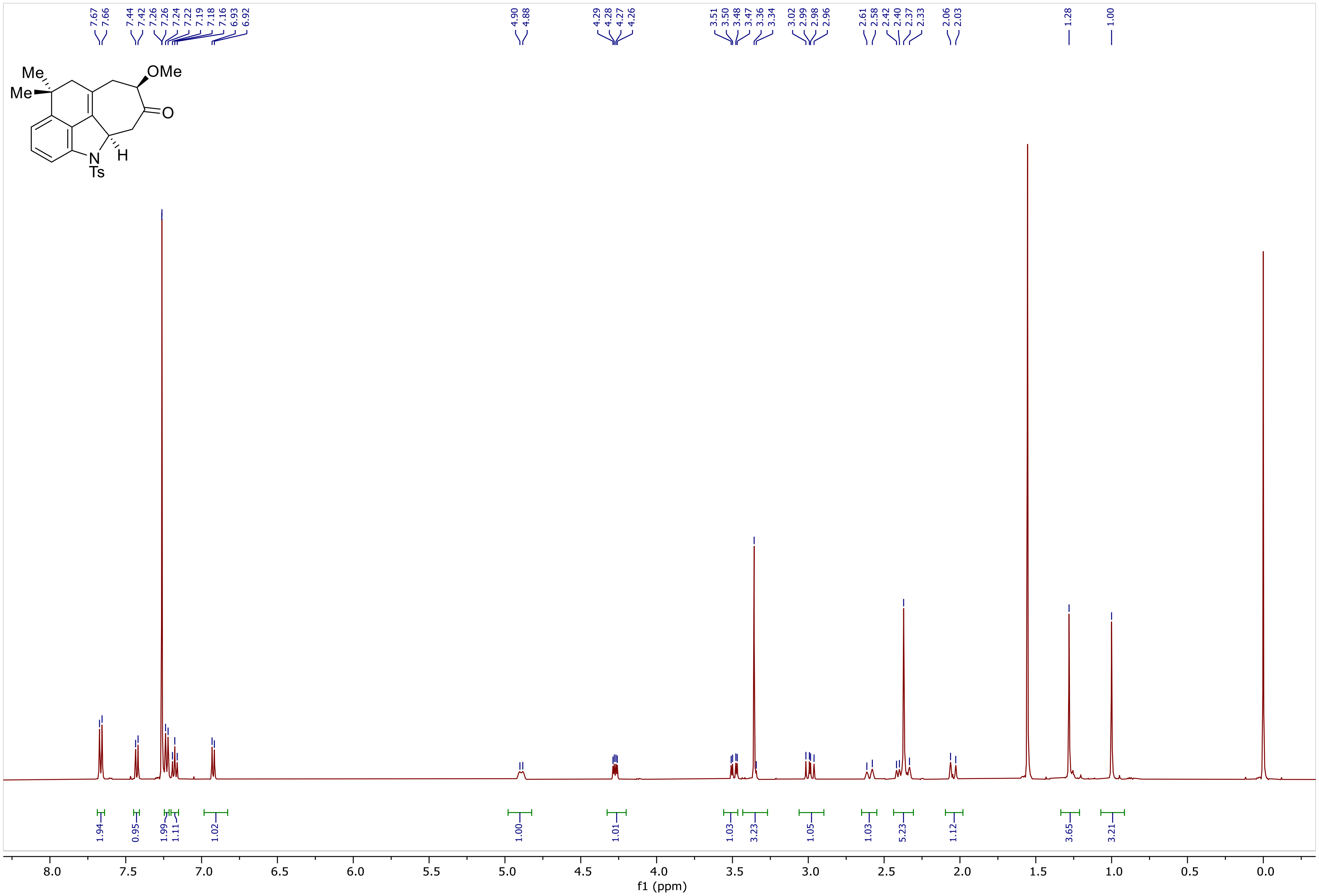

^{1}H NMR (400 MHz, $CDCl_3$) of compound **4a**

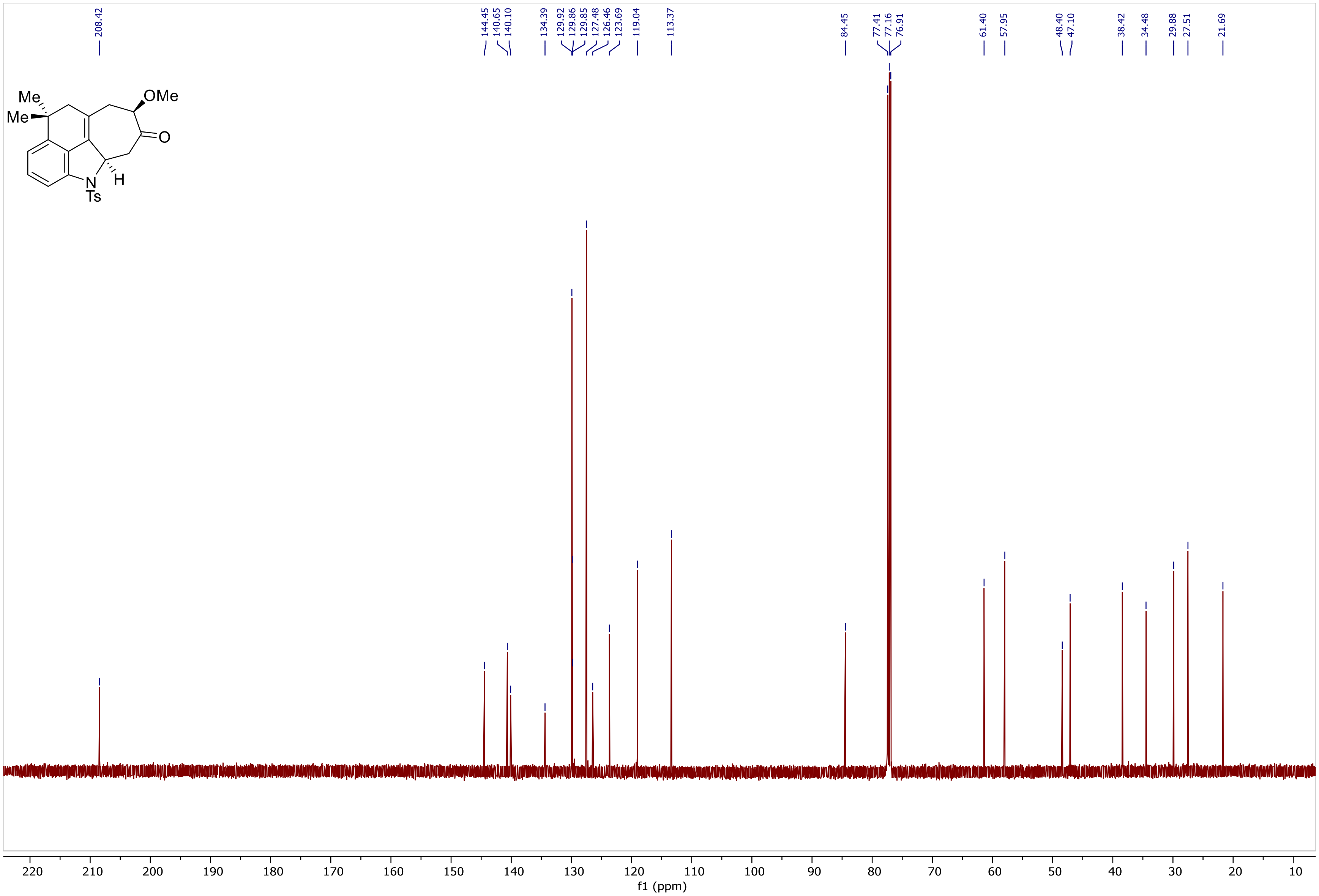

^{13}C NMR (101 MHz, $CDCl_3$) of compound **4a**

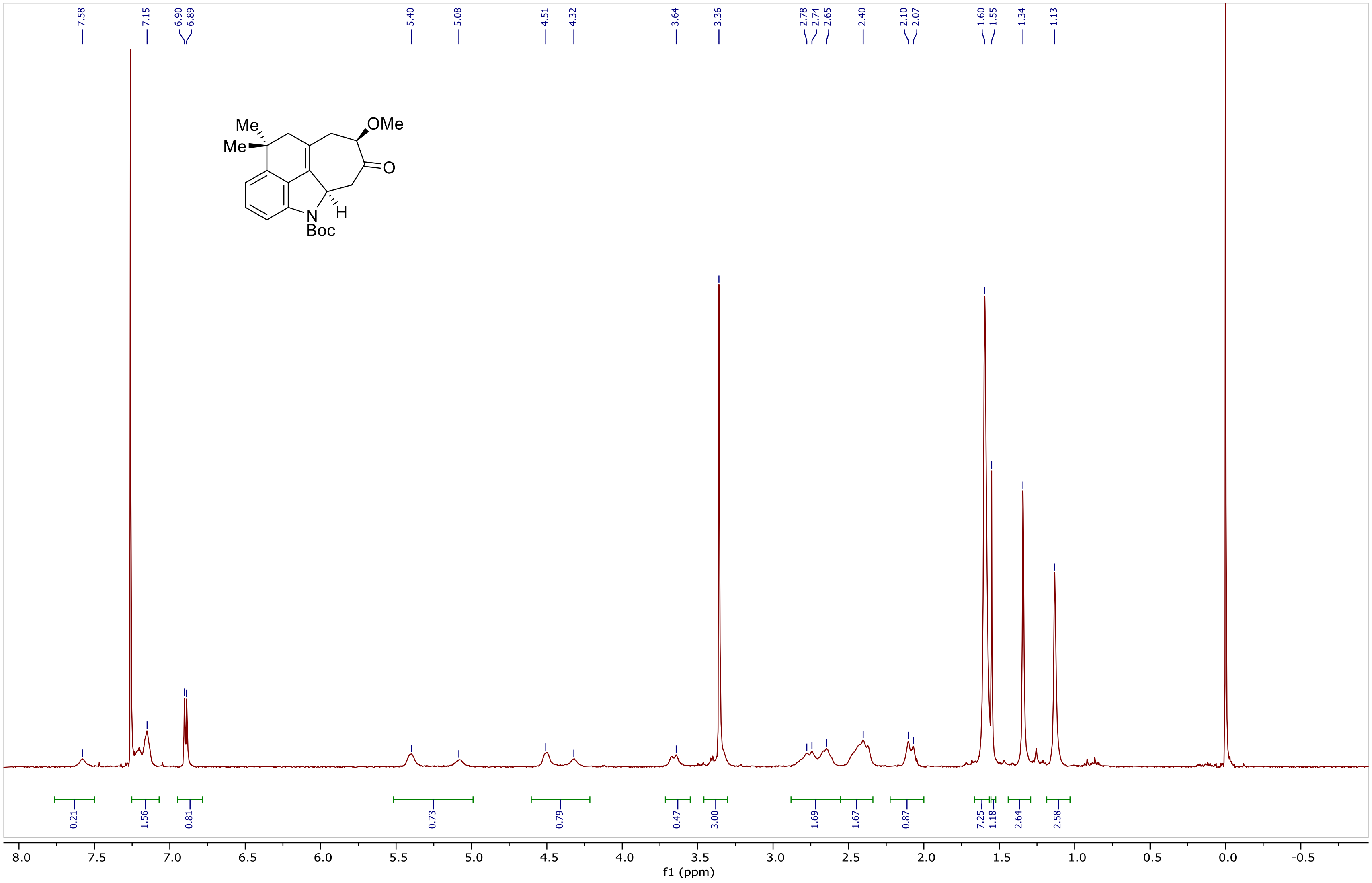

^{1}H NMR (400 MHz, $CDCl_3$) of compound **4b**

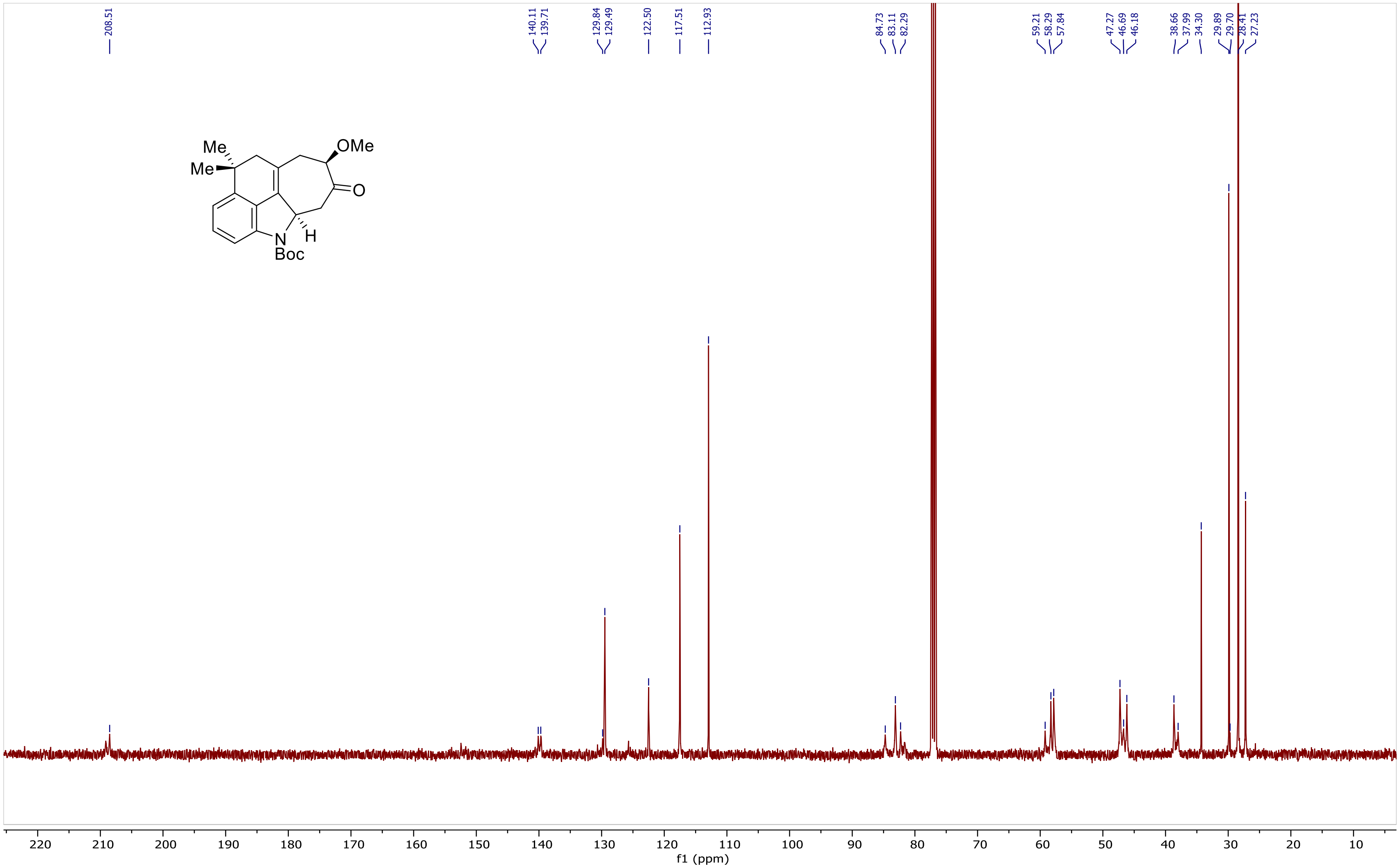

^{13}C NMR (101 MHz, $CDCl_3$) of compound **4b**

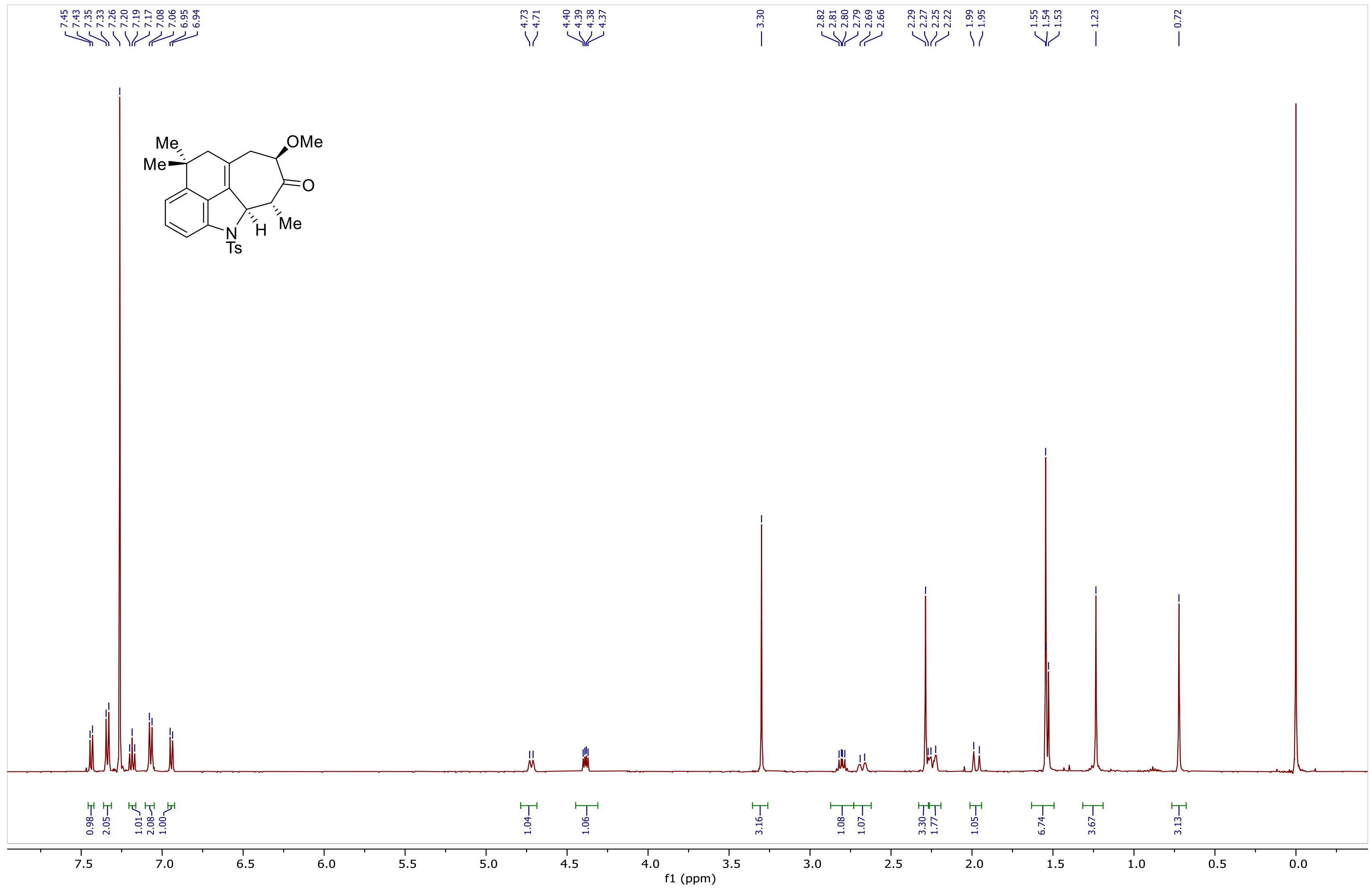

^{1}H NMR (400 MHz, $CDCl_3$) of compound **4c**

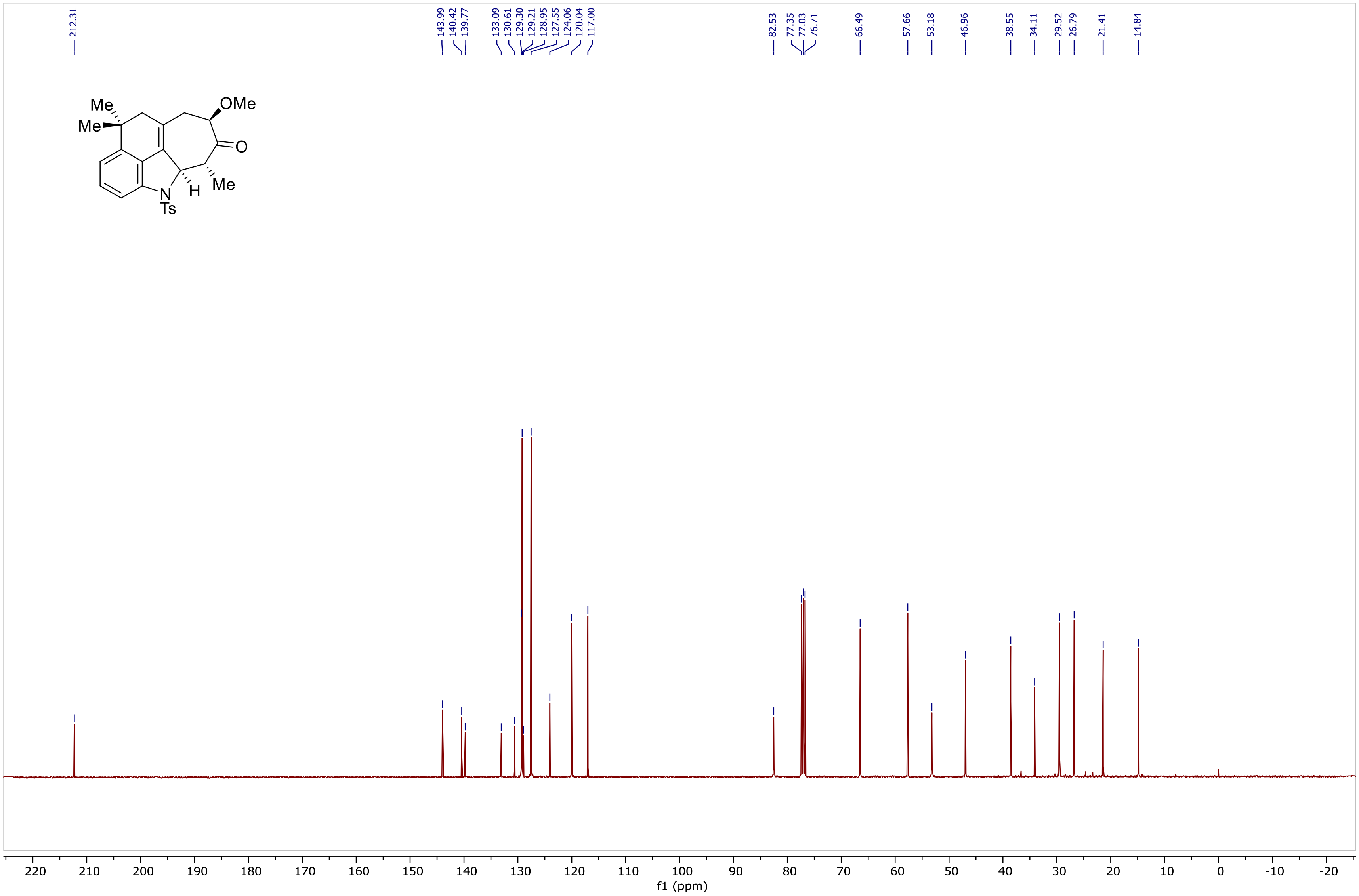

^{13}C NMR (101 MHz, $CDCl_3$) of compound **4c**

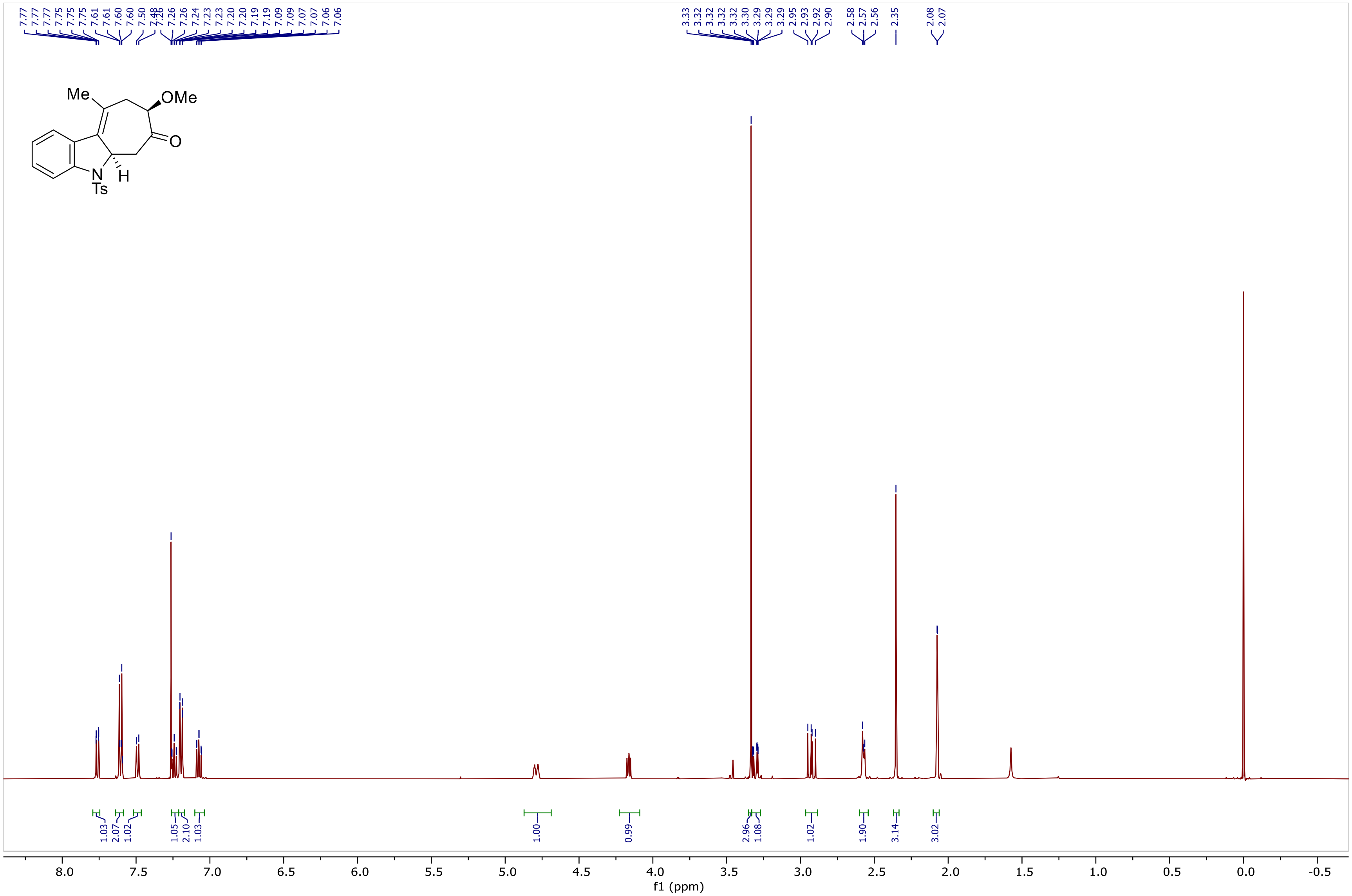

^{1}H NMR (400 MHz, $CDCl_3$) of compound **6a**

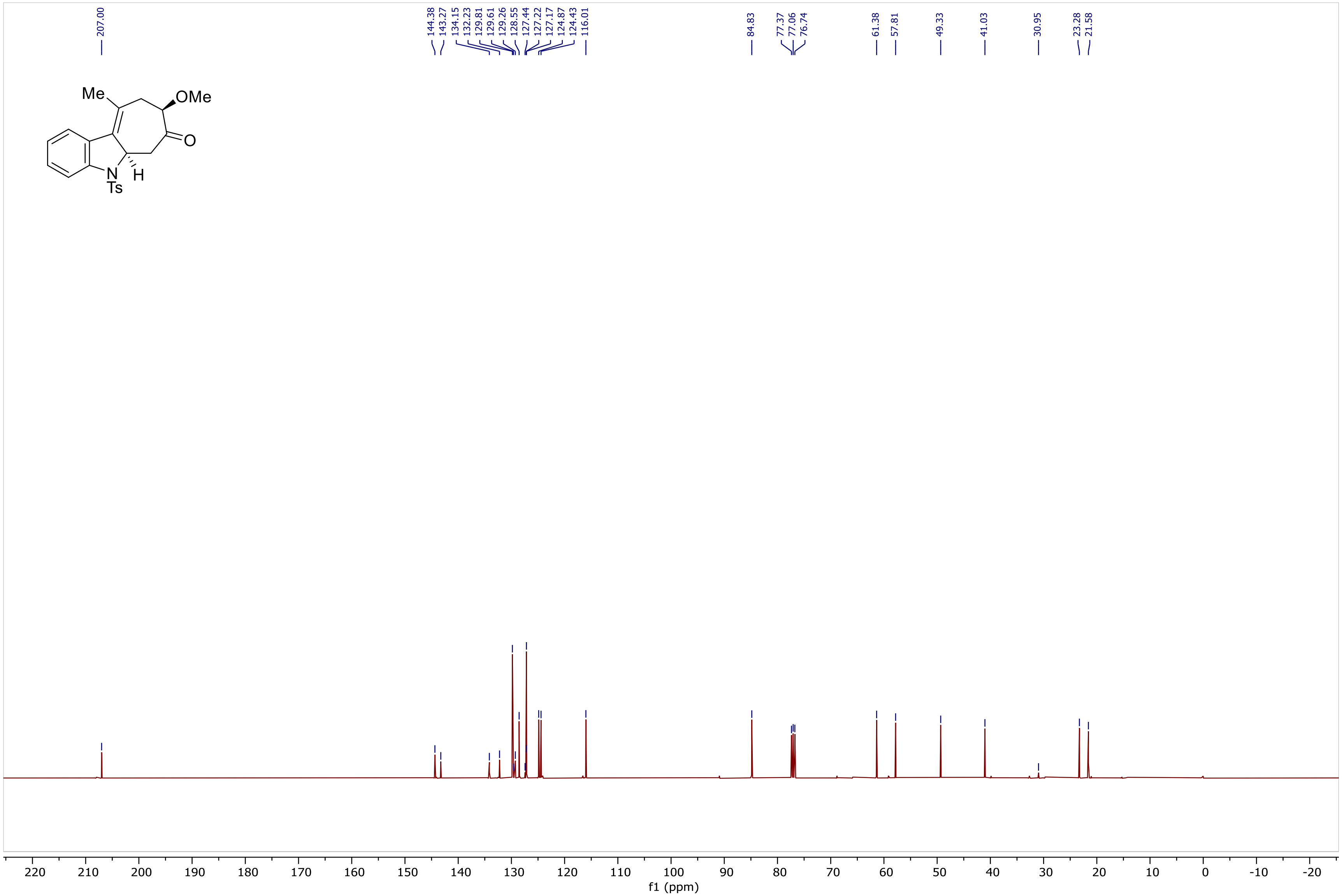

^{13}C NMR (101 MHz, $CDCl_3$) of compound **6a**

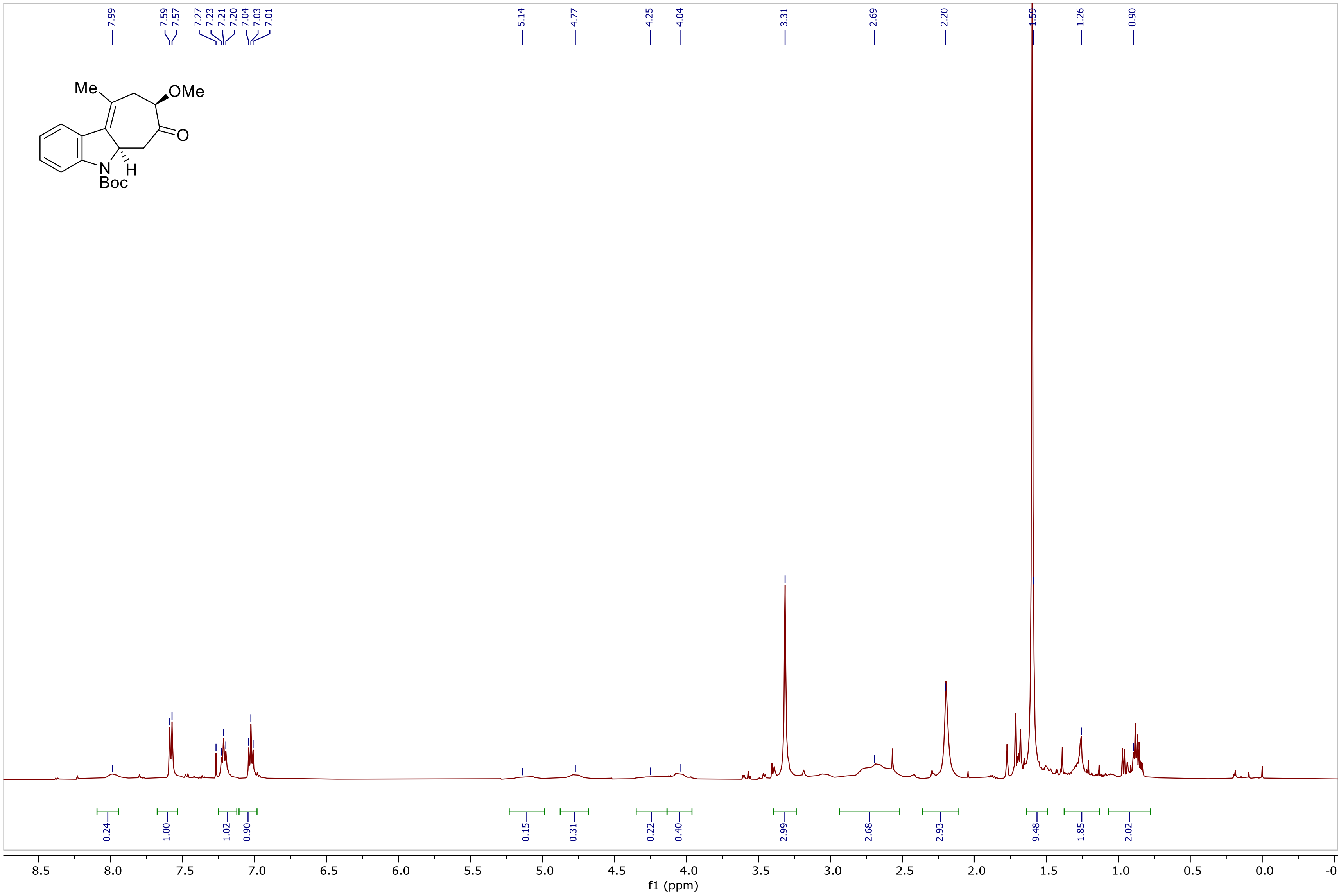

^{1}H NMR (400 MHz, $CDCl_3$) of compound **6b**

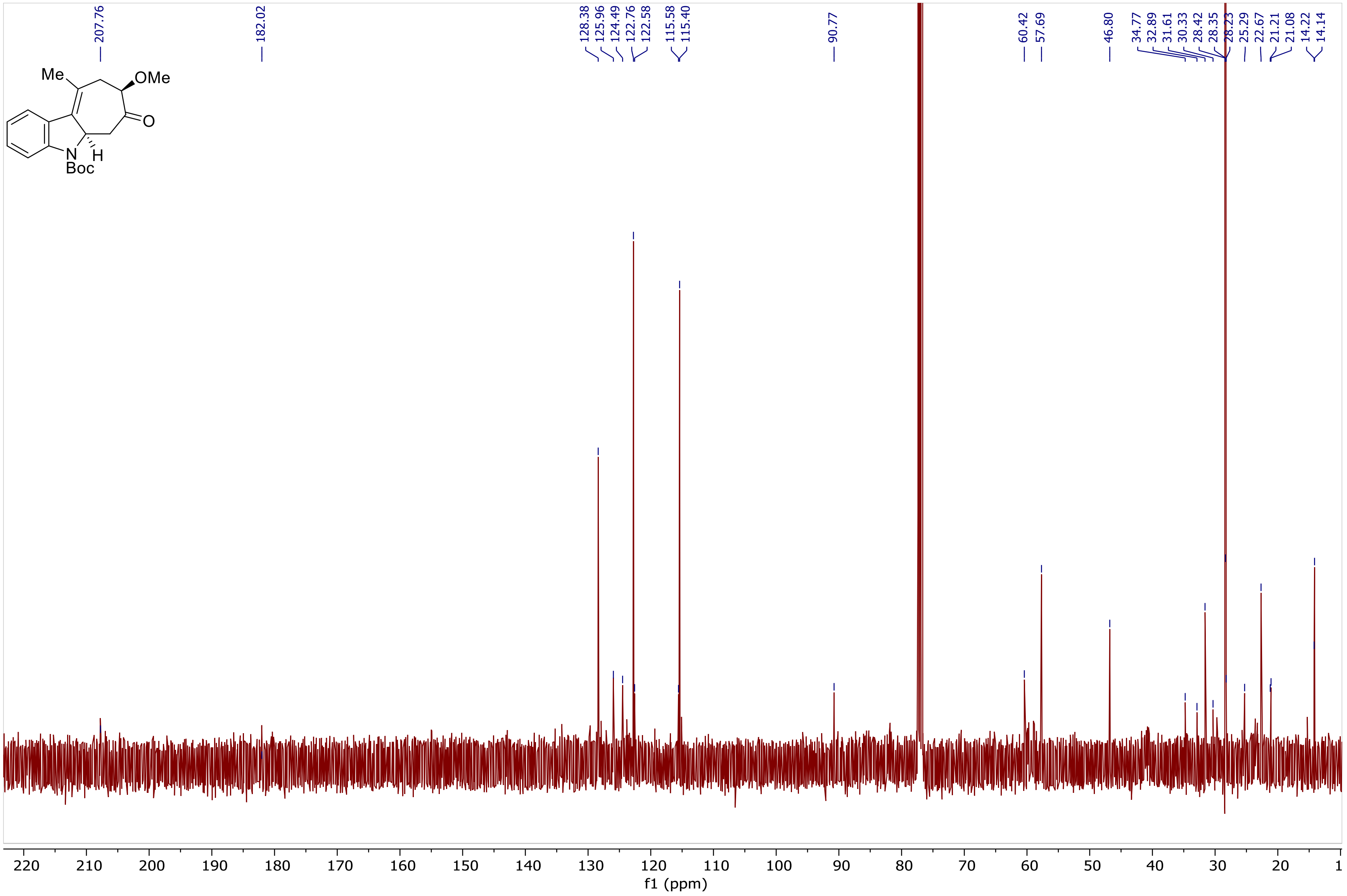

^{13}C NMR (101 MHz, $CDCl_3$) of compound **6b**

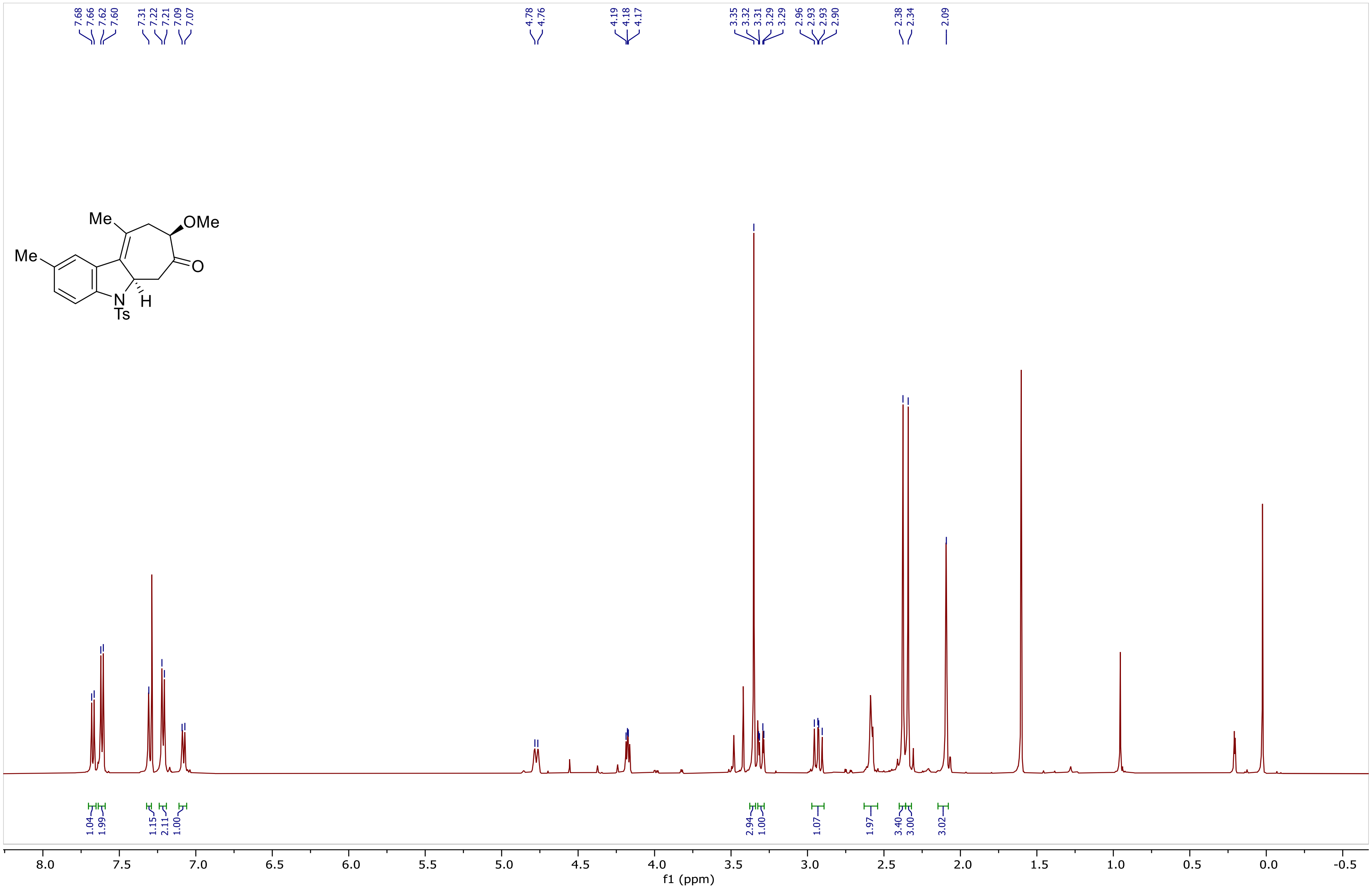

^{1}H NMR (400 MHz, $CDCl_3$) of compound **6c**

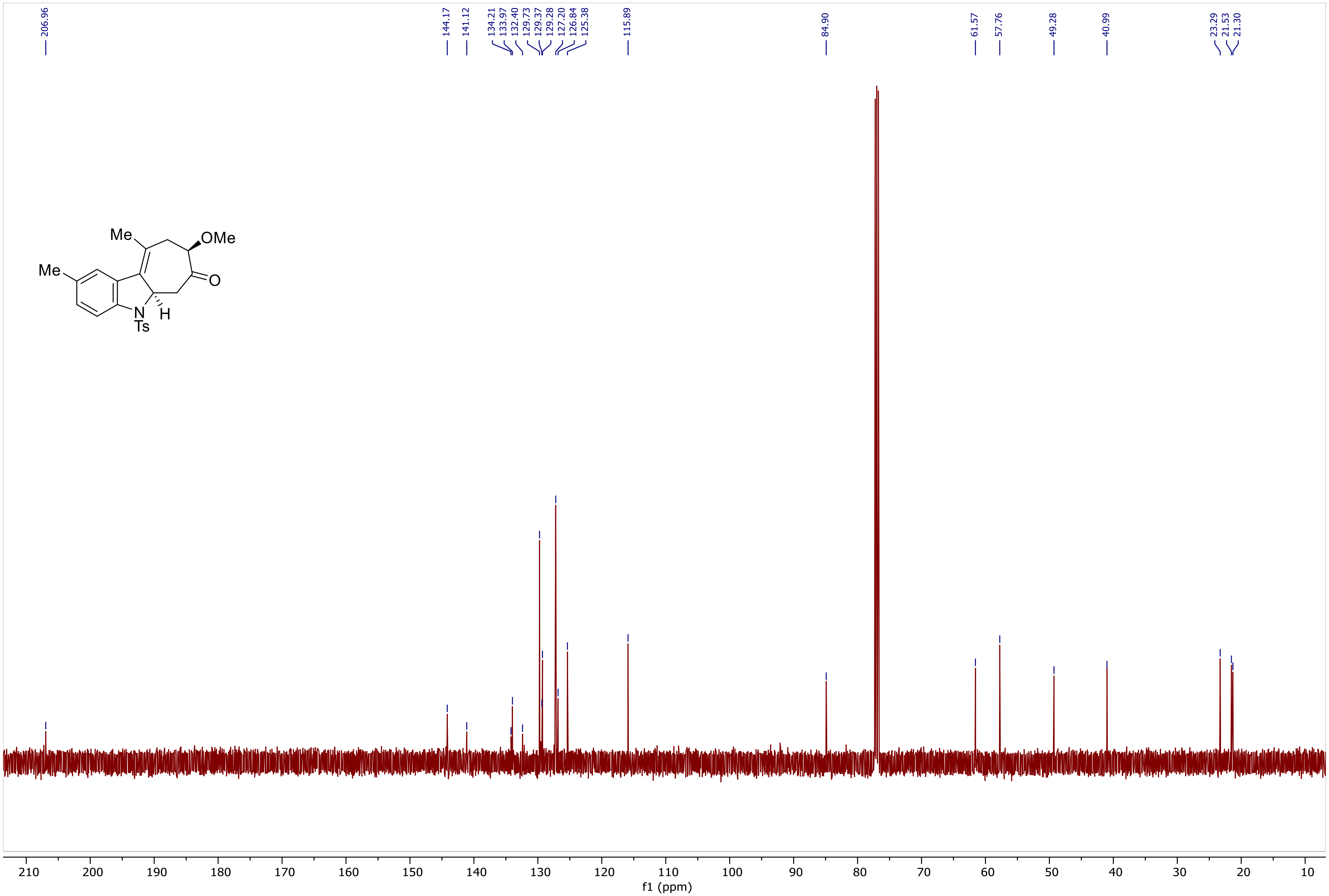

^{13}C NMR (101 MHz, $CDCl_3$) of compound **6c**

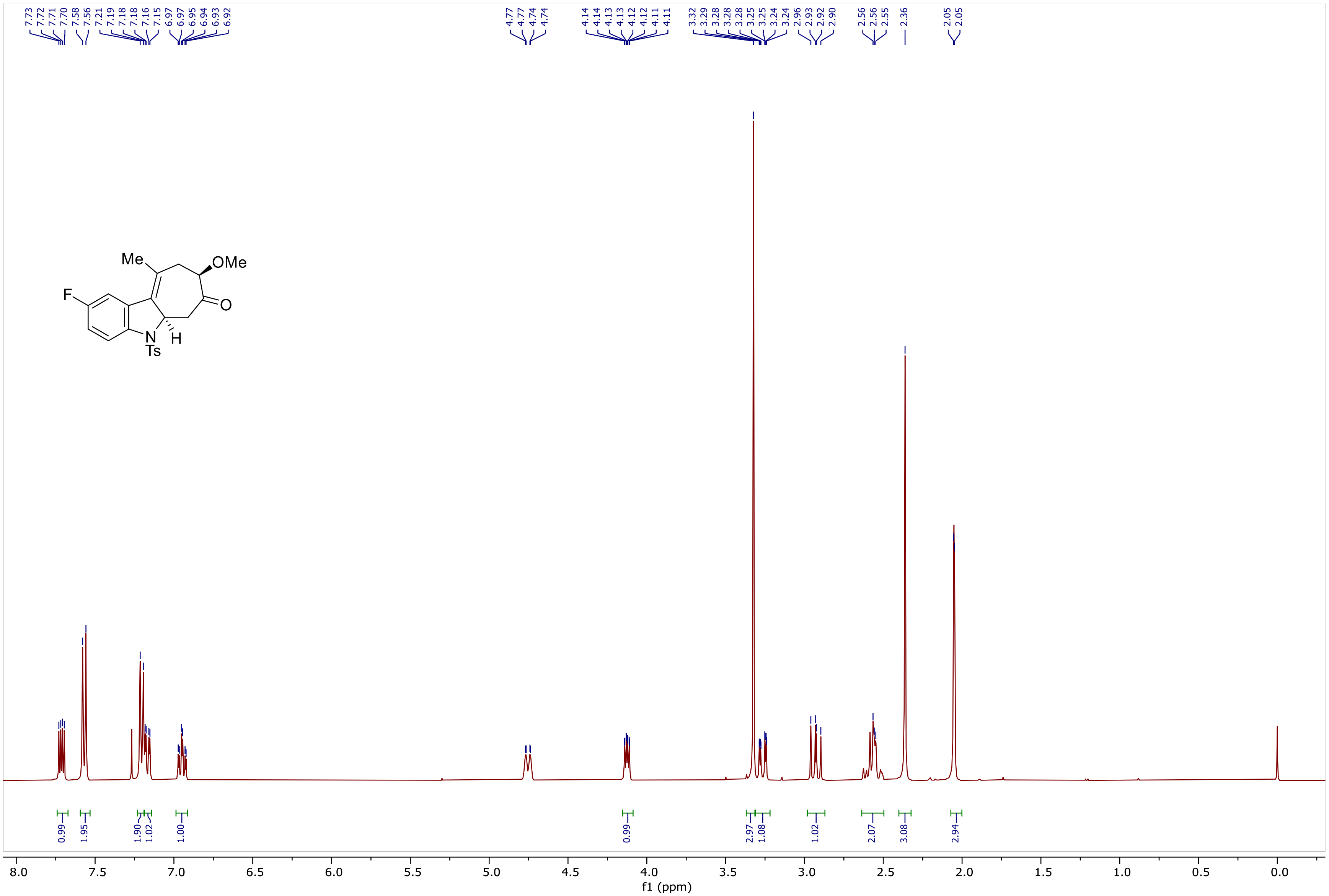

^{1}H NMR (400 MHz, $CDCl_3$) of compound **6d**

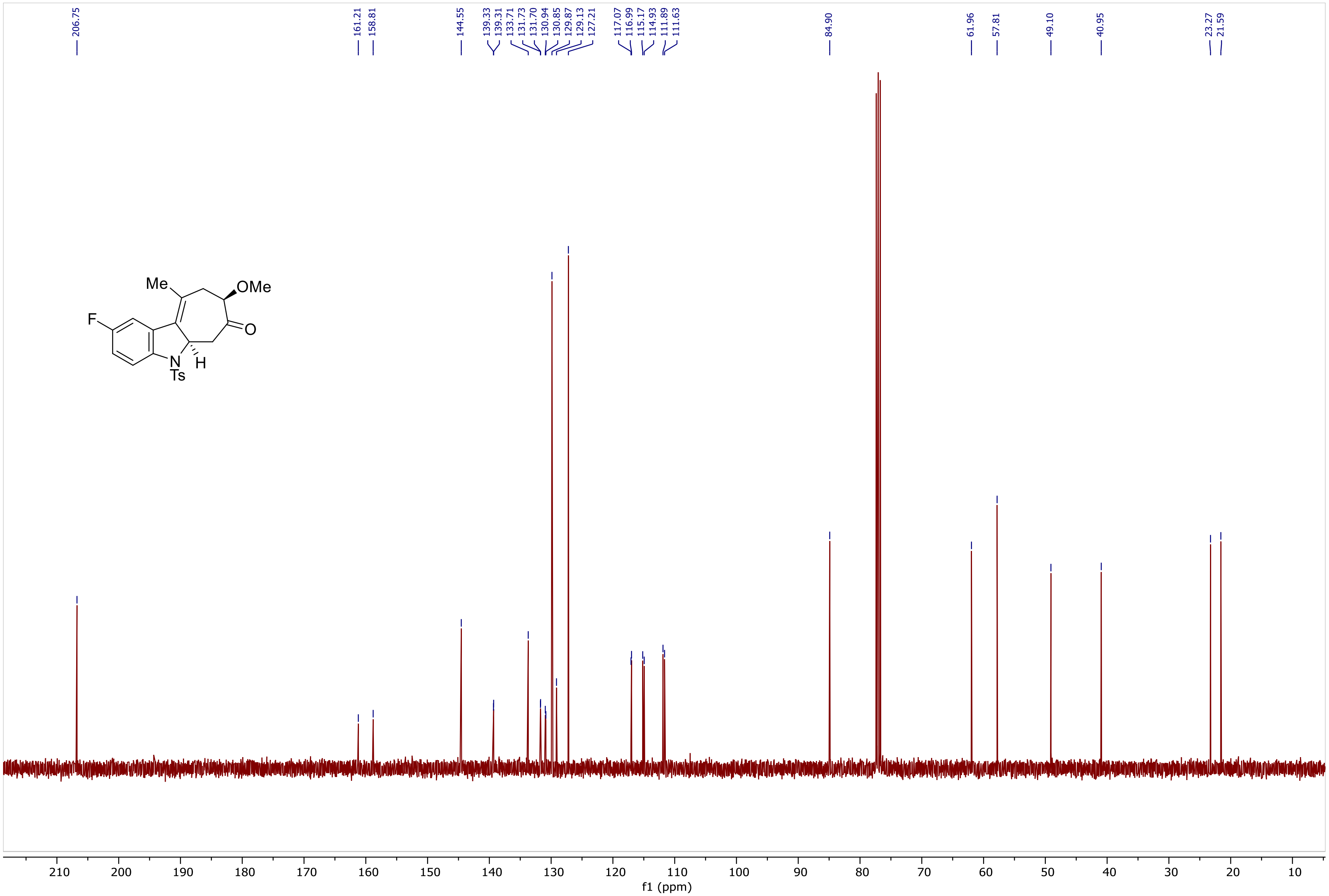

^{13}C NMR (101 MHz, $CDCl_3$) of compound **6d**

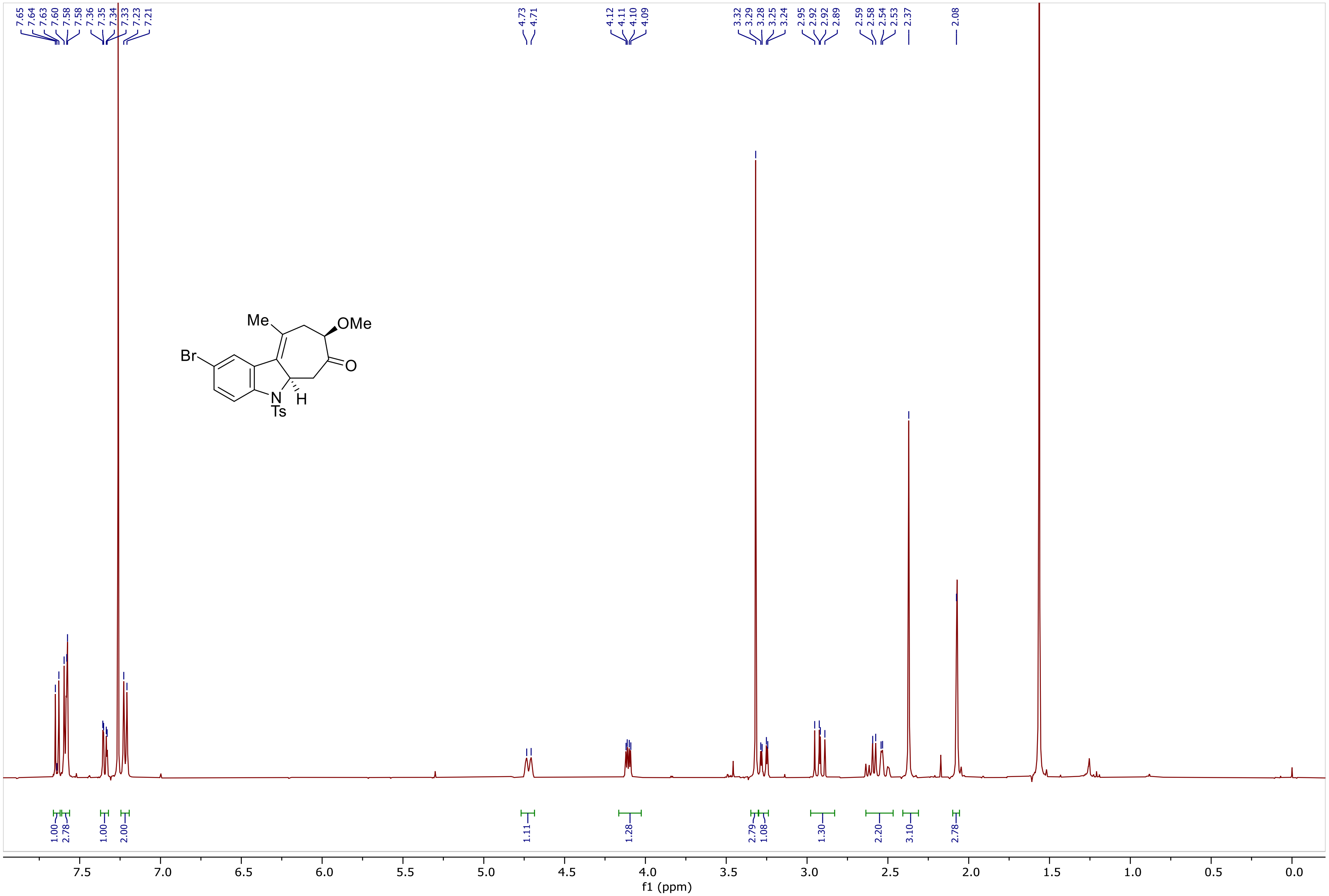

^{1}H NMR (400 MHz, $CDCl_3$) of compound **6e**

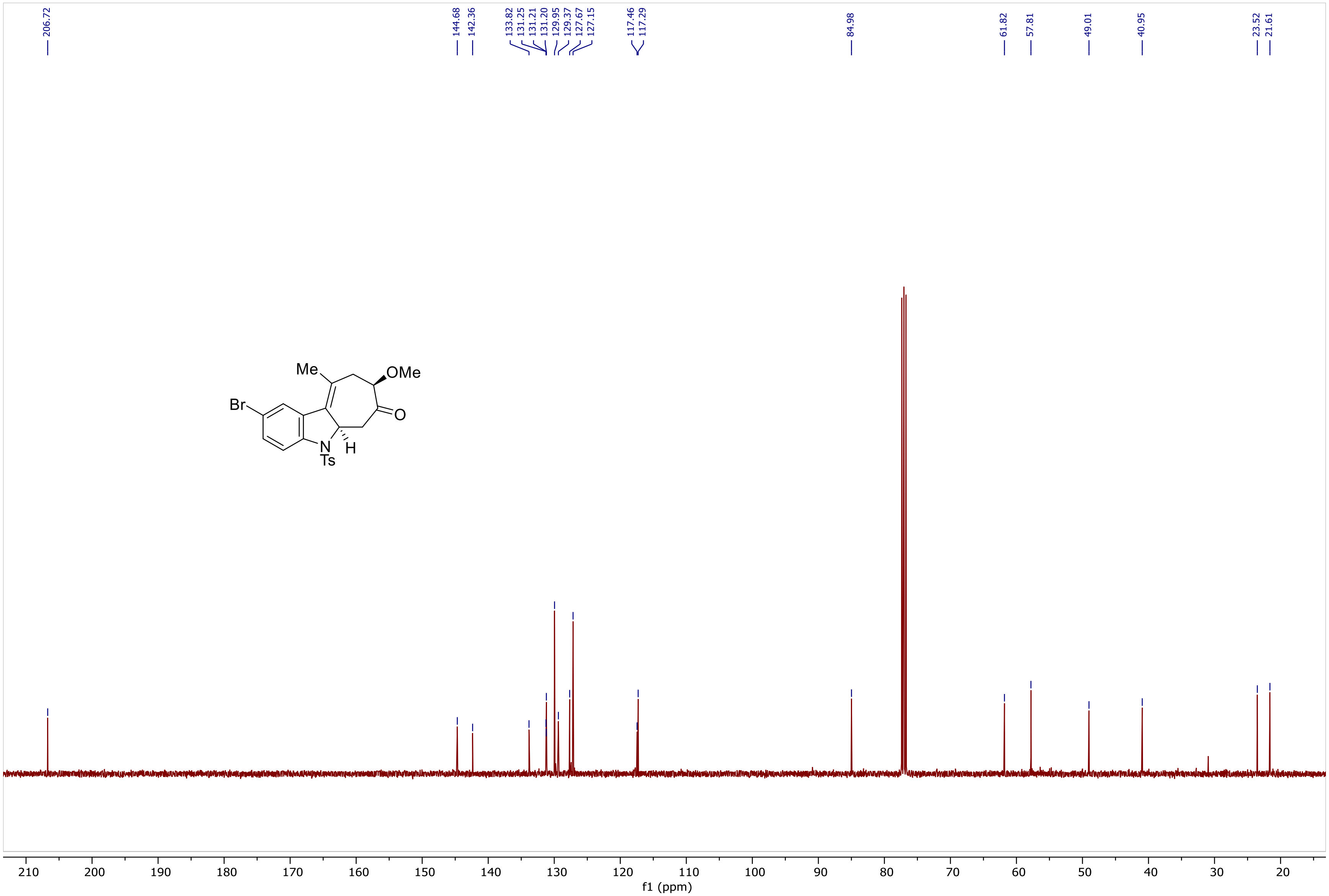

^{13}C NMR (101 MHz, $CDCl_3$) of compound **6e**

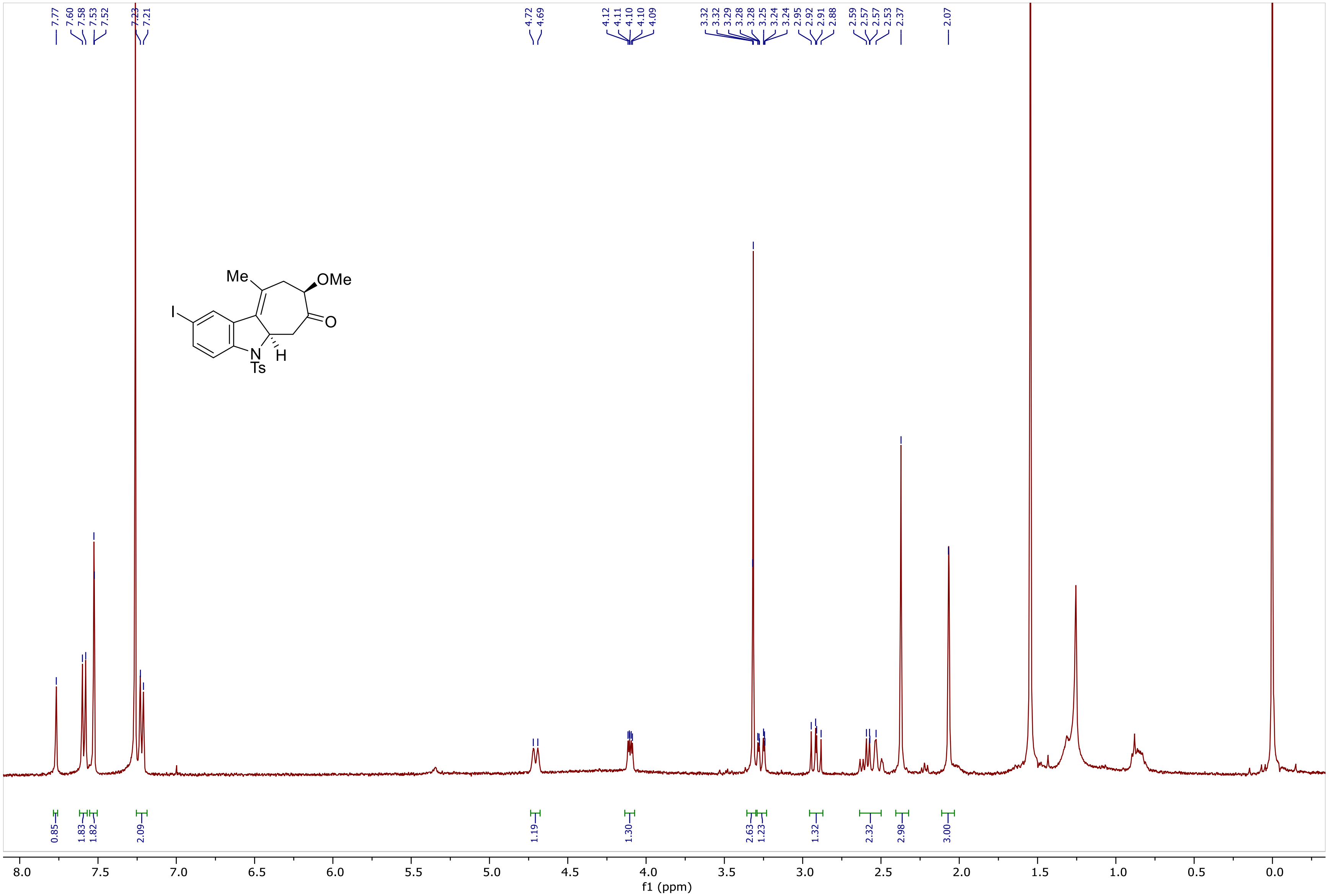

^{1}H NMR (400 MHz, $CDCl_3$) of compound **6f**

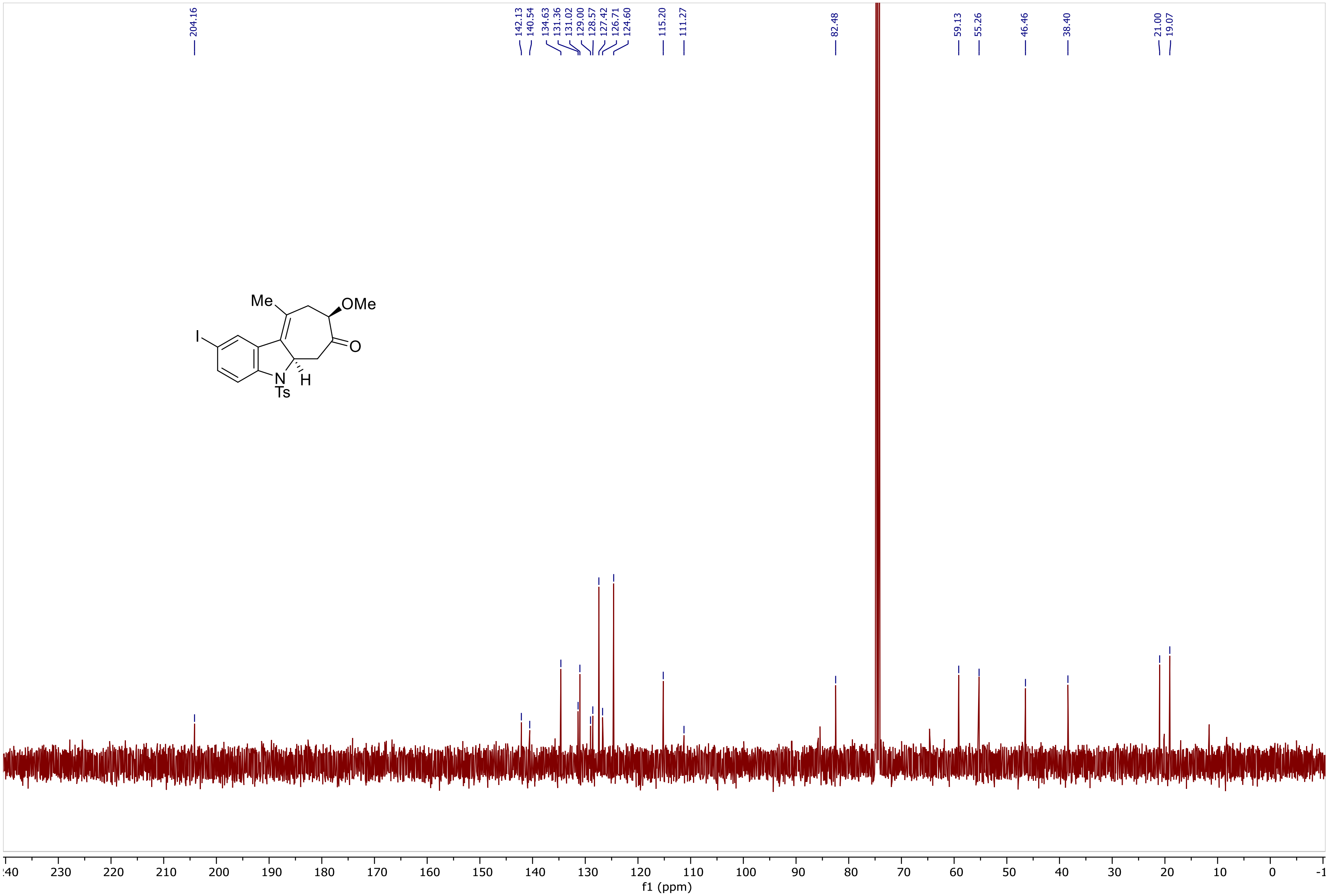

^{13}C NMR (101 MHz, CH_3CN+D_2O) of compound **6f**

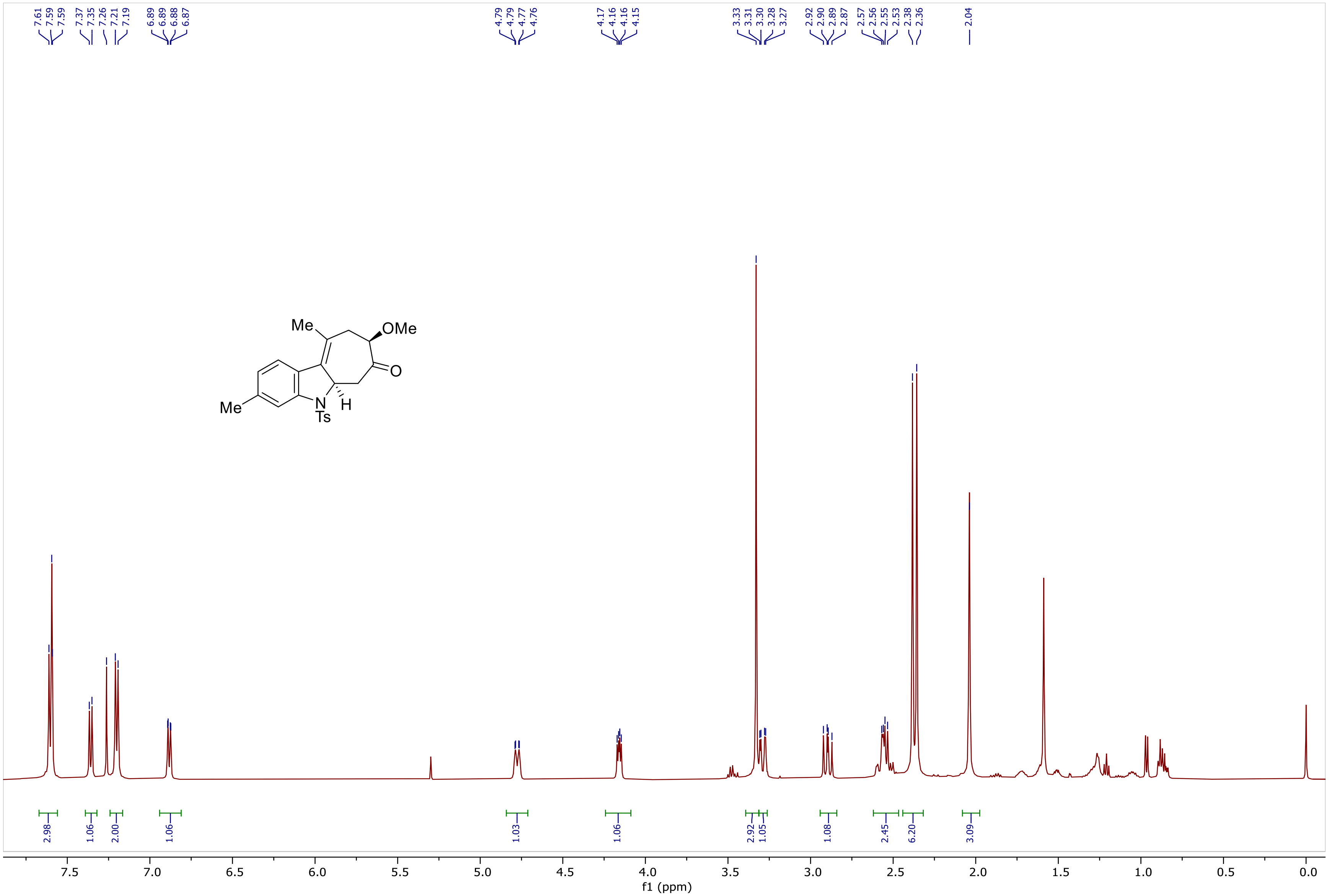

^{1}H NMR (400 MHz, $CDCl_3$) of compound **6g**

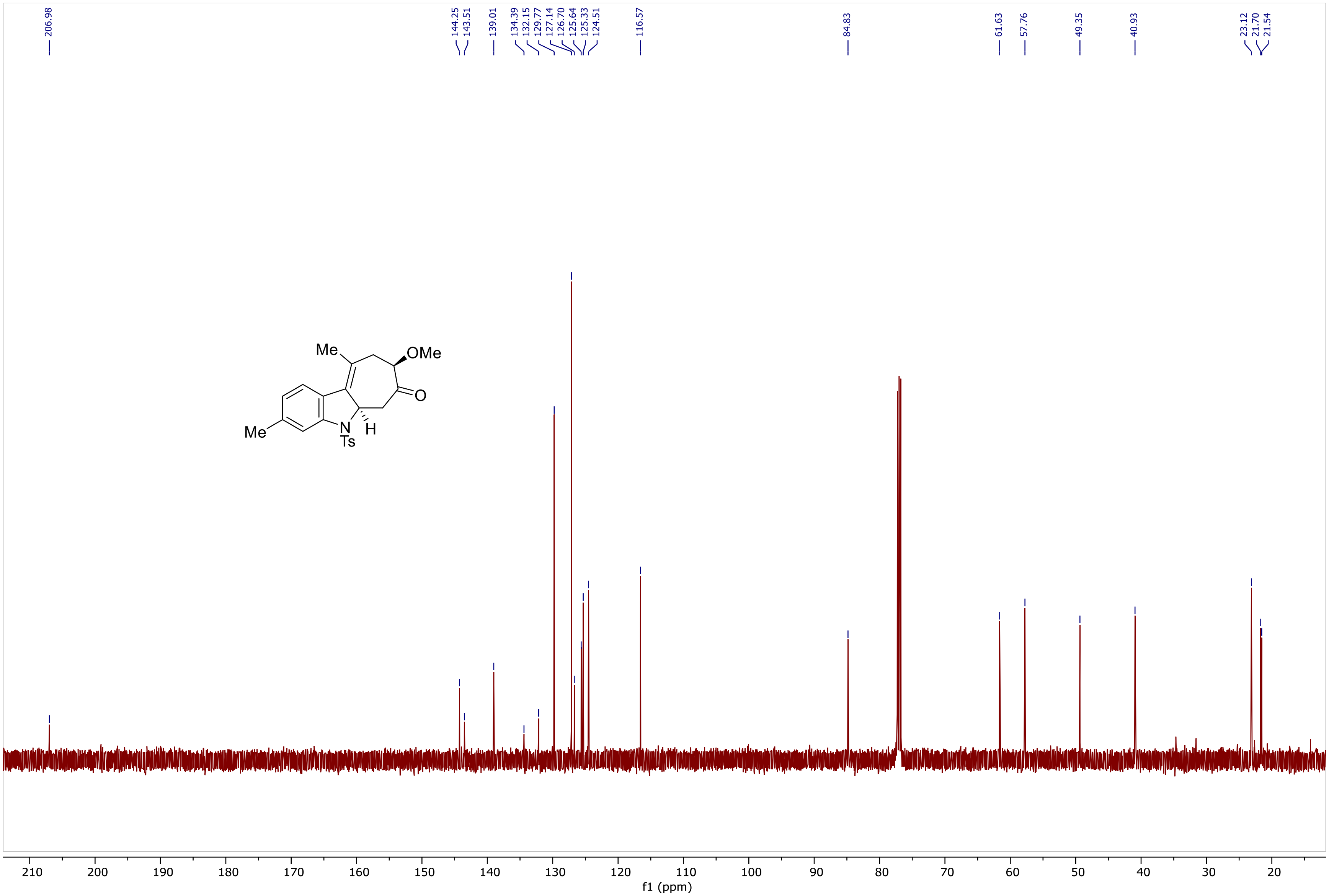

^{13}C NMR (101 MHz, $CDCl_3$) of compound **6g**

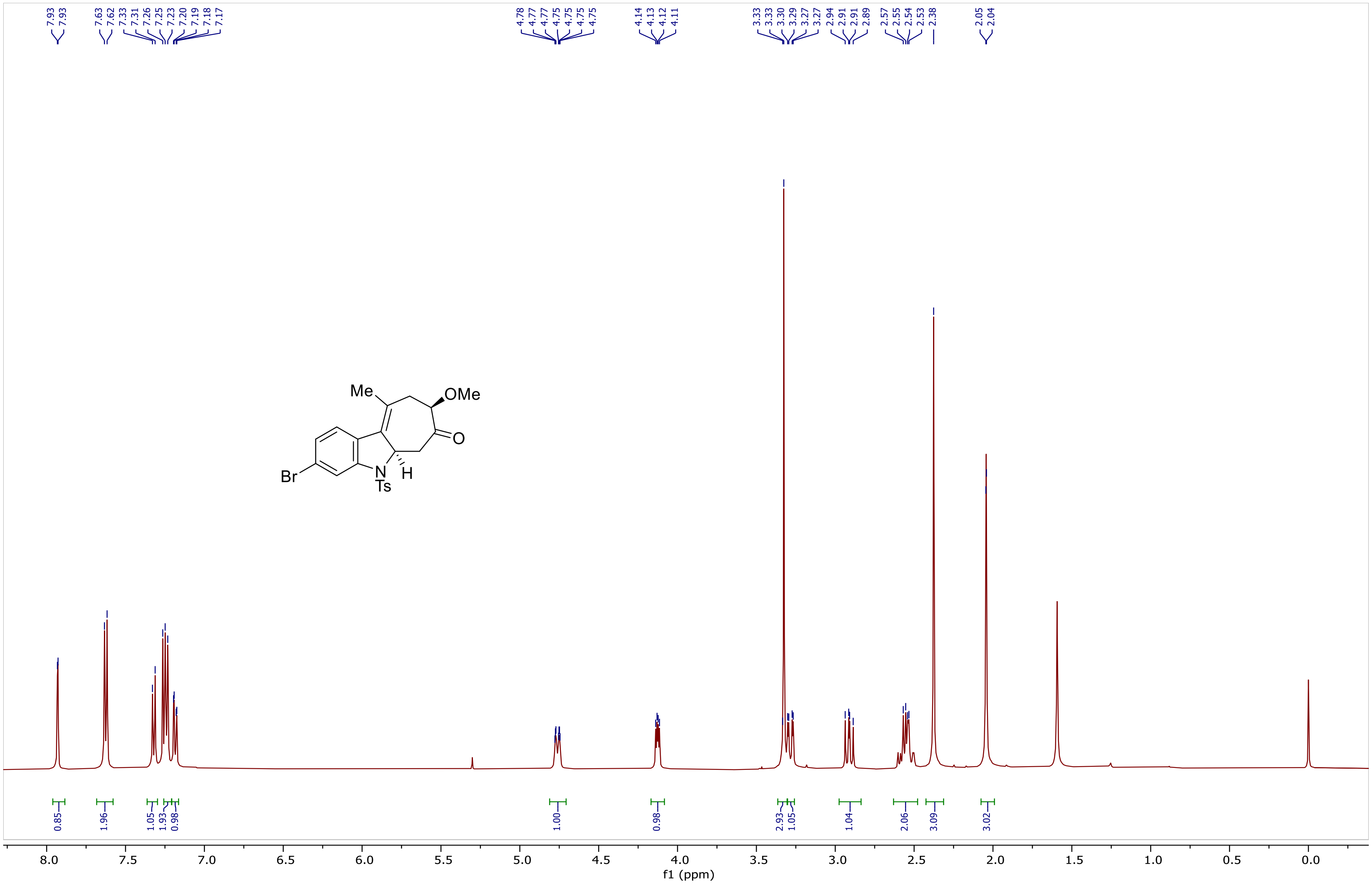

^{1}H NMR (400 MHz, $CDCl_3$) of compound **6h**

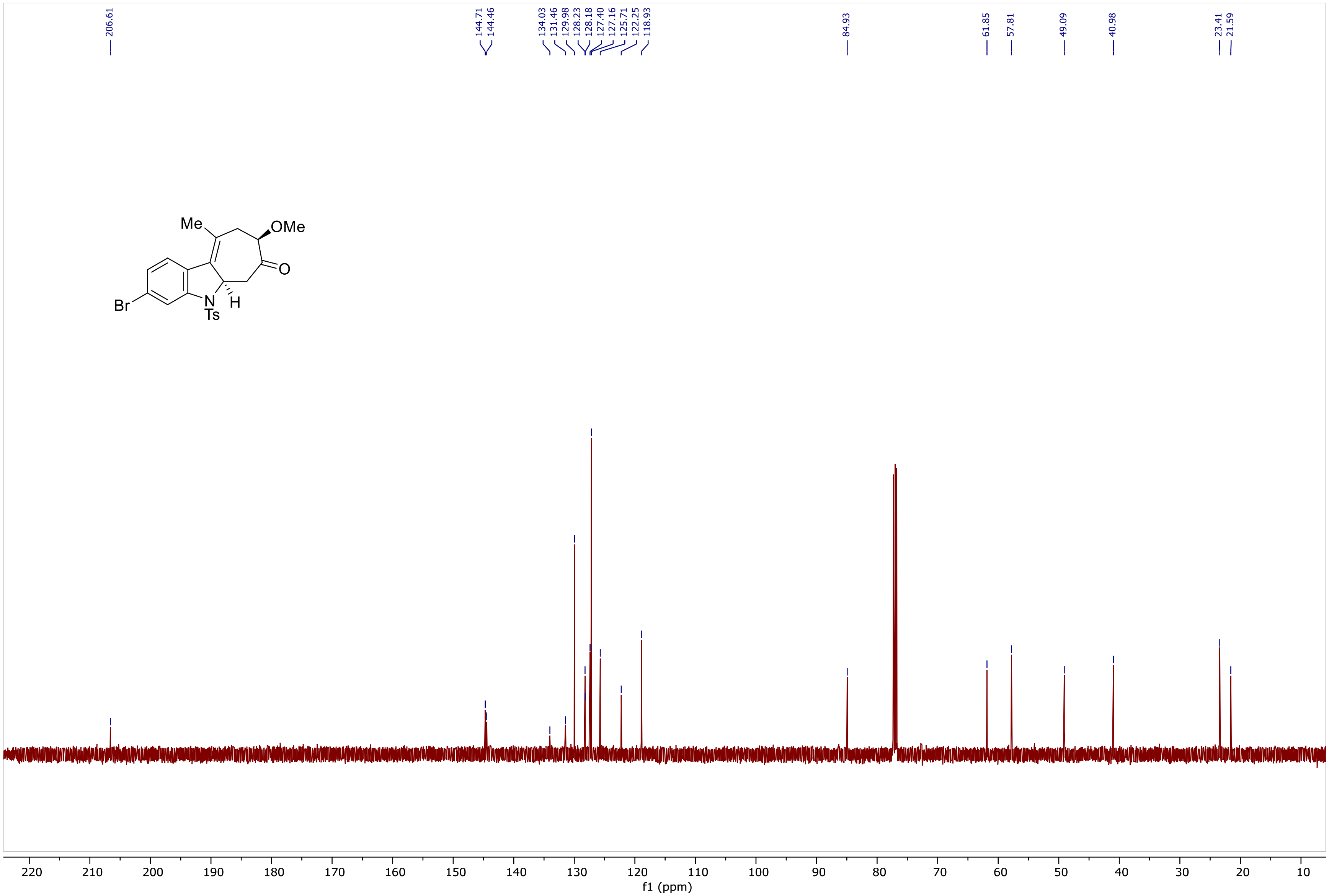

^{13}C NMR (101 MHz, $CDCl_3$) of compound of **6h**

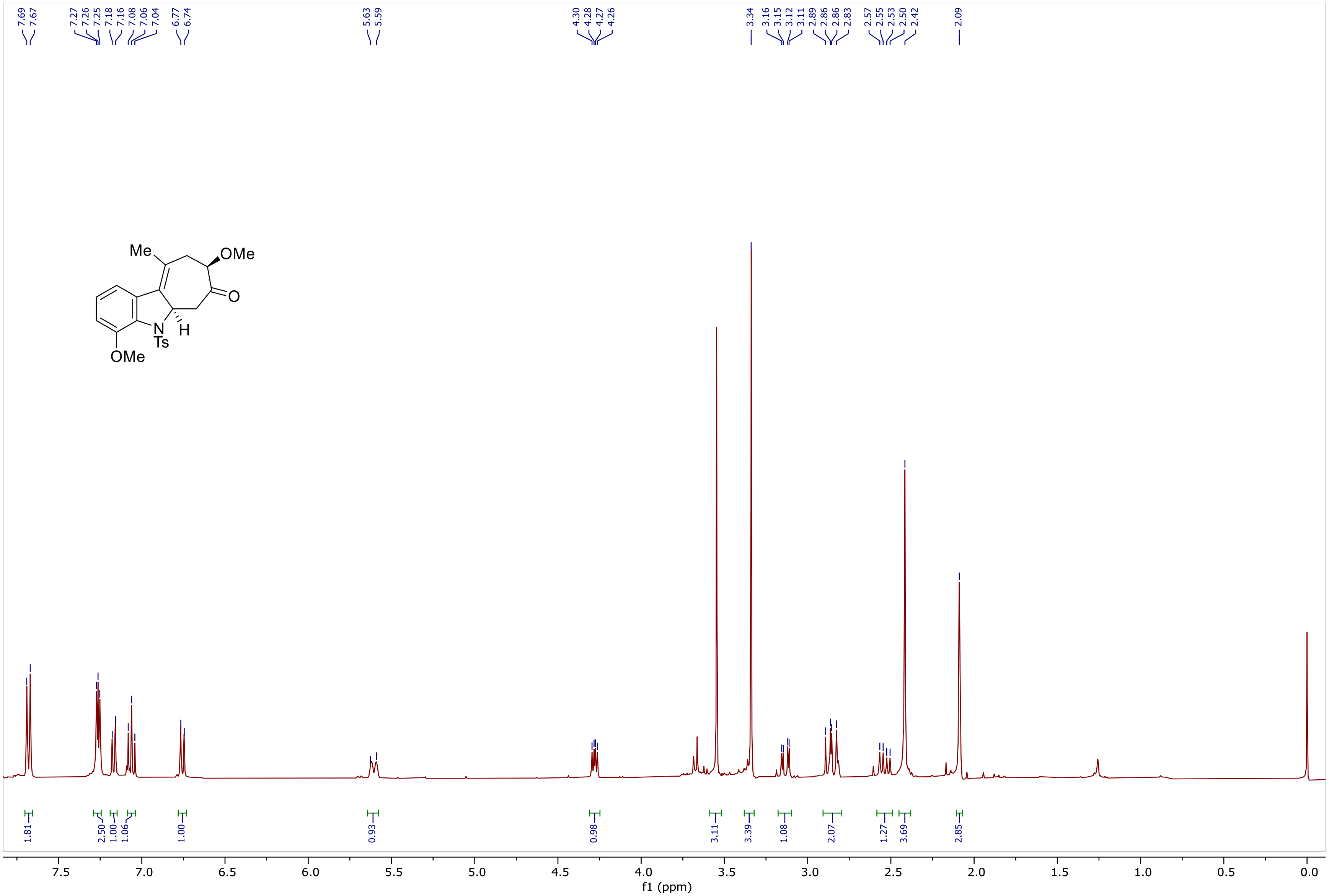

^{1}H NMR (400 MHz, $CDCl_3$) of compound **6i**

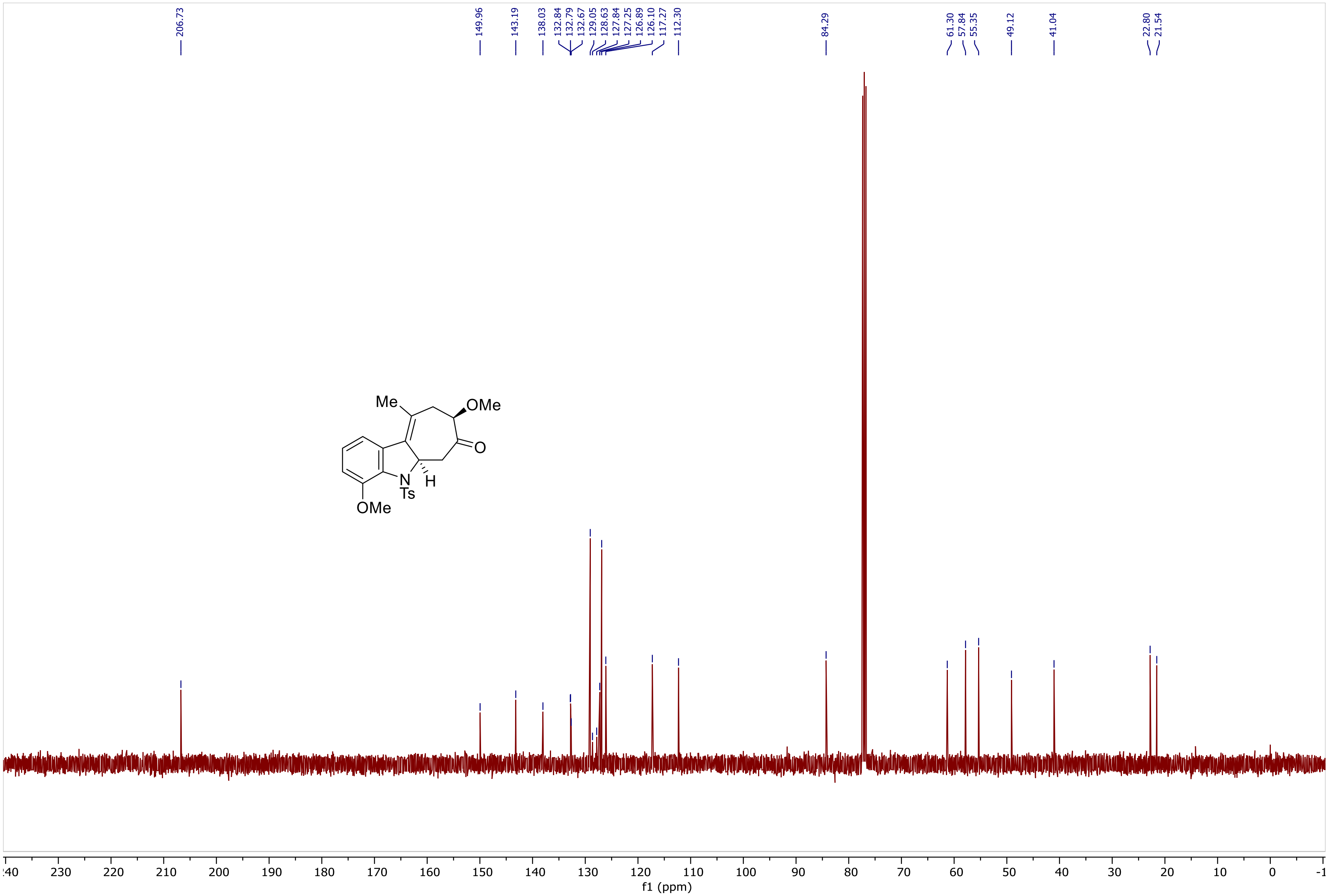

^{13}C NMR (101 MHz, $CDCl_3$) of compound **6i**

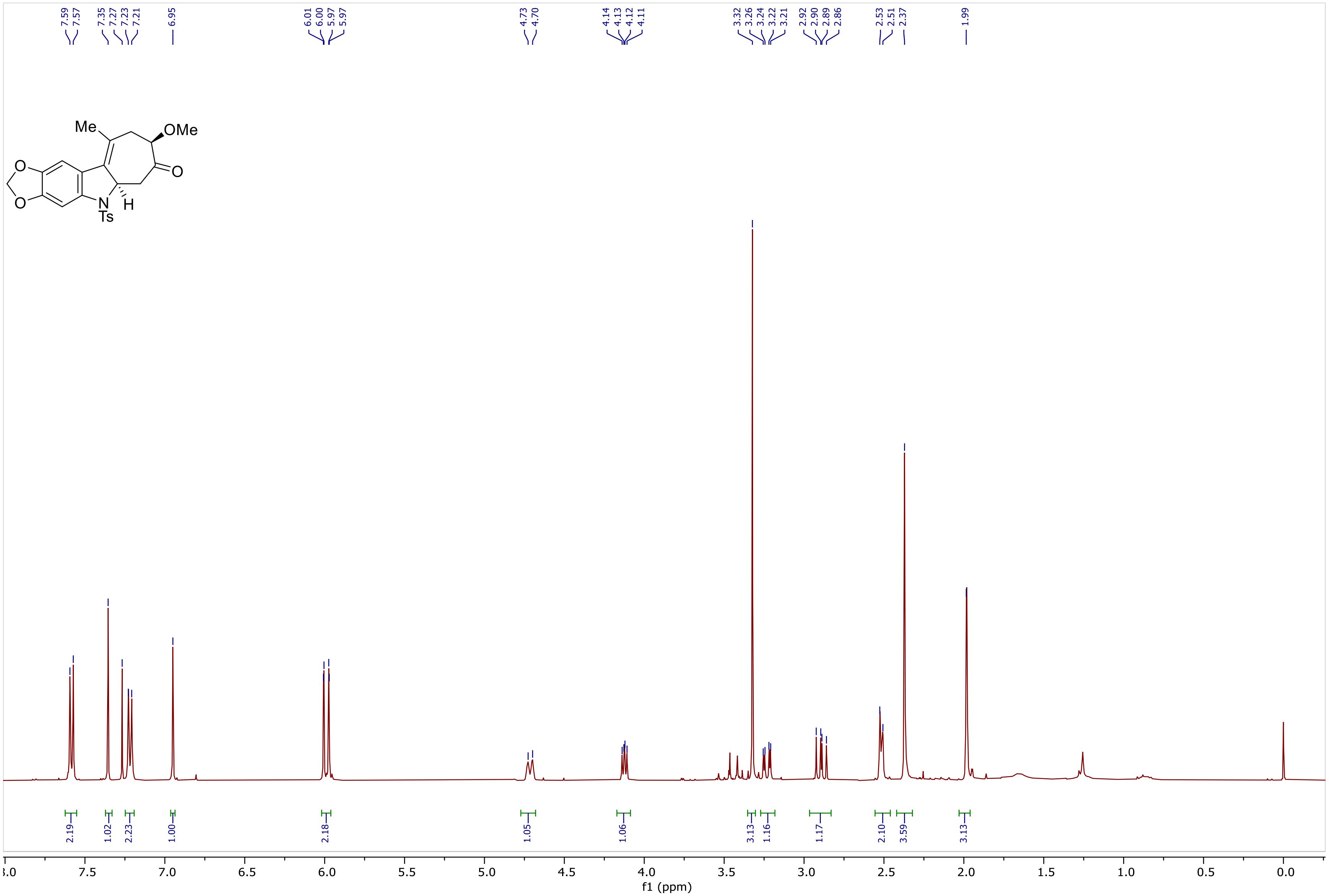

^{1}H NMR (400 MHz, CDCl$_3$) of compound **6j**

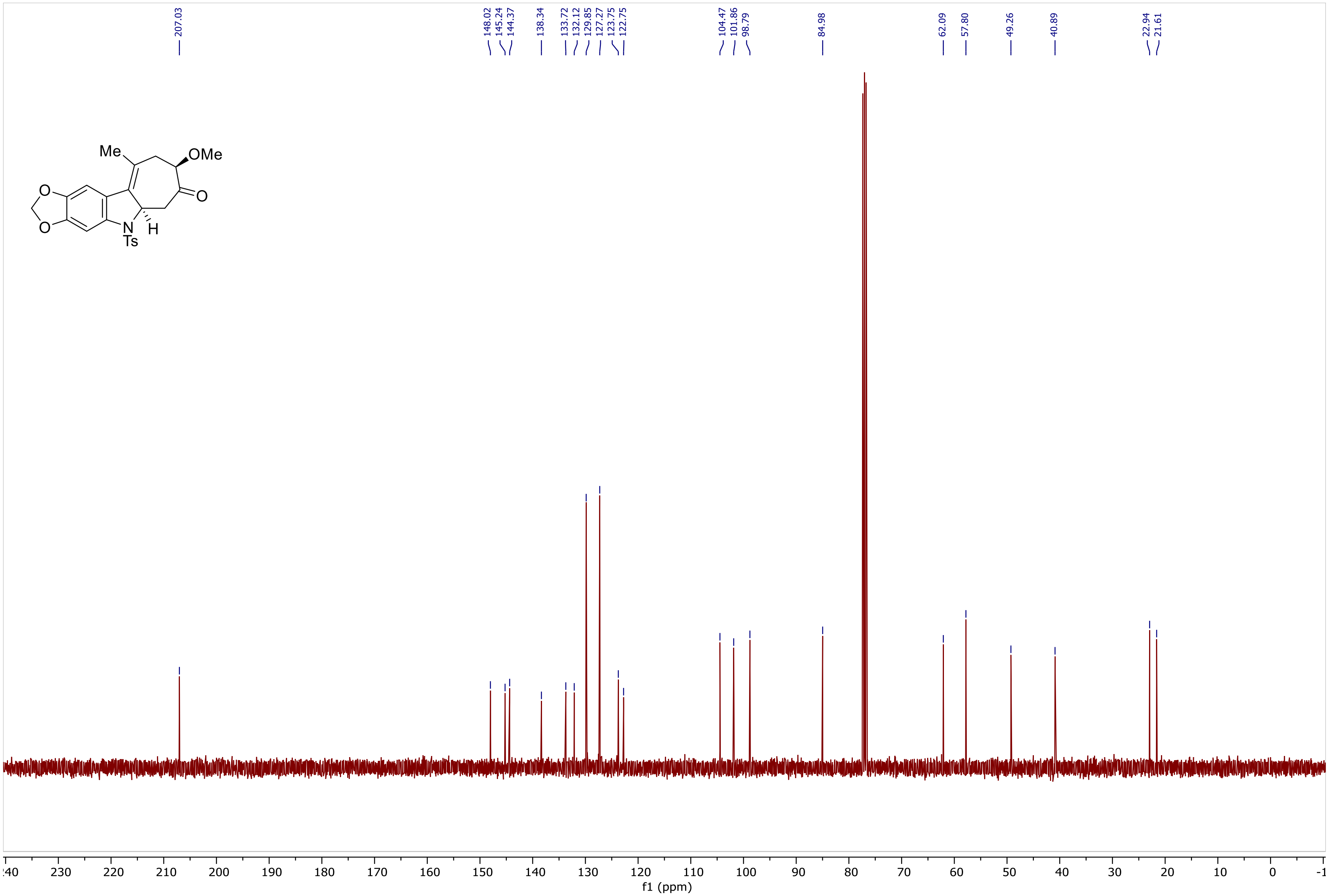

^{13}C NMR (101 MHz, $CDCl_3$) of compound **6j**

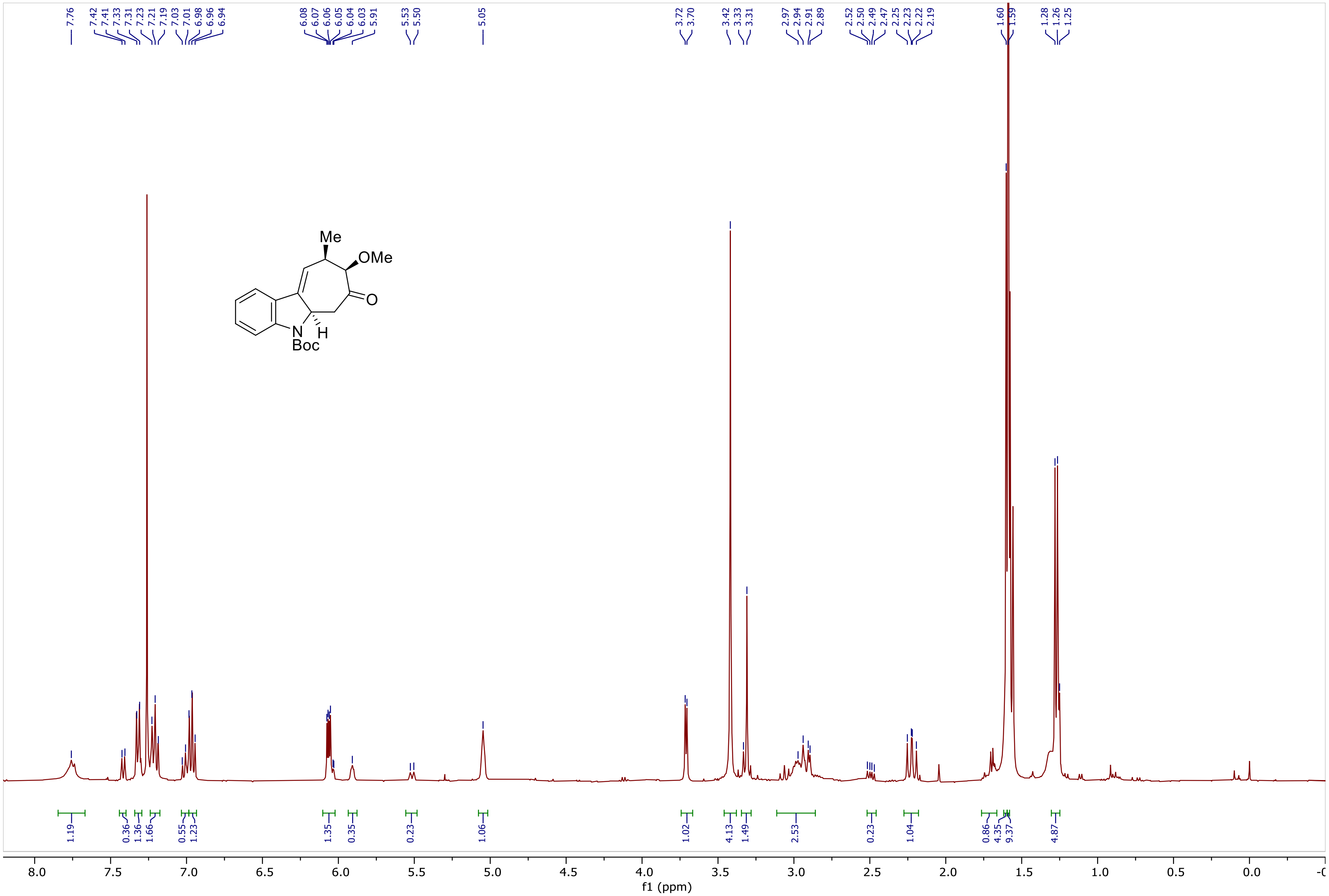

^{1}H NMR (400 MHz, $CDCl_3$) of compound **6k**

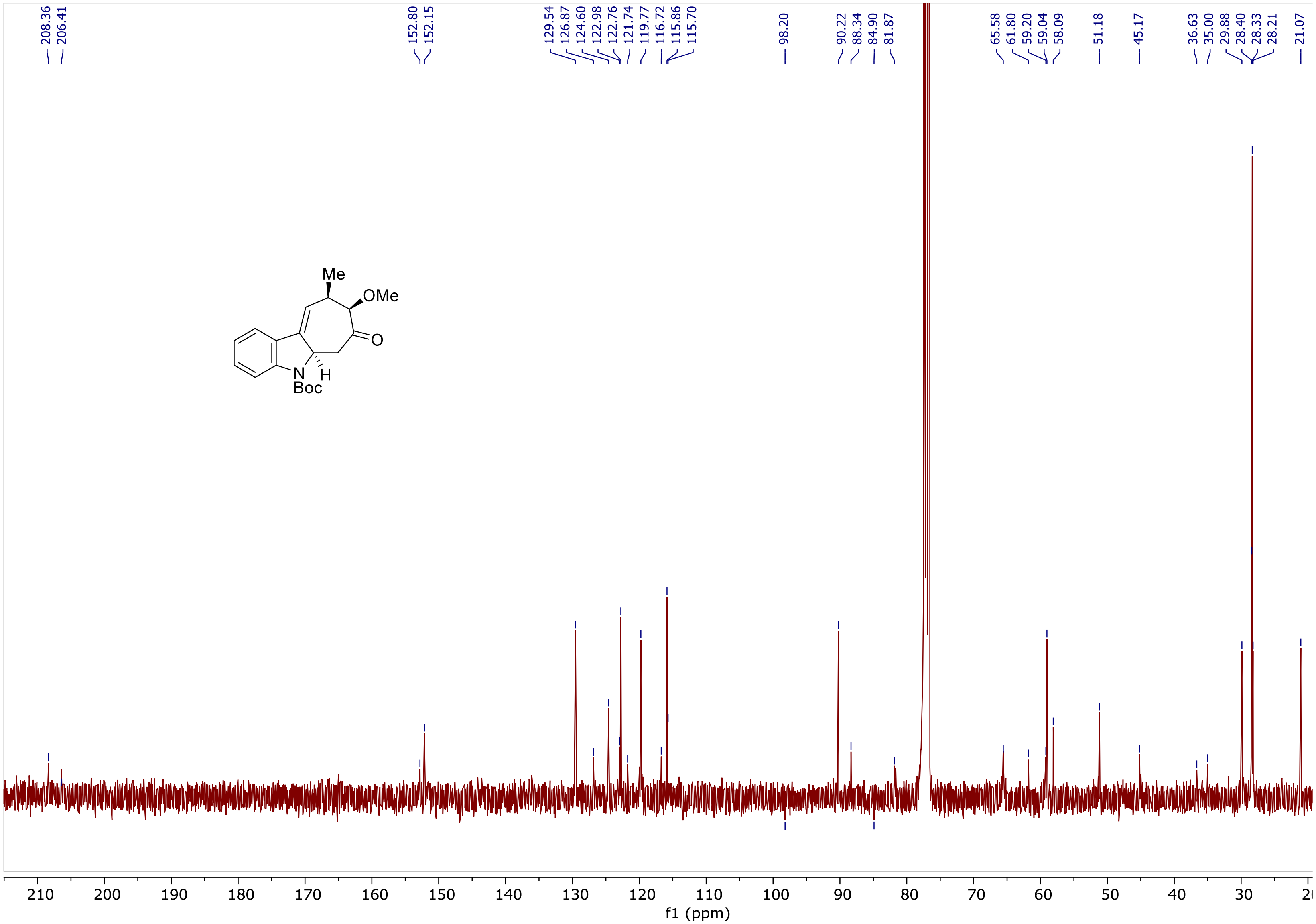

^{13}C NMR (101 MHz, $CDCl_3$) of compound **6k**

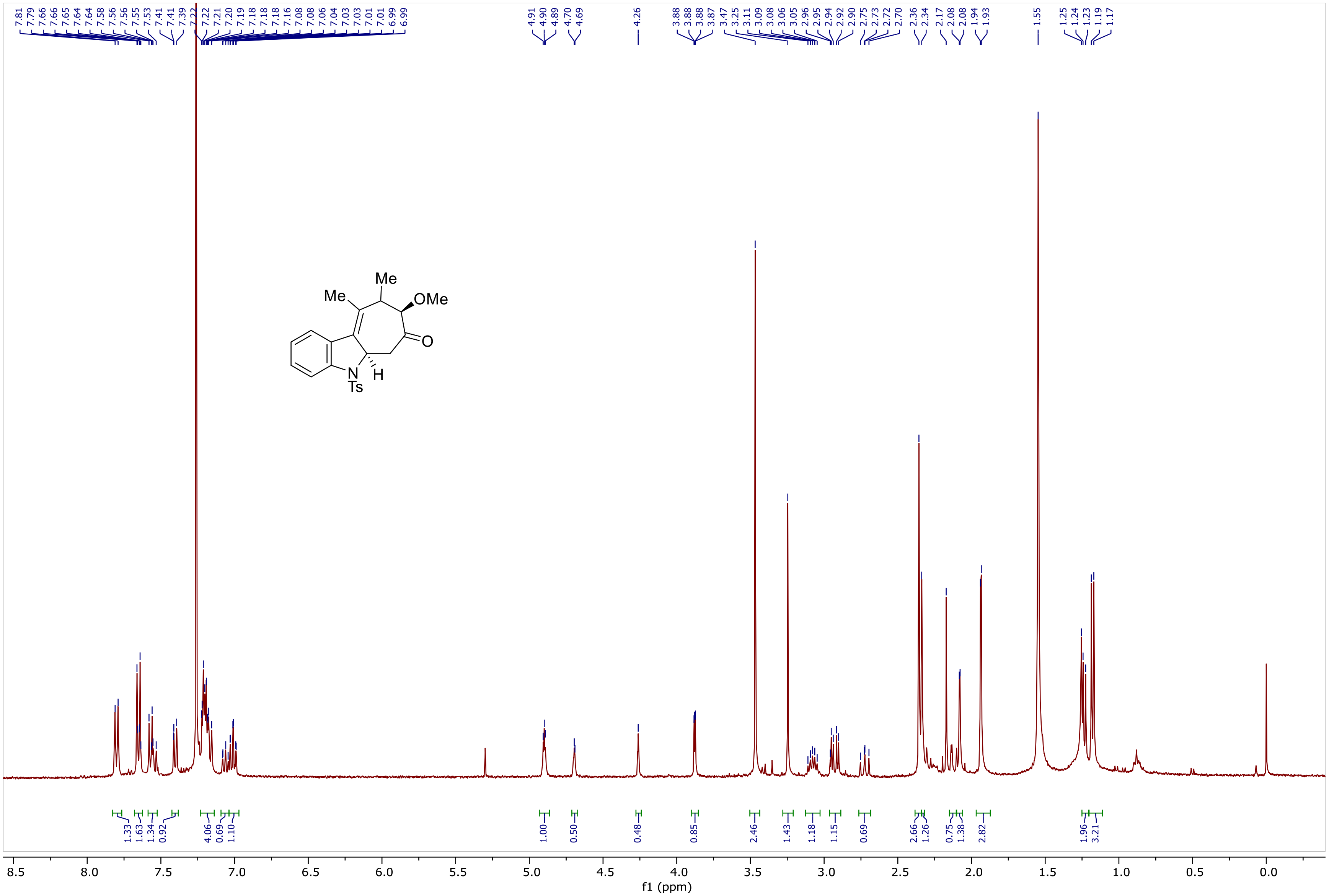

^1H NMR (400 MHz, $CDCl_3$) of compound **6l**

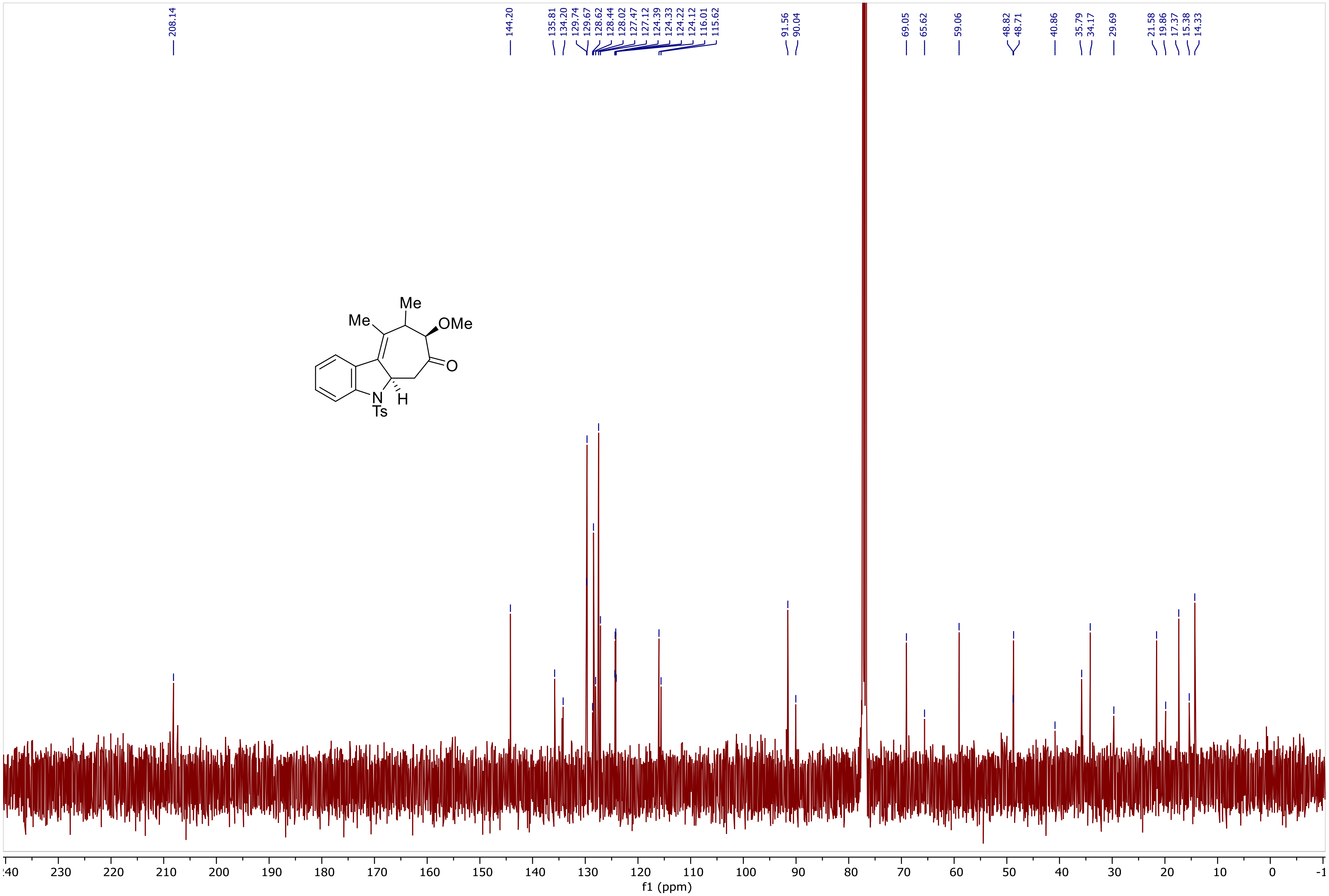

^{13}C NMR (101 MHz, $CDCl_3$) of compound **6l**

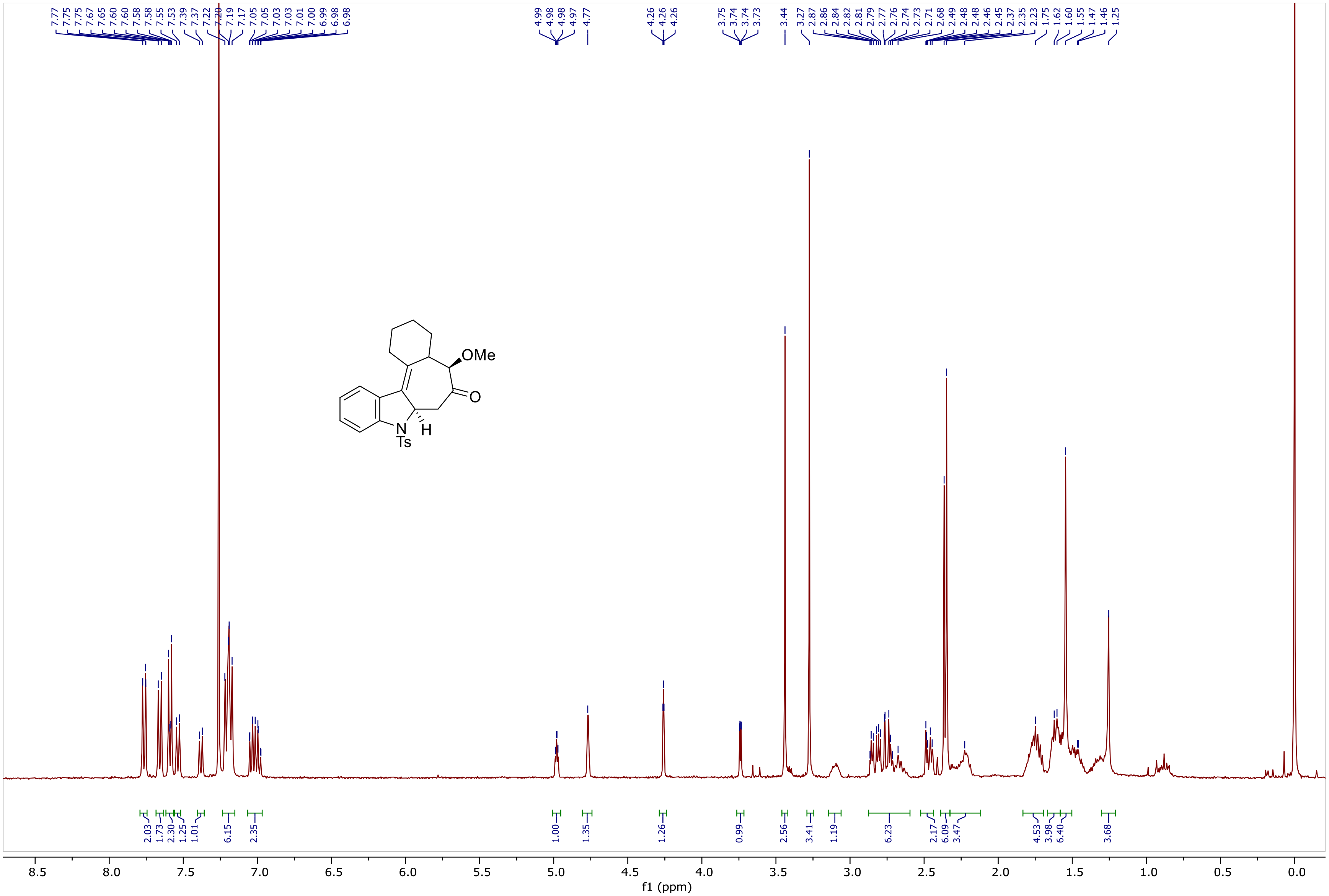

^{1}H NMR (400 MHz, $CDCl_3$) of compound **6m**

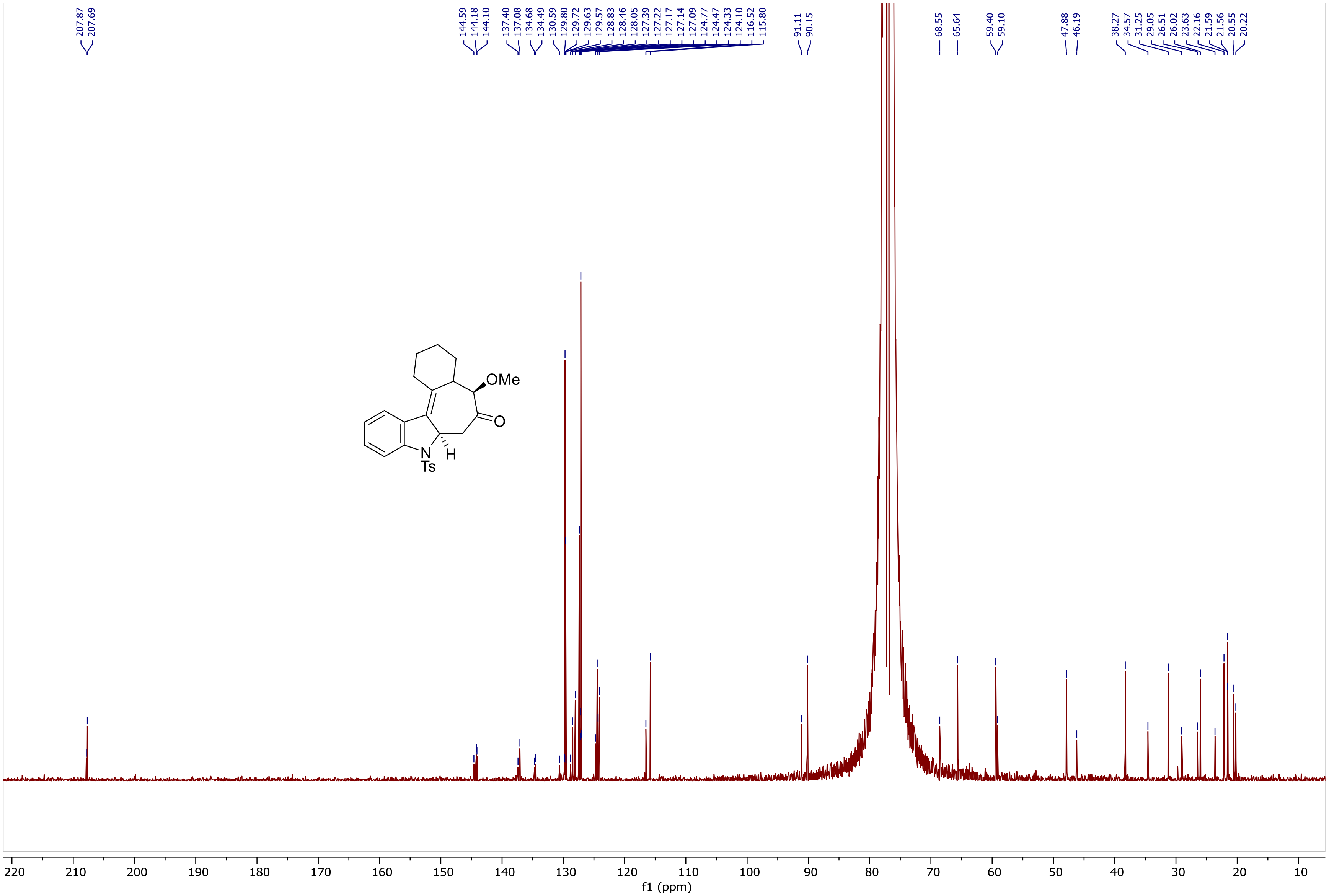

^{13}C NMR (101 MHz, $CDCl_3$) of compound **6m**

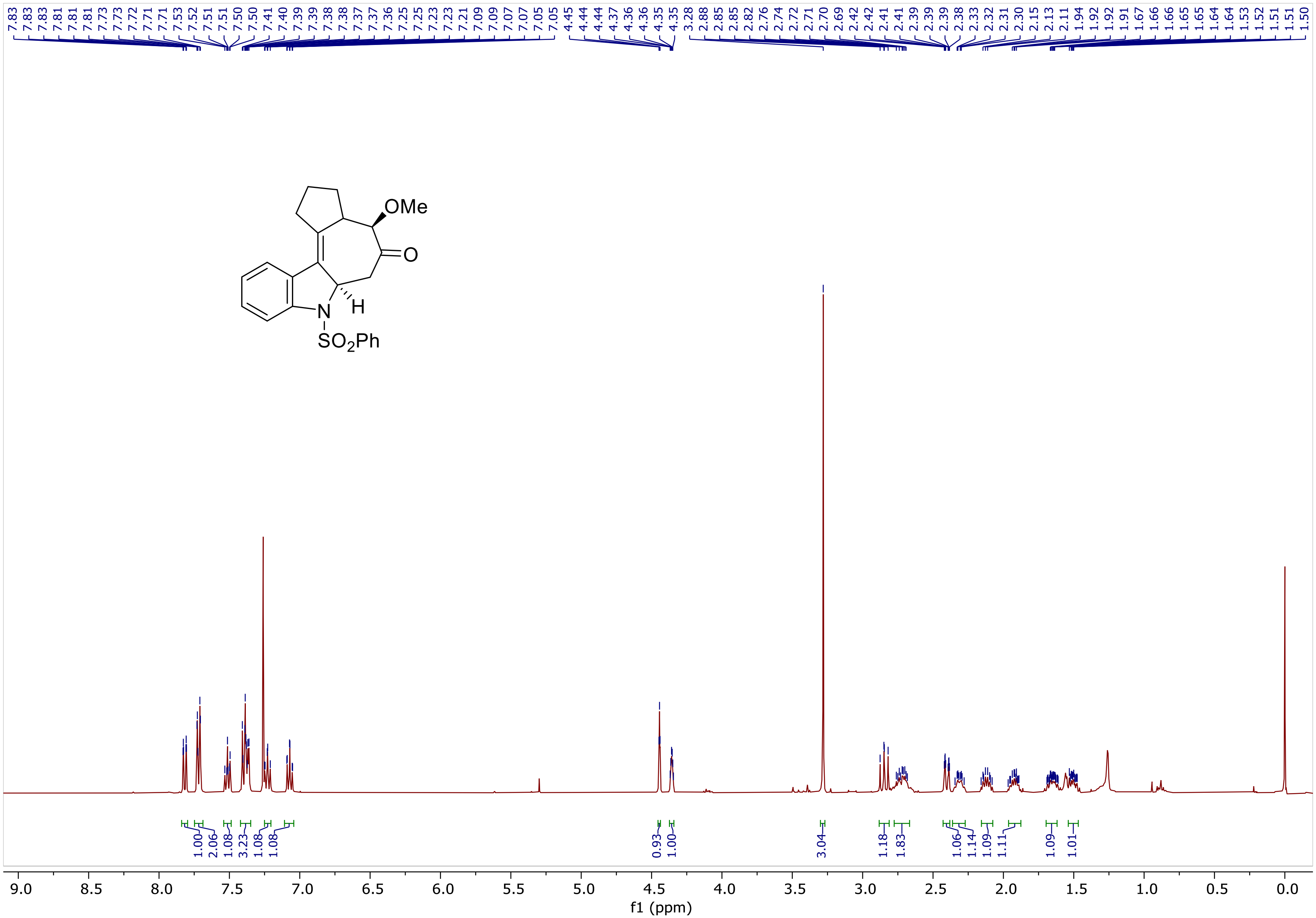

^{1}H NMR (400 MHz, $CDCl_3$) of compound **6n**

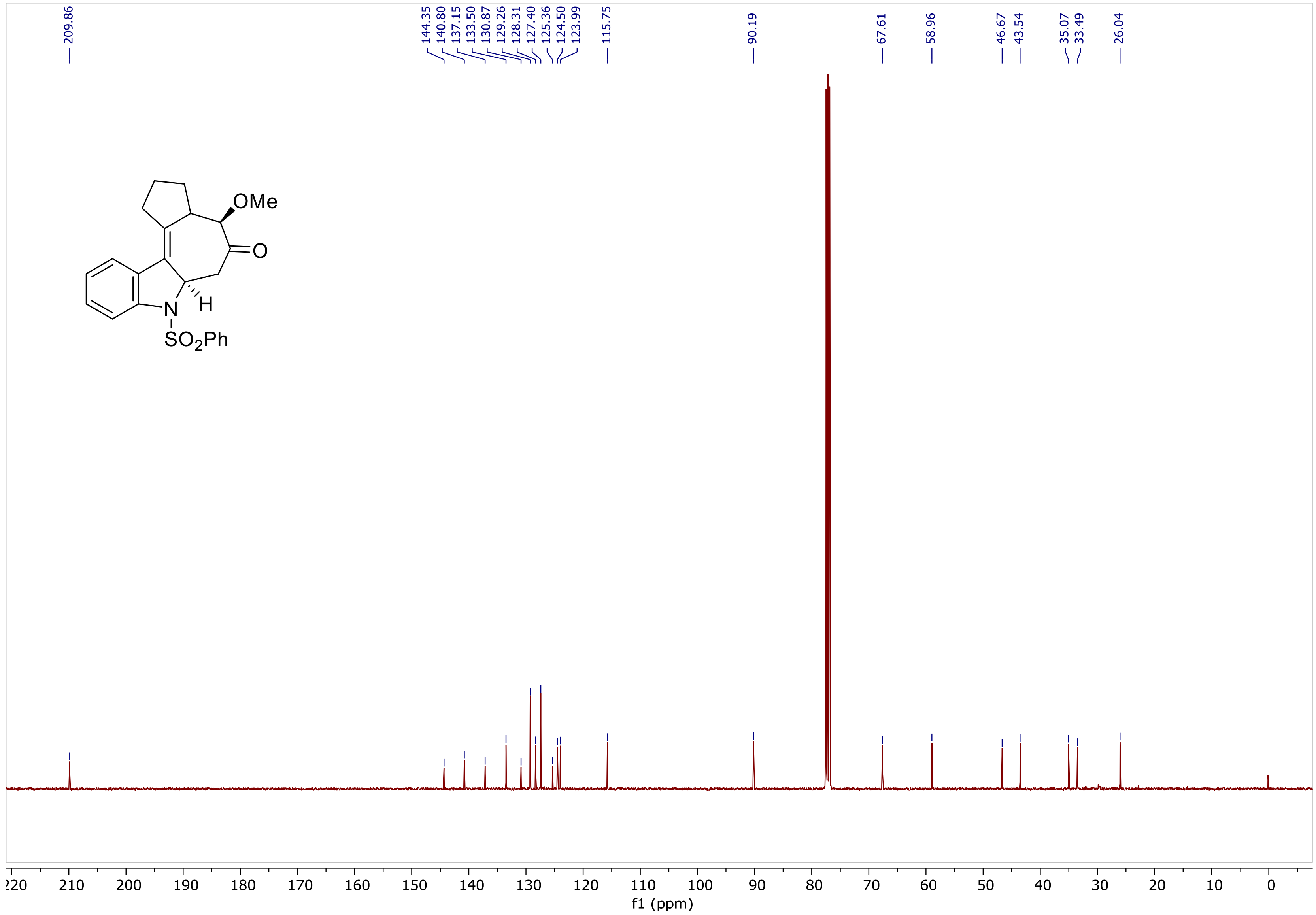

^{13}C NMR (101 MHz, $CDCl_3$) of compound **6n**

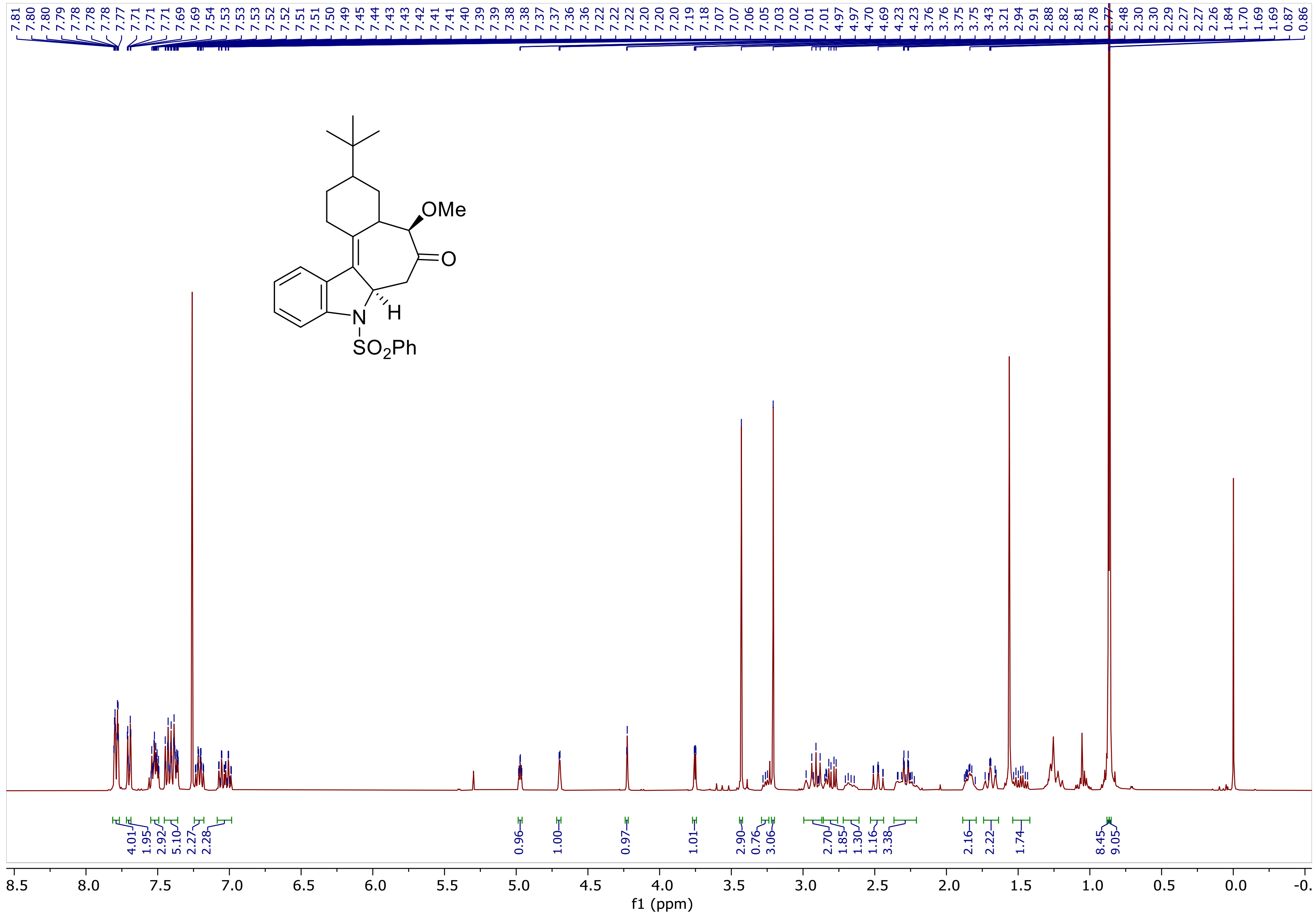

^{1}H NMR (400 MHz, $CDCl_3$) of compound **6o**

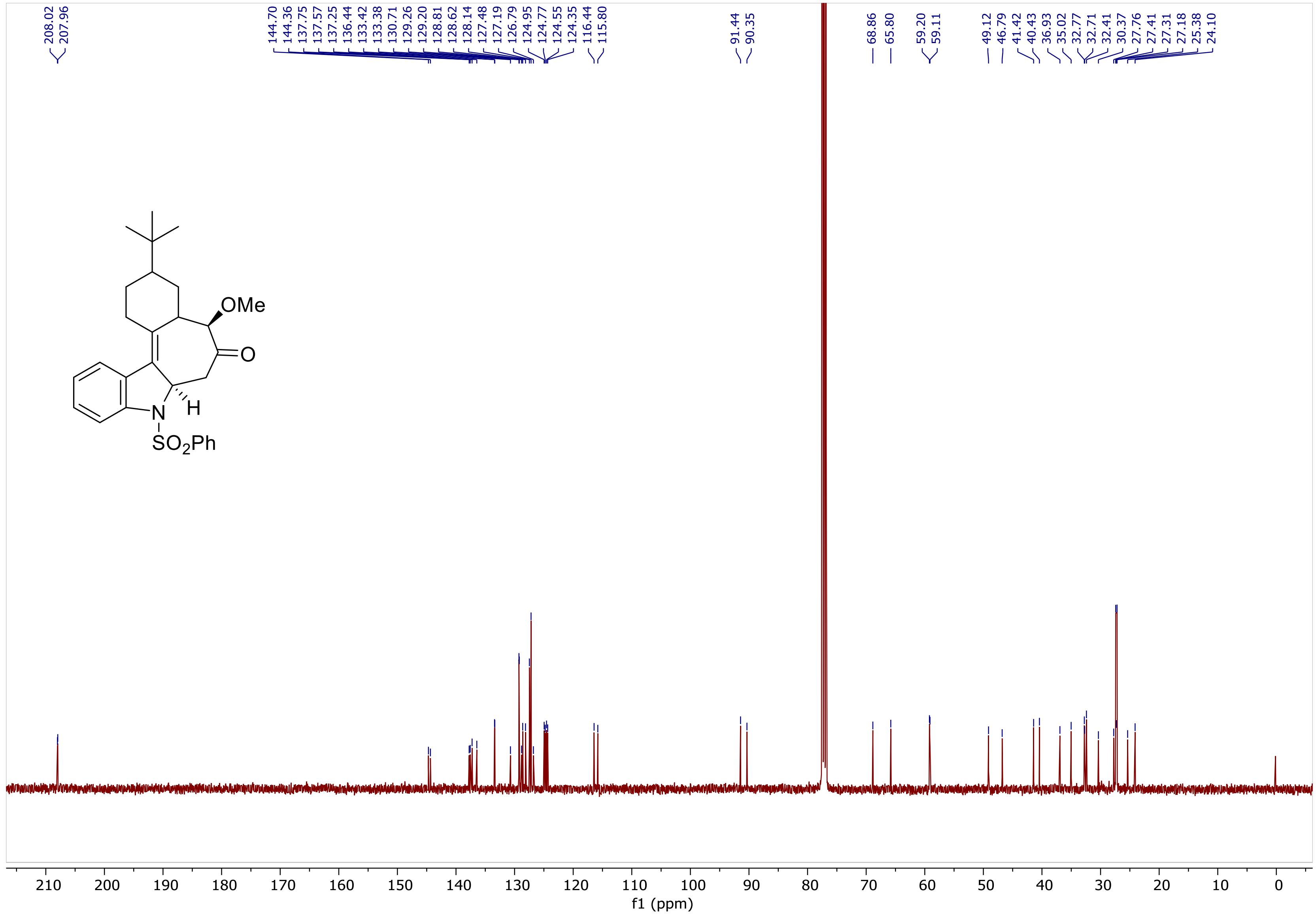

^{13}C NMR (101 MHz, $CDCl_3$) of compound **6o**

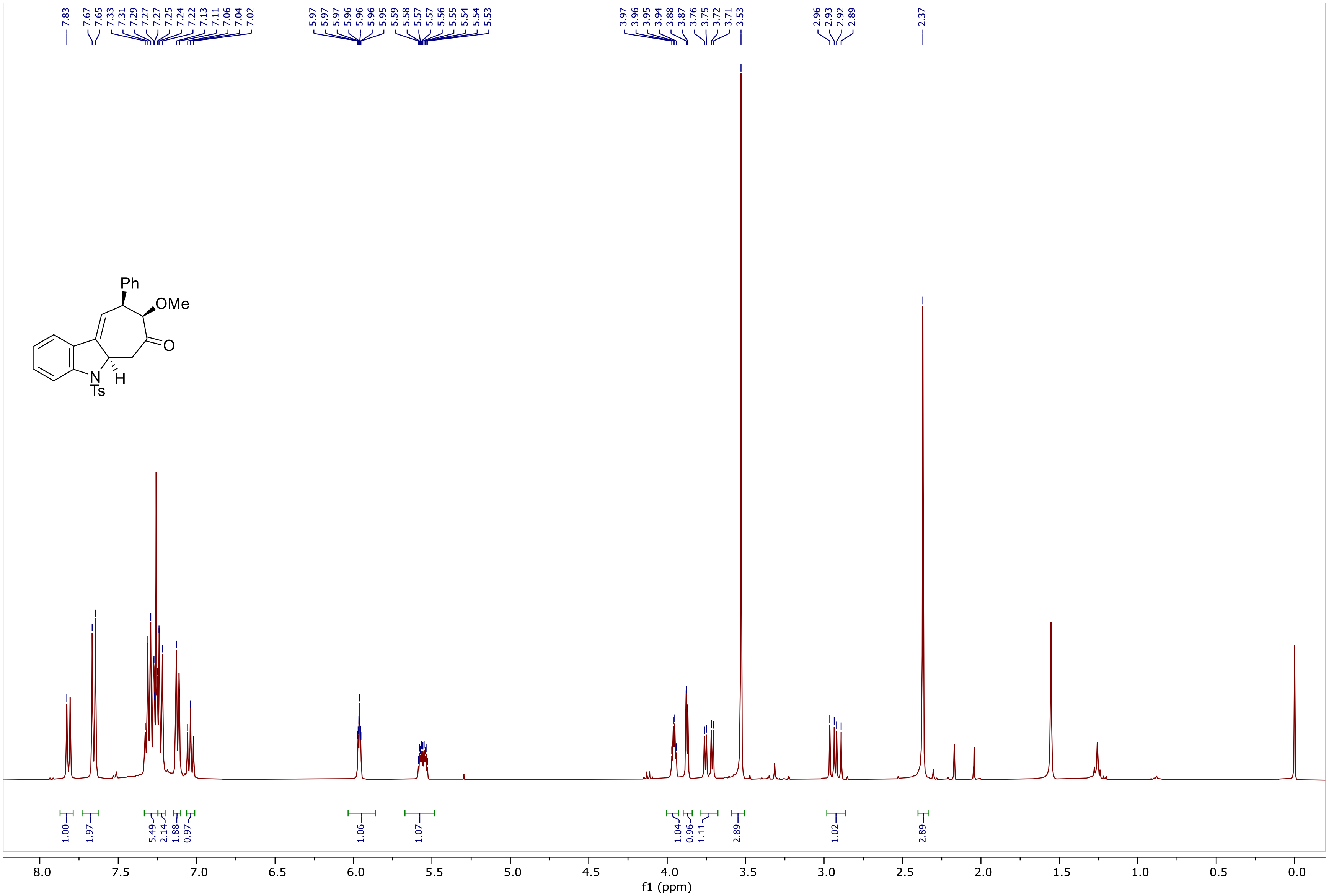

^{1}H NMR (400 MHz, CDCl$_3$) of compound **6p**

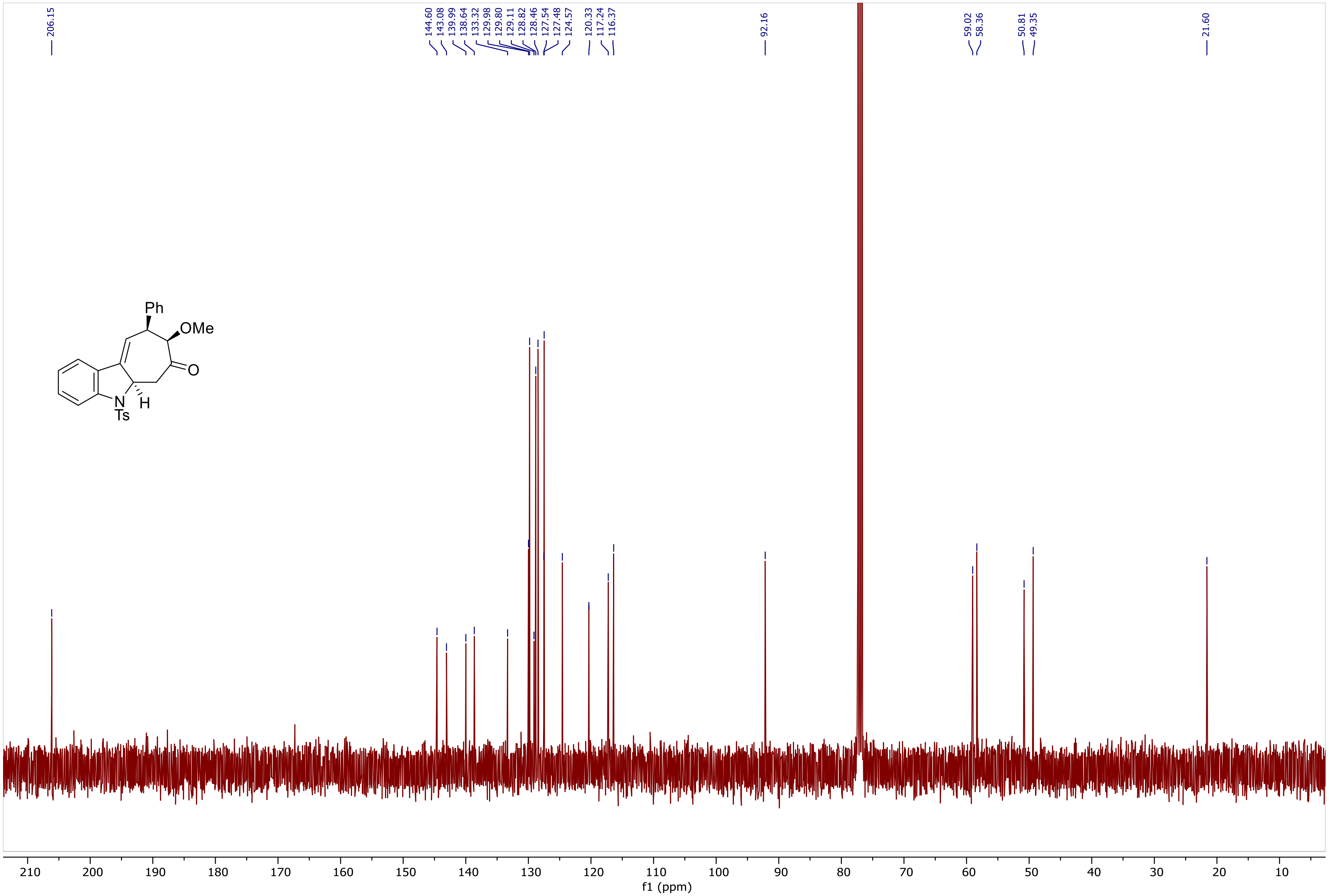

^{13}C NMR (101 MHz, $CDCl_3$) of compound **6p**

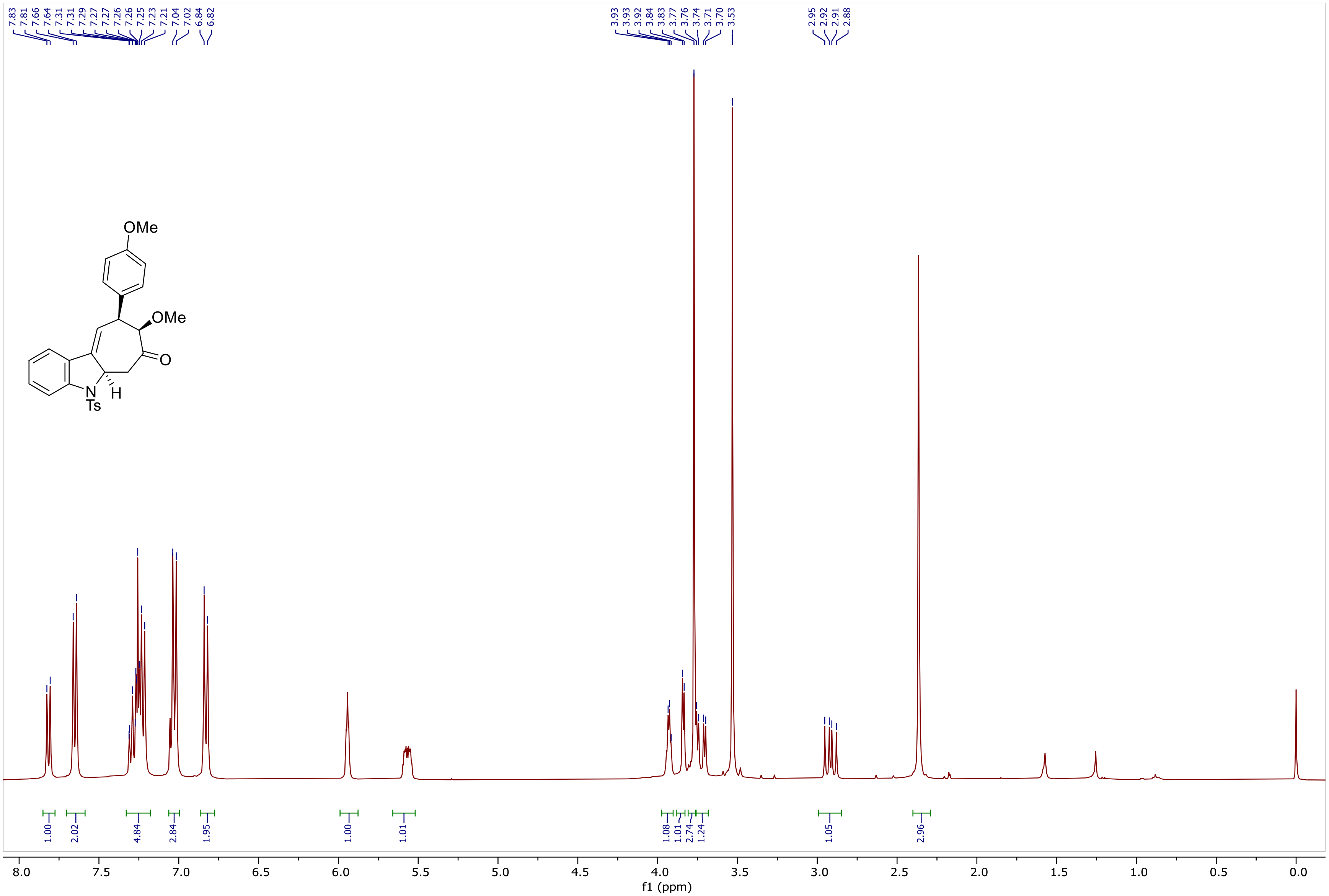

^{1}H NMR (400 MHz, $CDCl_3$) of compound **6q**

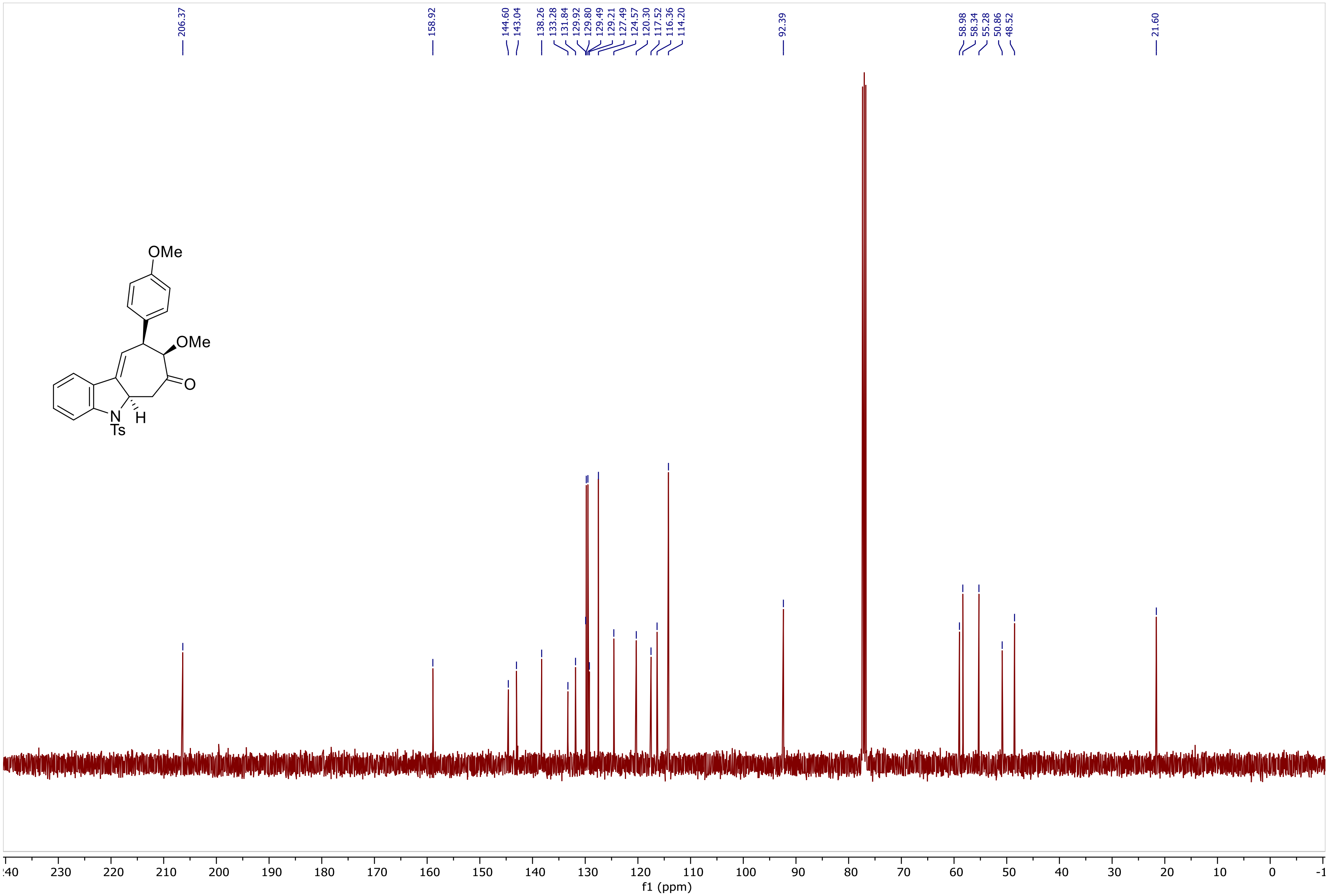

^{13}C NMR (101 MHz, $CDCl_3$) of compound **6q**

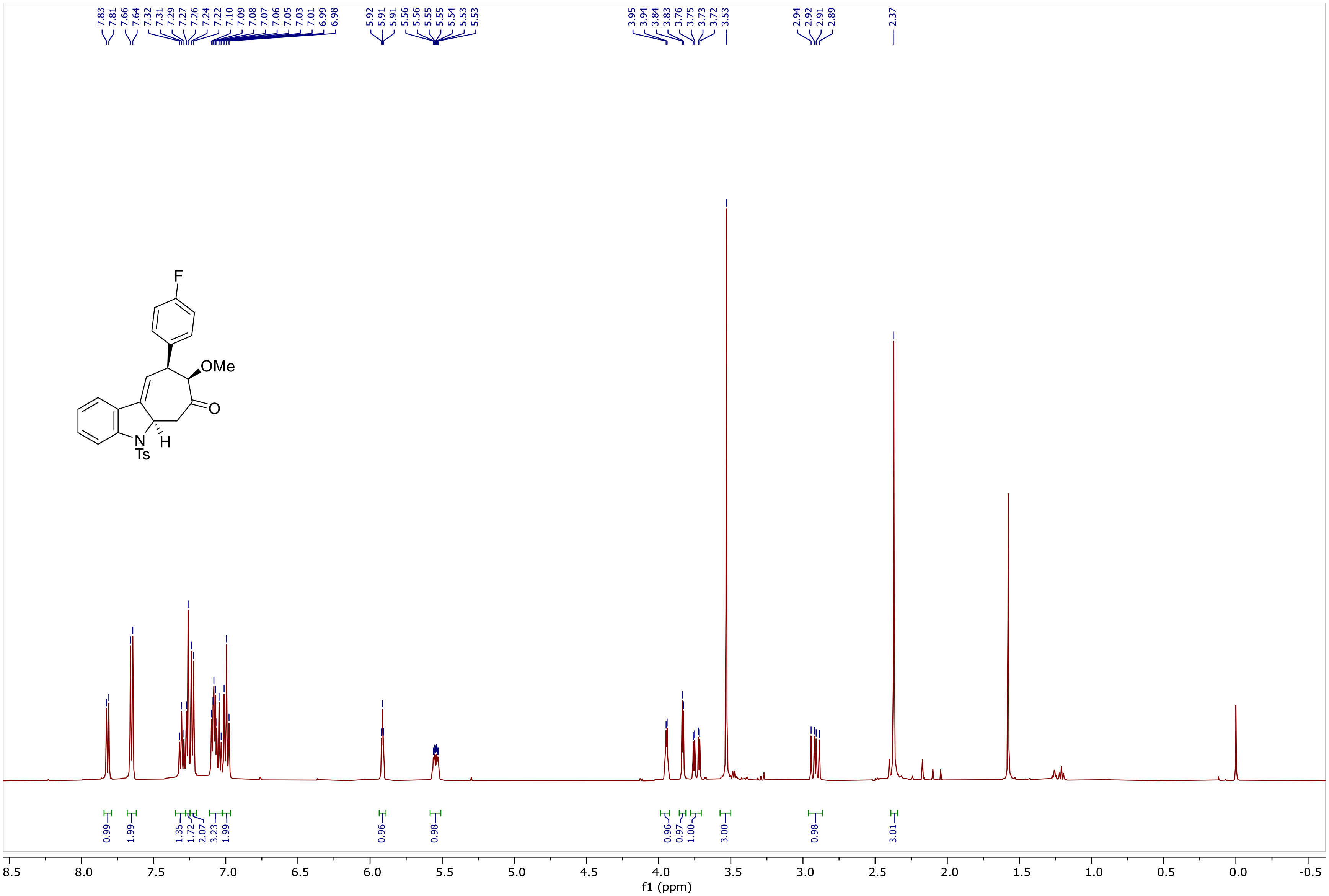

^{1}H NMR (400 MHz, $CDCl_3$) of compound **6r**

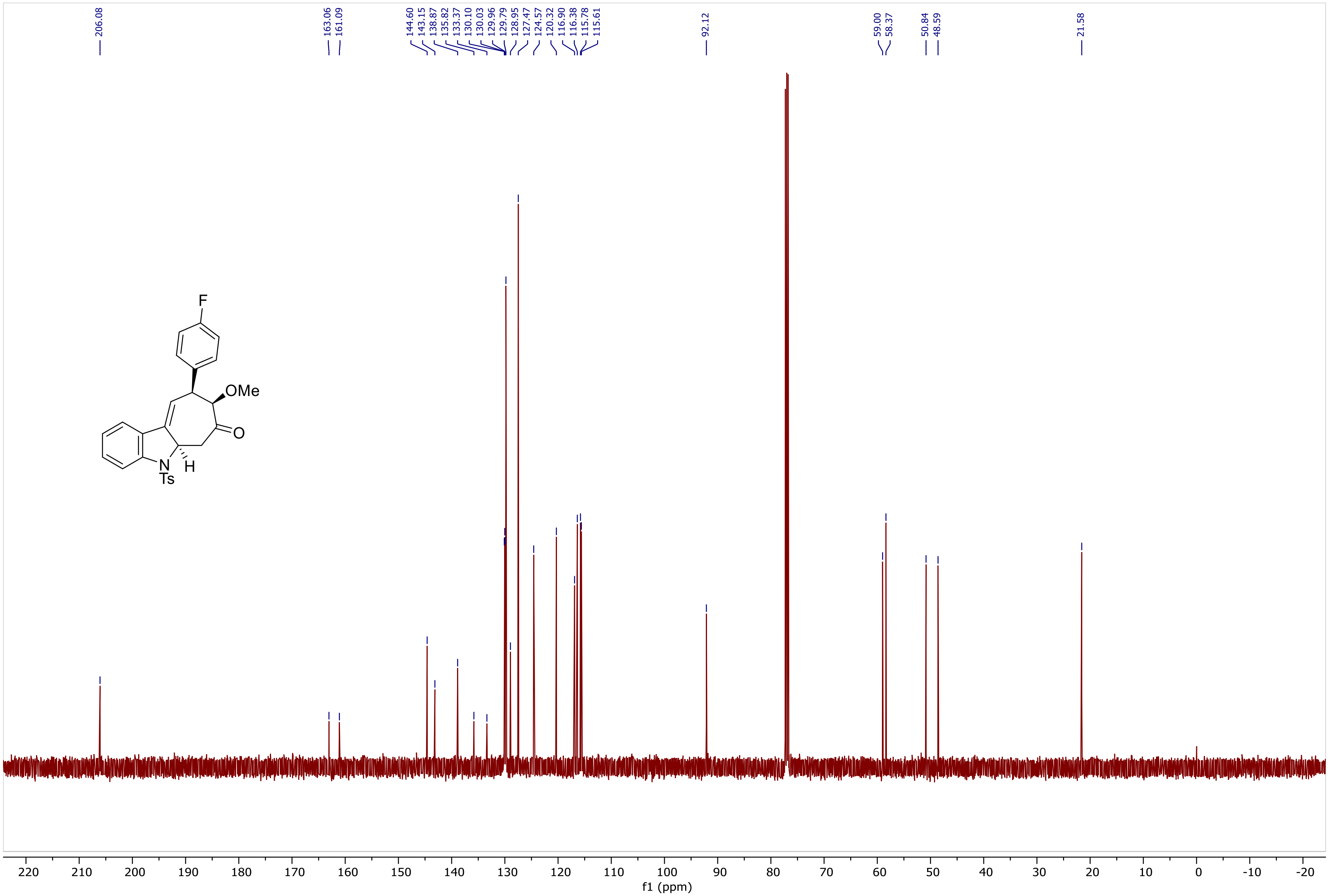

^{13}C NMR (101 MHz, $CDCl_3$) of compound **6r**

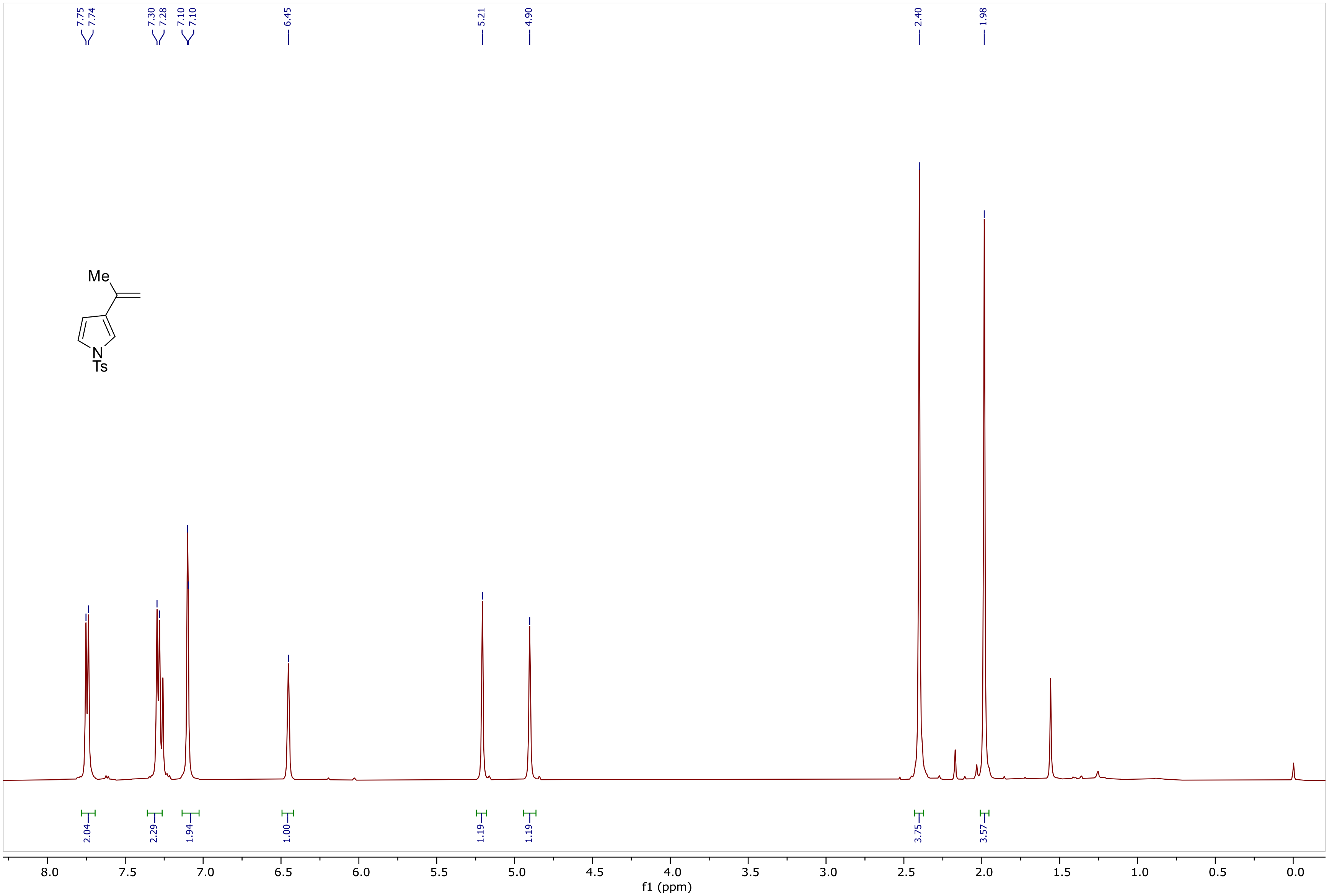

^{1}H NMR (400 MHz, $CDCl_3$) of compound **7a**

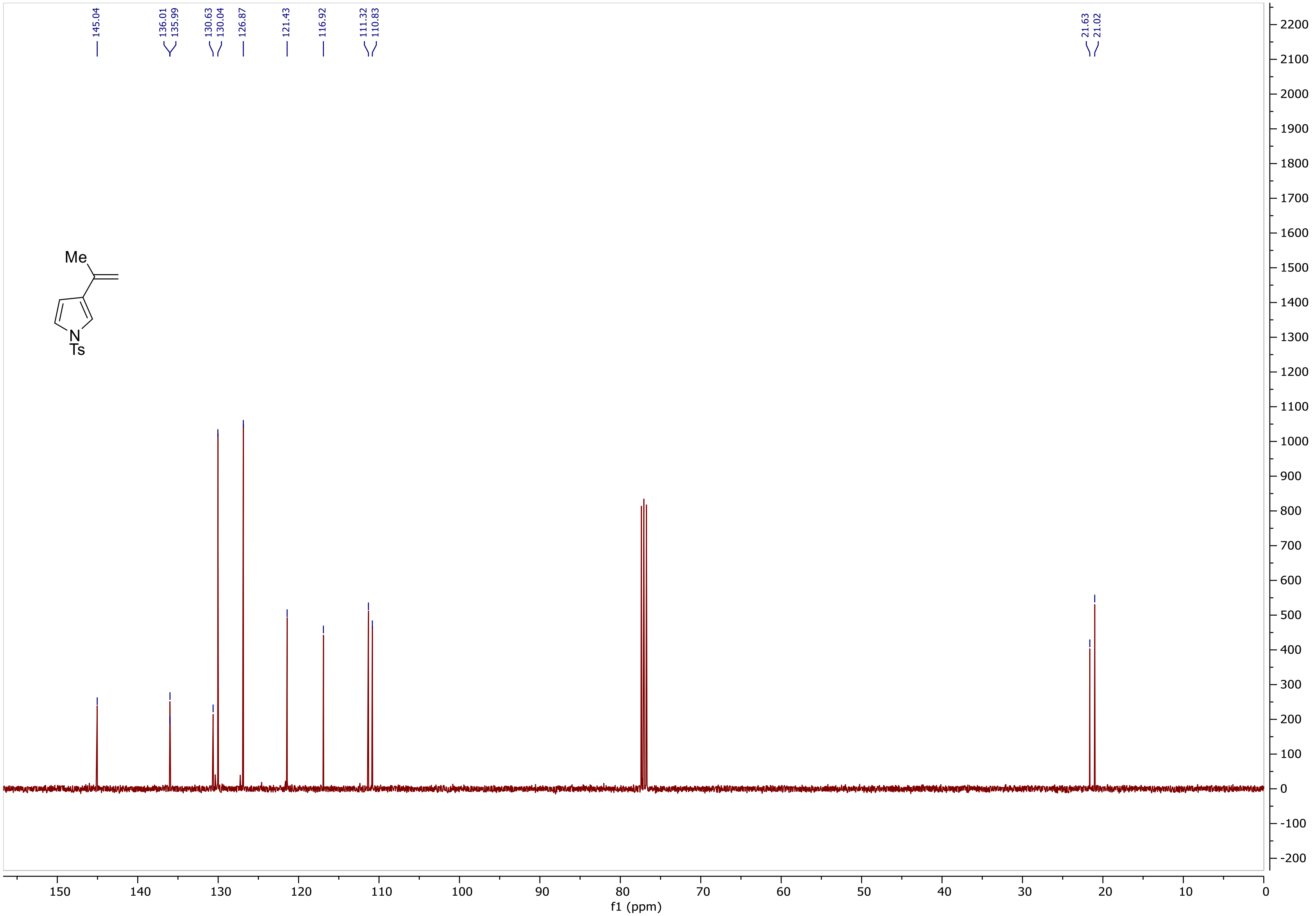

^{13}C NMR (101 MHz, $CDCl_3$) of compound **7a**

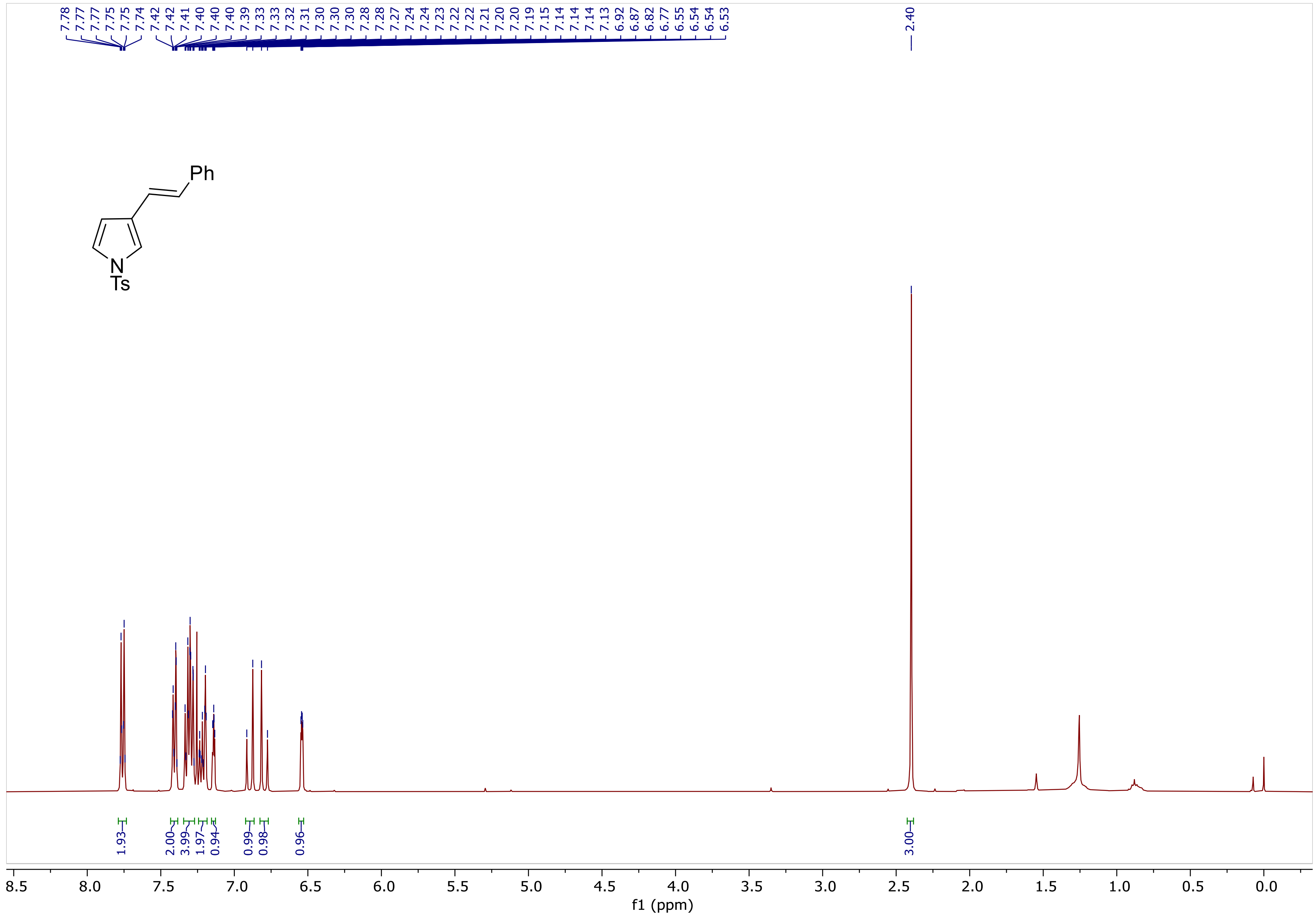

^{1}H NMR (400 MHz, $CDCl_3$) of compound **7b**

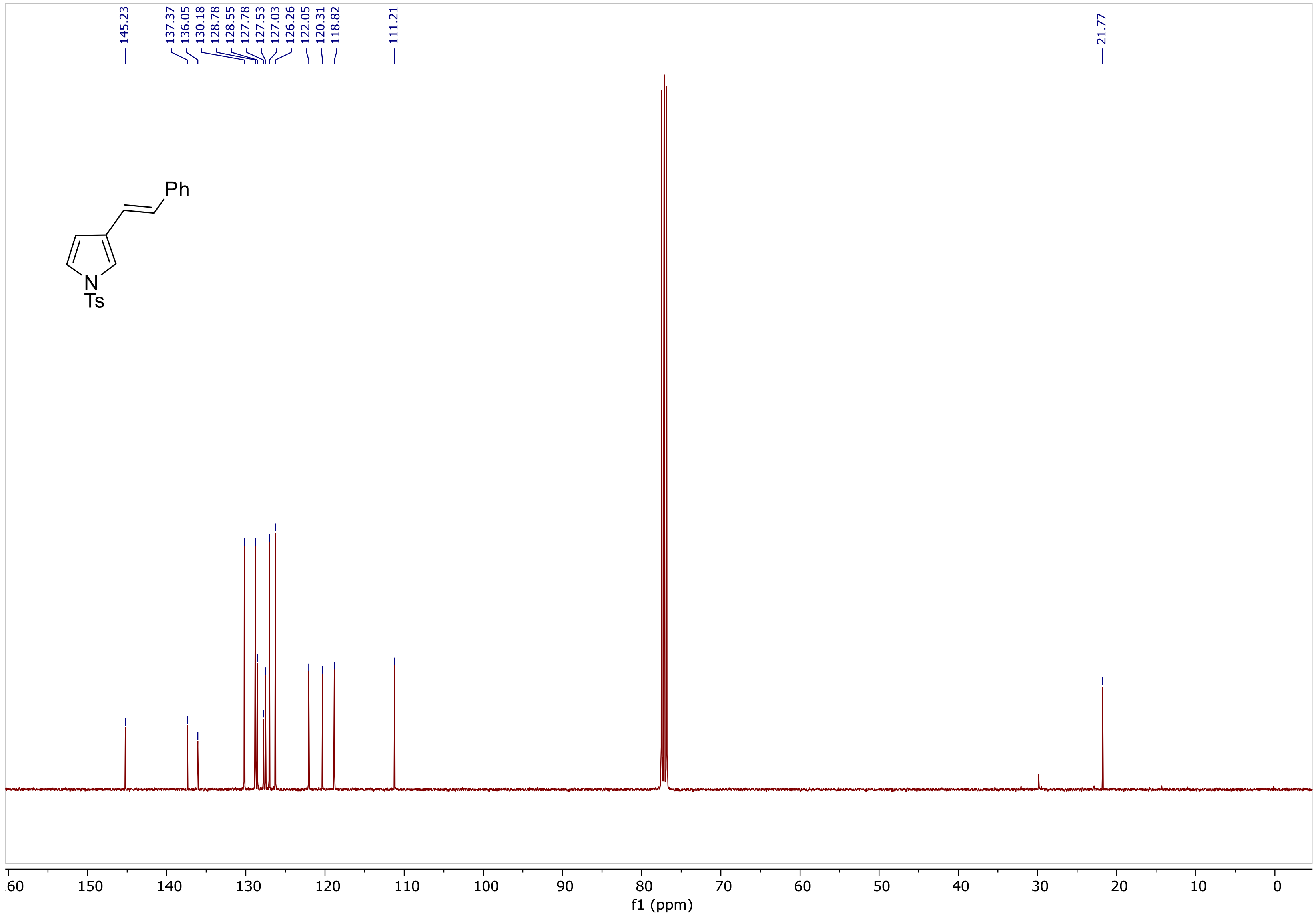

^{13}C NMR (101 MHz, $CDCl_3$) of compound **7b**

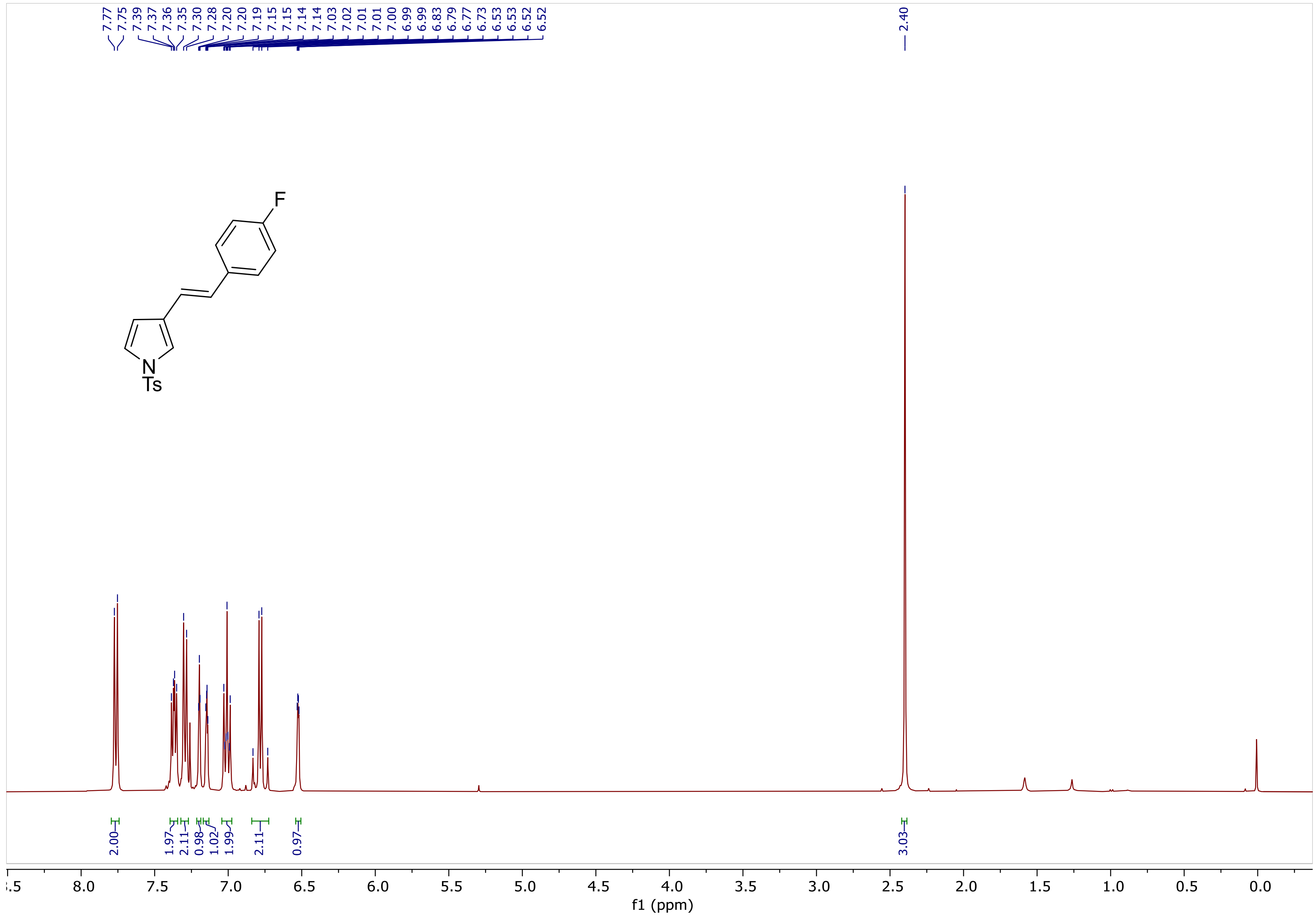

^{1}H NMR (400 MHz, $CDCl_3$) of compound **7c**

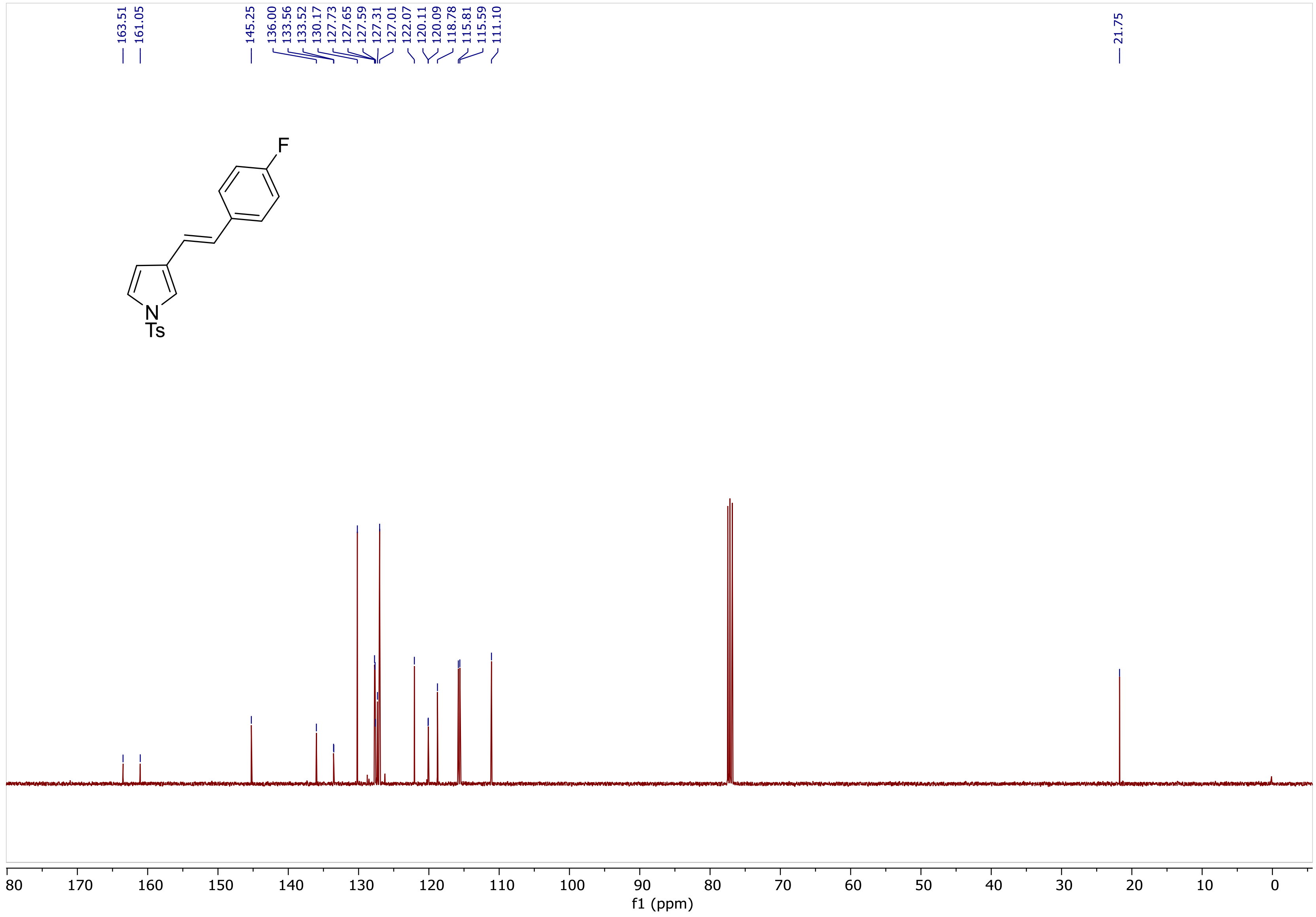

^{13}C NMR (101 MHz, $CDCl_3$) of compound **7c**

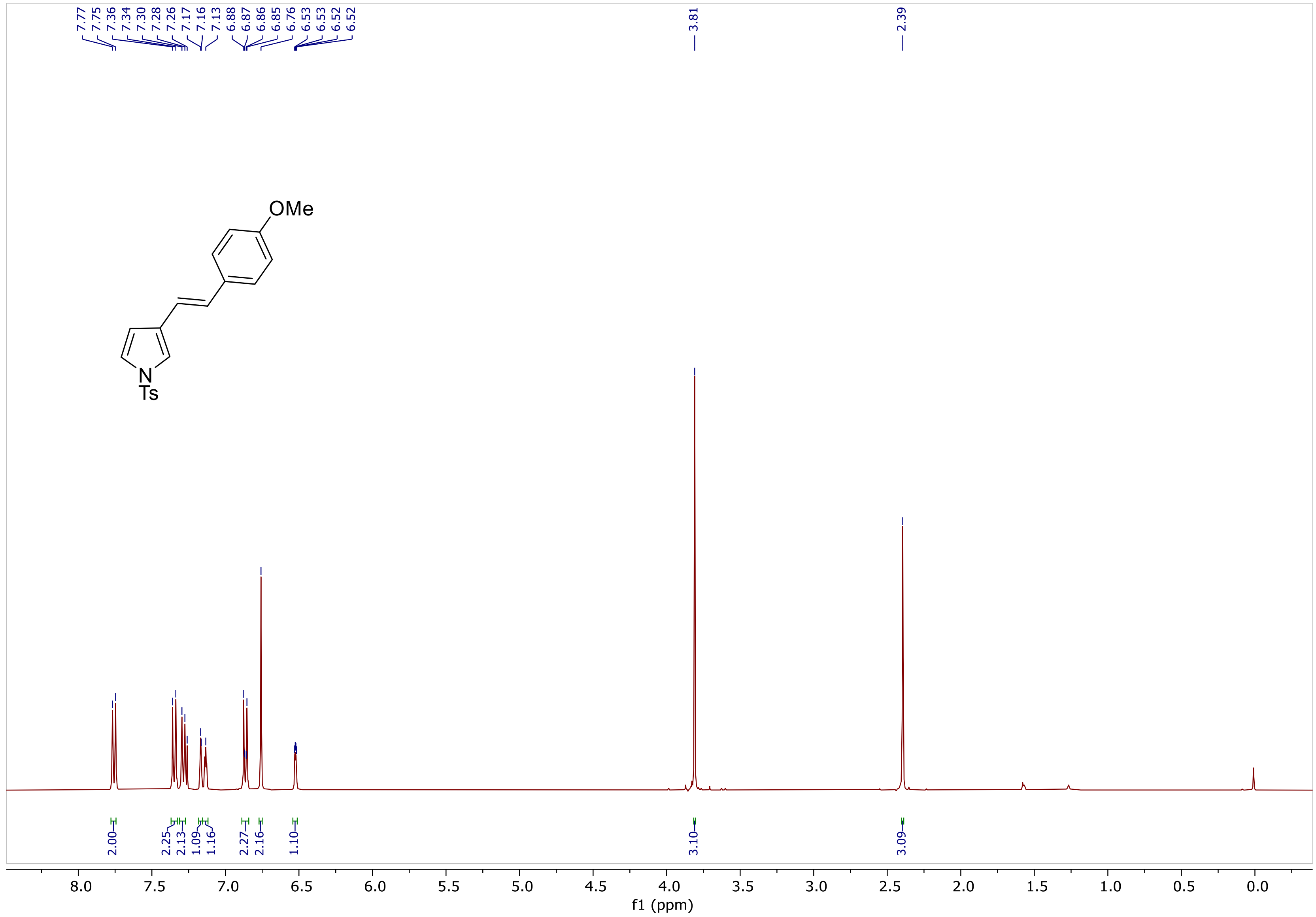

^{1}H NMR (400 MHz, $CDCl_3$) of compound **7d**

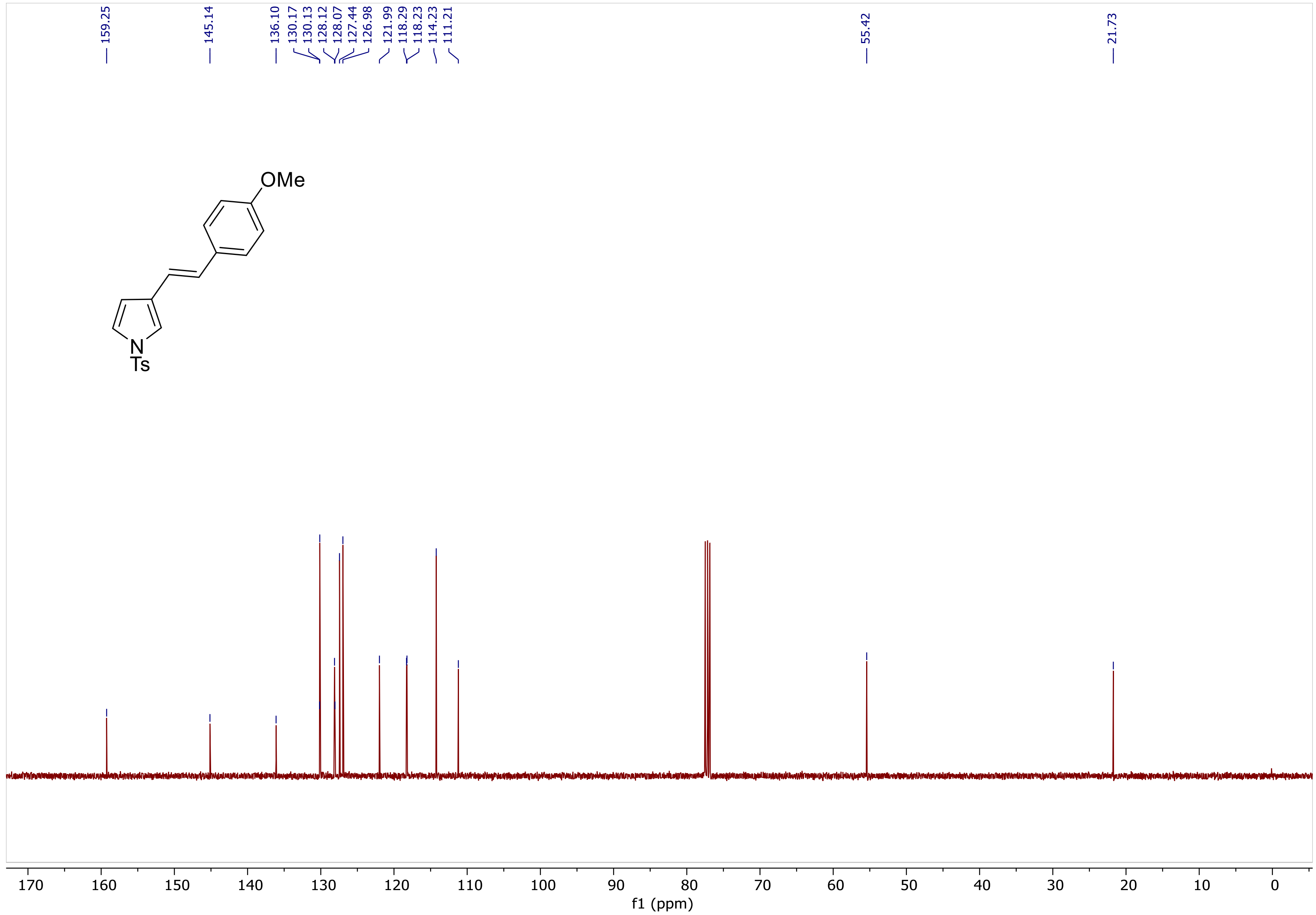

^{13}C NMR (101 MHz, $CDCl_3$) of compound **7d**

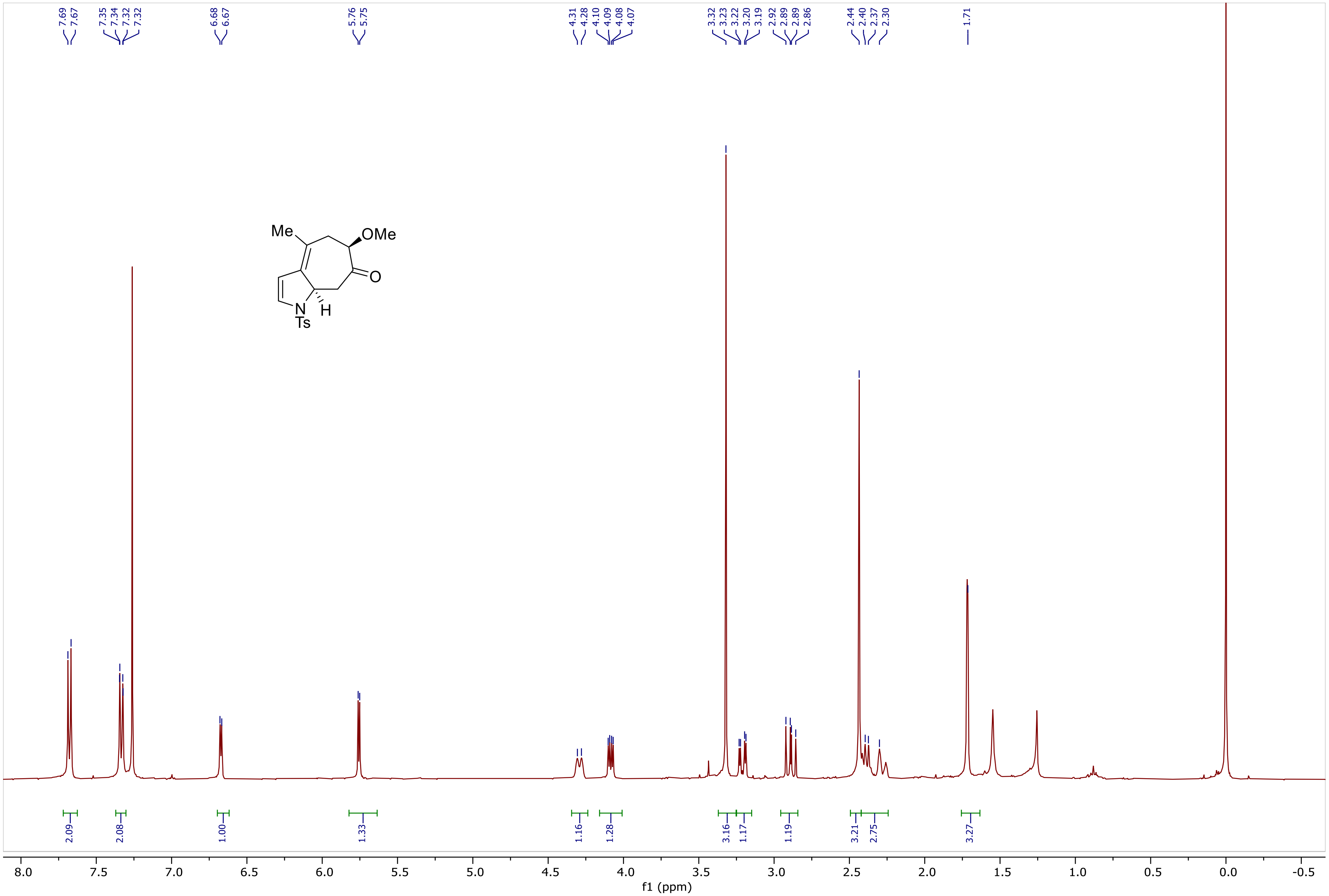

^{1}H NMR (400 MHz, $CDCl_3$) of compound **8a**

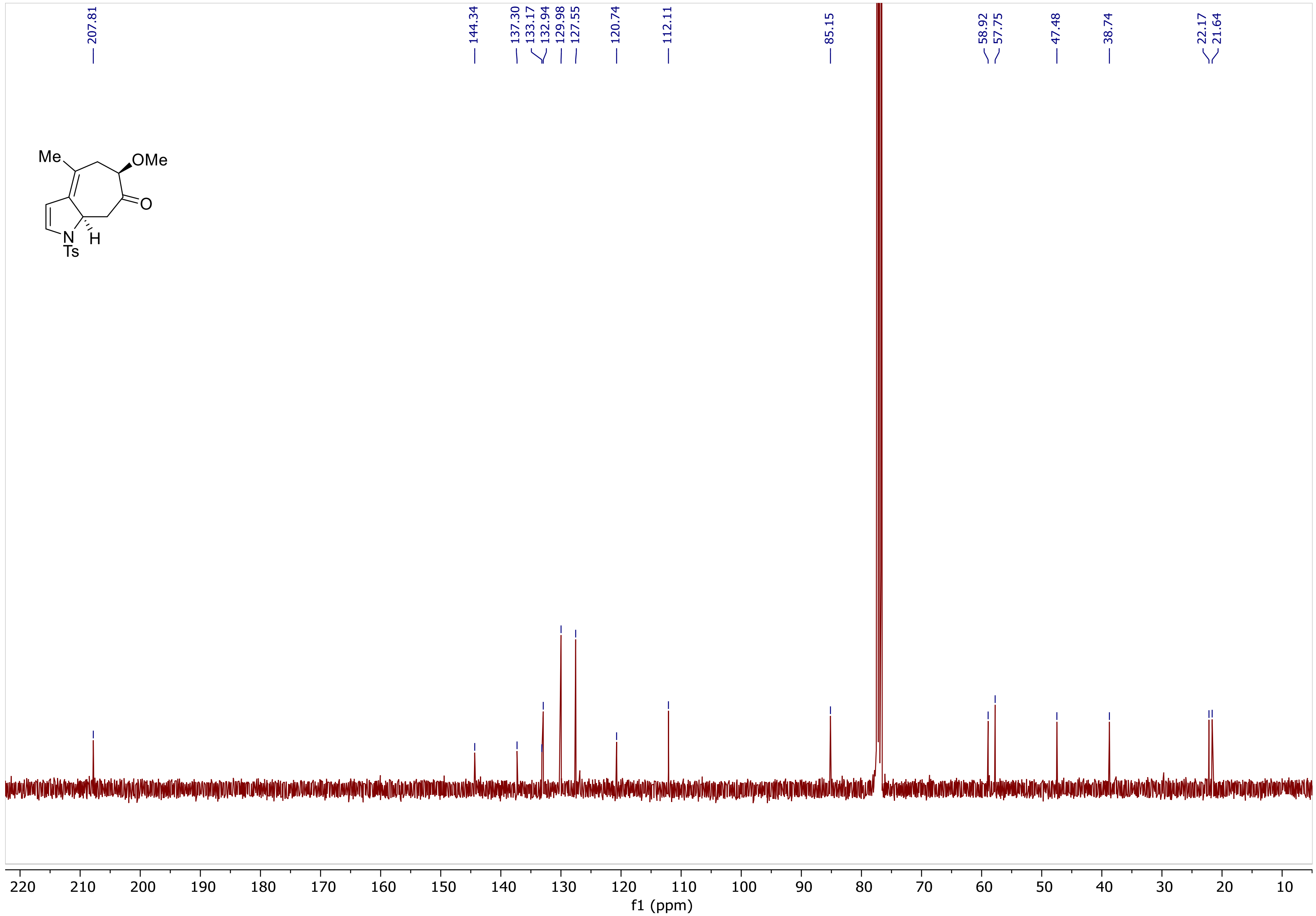

^{13}C NMR (101 MHz, $CDCl_3$) of compound **8a**

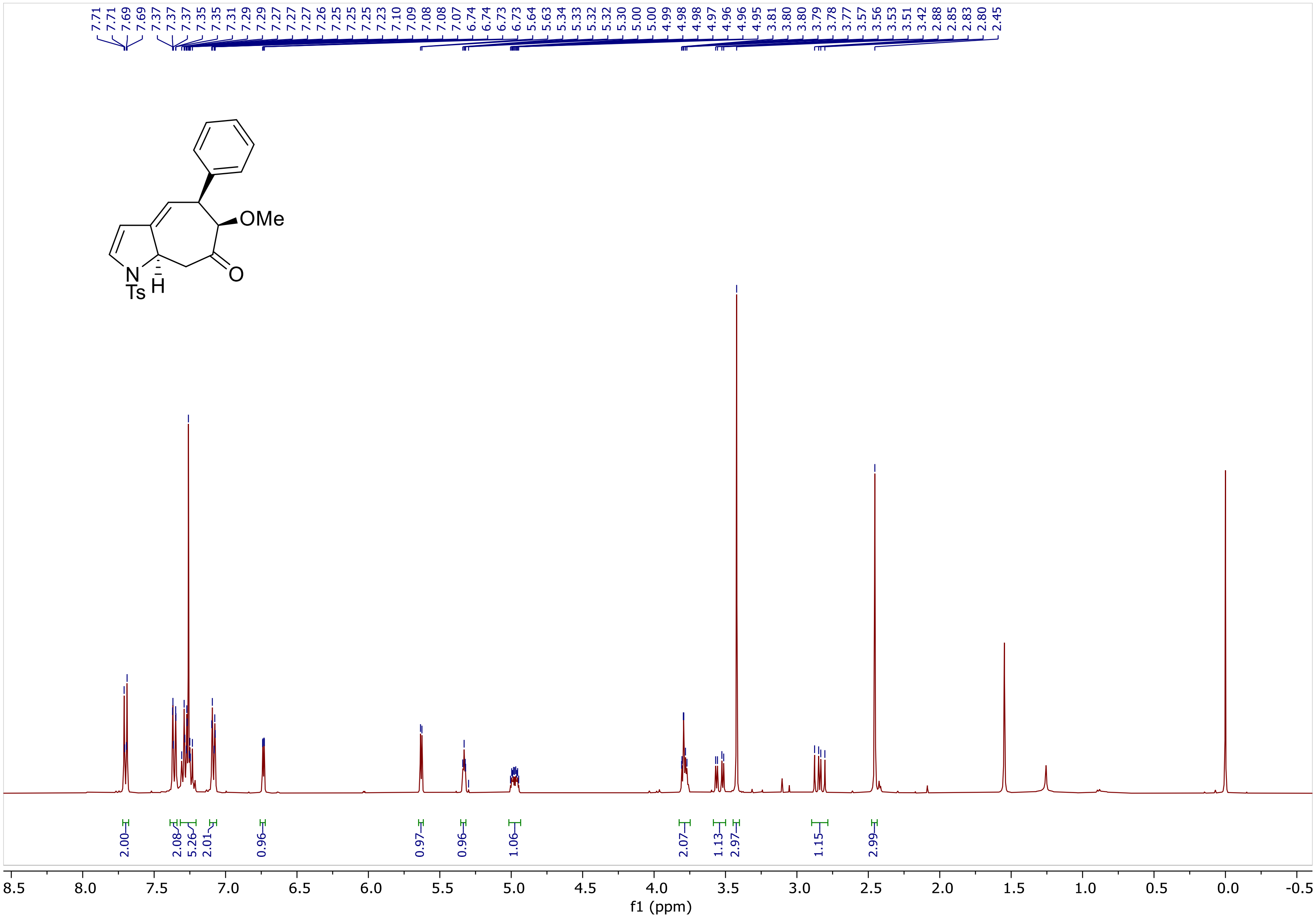

^{1}H NMR (400 MHz, $CDCl_3$) of compound **8b**

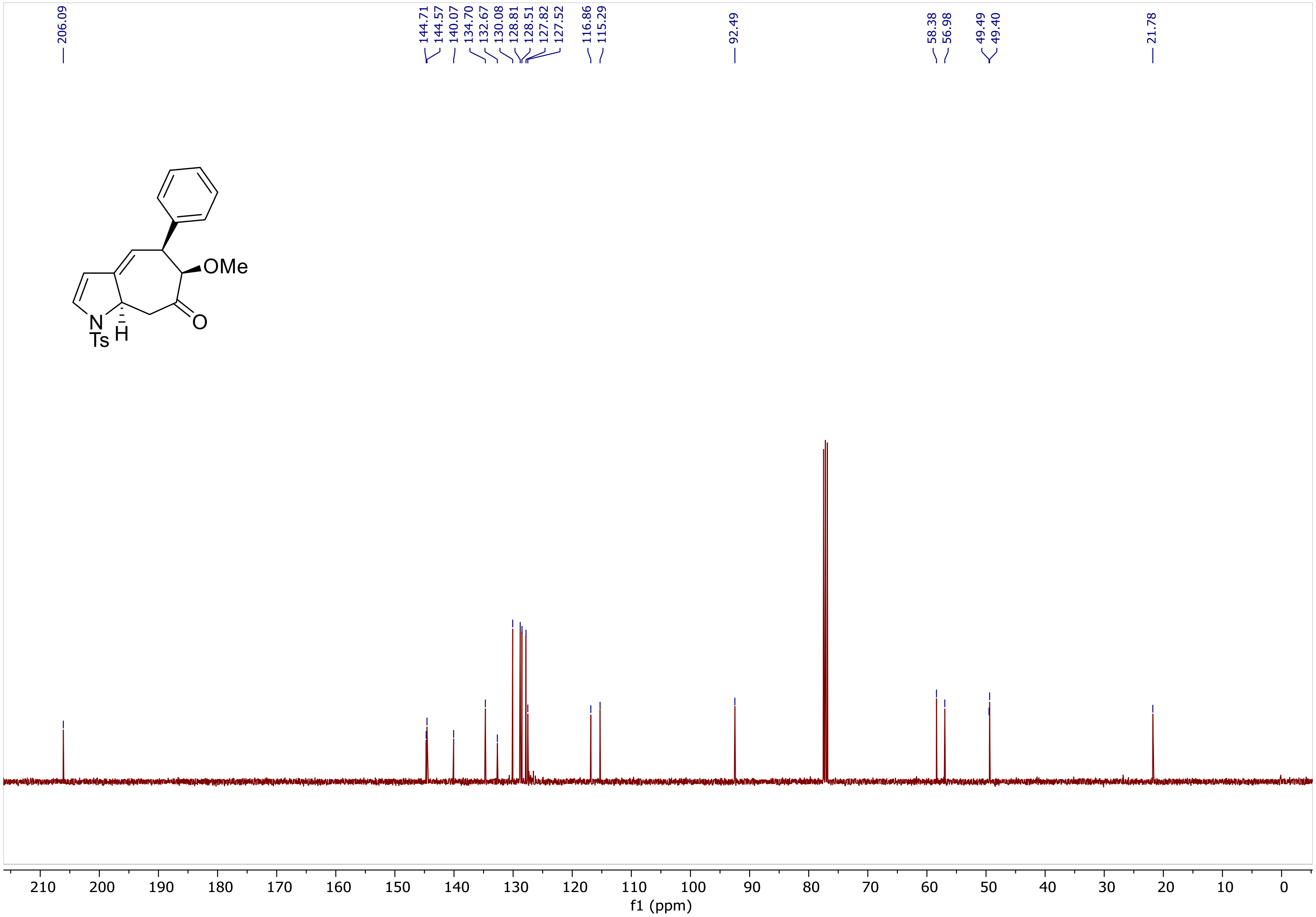

^{13}C NMR (101 MHz, $CDCl_3$) of compound **8b**

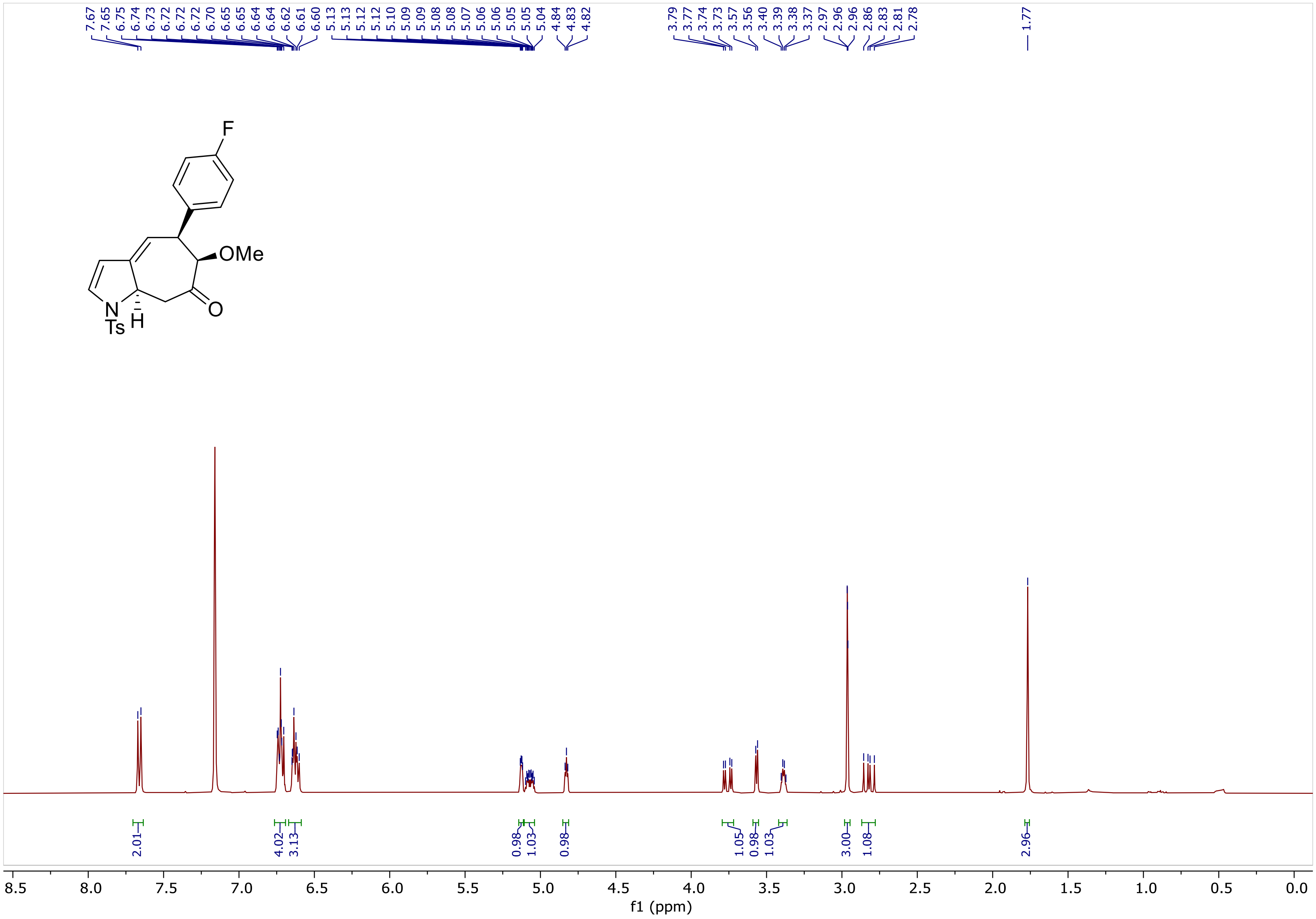

^{1}H NMR (400 MHz, $CDCl_3$) of compound **8c**

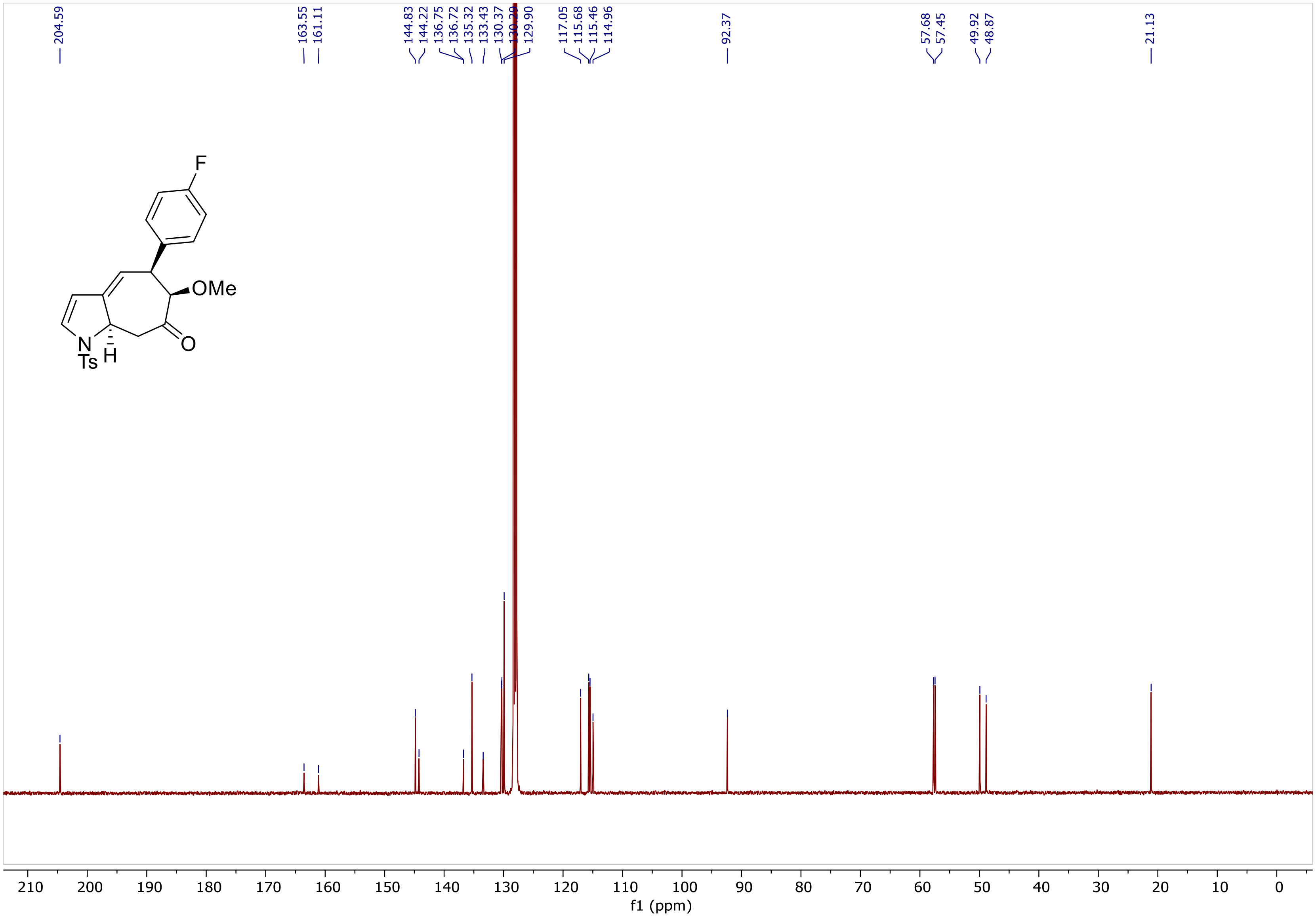

^{13}C NMR (101 MHz, $CDCl_3$) of compound **8c**

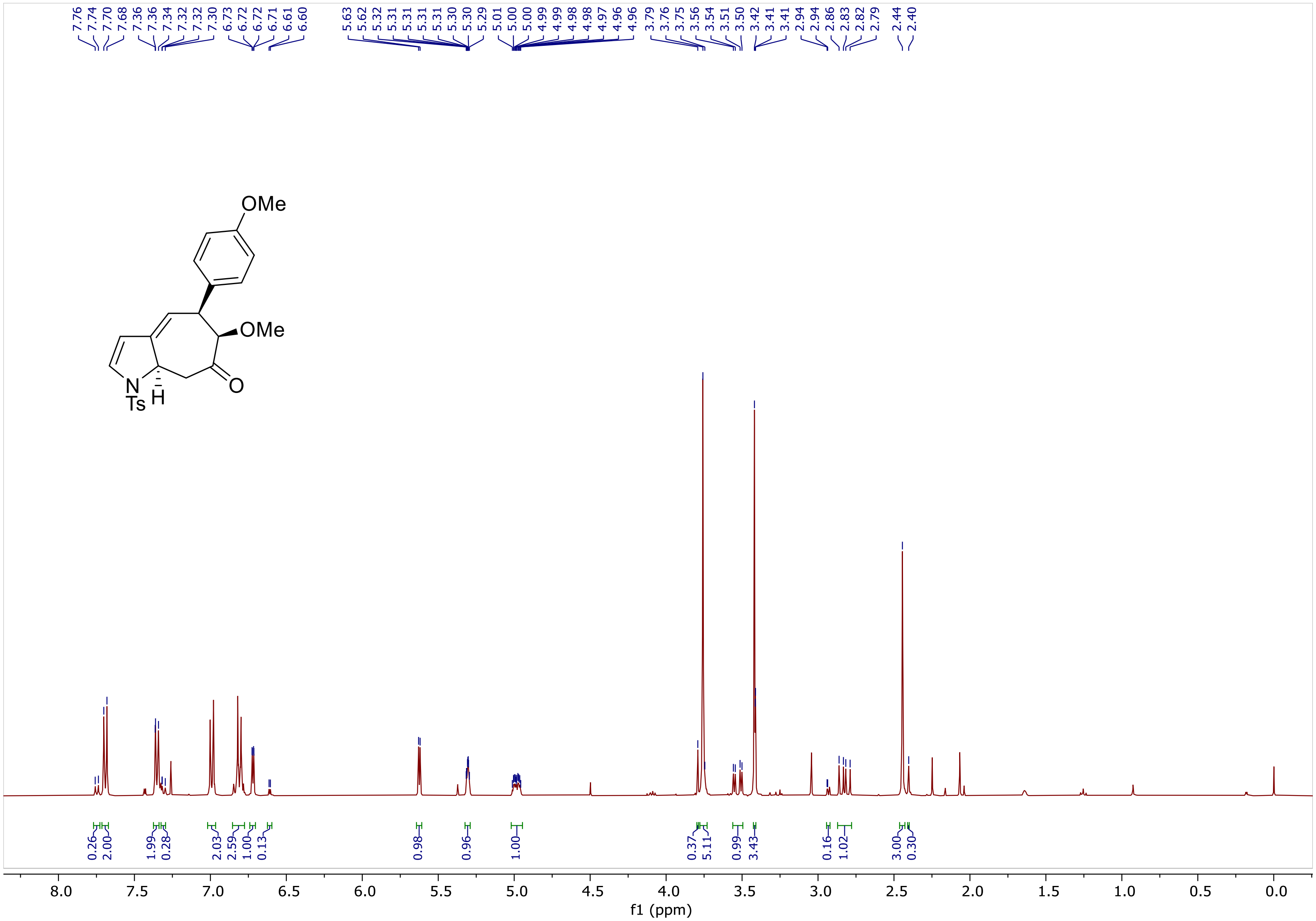

^{1}H NMR (400 MHz, $CDCl_3$) of compound **8d**

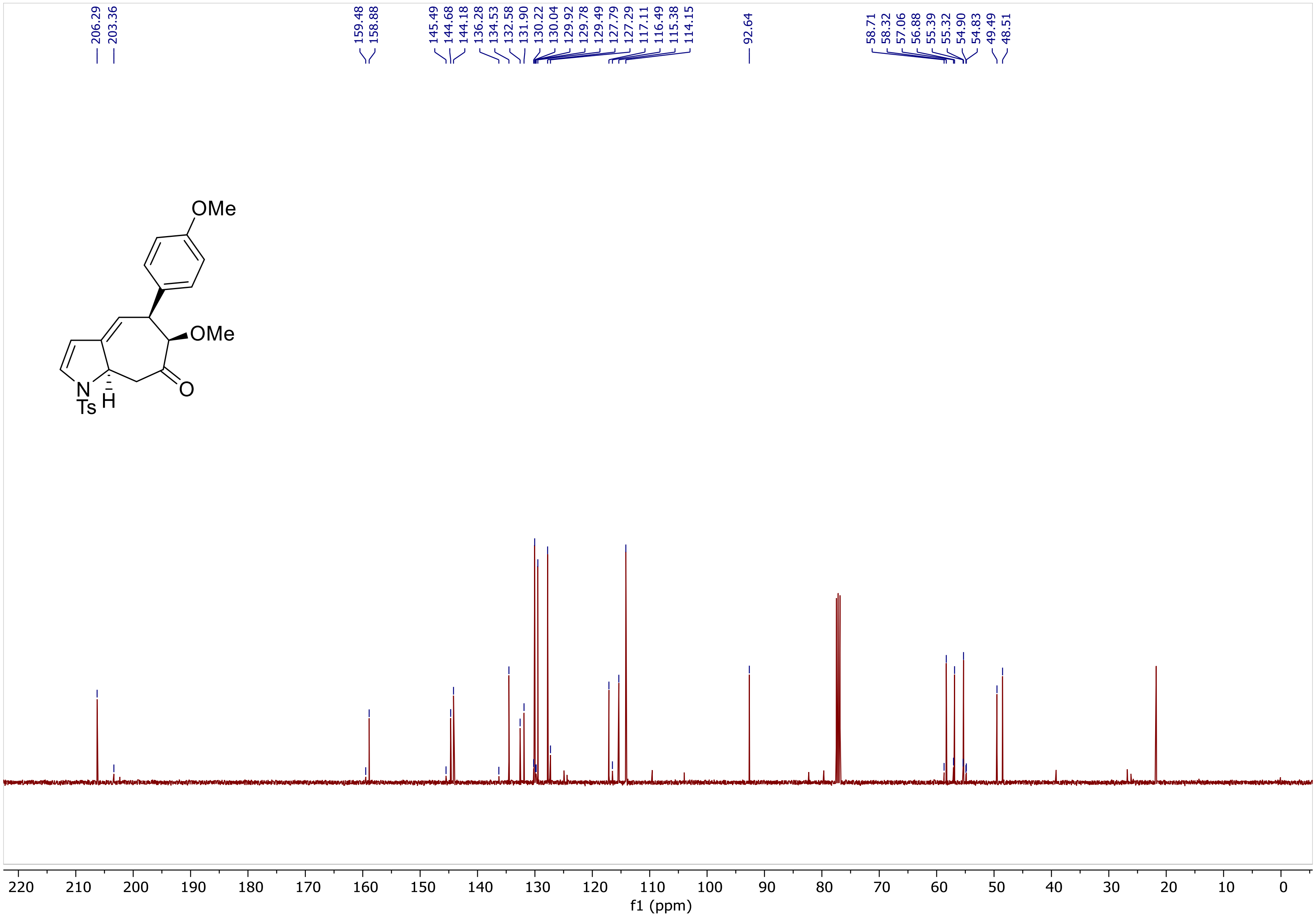

^{13}C NMR (101 MHz, $CDCl_3$) of compound **8d**

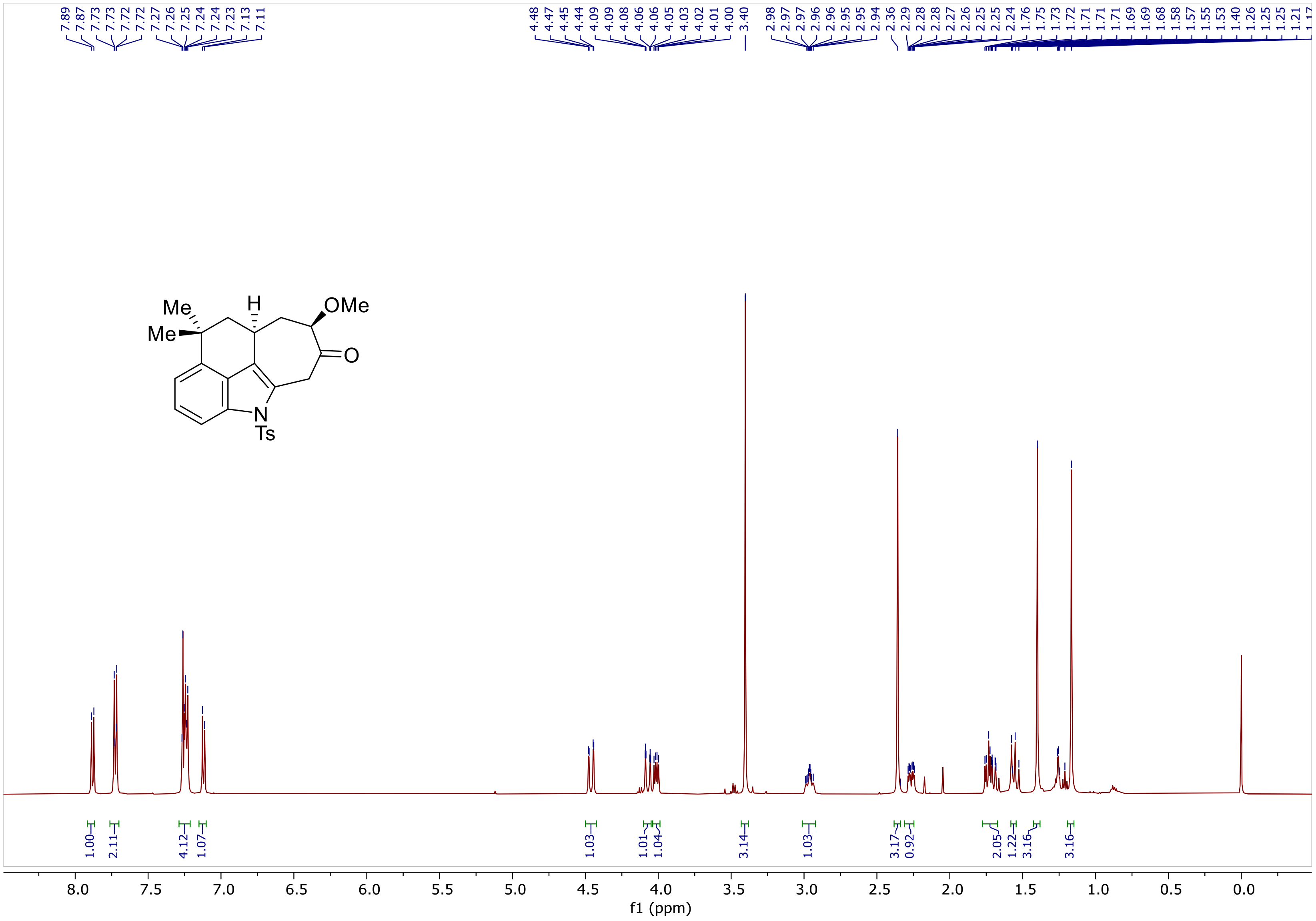

^{1}H NMR (400 MHz, $CDCl_3$) of compound **9**

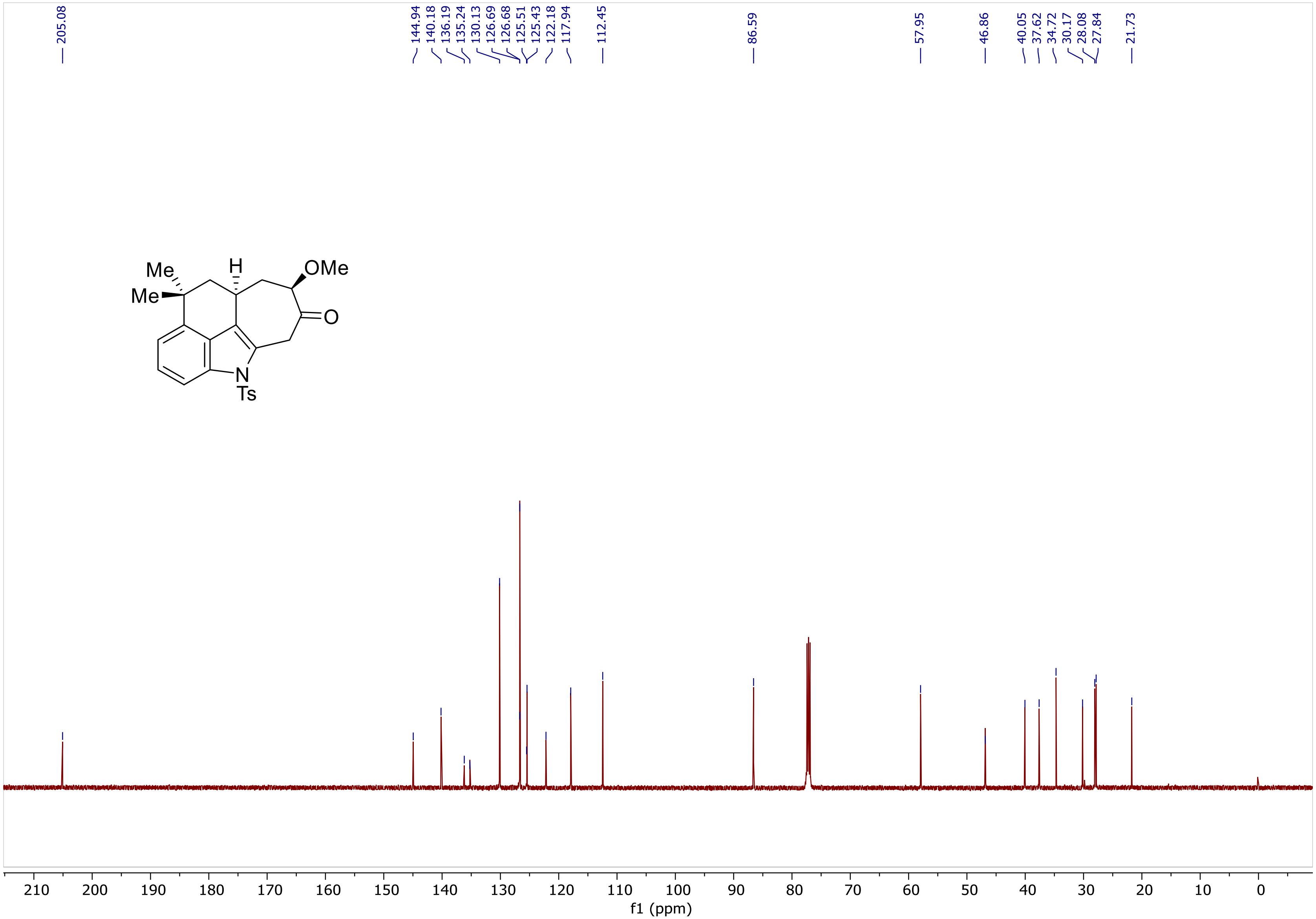

^{13}C NMR (101 MHz, $CDCl_3$) of compound **9**

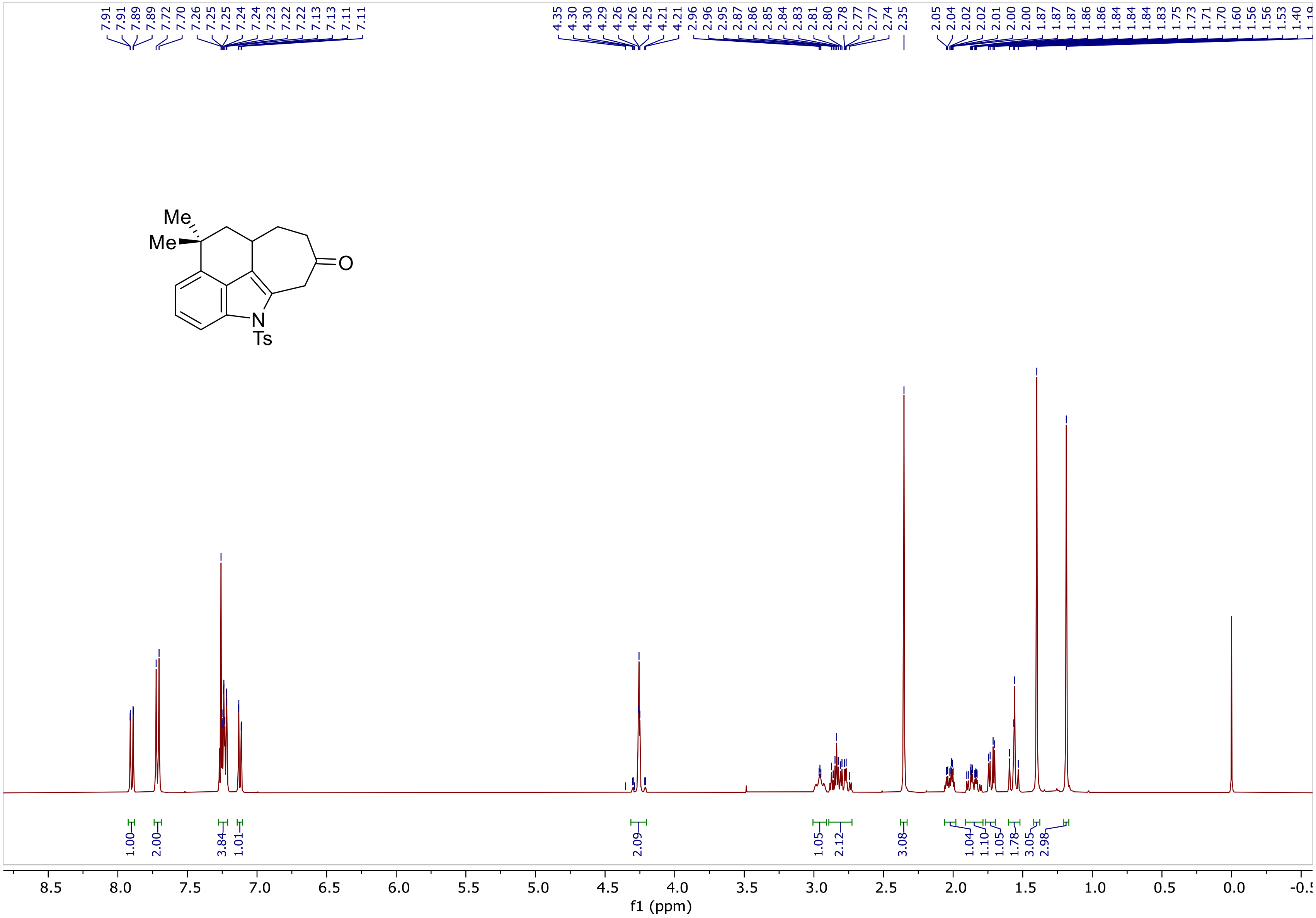

^{1}H NMR (400 MHz, $CDCl_3$) of compound **10**

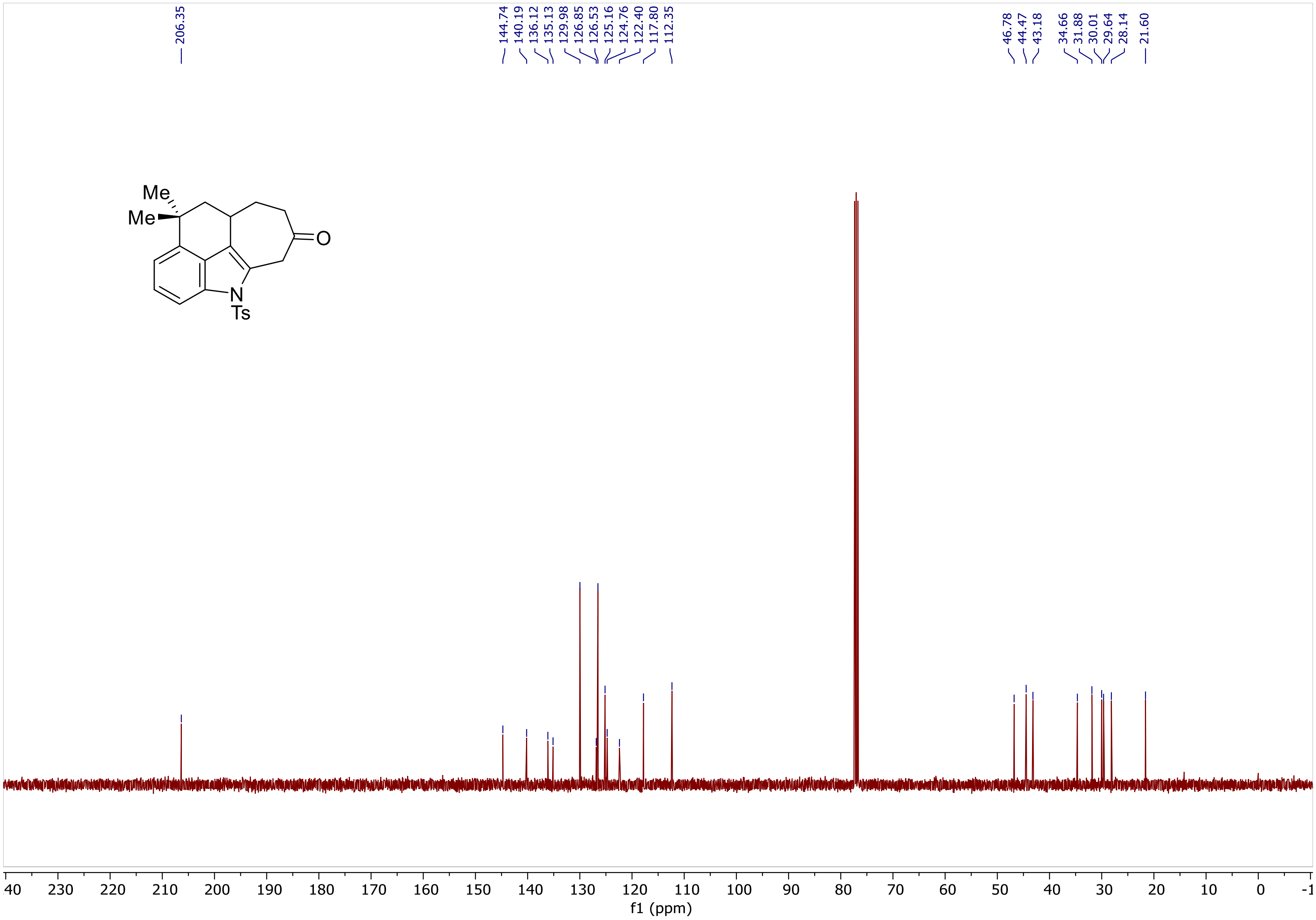

^{13}C NMR (101 MHz, $CDCl_3$) of compound **10**

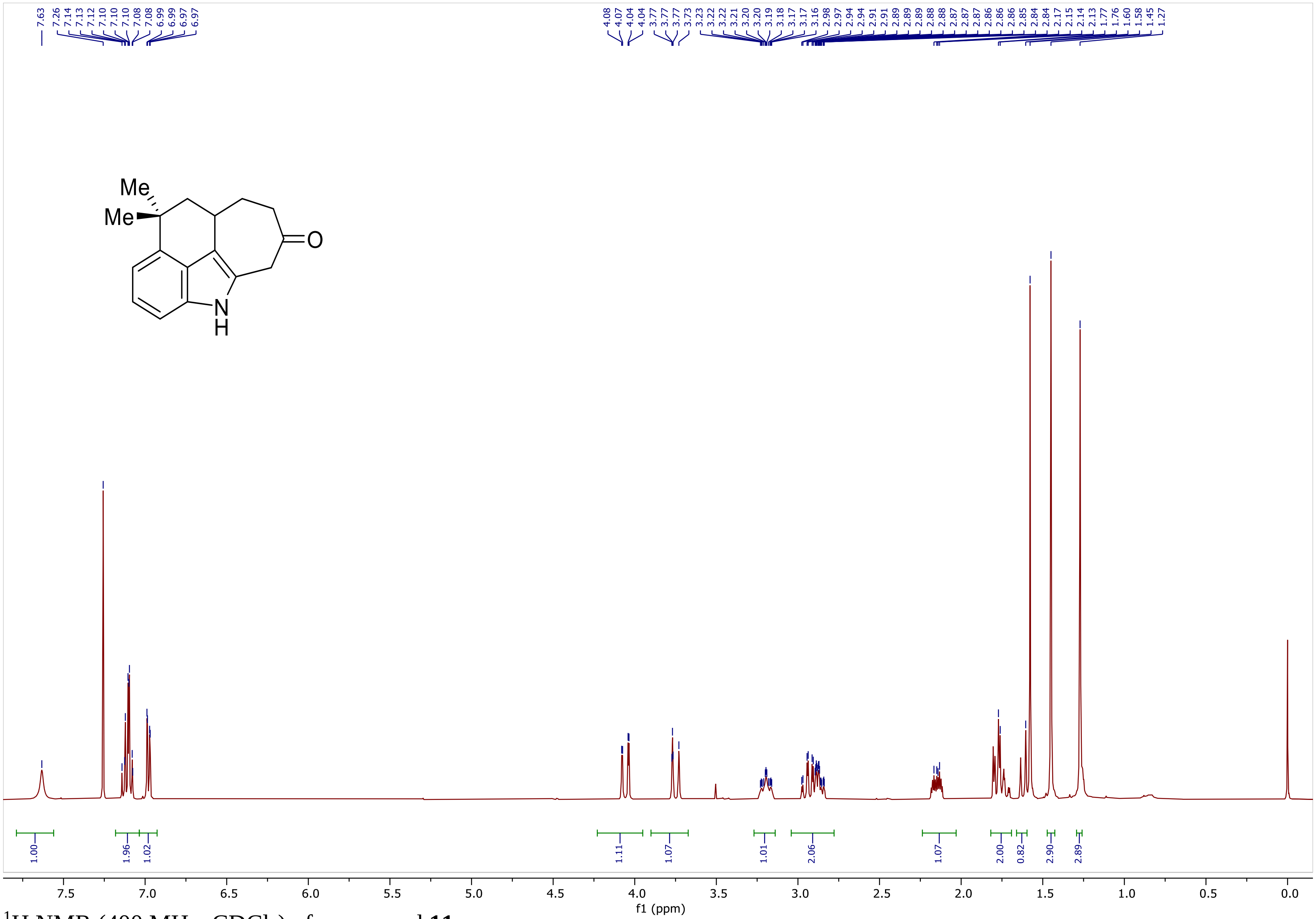

^{1}H NMR (400 MHz, $CDCl_3$) of compound **11**

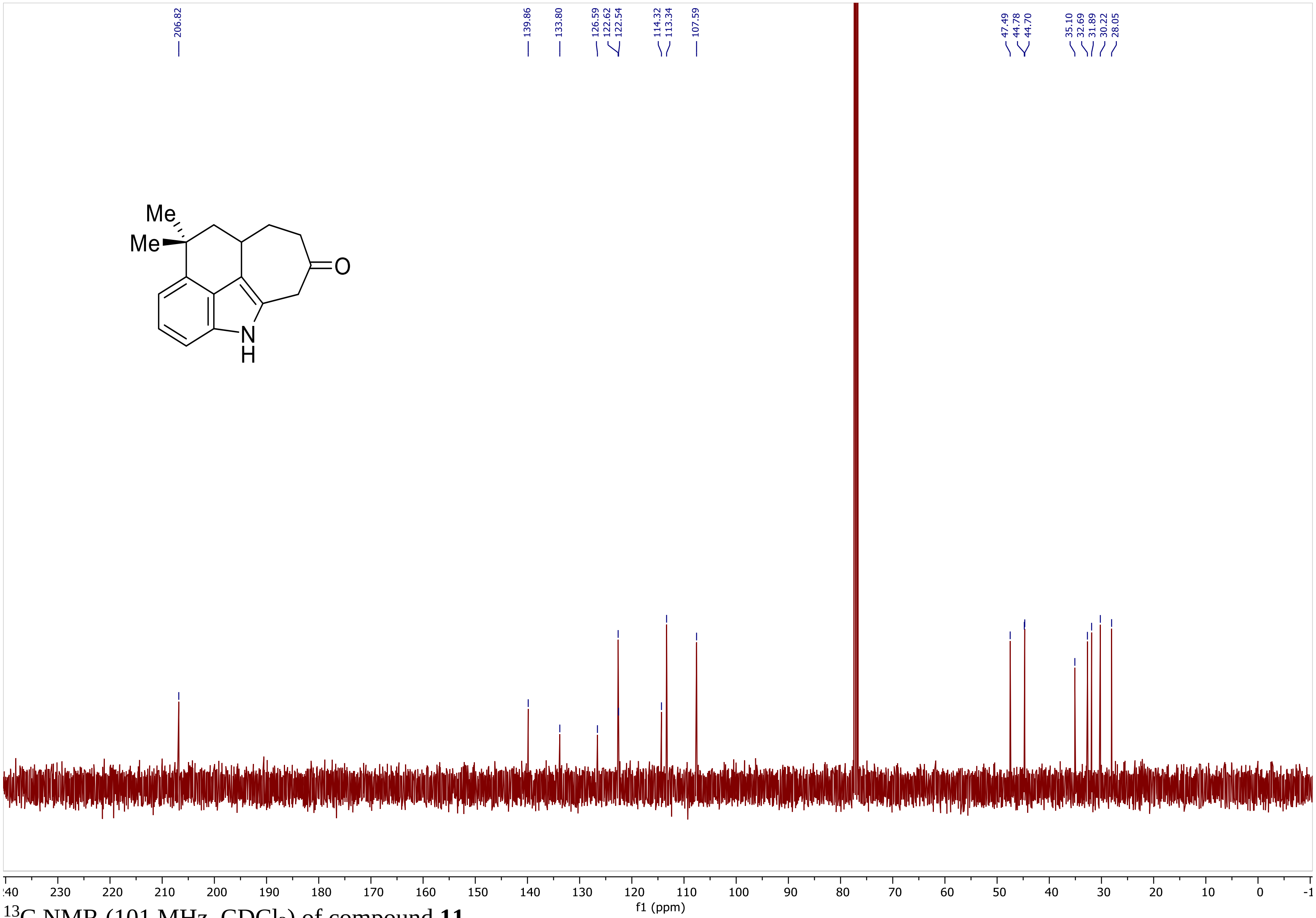

^{13}C NMR (101 MHz, $CDCl_3$) of compound **11**

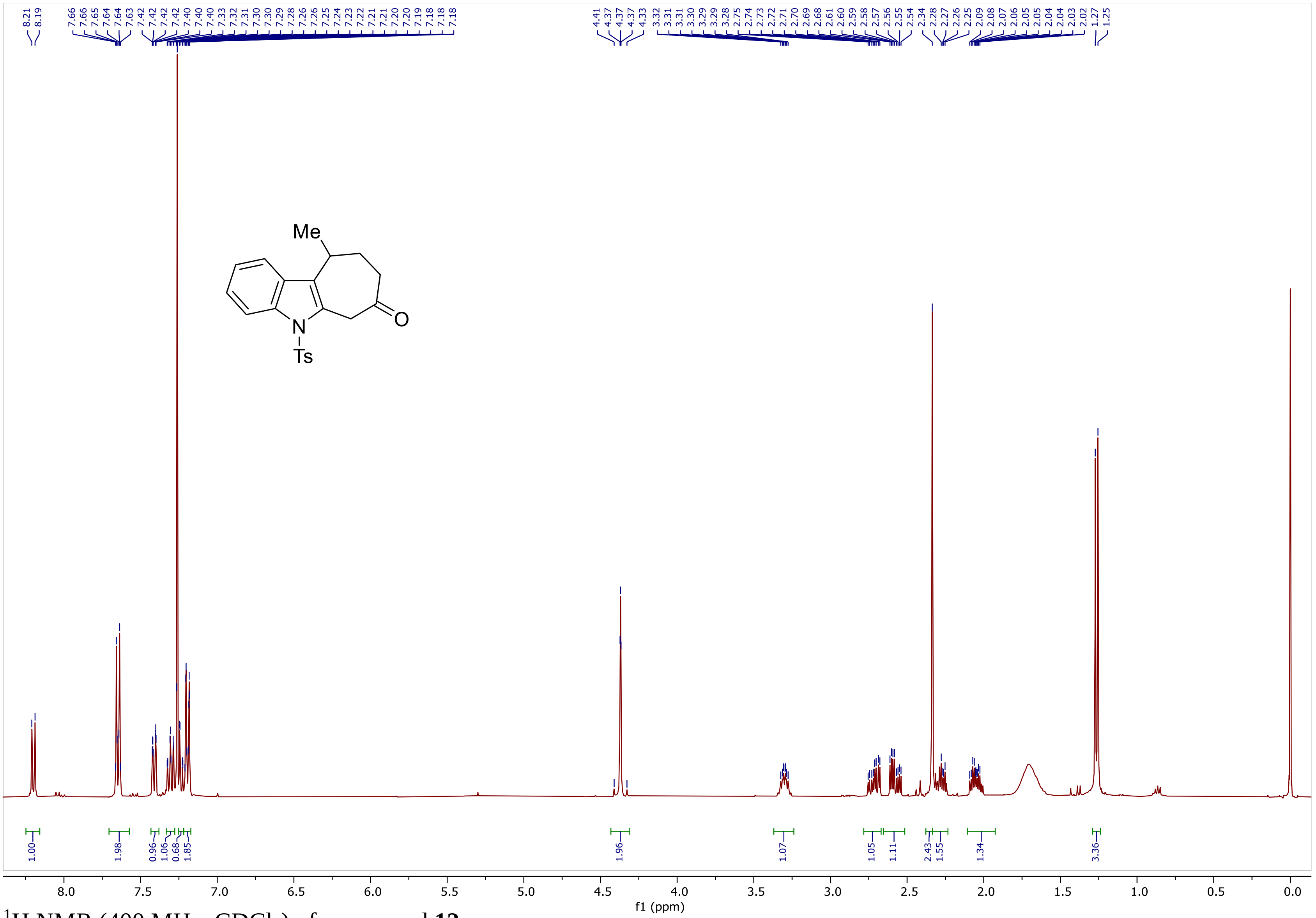

^{1}H NMR (400 MHz, $CDCl_3$) of compound **12**

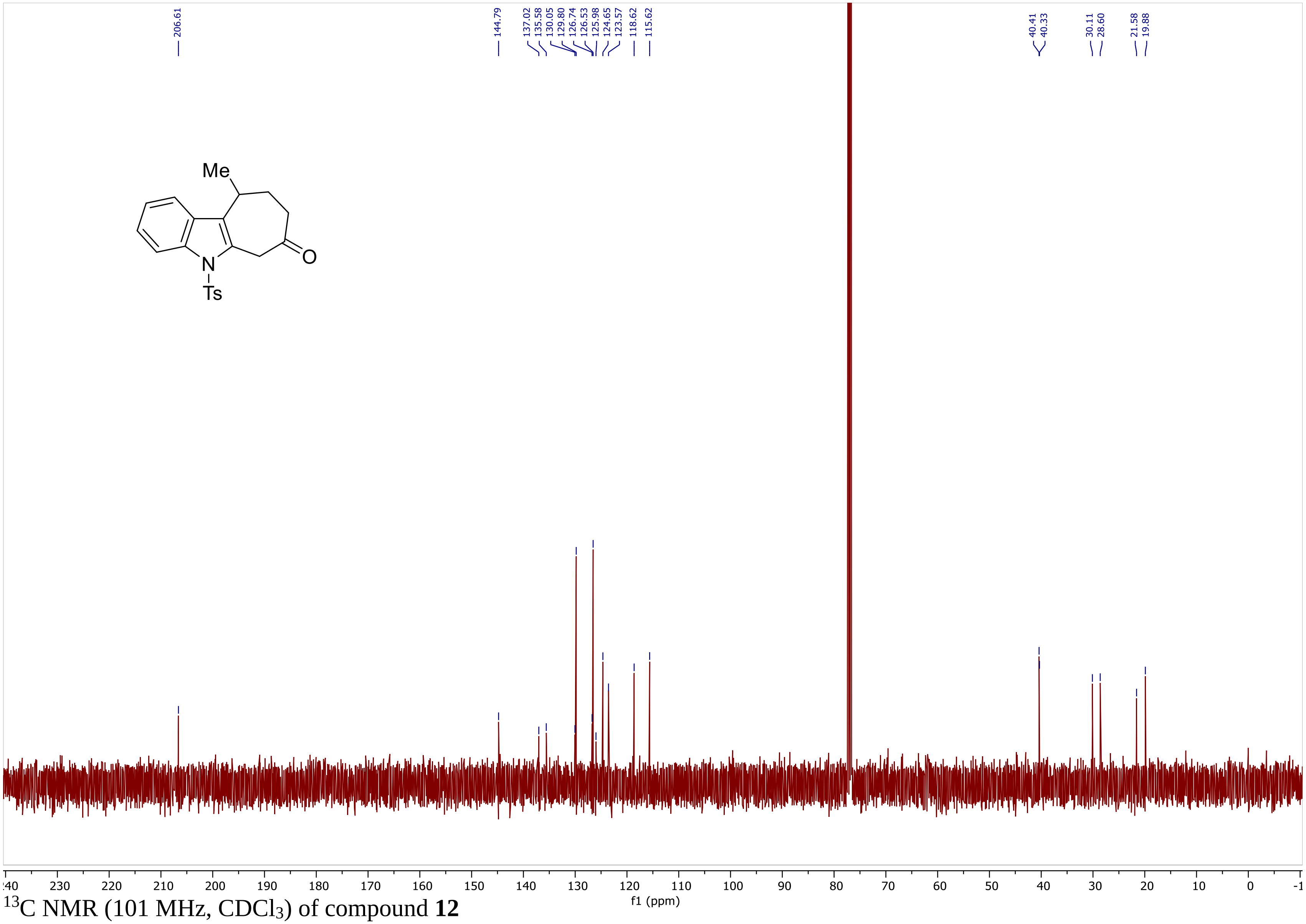

^{13}C NMR (101 MHz, CDCl$_3$) of compound **12**

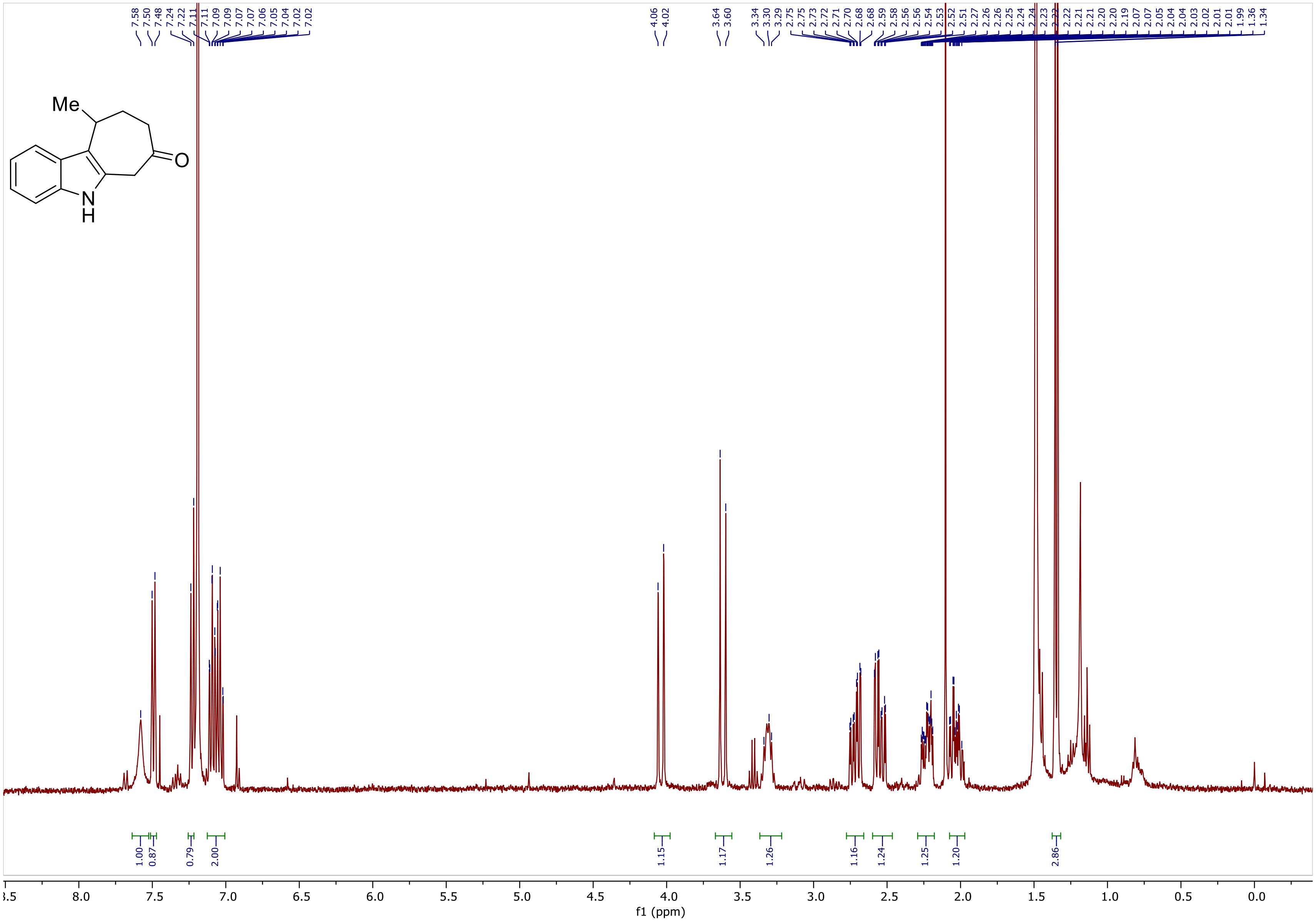

^1H NMR (400 MHz, $CDCl_3$) of compound **13**

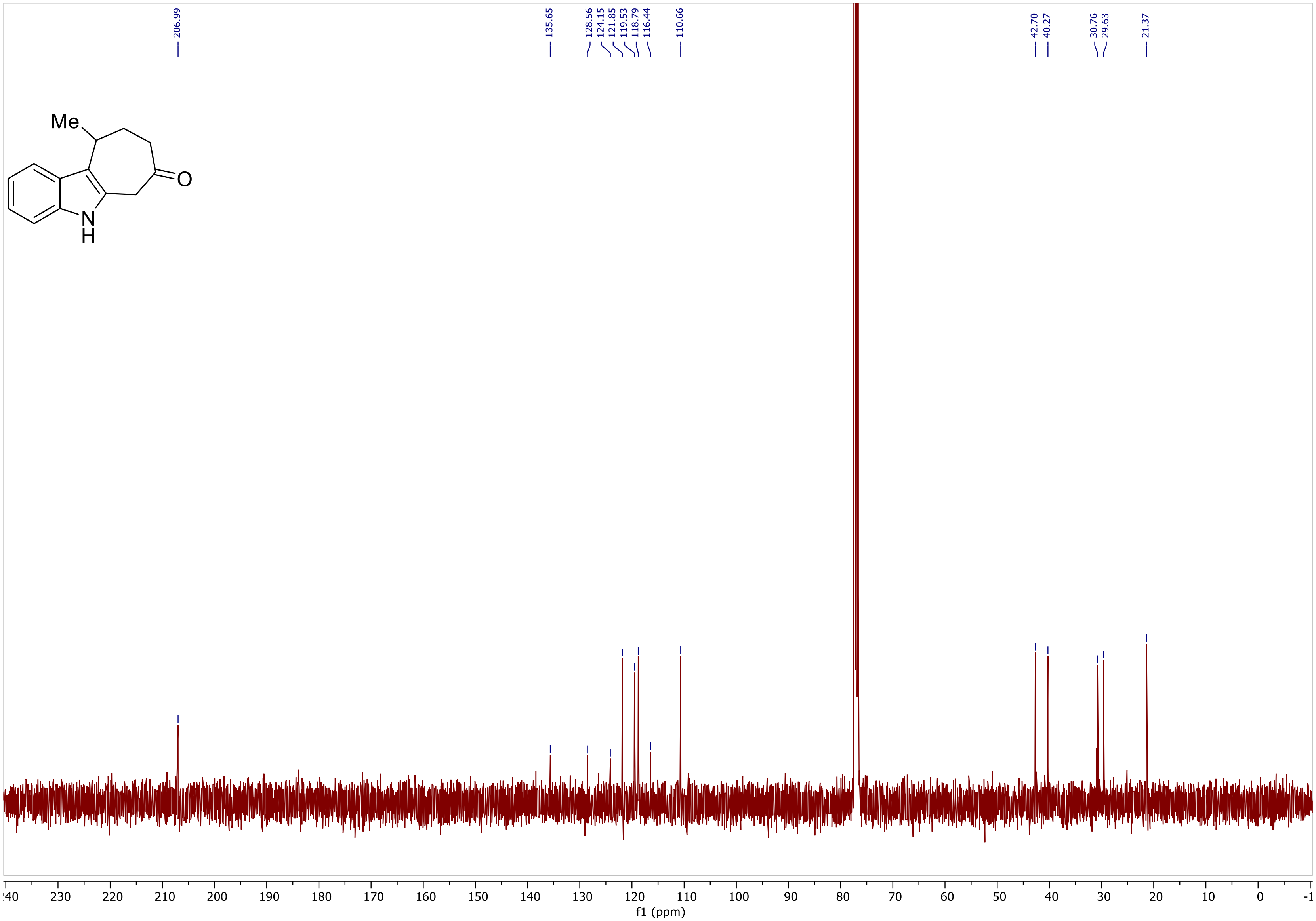

^{13}C NMR (101 MHz, $CDCl_3$) of compound **13**

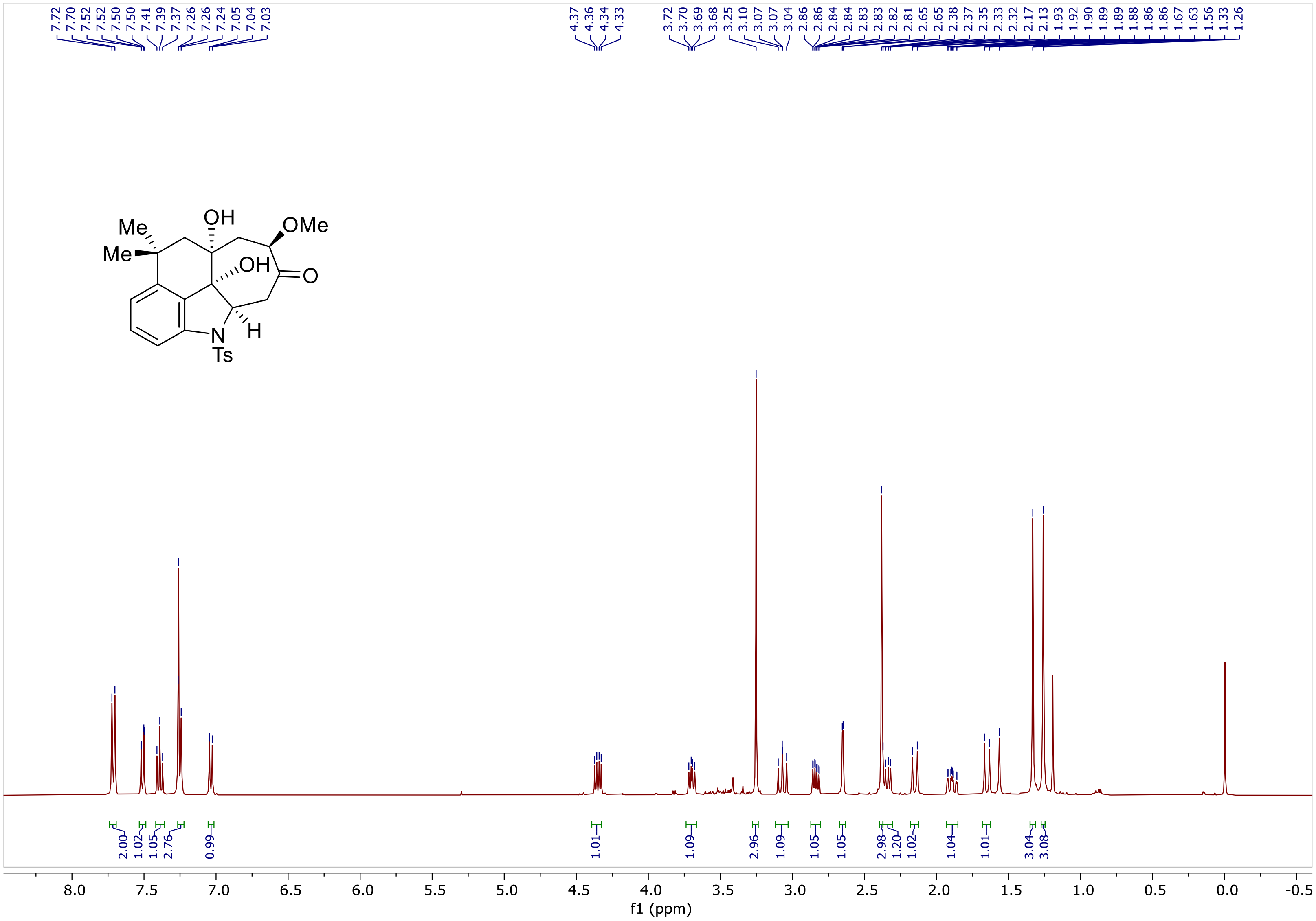

^{1}H NMR (400 MHz, $CDCl_3$) of compound **14**

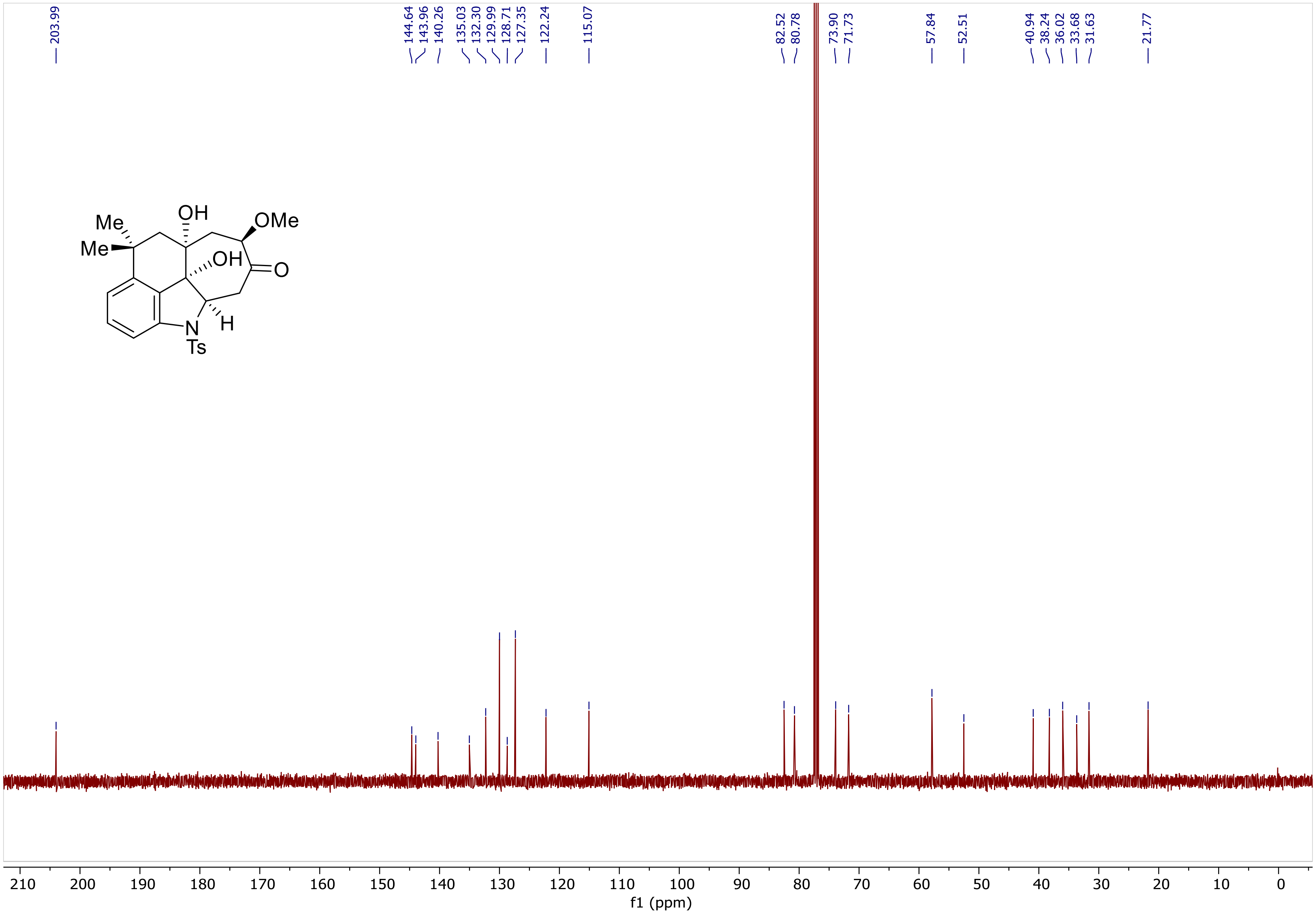

^{13}C NMR (101 MHz, $CDCl_3$) of compound **14**

X-ray Crystallographic Data for 4a

Data collected and structure solved/refined: Alexander S. Filatov, October/2017 (X-ray Laboratory, Searle B013, Department of Chemistry, the University of Chicago, Chicago, Il).

General information: a colorless plate (0.08 x 0.16 x 0.28 mm) was mounted on a Dual-Thickness MicroMount^{tm} (MiTeGen) with 30 μm sample aperture. The diffraction data were measured at 100 K on a Bruker D8 VENTURE diffractometer equipped with a microfocus Mo-target X-ray tube (λ = 0.71073 Å) and PHOTON 100 CMOS detector. Data were collected using ω scans to survey a hemisphere of reciprocal space. Data reduction and integration were performed with the Bruker APEX3 software package (Bruker AXS, version 2016.5-0, 2016). Data were scaled and corrected for absorption effects using the multi-scan procedure as implemented in SADABS (Bruker AXS, version 2014/5, Krause, Herbst-Irmer, Sheldrick & Stalke, *J. Appl. Cryst.* **2015**, *48*, 3-10). The structure was solved by SHELXT (Version 2014/5: Sheldrick, G. M. *Acta Crystallogr*. **2015**, *A71*, 3-8) and refined by a full-matrix least-squares procedure using OLEX2 (O. V. Dolomanov, L. J. Bourhis, R. J. Gildea, J. A. K. Howard and H. Puschmann. J. Appl. Crystallogr. **2009**, *42*, 339-341) (XL refinement program version 2016/6, *Sheldrick, G. M. Acta Crystallogr.* **2015**, *C71*, 3-8). Crystallographic data and details of the data collection and structure refinement are listed in Table 1.

Specific details for structure refinement: All atoms were refined with anisotropic thermal parameters. Hydrogen atoms were included in idealized positions for structure factor calculations. All structures are drawn with thermal ellipsoids at 40% probability.

Table 1 Crystal data and structure refinement for 0478.

Identification code	0478_jiasu
Empirical formula	$C_{25}H_{27}NO_4S$
Formula weight	437.53
Temperature/K	100(2)
Crystal system	monoclinic
Space group	$P2_1/c$
a/Å	15.1840(7)
b/Å	14.4401(7)
c/Å	20.0786(10)
α/°	90
β/°	94.491(2)
γ/°	90
Volume/Å^3	4388.9(4)
Z	8
ρ_{calc}g/cm^3	1.324
μ/mm^{-1}	0.180
F(000)	1856.0
Crystal size/mm^3	0.28 × 0.16 × 0.08
Radiation	MoKα (λ = 0.71073)
2Θ range for data collection/°	4.298 to 52.856
Index ranges	$-18 \le h \le 16$, $-15 \le k \le 18$, $-25 \le l \le 25$
Reflections collected	46181
Independent reflections	8999 [R_{int} = 0.0620, R_{sigma} = 0.0582]
Data/restraints/parameters	8999/9/567
Goodness-of-fit on F^2	1.015
Final R indexes [I>=2σ (I)]	R_1 = 0.0532, wR_2 = 0.1115
Final R indexes [all data]	R_1 = 0.1033, wR_2 = 0.1297
Largest diff. peak/hole / e Å^{-3}	0.55/-0.72

R_{int} = □□| F_o^2 - <F_o^2> | / □ | F_o^2|

R1 = $\Sigma || F_o| - | F_c|| / \Sigma| F_o|$

wR2 = $[\Sigma [w (F_o^2 - F_c^2)^2] / \Sigma [w (F_o^2)^2]]^{1/2}$

Goodness-of-fit = $[\Sigma [w (F_o^2 - F_c^2)^2] / (n-p)^{1/2}$

n: number of independent reflections; p: number of refined parameters

There are 2 co-crystallized symmetrically independent molecules:

“1”

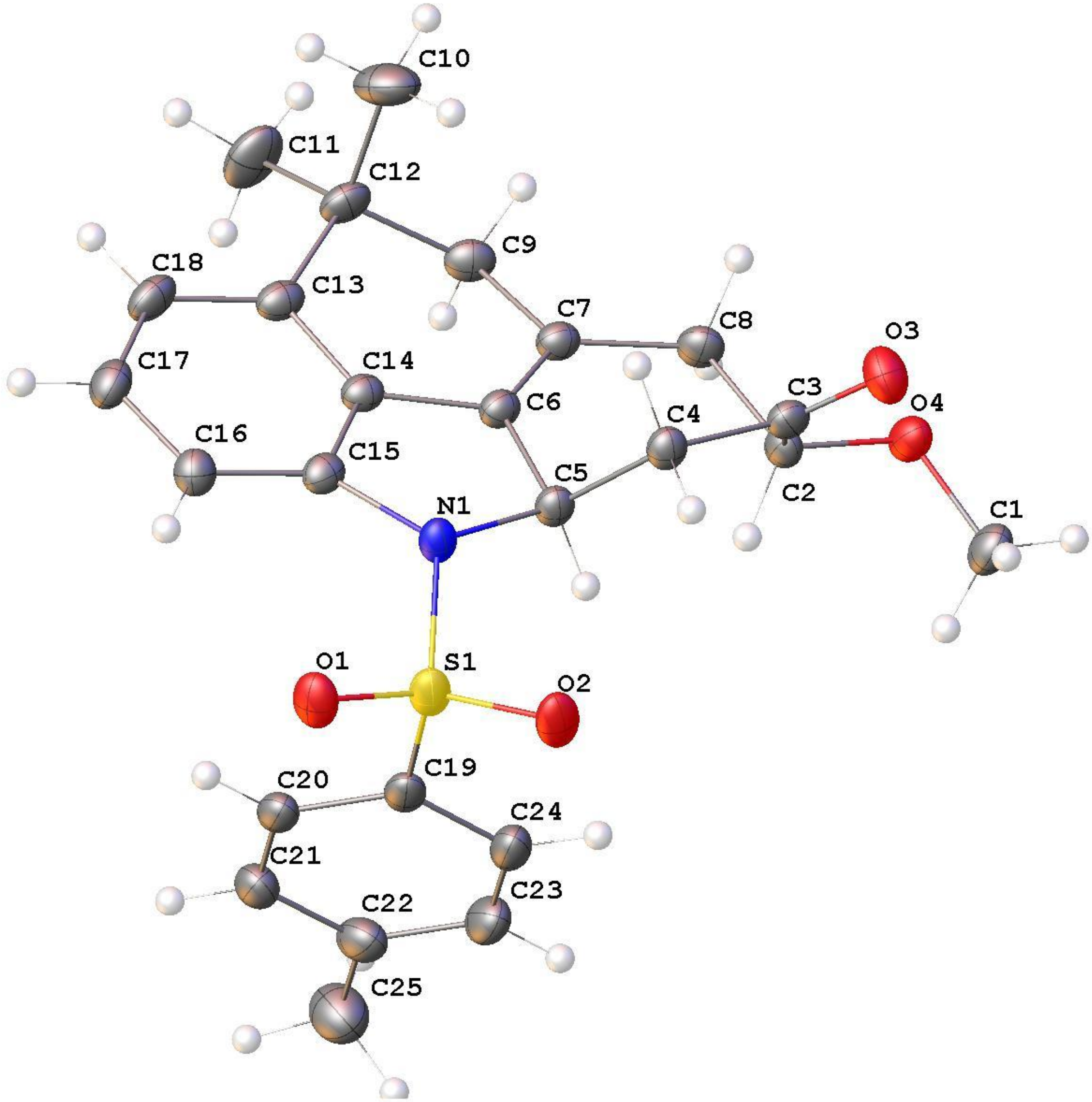

C10
C11
C12
C9
C18
C13
C7
C8
O3
C17
C14
C6
C3
O4
C4
C16
C15
C5
C2
N1
C1
O1
S1
O2
C19
C20
C24
C21
C23
C22
C25

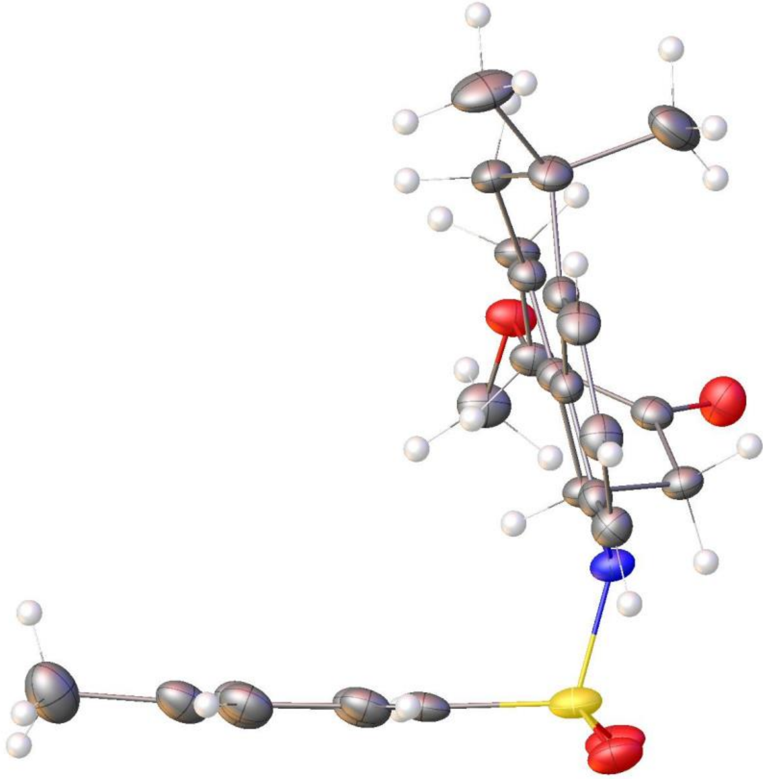

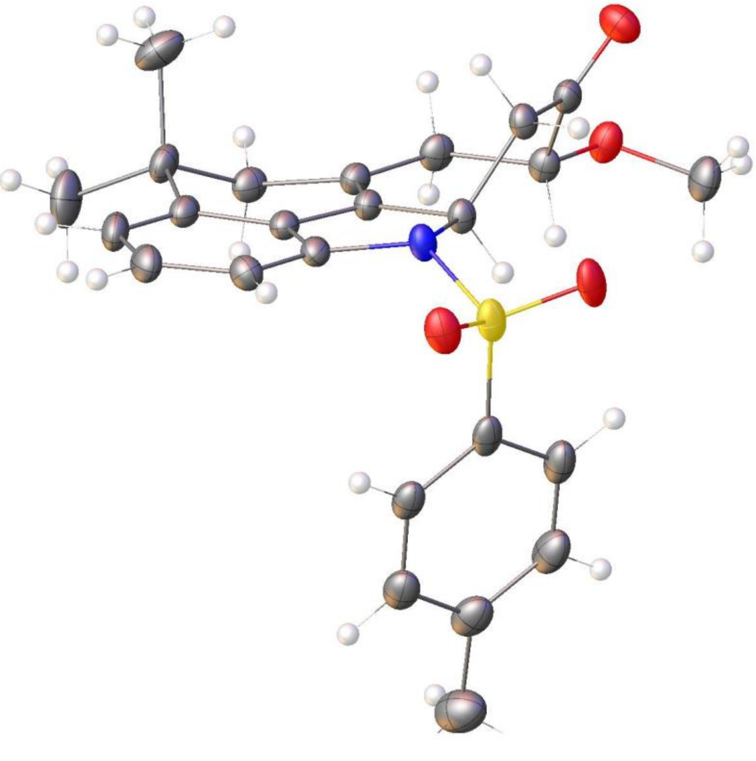

“2”

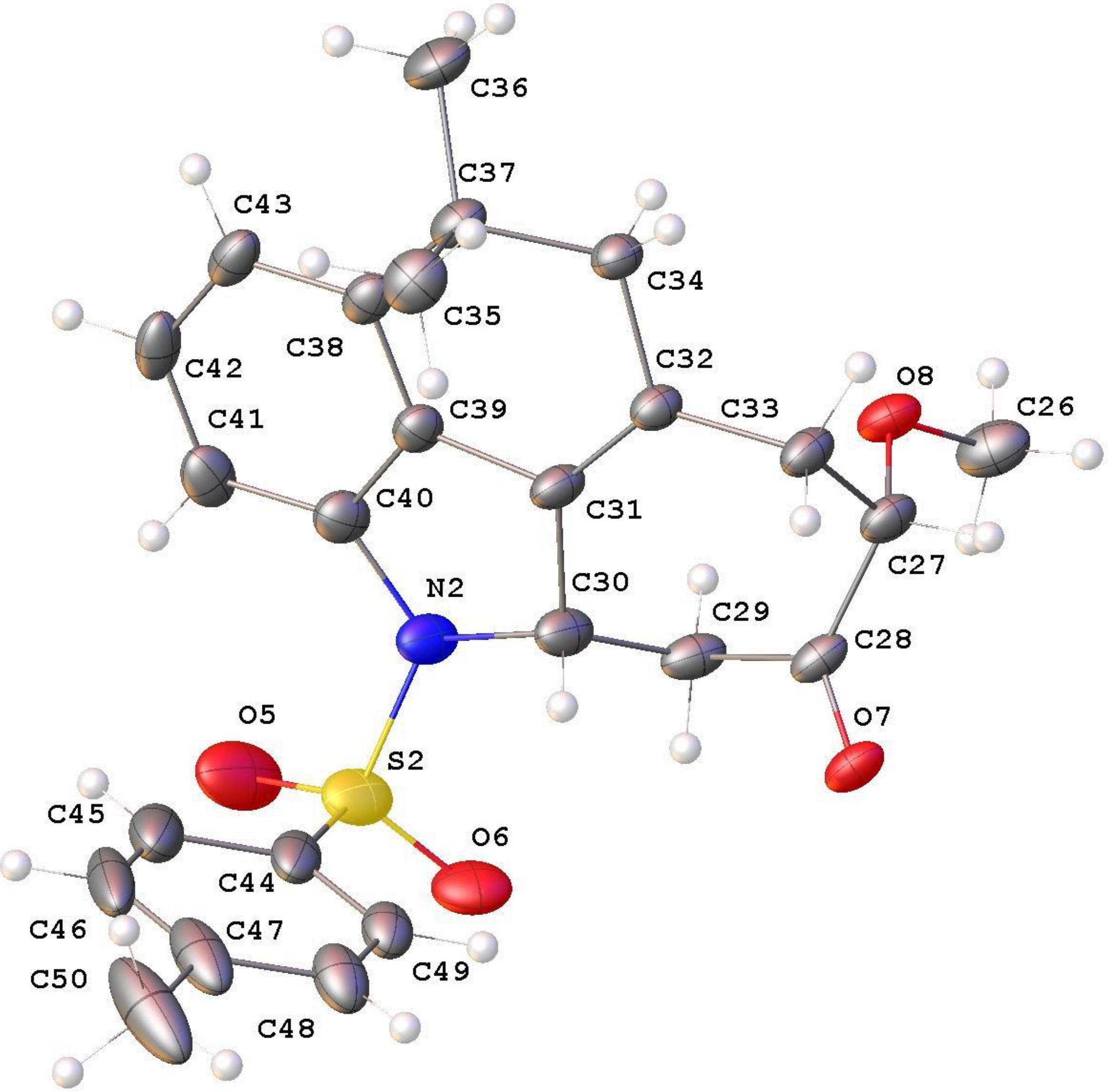
C36
C37
C43
C34
C35
C38
C42
C32
C41
C39
C33
O8
C26
C40
C31
C27
N2
C30
C29
C28
O5
O7
S2
C45
O6
C44
C46
C47
C49
C50
C48

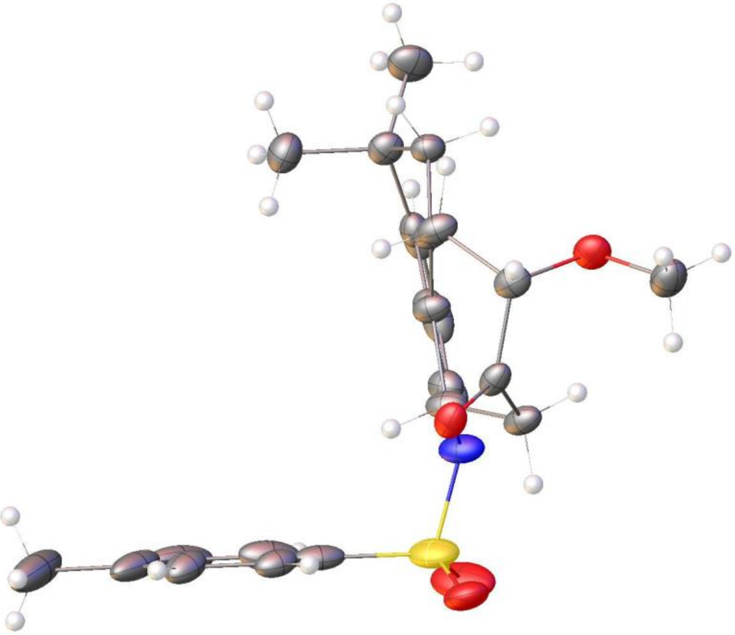

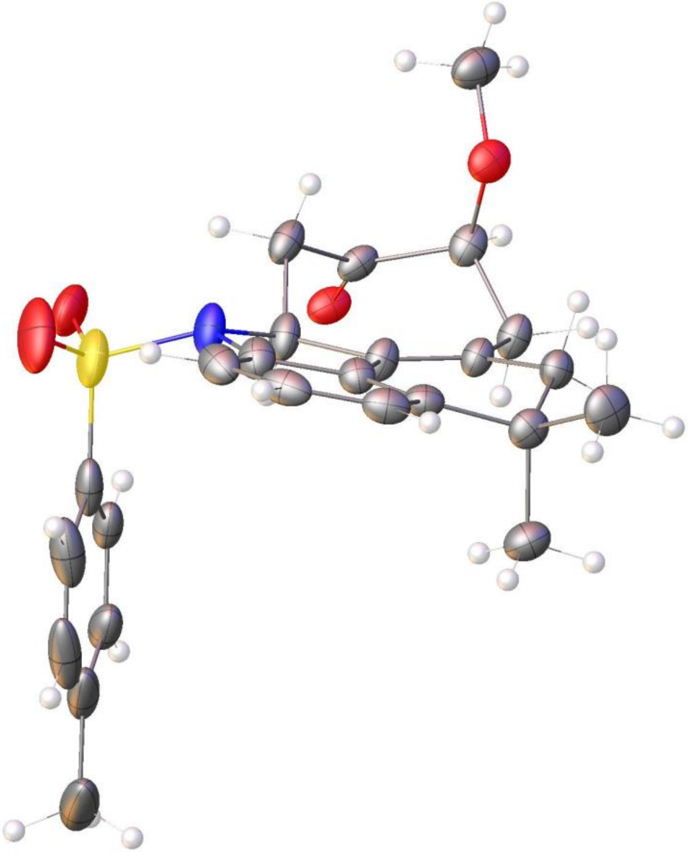

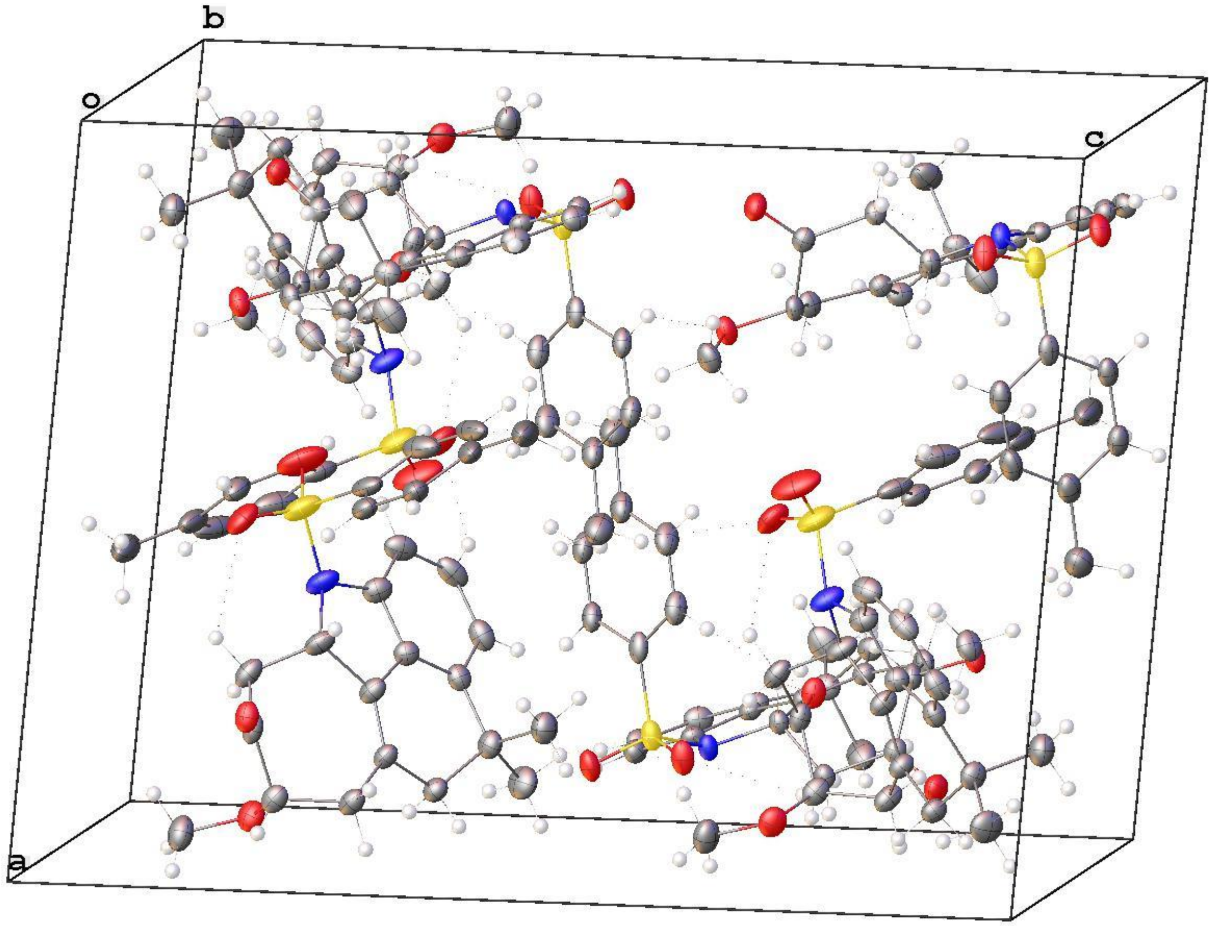
b
o
c
a

Table 2 Fractional Atomic Coordinates (×10^4) and Equivalent Isotropic Displacement Parameters (Å^2×10^3) for 0478_jiasu. U_{eq} is defined as 1/3 of of the trace of the orthogonalised U_{IJ} tensor.

Atom	*X*	*y*	*Z*	U(eq)
S1	1530.4(4)	3679.9(4)	4465.3(3)	31.48(17)
O1	1089.0(12)	3551.0(11)	5061.3(8)	36.6(4)
O2	1341.3(12)	4464.0(11)	4041.9(8)	38.1(4)
O3	960.5(12)	3485.0(12)	1609.2(8)	38.9(4)
O4	2640.0(11)	3087.6(11)	1396.2(8)	33.1(4)
N1	1298.0(13)	2767.3(12)	3991.5(9)	25.7(4)
C1	2902.1(18)	4022.8(17)	1355.9(13)	38.6(6)
C2	2338.2(16)	2835.0(15)	2021.0(11)	26.1(5)
C3	1399.9(16)	3182.5(15)	2086.0(12)	27.0(5)
C4	1020.6(15)	3140.1(15)	2767.5(11)	25.5(5)
C5	1664.4(15)	2763.2(15)	3314.3(11)	24.2(5)
C6	1867.0(15)	1753.4(15)	3202.5(11)	23.9(5)
C7	2175.1(15)	1323.3(15)	2680.7(11)	25.6(5)
C8	2402.0(17)	1774.8(15)	2045.4(12)	29.9(6)
C9	2389.0(16)	299.4(15)	2762.8(12)	29.7(6)
C10	918.2(19)	-430(2)	2829.7(15)	49.4(8)
C11	2259(2)	-1179.4(17)	3386.8(16)	51.7(8)
C12	1810.3(16)	-256.1(16)	3218.7(13)	32.0(6)
C13	1624.6(15)	293.4(15)	3842.8(12)	27.8(6)
C14	1633.9(14)	1253.0(15)	3783.6(11)	23.7(5)
C15	1336.2(14)	1841.9(15)	4261.4(12)	24.8(5)
C16	1068.0(16)	1496.8(16)	4858.9(12)	29.4(6)
C17	1093.0(16)	541.0(17)	4934.5(13)	33.3(6)
C18	1347.7(16)	-53.8(17)	4436.9(13)	33.9(6)
C19	2672.2(17)	3652.2(16)	4679.1(12)	31.4(6)
C20	2988.4(17)	3135.9(16)	5229.9(12)	31.8(6)
C21	3885.7(18)	3073.9(18)	5389.8(13)	37.4(6)
C22	4482.3(19)	3515.4(19)	5000.2(13)	40.9(7)
C23	4151(2)	4041.5(19)	4459.5(13)	43.0(7)
C24	3256.7(19)	4115.8(17)	4294.2(13)	38.1(7)
C25	5465(2)	3396(2)	5151.9(16)	57.7(9)
S2	4817.2(5)	7005.0(5)	2655.9(4)	52.9(2)

O5	5342.8(14)	7807.8(15)	2796.1(14)	80.5(8)
O6	4721.4(13)	6324.6(14)	3164.2(10)	54.2(5)
O7	2298.1(11)	4833.1(11)	2761.2(8)	34.8(4)
O8	794.9(12)	6689.0(11)	2834.7(9)	40.0(5)
N2	3815.5(14)	7378.5(14)	2428.0(12)	42.0(6)
C26	553(2)	6518(2)	3498.3(14)	48.9(8)
C27	1121.6(17)	5890.6(16)	2527.5(13)	34.4(6)
C28	2053.5(17)	5633.5(16)	2781.0(12)	31.2(6)
C29	2700.5(17)	6394.3(17)	2962.9(13)	36.6(6)
C30	3095.7(16)	6664.7(17)	2315.0(13)	37.0(6)
C31	2450.5(17)	7120.0(16)	1798.2(13)	32.4(6)
C32	1616.4(16)	6919.3(16)	1581.8(12)	29.5(6)
C33	1094.2(17)	6085.7(17)	1778.3(12)	34.5(6)
C34	1125.8(16)	7620.8(16)	1135.2(12)	31.2(6)
C35	1981(2)	7685(2)	118.9(14)	45.9(7)
C36	1102(2)	9052(2)	438.0(15)	50.7(8)
C37	1675.5(17)	8252.4(17)	709.0(13)	35.3(6)
C38	2500.0(16)	8581.1(16)	1116.9(12)	30.8(6)
C39	2860.9(16)	7970.2(16)	1592.5(12)	29.9(6)
C40	3655.6(17)	8124.3(17)	1959.2(14)	36.6(6)
C41	4128.3(18)	8929.7(17)	1862.0(14)	40.4(7)
C42	3765.4(19)	9553.3(18)	1396.8(14)	41.3(7)
C43	2973.0(18)	9393.2(17)	1025.4(14)	37.4(6)
C44	5195.1(17)	6435(2)	1967.7(15)	46.7(8)
C45	5685(2)	6905(3)	1520(2)	66.7(11)
C46	5977(2)	6437(3)	984(2)	76.0(13)
C47	5798.9(19)	5494(3)	873.2(15)	65.8(11)
C48	5304.6(17)	5038(2)	1326.4(14)	49.3(8)
C49	5010.7(17)	5499(2)	1865.5(14)	40.6(7)
C50	6129(2)	4984(4)	286.4(16)	103.4(18)

Table 3 Anisotropic Displacement Parameters (Å^2×10^3) for 0478_jiasu. The Anisotropic displacement factor exponent takes the form: $-2\pi^2[h^2a^{*2}U_{11}+2hka^*b^*U_{12}+\ldots]$.

Atom	U_{11}	U_{22}	U_{33}	U_{23}	U_{13}	U_{12}
S1	51.6(4)	19.0(3)	24.4(3)	-1.7(3)	6.9(3)	4.9(3)
O1	54.4(11)	31(1)	25.9(9)	-2.3(8)	12.0(8)	8.7(8)

O2	66.2(13)	19.0(8)	29.8(10)	0.5(7)	6.8(9)	10.8(8)
O3	41.8(11)	46.4(11)	28(1)	5.6(8)	0.5(8)	4.8(9)
O4	45.8(11)	28.2(9)	26.4(9)	-2.2(7)	10.1(8)	-6.2(8)
N1	36.8(12)	18.9(10)	21.8(11)	0.6(8)	5.1(9)	4.4(8)
C1	50.5(17)	30.9(14)	35.8(15)	4.1(12)	12.3(13)	-7.6(13)
C2	35.3(14)	24.8(12)	18.7(12)	-1.6(10)	5.4(10)	-2.3(10)
C3	34.2(14)	18.2(12)	28.2(14)	-0.6(10)	-0.1(11)	-1.4(10)
C4	32.2(13)	19.3(12)	25.3(13)	-0.1(10)	4.3(10)	3.2(10)
C5	31.8(13)	18.1(11)	23.1(13)	-1.1(9)	4.2(10)	1.6(10)
C6	25.6(12)	18.9(11)	26.8(13)	0.5(10)	0(1)	-0.2(10)
C7	27.4(13)	20.7(12)	28.3(13)	-1.7(10)	0.4(10)	-0.4(10)
C8	37.9(15)	24.6(13)	27.6(14)	-6.9(11)	4.9(11)	-0.2(11)
C9	30.1(13)	23.6(12)	35.3(14)	-7.0(11)	2.5(11)	3.5(11)
C10	46.6(17)	50.6(18)	51.0(19)	-16.5(15)	3.8(14)	-18.5(15)
C11	73(2)	20.5(14)	64(2)	0.9(13)	22.0(17)	5.9(14)
C12	36.8(14)	17.4(12)	41.9(16)	-2.6(11)	3.2(12)	-2.4(11)
C13	25.0(13)	20.3(12)	37.4(15)	-0.5(11)	-1.5(11)	0.5(10)
C14	22.2(12)	20.5(12)	27.8(13)	-0.6(10)	-1(1)	0.4(10)
C15	22.2(12)	21.9(12)	29.9(13)	2.2(10)	-0.6(10)	1(1)
C16	31.1(14)	29.6(13)	27.6(14)	2.7(11)	2.8(11)	1.6(11)
C17	34.0(14)	30.4(14)	35.6(15)	8.4(12)	4.3(11)	-4.2(11)
C18	35.5(15)	19.9(12)	46.0(17)	6.4(12)	1.8(12)	-2.2(11)
C19	53.0(16)	19.2(12)	22.1(13)	-4.9(10)	4.3(11)	-4.5(11)
C20	47.2(16)	26.6(13)	22.0(13)	-3.3(11)	4.1(11)	-8.3(12)
C21	53.1(18)	34.6(15)	24.2(14)	-1.9(12)	0.7(12)	-7.6(13)
C22	51.4(18)	43.1(16)	28.6(15)	-6.8(13)	4.6(13)	-12.5(14)
C23	57.3(19)	41.2(16)	31.4(16)	1.6(13)	9.1(13)	-17.5(14)
C24	60.3(19)	27.3(13)	26.8(14)	1.4(11)	4.5(13)	-9.5(13)
C25	53(2)	73(2)	46.6(19)	2.1(17)	2.7(15)	-14.4(17)
S2	38.1(4)	45.7(4)	70.6(6)	-1.6(4)	-24.2(4)	-4.5(3)
O5	51.4(14)	54.2(13)	129(2)	-11.6(14)	-38.7(14)	-11.4(10)
O6	52.1(12)	59.1(13)	47.2(12)	1.8(9)	-21.7(9)	10.9(10)
O7	52.8(11)	20.6(9)	30.4(10)	2.4(7)	0.9(8)	2.9(8)
O8	51.4(12)	27.2(9)	40.4(11)	5.6(8)	-2.5(9)	6.7(8)
N2	35.5(13)	29.3(12)	57.8(16)	0.9(11)	-18.2(11)	-5.7(10)
C26	67(2)	42.8(17)	35.8(17)	3.2(13)	-1.6(14)	10.5(15)

C27	43.5(16)	20.3(12)	37.9(15)	3.1(11)	-5.6(12)	-2.1(11)
C28	48.4(16)	20.2(13)	24.0(13)	2.4(10)	-3.4(11)	0.2(11)
C29	45.5(16)	24.2(13)	37.3(15)	-1.2(12)	-14.4(12)	2.7(12)
C30	33.5(15)	26.7(13)	48.2(17)	-0.5(12)	-14.2(12)	-3.6(11)
C31	37.0(15)	21.6(12)	36.9(15)	4.5(11)	-8.3(12)	-0.8(11)
C32	33.1(14)	23.3(12)	30.8(14)	1.5(11)	-6.1(11)	-1.7(11)
C33	39.5(15)	25.0(13)	37.2(15)	6.6(11)	-9.3(12)	-5.5(11)
C34	32.2(14)	27.4(13)	33.3(15)	3.9(11)	-2.7(11)	1.0(11)
C35	57.4(19)	43.2(16)	36.8(16)	-3.3(13)	2.9(14)	-1.7(14)
C36	58.8(19)	41.2(16)	51.2(19)	18.8(14)	-1.0(15)	8.4(15)
C37	44.0(16)	27.4(13)	33.9(15)	6.6(11)	-0.4(12)	1.4(12)
C38	38.6(15)	23.3(12)	31.4(14)	-1.3(11)	8.0(11)	2.0(11)
C39	32.9(14)	21.5(12)	35.1(14)	-2.0(11)	2.0(11)	-0.2(11)
C40	36.3(15)	26.6(14)	45.8(17)	-4.9(12)	-2.3(12)	-1.5(12)
C41	36.7(15)	29.1(14)	55.8(19)	-13.6(13)	6.2(13)	-6.5(12)
C42	49.9(18)	23.0(13)	54.0(18)	-8.9(13)	23.1(15)	-10.8(13)
C43	51.6(18)	22.6(13)	39.8(16)	2.9(12)	15.1(13)	0.8(12)
C44	24.9(14)	55.4(19)	57.2(19)	24.9(15)	-13.6(13)	-8.9(13)
C45	31.4(17)	75(2)	92(3)	44(2)	-8.7(18)	-7.0(17)
C46	27.2(17)	123(4)	76(3)	67(3)	-5.7(17)	-12(2)
C47	29.6(17)	131(4)	35.4(18)	24(2)	-5.2(14)	-7(2)
C48	32.3(15)	78(2)	36.1(17)	6.1(16)	-4.5(13)	-11.5(15)
C49	28.0(14)	54.0(18)	38.3(17)	12.1(14)	-7.2(12)	-7.4(13)
C50	38.7(19)	234(6)	37(2)	8(3)	1.1(16)	-12(3)

Table 4 Bond Lengths for 0478

**Atom**	**Atom**	**Length/Å**	**Atom**	**Atom**	**Length/Å**
S1	O1	1.4288(17)	S2	O5	1.423(2)
S1	O2	1.4313(17)	S2	O6	1.432(2)
S1	N1	1.6474(19)	S2	N2	1.645(2)
S1	C19	1.754(3)	S2	C44	1.744(3)
O3	C3	1.205(3)	O7	C28	1.216(3)
O4	C1	1.412(3)	O8	C26	1.431(3)
O4	C2	1.417(3)	O8	C27	1.416(3)

N1	C5	1.509(3)	N2	C30	1.507(3)
N1	C15	1.441(3)	N2	C40	1.438(3)
C2	C3	1.526(3)	C27	C28	1.512(3)
C2	C8	1.535(3)	C27	C33	1.528(3)
C3	C4	1.527(3)	C28	C29	1.500(3)
C4	C5	1.513(3)	C29	C30	1.525(4)
C5	C6	1.511(3)	C30	C31	1.520(3)
C6	C7	1.334(3)	C31	C32	1.338(3)
C6	C14	1.440(3)	C31	C39	1.451(3)
C7	C8	1.497(3)	C32	C33	1.511(3)
C7	C9	1.520(3)	C32	C34	1.510(3)
C9	C12	1.543(3)	C34	C37	1.540(3)
C10	C12	1.530(4)	C35	C37	1.542(4)
C11	C12	1.523(3)	C36	C37	1.520(4)
C12	C13	1.528(3)	C37	C38	1.518(4)
C13	C14	1.391(3)	C38	C39	1.381(3)
C13	C18	1.389(3)	C38	C43	1.395(3)
C14	C15	1.384(3)	C39	C40	1.382(3)
C15	C16	1.389(3)	C40	C41	1.388(4)
C16	C17	1.389(3)	C41	C42	1.381(4)
C17	C18	1.395(4)	C42	C43	1.384(4)
C19	C20	1.388(3)	C44	C45	1.387(4)
C19	C24	1.393(3)	C44	C49	1.392(4)
C20	C21	1.378(4)	C45	C46	1.374(5)
C21	C22	1.396(4)	C46	C47	1.402(5)
C22	C23	1.387(4)	C47	C48	1.390(4)
C22	C25	1.510(4)	C47	C50	1.508(5)
C23	C24	1.376(4)	C48	C49	1.375(4)

Table 5 Bond Angles for 0478.

Atom	Atom	Atom	Angle/°	Atom	Atom	Atom	Angle/°
O1	S1	O2	121.00(10)	O5	S2	O6	120.30(14)
O1	S1	N1	106.63(10)	O5	S2	N2	106.25(12)
O1	S1	C19	108.15(11)	O5	S2	C44	109.11(16)
O2	S1	N1	105.42(10)	O6	S2	N2	106.25(12)

O2	S1	C19	108.18(11)	O6	S2	C44	107.39(13)
N1	S1	C19	106.62(10)	N2	S2	C44	106.80(12)
C1	O4	C2	114.15(18)	C27	O8	C26	112.95(19)
C5	N1	S1	116.49(14)	C30	N2	S2	117.50(16)
C15	N1	S1	121.60(15)	C40	N2	S2	122.19(18)
C15	N1	C5	109.17(17)	C40	N2	C30	109.29(19)
O4	C2	C3	111.10(19)	O8	C27	C28	113.8(2)
O4	C2	C8	105.14(18)	O8	C27	C33	107.18(19)
C3	C2	C8	112.49(19)	C28	C27	C33	109.2(2)
O3	C3	C2	120.9(2)	O7	C28	C27	120.2(2)
O3	C3	C4	120.1(2)	O7	C28	C29	120.6(2)
C2	C3	C4	118.9(2)	C29	C28	C27	118.7(2)
C5	C4	C3	113.39(19)	C28	C29	C30	105.9(2)
N1	C5	C4	112.93(18)	N2	C30	C29	112.1(2)
N1	C5	C6	103.38(17)	N2	C30	C31	103.19(19)
C6	C5	C4	111.53(18)	C31	C30	C29	114.7(2)
C7	C6	C5	130.9(2)	C32	C31	C30	132.2(2)
C7	C6	C14	121.8(2)	C32	C31	C39	120.5(2)
C14	C6	C5	107.34(19)	C39	C31	C30	107.0(2)
C6	C7	C8	125.8(2)	C31	C32	C33	126.2(2)
C6	C7	C9	116.9(2)	C31	C32	C34	117.2(2)
C8	C7	C9	117.04(19)	C34	C32	C33	116.4(2)
C7	C8	C2	116.33(19)	C32	C33	C27	115.4(2)
C7	C9	C12	116.32(19)	C32	C34	C37	117.6(2)
C10	C12	C9	107.8(2)	C34	C37	C35	108.7(2)
C11	C12	C9	108.7(2)	C36	C37	C34	109.2(2)
C11	C12	C10	109.4(2)	C36	C37	C35	109.1(2)
C11	C12	C13	112.4(2)	C38	C37	C34	110.2(2)
C13	C12	C9	111.53(19)	C38	C37	C35	107.2(2)
C13	C12	C10	106.9(2)	C38	C37	C36	112.4(2)
C14	C13	C12	116.3(2)	C39	C38	C37	116.1(2)
C18	C13	C12	127.2(2)	C39	C38	C43	116.6(2)
C18	C13	C14	116.0(2)	C43	C38	C37	127.1(2)
C13	C14	C6	125.0(2)	C38	C39	C31	125.5(2)
C15	C14	C6	111.58(19)	C38	C39	C40	123.2(2)
C15	C14	C13	123.1(2)	C40	C39	C31	111.2(2)

C14	C15	N1	108.33(19)	C39	C40	N2	108.9(2)
C14	C15	C16	120.8(2)	C39	C40	C41	120.0(2)
C16	C15	N1	130.6(2)	C41	C40	N2	131.0(2)
C17	C16	C15	116.3(2)	C42	C41	C40	117.3(2)
C16	C17	C18	122.7(2)	C41	C42	C43	122.6(2)
C13	C18	C17	120.8(2)	C42	C43	C38	120.3(2)
C20	C19	S1	118.75(19)	C45	C44	S2	120.5(3)
C20	C19	C24	120.3(2)	C45	C44	C49	119.4(3)
C24	C19	S1	120.9(2)	C49	C44	S2	120.0(2)
C21	C20	C19	119.6(2)	C46	C45	C44	119.3(4)
C20	C21	C22	120.8(2)	C45	C46	C47	122.1(3)
C21	C22	C25	120.6(3)	C46	C47	C50	121.8(4)
C23	C22	C21	118.5(3)	C48	C47	C46	117.6(3)
C23	C22	C25	120.9(3)	C48	C47	C50	120.6(4)
C24	C23	C22	121.5(3)	C49	C48	C47	120.8(3)
C23	C24	C19	119.2(2)	C48	C49	C44	120.8(3)

Table 6 Torsion Angles for 0478.

A	B	C	D	Angle/°	A	B	C	D	Angle/°
S1	N1	C5	C4	-93.8(2)	S2	N2	C30	C29	84.6(2)
S1	N1	C5	C6	145.54(15)	S2	N2	C30	C31	-151.53(19)
S1	N1	C15	C14	-144.74(17)	S2	N2	C40	C39	147.21(19)
S1	N1	C15	C16	40.8(3)	S2	N2	C40	C41	-36.5(4)
S1	C19	C20	C21	177.32(18)	S2	C44	C45	C46	179.7(2)
S1	C19	C24	C23	-176.9(2)	S2	C44	C49	C48	-179.9(2)
O1	S1	N1	C5	177.58(15)	O5	S2	N2	C30	-174.0(2)
O1	S1	N1	C15	-44.9(2)	O5	S2	N2	C40	45.8(3)
O1	S1	C19	C20	28.6(2)	O5	S2	C44	C45	-21.4(3)
O1	S1	C19	C24	-153.29(19)	O5	S2	C44	C49	158.2(2)
O2	S1	N1	C5	47.78(18)	O6	S2	N2	C30	-44.8(2)
O2	S1	N1	C15	-174.70(18)	O6	S2	N2	C40	175.0(2)
O2	S1	C19	C20	161.28(18)	O6	S2	C44	C45	-153.4(2)
O2	S1	C19	C24	-20.6(2)	O6	S2	C44	C49	26.3(2)
O3	C3	C4	C5	-178.7(2)	O7	C28	C29	C30	85.5(3)
O4	C2	C3	O3	11.4(3)	O8	C27	C28	O7	152.9(2)

O4	C2	C3	C4	-168.69(18)	O8	C27	C28	C29	-35.7(3)
O4	C2	C8	C7	176.13(19)	O8	C27	C33	C32	58.3(3)
N1	S1	C19	C20	-85.8(2)	N2	S2	C44	C45	93.0(2)
N1	S1	C19	C24	92.4(2)	N2	S2	C44	C49	-87.4(2)
N1	C5	C6	C7	177.4(2)	N2	C30	C31	C32	-166.2(3)
N1	C5	C6	C14	-0.6(2)	N2	C30	C31	C39	6.5(3)
N1	C15	C16	C17	172.0(2)	N2	C40	C41	C42	-175.3(3)
C1	O4	C2	C3	77.3(2)	C26	O8	C27	C28	-75.5(3)
C1	O4	C2	C8	-160.8(2)	C26	O8	C27	C33	163.6(2)
C2	C3	C4	C5	1.5(3)	C27	C28	C29	C30	-85.8(3)
C3	C2	C8	C7	-62.8(3)	C28	C27	C33	C32	-65.4(3)
C3	C4	C5	N1	177.24(18)	C28	C29	C30	N2	-175.6(2)
C3	C4	C5	C6	-66.8(2)	C28	C29	C30	C31	67.2(3)
C4	C5	C6	C7	55.7(3)	C29	C30	C31	C32	-44.0(4)
C4	C5	C6	C14	-122.2(2)	C29	C30	C31	C39	128.7(2)
C5	N1	C15	C14	-4.5(2)	C30	N2	C40	C39	4.2(3)
C5	N1	C15	C16	-179.0(2)	C30	N2	C40	C41	-179.5(3)
C5	C6	C7	C8	-0.2(4)	C30	C31	C32	C33	-5.1(5)
C5	C6	C7	C9	174.2(2)	C30	C31	C32	C34	170.5(3)
C5	C6	C14	C13	172.1(2)	C30	C31	C39	C38	178.5(2)
C5	C6	C14	C15	-2.2(3)	C30	C31	C39	C40	-4.4(3)
C6	C7	C8	C2	4.9(4)	C31	C32	C33	C27	48.2(4)
C6	C7	C9	C12	32.2(3)	C31	C32	C34	C37	27.2(3)
C6	C14	C15	N1	4.2(3)	C31	C39	C40	N2	0.2(3)
C6	C14	C15	C16	179.3(2)	C31	C39	C40	C41	-176.5(2)
C7	C6	C14	C13	-6.1(4)	C32	C31	C39	C38	-7.7(4)
C7	C6	C14	C15	179.6(2)	C32	C31	C39	C40	169.3(2)
C7	C9	C12	C10	76.2(3)	C32	C34	C37	C35	75.3(3)
C7	C9	C12	C11	-165.4(2)	C32	C34	C37	C36	-165.8(2)
C7	C9	C12	C13	-40.9(3)	C32	C34	C37	C38	-41.9(3)
C8	C2	C3	O3	-106.1(2)	C33	C27	C28	O7	-87.4(3)
C8	C2	C3	C4	73.7(3)	C33	C27	C28	C29	84.0(3)
C8	C7	C9	C12	-152.9(2)	C33	C32	C34	C37	-156.7(2)
C9	C7	C8	C2	-169.4(2)	C34	C32	C33	C27	-127.4(2)
C9	C12	C13	C14	27.2(3)	C34	C37	C38	C39	32.5(3)
C9	C12	C13	C18	-160.9(2)	C34	C37	C38	C43	-153.5(2)

C10	C12	C13	C14	-90.4(2)	C35	C37	C38	C39	-85.6(3)
C10	C12	C13	C18	81.5(3)	C35	C37	C38	C43	88.4(3)
C11	C12	C13	C14	149.6(2)	C36	C37	C38	C39	154.5(2)
C11	C12	C13	C18	-38.5(3)	C36	C37	C38	C43	-31.5(4)
C12	C13	C14	C6	-4.9(3)	C37	C38	C39	C31	-9.8(4)
C12	C13	C14	C15	168.8(2)	C37	C38	C39	C40	173.5(2)
C12	C13	C18	C17	-171.6(2)	C37	C38	C43	C42	-173.5(2)
C13	C14	C15	N1	-170.2(2)	C38	C39	C40	N2	177.3(2)
C13	C14	C15	C16	4.8(3)	C38	C39	C40	C41	0.6(4)
C14	C6	C7	C8	177.5(2)	C39	C31	C32	C33	-177.1(2)
C14	C6	C7	C9	-8.1(3)	C39	C31	C32	C34	-1.5(4)
C14	C13	C18	C17	0.3(3)	C39	C38	C43	C42	0.5(4)
C14	C15	C16	C17	-1.8(3)	C39	C40	C41	C42	0.6(4)
C15	N1	C5	C4	123.7(2)	C40	N2	C30	C29	-130.5(2)
C15	N1	C5	C6	3.1(2)	C40	N2	C30	C31	-6.6(3)
C15	C16	C17	C18	-1.7(4)	C40	C41	C42	C43	-1.3(4)
C16	C17	C18	C13	2.5(4)	C41	C42	C43	C38	0.7(4)
C18	C13	C14	C6	-177.7(2)	C43	C38	C39	C31	175.6(2)
C18	C13	C14	C15	-4.0(3)	C43	C38	C39	C40	-1.2(4)
C19	S1	N1	C5	-67.06(18)	C44	S2	N2	C30	69.6(2)
C19	S1	N1	C15	70.5(2)	C44	S2	N2	C40	-70.6(2)
C19	C20	C21	C22	-0.7(4)	C44	C45	C46	C47	-0.2(5)
C20	C19	C24	C23	1.2(4)	C45	C44	C49	C48	-0.3(4)
C20	C21	C22	C23	1.9(4)	C45	C46	C47	C48	0.4(5)
C20	C21	C22	C25	-176.1(2)	C45	C46	C47	C50	-179.5(3)
C21	C22	C23	C24	-1.6(4)	C46	C47	C48	C49	-0.6(4)
C22	C23	C24	C19	0.0(4)	C47	C48	C49	C44	0.5(4)
C24	C19	C20	C21	-0.8(3)	C49	C44	C45	C46	0.1(4)
C25	C22	C23	C24	176.5(3)	C50	C47	C48	C49	179.3(3)

Home Sessions People Exhibitors More Signup Login

https://www.morressier.com/o/event/5fc642c603137aa525863c7c/article/5fc643a32d78d1fec466981a

SUBMISSION

[4+3] Cycloaddition reactions of 3-alkenyl indoles

Ferdinand Taenzler, Jiasu Xu, Rawal Viresh H

0 views

0 downloads

Video

PDF

Abstract

0 Datasets

PRESENTED AT

2019 Spring National Meeting

Mar 27-Apr 3, 2019

EXPLORE MORE CONTENT FROM

American Chemical Society

Abstract

The cyclohepta[B]indole motif is prevalent in a variety of pharmaceutical agents and natural products, the most striking of which are the ambiguine family of natural products. Discussed will be a synthetic methodology which seeks to provide direct access to a variety of cyclohepta[B]indoles and their derivatives, their precursors, and a possible synthetic solution to the ambiguine skeleton.

By applying the [4+3] cycloaddition reaction of oxyallyl cations and 3-alkenyl indoles we present a possible solution to the formation of this challenging motif. This novel reaction furnishes the cyclohepta[B]indole in high yield and diastereoselectivity. In addition, a possible solution for the synthesis of the ambiguine skeleton will be shown as showcase of the methodology and its potential potency in total synthesis. This work is in complement to a separate [4+3] approach to the ambiguines currently under investigation by our group.
